# Supplementary material for: Mitochondrial Transplantation Promotes Protective Effector and Memory CD4+ T Cell Response During Mycobacterium Tuberculosis Infection and Diminishes Exhaustion and Senescence in Elderly CD4+ T cells
Source: Adv Sci (Weinh). 2024 Jul 22;11(36):2401077. doi: 10.1002/advs.202401077 (PMC11423092; doi:10.1002/advs.202401077)
Supplement: Supplementary file 1 — Supporting Information [file ADVS-11-2401077-s001.pdf]

ADVANCED SCIENCE
Open Access

Supporting Information

for *Adv. Sci.*, DOI 10.1002/advs.202401077

Mitochondrial Transplantation Promotes Protective Effector and Memory CD4$^+$ T Cell Response During *Mycobacterium Tuberculosis* Infection and Diminishes Exhaustion and Senescence in Elderly CD4$^+$ T cells

Colwyn A. Headley, Shalini Gautam, Angelica Olmo-Fontanez, Andreu Garcia-Vilanova, Varun Dwivedi, Alyssa Schami, Susan Weintraub, Philip S. Tsao, Jordi B. Torrelles and Joanne Turner**

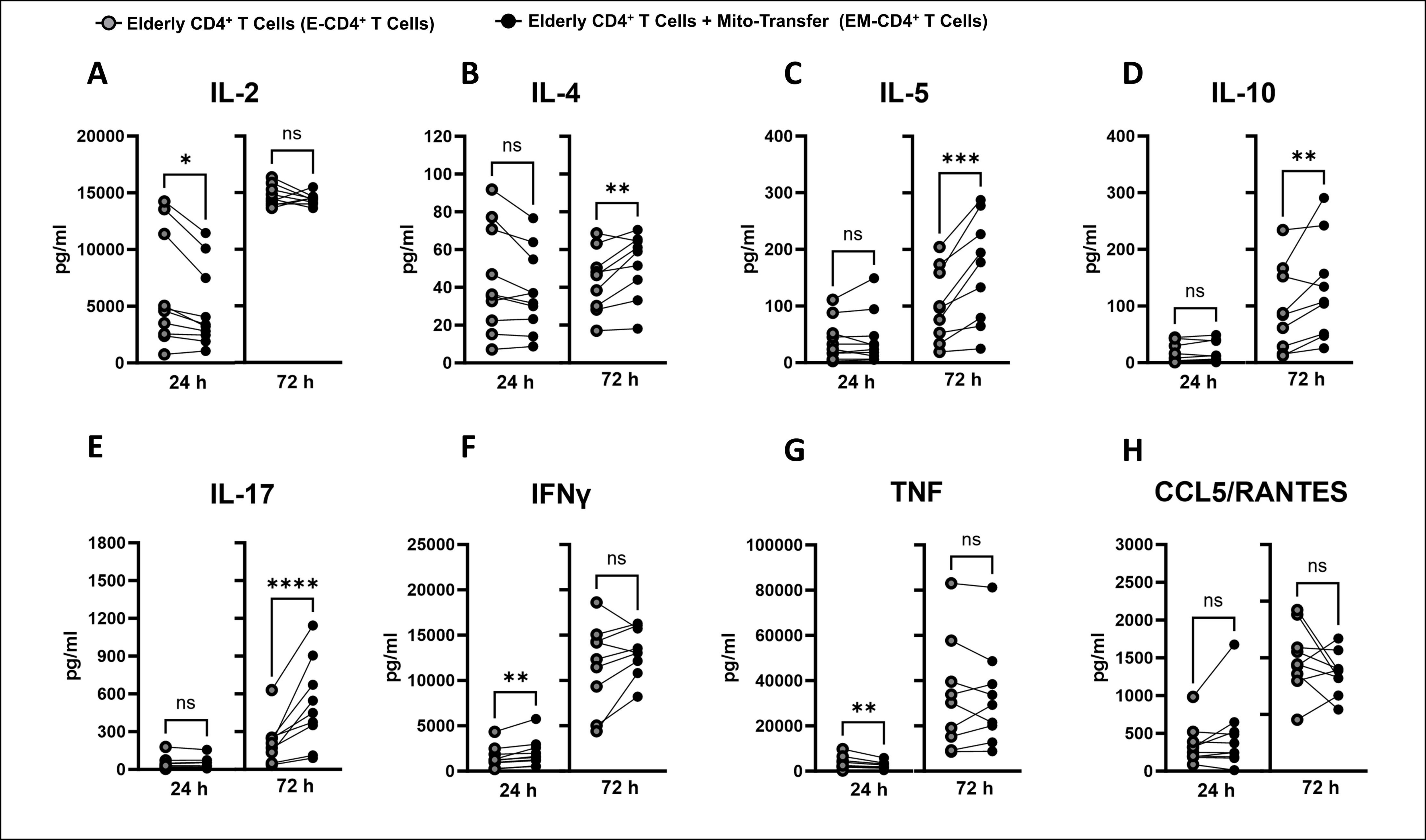

Elderly CD4+ T Cells (E-CD4+ T Cells)
Elderly CD4+ T Cells + Mito-Transfer (EM-CD4+ T Cells)
A
IL-2
B
IL-4
C
IL-5
D
IL-10
E
IL-17
F
IFNγ
G
TNF
H
CCL5/RANTES
pg/ml
24 h
72 h
ns
*
**
***
****

Supp. Fig. 1. Mito-transfer alters cytokine production of CD4+ T cells in aged humans. CD4+ T cells from aged humans with or without mito-transfer were stimulated with PMA/Ionomycin. After 24h and 72h stimulation with PMA/Ionomycin, the supernatants were examined by Luminex array for cytokines produced. 9-10 biological replicates per group with $p \leq 0.05$ = *, $p \leq 0.01$ = **, or $p \leq 0.001$ = *** using paired Student's *t*-test.

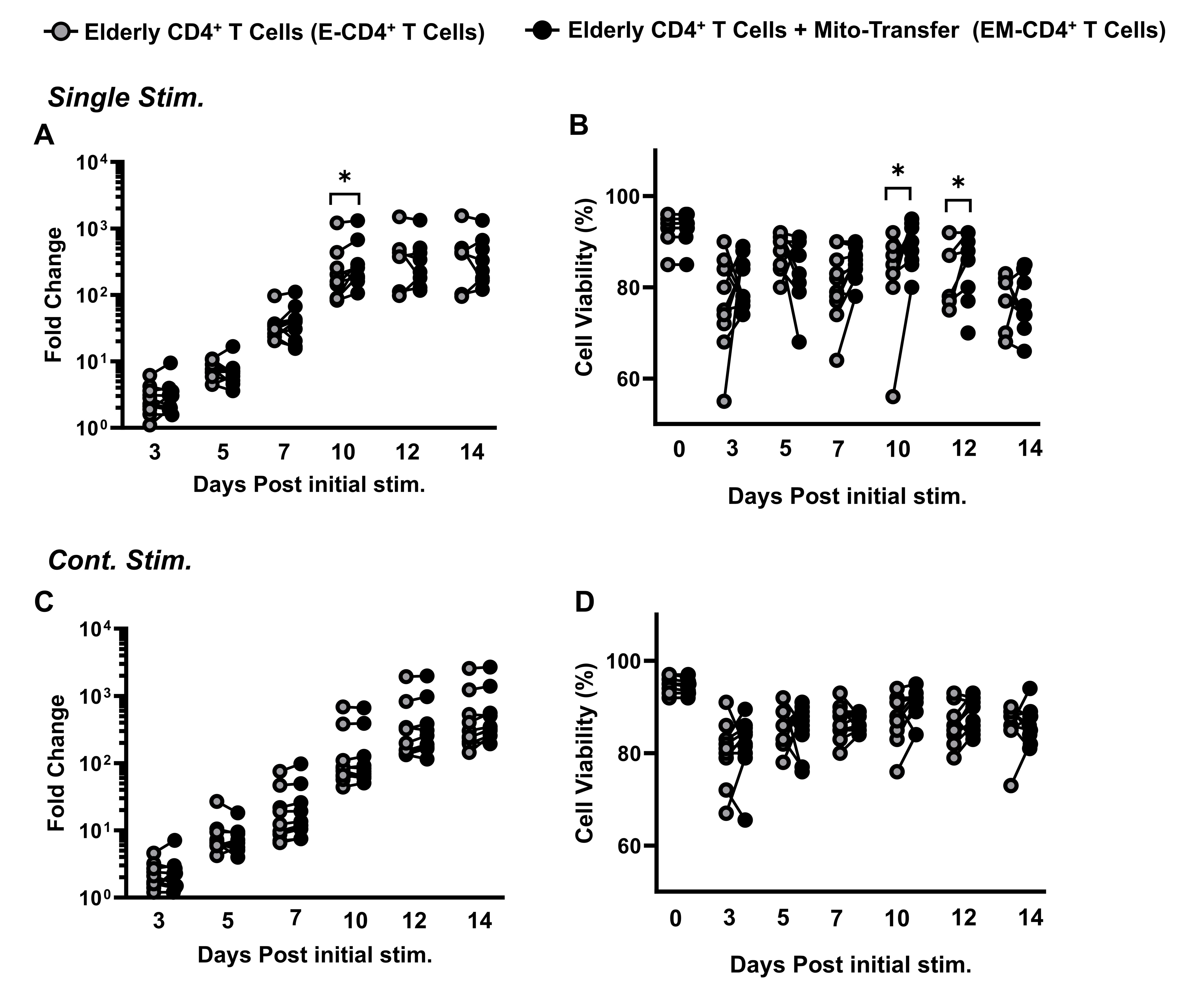
Elderly CD4+ T Cells (E-CD4+ T Cells)
Elderly CD4+ T Cells + Mito-Transfer (EM-CD4+ T Cells)
Single Stim.
A
Fold Change
Days Post initial stim.
B
Cell Viability (%)
Days Post initial stim.
Cont. Stim.
C
Fold Change
Days Post initial stim.
D
Cell Viability (%)
Days Post initial stim.

Supp. Fig 2. Impact of mito-transfer on elderly T cell proliferation and viability. Elderly CD4 T cells with or without mito-transfer were activated via surface cross-linking (CD3/CD28). CD4 T cells either received a single (single) or continuous (cont.) stimulation. **A)** Fold change and **B)** viability of CD4+ T cells with or without mito-transfer after single stimulation. **C)** Fold change and **D)** viability of CD4+ T cells with or without mito-transfer with continuous stimulation. 10 biological replicates per group, with $p \leq 0.05$ = *, $p \leq 0.01$ = **, using paired Student's *t*-test.

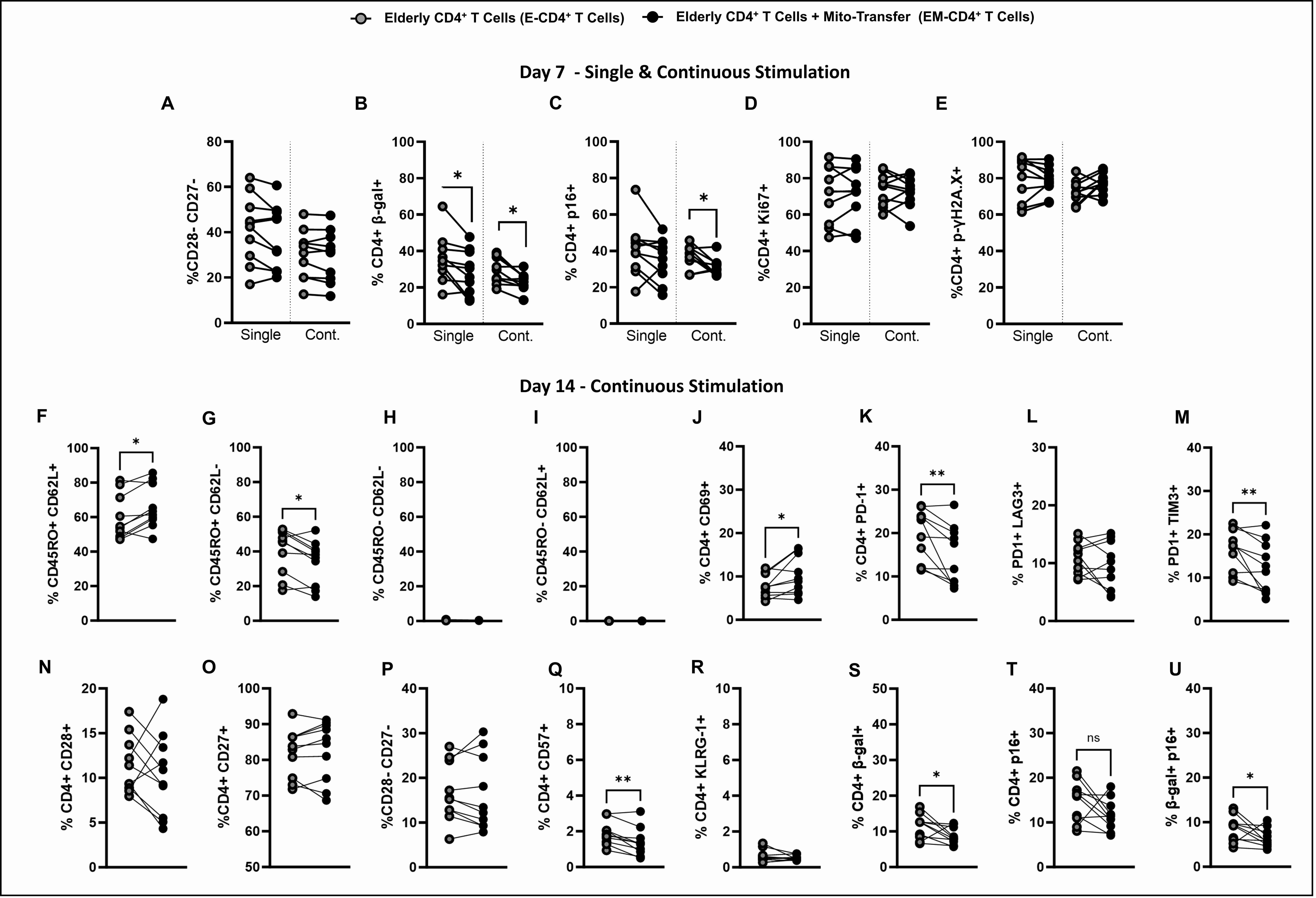

Elderly CD4⁺ T Cells (E-CD4⁺ T Cells)
Elderly CD4⁺ T Cells + Mito-Transfer (EM-CD4⁺ T Cells)
Day 7 - Single & Continuous Stimulation
A
%CD28- CD27-
B
% CD4+ β-gal+
C
% CD4+ p16+
D
%CD4+ Ki67+
E
%CD4+ p-γH2A.X+
Single
Cont.
Day 14 - Continuous Stimulation
F
% CD45RO+ CD62L+
G
% CD45RO+ CD62L-
H
% CD45RO- CD62L-
I
% CD45RO- CD62L+
J
% CD4+ CD69+
K
% CD4+ PD-1+
L
% PD1+ LAG3+
M
% PD1+ TIM3+
N
% CD4+ CD28+
O
%CD4+ CD27+
P
%CD28- CD27-
Q
% CD4+ CD57+
R
% CD4+ KLRG-1+
S
% CD4+ β-gal+
T
% CD4+ p16+
U
% β-gal+ p16+
ns

Supp Fig 3. Mito-transfer improves T cell activation and reduces human T cell exhaustion and senescence. Donor mitochondria isolated from primary human neonatal dermal fibroblasts were transplanted into CD4+ T cells from elderly humans. Elderly $CD4^+$ T cells with or without mito-transfer were activated via surface cross-linking (CD3/CD28), T cells either received a single (single) or continuous (cont.) stimulation. The percentages of **A)** CD28- CD27, **B)** β-gal+, **C)** p16+, **D)** Ki67+, and **E**) ɣH2A.X+ human CD4+ cells at 7 days after intial activation with CD3/CD28/CD2. The percentages of **F**) CD45RO+ CD62L+, **G**) CD45RO+ CD62L , **H**) CD45RO- CD62L-, **I**) CD45RO- CD62L-, **J**) CD69+, **K**) PD-1+, **L**) PD-1+ LAG3+, **M**) PD-1+ TIM3+, **N**) CD28+, **O**) CD27+, **P**) CD28- CD27 , **Q**) CD57+, **R**) KLRG1+, and **S**) β-Gal+, **T**) p16+, **U**) β-gal+ p16+ human CD4+ T cells at 14 days after continuous stimulation with CD3/CD28/CD2. 10 biological replicates per group, with $p \leq 0.05$ = *, $p \leq 0.01$ = **, using paired Student's t-test.

Uniprot ID	Gene Symbol	t-test	FDR (adj. P-val.)	O1	O2	O3	OM1	OM2	OM3
P35564	Canx	0.000	0.009	0.244	0.000	-0.086	1.700	1.720	1.860
P56959	Fus	0.739	1.000	-0.231	0.140	0.000	-0.070	-0.107	0.255
Q01147	Creb1	0.091	0.232	-0.101	0.000	0.267	-0.384	-0.333	-0.078
B2RQC6	Cad	0.675	0.952	0.244	0.000	-0.142	0.662	-0.451	-0.695
O09117	Sypl1	0.366	0.613	0.244	0.000	-0.323	0.744	-0.281	0.538
Q9JIK9	Mrps34	0.049	0.154	0.000	0.757	-0.497	1.210	1.140	1.010
Q8K1Z0	Coq9	0.032	0.117	0.000	0.186	-0.023	0.862	0.388	0.472
P12265	Gusb	0.016	0.079	0.311	0.000	-0.297	0.822	0.616	0.837
P0C0S6	H2afz	0.384	0.633	-0.205	0.000	0.096	-0.364	-0.361	0.097
Q3THW5	H2afv	0.384	0.633	-0.205	0.000	0.096	-0.364	-0.361	0.097
Q8BM55	Tmem214	0.001	0.020	0.000	0.450	-0.101	1.560	1.490	1.600
P70227	Itpr3	0.573	0.843	-0.451	0.154	0.000	0.017	-0.052	0.078
O08579	Emd	0.292	0.518	-0.552	0.000	0.097	0.094	-0.100	0.655
Q9CQ60	Pgls	0.384	0.633	0.000	1.190	-0.787	-0.304	-2.870	0.284
Q8K2K6	Agfg1	0.896	1.000	-0.030	0.000	1.700	0.753	0.090	1.090
P97450	Atp5j	0.033	0.121	0.000	0.125	-0.630	1.010	0.420	1.140
O55022	Pgrmc1	0.000	0.010	0.000	-0.086	0.009	1.480	1.240	1.540
Q9D0K2	Oxct1	0.000	0.012	0.000	0.092	-0.243	1.760	1.440	1.670
Q3UZ39	Lrrfip1	0.003	0.030	0.000	-0.058	0.148	-0.914	-0.930	-0.588
P52825	Cpt2	0.312	0.545	0.000	0.208	-0.370	1.210	0.043	0.051
P70460	Vasp	0.139	0.305	0.006	-0.256	0.000	-0.109	-0.539	-0.494
Q62422	Ostf1	0.025	0.102	0.488	-0.040	0.000	-0.345	-0.835	-0.846
Q8BMK4	Ckap4	0.000	0.010	0.093	0.000	-0.598	3.600	3.310	3.710
Q8BFY9	Tnpo1	0.030	0.112	-0.077	0.000	0.000	-0.433	-1.300	-0.849
Q9D6J6	Ndufv2	0.049	0.154	0.000	0.342	-0.183	0.718	0.363	0.856
P51125	Cast	0.847	1.000	-0.076	0.000	0.118	0.232	-0.209	-0.068
P06800	Ptprc	0.199	0.394	-0.090	0.130	0.000	-0.302	-0.348	0.046
Q9Z277	Baz1b	0.955	1.000	-0.231	0.238	0.000	0.030	-0.197	0.206
Q9CWL8	Ctnnbl1	0.003	0.033	0.000	0.296	-0.044	-0.669	-1.050	-0.968
Q7TPR4	Actn1	0.680	0.956	0.455	0.000	-0.439	0.662	-0.251	0.099
P35979	Rpl12	0.491	0.755	0.266	-0.151	0.000	0.271	-0.094	0.359
O09167	Rpl21	0.336	0.573	0.282	-1.900	0.000	0.415	-0.668	2.110
Q8R081	Hnrnpl	0.025	0.101	-0.126	0.000	0.040	-0.348	-0.636	-0.308
P70315	Was	0.034	0.122	0.000	-0.169	0.494	-0.488	-1.180	-0.715
Q91YH5	Atl3	0.038	0.130	-0.218	0.589	0.000	0.827	0.935	0.843
P14824	Anxa6	0.623	0.898	-0.261	0.274	0.000	-0.273	-0.143	0.121
Q99L45	Eif2s2	0.540	0.807	0.342	-0.259	0.000	0.266	-0.631	-0.179
Q9Z2D6	Mecp2	0.063	0.181	-0.130	0.000	0.020	-0.393	-0.654	-0.177
Q9D1A2	Cndp2	0.006	0.043	0.000	0.087	-0.305	-0.761	-1.170	-1.080
Q6IRU2	Tpm4	0.699	0.975	0.000	0.258	-0.242	0.139	-0.352	-0.025
Q9JKF1	Iqgap1	0.611	0.885	0.080	0.000	-0.164	0.186	-0.420	-0.164
O08734	Bak1	0.010	0.060	0.000	0.028	-0.547	0.687	0.764	0.655
Q9DBJ1	Pgam1	0.020	0.088	0.582	-0.030	0.000	-0.473	-1.080	-1.040
P24270	Cat	0.046	0.148	0.177	0.000	-0.253	1.040	0.403	0.494
P47757	Capzb	0.016	0.077	0.000	0.178	-0.196	-0.410	-0.725	-0.604
Q8BK67	Rcc2	0.253	0.466	0.487	-0.092	0.000	0.079	-0.473	-0.172
Q8C0G2	Traf3ip3	0.025	0.100	-0.492	0.050	0.000	-0.704	-0.767	-0.938
Q9CYN2	Spcs2	0.069	0.192	0.000	0.270	-0.026	0.525	0.226	0.467
Q9D0F9	Pgm1	0.000	0.014	-0.069	0.000	0.150	-1.850	-2.270	-1.690
Q5SUA5	Myo1g	0.101	0.247	0.000	-0.027	0.074	-0.171	-0.701	-0.196
Q8BJZ4	Mrps35	0.007	0.051	0.062	0.000	-0.636	1.340	1.050	0.901
Q920B9	Supt16h	0.019	0.087	0.022	0.000	-0.102	-0.331	-0.845	-0.661
D3Z7P3	Gls	0.500	0.765	0.000	0.006	-0.037	0.732	-0.191	0.043
Q8BRG8	Tmem209	0.181	0.368	0.000	-1.610	0.980	0.868	1.100	1.090
Q9DBE8	Alg2	0.000	0.012	0.004	0.000	-0.115	0.889	0.810	1.040
Q9JIX8	Acin1	0.008	0.051	-0.012	0.228	0.000	-0.410	-0.622	-0.382
Q9JI39	Abcb10	0.138	0.303	0.860	-0.266	0.000	1.480	0.456	1.190

O55201	Supt5h	0.014	0.073	0.000	-0.043	0.190	-0.500	-1.070	-0.621
P47754	Capza2	0.597	0.870	0.242	-0.135	0.000	-0.200	-0.355	0.281
Q9R190	Mta2	0.043	0.142	-0.073	0.000	0.242	-0.313	-0.546	-0.196
Q99PL5	Rrbp1	0.476	0.736	0.000	0.054	-0.281	0.493	-0.283	0.146
Q6DFW4	Nop58	0.101	0.246	0.101	-0.080	0.000	-0.065	-0.661	-0.403
P26041	Msn	0.350	0.592	0.047	0.000	-0.054	0.016	-0.303	-0.044
Q9DCT5	Sdf2	0.001	0.020	-0.108	0.000	0.394	2.260	1.720	1.880
Q9CQJ8	Ndufb9	0.003	0.033	-0.196	0.220	0.000	0.740	0.744	0.811
P97742	Cpt1a	0.015	0.076	0.000	0.296	-0.452	0.925	0.756	0.903
Q9JKR6	Hyou1	0.008	0.053	0.225	0.000	-0.131	1.020	0.600	0.825
P13020	Gsn	0.025	0.102	0.242	0.000	-0.363	0.994	0.554	0.643
P11087	Col1a1	0.000	0.002	0.000	0.029	-0.359	7.740	7.340	7.640
P62264	Rps14	0.453	0.711	0.151	-0.252	0.000	0.120	-0.562	-0.229
P70441	Slc9a3r1	0.003	0.033	0.000	-0.076	0.282	-1.040	-1.680	-1.200
Q9D4J7	Phf6	0.051	0.158	0.066	-0.328	0.000	-0.462	-1.120	-0.611
P47738	Aldh2	0.815	1.000	0.398	0.000	-0.923	0.773	-0.269	-0.602
Q00PI9	Hnrnpul2	0.026	0.104	-0.040	0.228	0.000	-0.233	-0.569	-0.346
Q8VE37	Rcc1	0.021	0.092	-0.073	0.000	0.088	-0.349	-0.451	-0.184
Q62351	Tfrc	0.848	1.000	1.330	0.000	-0.491	1.130	-0.397	-0.350
Q9Z2I8	Suclg2	0.566	0.835	0.026	0.000	-0.159	0.510	-0.235	0.016
Q6P5E4	Uggt1	0.004	0.038	0.000	0.111	-0.056	0.592	0.431	0.718
P22892	Ap1g1	0.026	0.103	0.208	-0.088	0.000	-0.441	-1.260	-1.380
P68404	Prkcb	0.078	0.209	0.000	0.147	-0.418	-0.334	-0.753	-0.690
Q9D554	Sf3a3	0.380	0.629	-0.549	0.464	0.000	-1.380	-0.697	0.305
Q9DCX2	Atp5h	0.025	0.101	0.000	0.466	-0.336	1.070	1.120	0.719
P20152	Vim	0.215	0.412	-0.323	0.000	0.034	0.296	-0.083	0.213
Q3THK7	Gmps	0.013	0.069	0.144	0.000	-0.093	-0.611	-1.310	-0.837
P35550	Fbl	0.028	0.108	0.000	0.208	-0.044	-0.216	-0.596	-0.384
Q8VE22	Mrps23	0.006	0.044	-0.054	0.132	0.000	0.889	0.501	0.810
P60229	Eif3e	0.509	0.773	0.118	0.000	-0.169	0.147	-0.427	-0.174
Q569Z6	Thrap3	0.018	0.083	0.000	-0.095	0.105	-0.329	-0.780	-0.538
P50396	Gdi1	0.000	0.008	-0.087	0.000	0.016	-1.240	-1.480	-1.310
Q9DAR7	Dcps	0.002	0.027	0.000	-0.191	0.360	-1.520	-1.940	-2.320
Q02053	Uba1	0.001	0.019	-0.136	0.000	0.019	-0.926	-1.240	-1.310
Q99L43	Cds2	0.069	0.191	0.096	0.000	-0.041	0.400	0.094	0.473
P68373	Tuba1c	0.757	1.000	-0.157	0.382	0.000	0.181	-0.134	-0.005
Q8R550	Sh3kbp1	0.065	0.185	-0.456	0.012	0.000	-0.738	-0.809	-0.400
Q64511	Top2b	0.552	0.820	-0.077	0.020	0.000	-0.131	-0.265	0.115
Q62418	Dbnl	0.002	0.028	-0.131	0.000	0.056	-1.040	-1.690	-1.410
Q9D404	Oxsm	0.917	1.000	0.000	0.611	-0.220	0.234	0.026	0.046
Q8BPU7	Elmo1	0.003	0.033	-0.217	0.000	0.110	-0.699	-0.889	-0.683
Q99JB2	Stoml2	0.007	0.048	0.000	0.206	-0.196	0.991	0.626	0.828
P11499	Hsp90ab1	0.018	0.084	0.026	0.000	-0.037	-0.346	-0.932	-0.757
P21619	Lmnb2	0.066	0.186	-0.179	0.000	0.032	-0.265	-0.402	-0.177
Q6NZC7	Sec23ip	0.136	0.301	0.293	0.000	-0.031	-0.215	-1.310	-0.279
Q922R8	Pdia6	0.018	0.083	0.000	0.091	-0.433	1.380	1.200	0.553
P49710	Hcls1	0.004	0.034	-0.319	0.000	0.235	-1.270	-1.490	-1.060
Q9CY58	Serbp1	0.592	0.864	-0.242	0.000	0.112	0.215	-0.198	0.139
O89090	Sp1	0.189	0.379	0.000	1.060	-0.104	-0.224	-0.256	-0.338
P59017	Bcl2l13	0.118	0.274	0.000	-0.077	0.194	-0.067	-0.784	-0.362
Q9JI13	Utp3	0.366	0.612	1.970	-0.999	0.000	1.660	0.754	1.350
P27659	Rpl3	0.218	0.417	0.230	-0.104	0.000	0.727	0.075	0.273
P97807	Fh	0.048	0.152	0.000	0.319	-0.305	0.689	0.384	0.857
P11983	Tcp1	0.019	0.087	0.010	-0.033	0.000	-0.369	-0.934	-0.590
Q9QY81	Nup210	0.079	0.209	0.000	0.154	-0.034	-0.133	-0.253	-0.062
Q9EQP2	Ehd4	0.227	0.429	0.000	0.001	-0.845	0.451	0.115	-0.049
Q8R010	Aimp2	0.173	0.357	0.013	0.000	-0.531	-0.551	-1.030	-0.312
Q8CBY8	Dctn4	0.037	0.129	0.000	-0.092	0.128	-0.321	-0.625	-0.243
Q3TBT3	Tmem173	0.649	0.925	-0.395	0.164	0.000	-0.291	-0.323	0.074

P14733	Lmnb1	0.060	0.175	-0.138	0.000	0.107	-0.348	-0.608	-0.184
P17225	Ptbp1	0.985	1.000	-0.053	0.090	0.000	-0.272	-0.102	0.336
P61979	Hnrnpk	0.004	0.034	-0.236	0.011	0.000	-0.622	-0.888	-0.763
P46935	Nedd4	0.009	0.057	1.270	0.000	-0.301	3.520	2.530	2.910
Q99KV1	Dnajb11	0.002	0.025	0.038	0.000	-0.045	0.752	0.934	1.200
Q9JJA4	Wdr12	0.164	0.343	0.164	0.000	-0.215	-0.313	-0.653	-0.091
Q9JHU4	Dync1h1	0.241	0.450	0.000	0.014	-0.111	0.549	-0.014	0.098
Q5PSV9	Mdc1	0.020	0.088	0.000	-0.100	0.030	-0.538	-0.554	-0.234
P07356	Anxa2	0.002	0.028	-0.294	0.146	0.000	0.920	0.824	1.030
Q6PDM2	Srsf1	0.015	0.076	0.288	0.000	-0.003	-0.389	-0.976	-0.902
Q9Z1Q9	Vars	0.018	0.083	0.137	-0.048	0.000	-0.459	-1.210	-0.842
P22646	Cd3e	0.501	0.766	-0.349	0.000	0.371	-0.475	-0.277	0.156
Q923D5	Wbp11	0.022	0.095	-0.054	0.000	0.115	-0.351	-0.733	-0.346
O08583	Alyref	0.509	0.773	-0.288	0.193	0.000	-0.401	0.188	1.140
Q8BGH2	Samm50	0.000	0.013	0.000	0.083	-0.194	0.973	0.871	0.934
Q99KK7	Dpp3	0.000	0.013	-0.119	0.102	0.000	-1.230	-1.360	-1.600
P16546	Sptan1	0.055	0.165	-0.057	0.095	0.000	0.323	0.097	0.320
Q8VIJ6	Sfpq	0.012	0.066	-0.229	0.123	0.000	-0.574	-0.596	-0.442
Q9CZR8	Tsfm	0.707	0.983	0.245	0.000	-0.207	0.426	-0.475	-0.282
Q9D1J3	Sarnp	0.287	0.512	-0.551	0.000	0.172	-0.814	-0.430	-0.184
P23116	Eif3a	0.339	0.578	0.109	0.000	-0.242	0.055	-0.402	-0.373
Q8K4Z5	Sf3a1	0.029	0.110	-0.065	0.185	0.000	-0.670	-0.424	-0.241
Q9WVA3	Bub3	0.226	0.429	-0.180	0.000	0.110	-0.149	-0.518	-0.085
Q8BFW7	Lpp	0.000	0.009	0.000	0.546	-0.074	4.720	4.080	4.430
Q3TZZ7	Esyt2	0.004	0.037	-0.192	0.000	0.048	0.458	0.367	0.491
P80314	Cct2	0.030	0.113	-0.001	0.000	0.055	-0.220	-0.792	-0.640
Q6PB66	Lrpprc	0.035	0.125	0.000	0.121	-0.292	0.941	0.428	0.430
E9PVX6	Mki67	0.500	0.765	0.712	-0.541	0.000	0.430	-0.788	-0.647
Q7TSV4	Pgm2	0.000	0.013	0.000	-0.203	0.179	-1.780	-2.280	-2.070
Q9R0P3	Esd	0.086	0.222	0.000	0.047	-0.224	-0.212	-0.757	-0.429
Q9D824	Fip1l1	0.562	0.831	-0.526	0.000	0.347	-0.360	-0.548	0.118
Q8CI51	Pdlim5	0.002	0.025	0.018	0.000	-0.246	2.180	1.340	1.770
Q99MR6	Srrt	0.012	0.069	0.089	-0.063	0.000	-0.600	-1.420	-1.250
P55194	Sh3bp1	0.018	0.083	-0.199	0.000	0.014	-0.550	-1.070	-0.614
Q922P9	Glyr1	0.200	0.394	-0.174	0.000	0.112	-0.349	-0.510	0.002
Q9D819	Ppa1	0.012	0.069	0.055	-0.145	0.000	-1.050	-1.690	-0.821
Q9QUM0	Itga2b	0.351	0.592	0.000	0.515	-0.869	-0.489	-0.209	-1.310
Q80U93	Nup214	0.291	0.518	-0.291	0.118	0.000	-0.193	-0.509	-0.103
Q8BVY0	Rsl1d1	0.050	0.155	0.000	-0.234	0.038	-0.355	-1.110	-0.679
O09106	Hdac1	0.745	1.000	-0.195	0.107	0.000	-0.161	-0.227	0.147
O09111	Ndufb11	0.001	0.018	0.019	-0.197	0.000	0.578	0.575	0.648
Q9D6R2	Idh3a	0.546	0.814	0.000	0.280	-0.166	0.748	-0.097	0.040
Q80WJ7	Mtdh	0.233	0.439	0.000	-0.069	0.038	0.134	-0.029	0.382
Q60611	Satb1	0.855	1.000	0.000	-0.019	0.104	0.646	-1.180	0.293
P40124	Cap1	0.001	0.015	0.000	0.284	-0.079	-1.070	-1.130	-1.270
Q9ERU9	Ranbp2	0.147	0.315	0.000	-0.026	0.041	0.012	-0.328	-0.218
P08113	Hsp90b1	0.004	0.035	0.176	0.000	-0.151	1.090	0.696	0.949
P63024	Vamp3	0.003	0.033	0.045	0.000	-0.153	0.674	0.557	0.918
P54823	Ddx6	0.098	0.242	0.066	0.000	-0.011	-0.081	-0.692	-0.327
P13864	Dnmt1	0.113	0.267	0.336	0.000	-0.055	-0.098	-0.924	-0.353
O08784	Tcof1	0.113	0.266	-0.009	0.000	0.150	-0.099	-0.536	-0.133
Q9D1G1	Rab1b	0.303	0.533	0.158	-0.095	0.000	-0.172	-0.134	0.029
Q99JY9	Actr3	0.188	0.378	0.048	-0.092	0.000	0.345	0.013	0.107
P60710	Actb	0.156	0.330	-0.073	0.121	0.000	-0.014	-0.514	-0.237
P63260	Actg1	0.156	0.330	-0.073	0.121	0.000	-0.014	-0.514	-0.237
Q80V86	Ints8	0.570	0.839	-0.504	3.710	0.000	0.053	1.310	-0.890
Q61655	Ddx19a	0.390	0.639	0.118	-0.022	0.000	0.132	-0.526	-0.084
P62830	Rpl23	0.643	0.919	0.106	-0.204	0.000	0.399	-0.679	-0.312
Q9CQ92	Fis1	0.078	0.209	0.240	-0.067	0.000	-0.226	-0.500	-0.127

P54276	Msh6	0.033	0.120	0.135	0.000	-0.179	-0.428	-1.340	-0.957
P06240	Lck	0.294	0.521	-0.202	0.459	0.000	-0.260	-0.257	-0.001
Q07235	Serpine2	0.277	0.498	0.428	0.000	-0.162	0.144	-0.483	-0.384
P30681	Hmgb2	0.095	0.238	-0.015	0.000	0.040	-0.144	-0.615	-0.195
Q60953	Pml	0.062	0.178	0.071	0.000	-0.108	-0.221	-1.170	-0.818
Q9ESX5	Dkc1	0.025	0.102	0.012	0.000	-0.193	-0.277	-0.447	-0.330
Q62189	Snrpa	0.029	0.111	0.000	-0.283	0.089	-0.543	-0.927	-0.493
Q8VDD5	Myh9	0.002	0.027	0.000	0.052	-0.141	0.796	0.520	0.751
Q922U1	Prpf3	0.013	0.071	-0.006	0.000	0.443	-0.588	-0.960	-0.545
Q78PY7	Snd1	0.431	0.687	0.121	0.000	-0.224	0.151	-0.543	-0.309
Q80UG5	Sept 9	0.237	0.444	-0.272	0.086	0.000	-0.200	-0.429	-0.138
P24452	Capg	0.026	0.104	-0.297	0.189	0.000	-0.617	-0.622	-0.450
P70372	Elavl1	0.445	0.702	-0.426	0.124	0.000	-0.293	-0.336	-0.126
Q9D0W5	Ppil1	0.009	0.056	-0.094	0.000	0.110	-0.708	-1.100	-0.574
P17182	Eno1	0.002	0.028	0.055	-0.141	0.000	-0.948	-1.530	-1.280
Q61599	Arhgdib	0.001	0.015	0.372	-0.115	0.000	-1.450	-1.770	-1.570
P29758	Oat	0.001	0.018	0.000	0.170	-0.473	1.810	1.920	2.200
Q01320	Top2a	0.345	0.585	0.420	0.000	-0.276	0.181	-0.632	-0.423
Q9CYA0	Creld2	0.202	0.398	0.461	0.000	-0.153	0.057	-0.768	-0.358
Q99KP6	Prpf19	0.018	0.083	0.000	-0.070	0.105	-0.384	-0.924	-0.583
Q9WV60	Gsk3b	0.103	0.249	0.000	-0.045	0.044	-0.511	-1.120	-0.157
Q80VD1	Fam98b	0.136	0.301	0.000	-0.052	0.036	-0.205	-0.328	-0.014
Q9Z2G6	Sel1l	0.015	0.077	0.000	0.785	-0.235	1.410	1.440	1.460
Q8QZY1	Eif3l	0.322	0.557	0.286	0.000	-0.080	0.235	-0.651	-0.334
Q9DBC7	Prkar1a	0.005	0.042	-0.209	0.003	0.000	-0.670	-0.996	-0.671
Q9QZE5	Copg1	0.155	0.329	-0.425	0.159	0.000	-0.405	-0.615	-0.289
O08749	Dld	0.005	0.039	0.000	0.188	-0.179	0.904	0.625	0.763
Q9QXS1	Plec	0.017	0.081	-0.164	0.000	0.083	0.797	0.326	0.702
P38647	Hspa9	0.072	0.196	0.035	0.000	-0.356	1.510	0.407	0.498
P63038	Hspd1	0.006	0.044	0.000	0.119	-0.240	1.090	0.650	0.822
Q99KJ8	Dctn2	0.536	0.803	-0.313	0.028	0.000	-0.031	-0.389	-0.172
Q9DBL1	Acadsb	0.001	0.022	0.000	0.020	-0.201	1.980	1.390	1.380
Q61335	Bcap31	0.080	0.212	0.000	-0.052	0.226	1.140	0.266	0.636
Q9Z2I0	Letm1	0.002	0.025	0.000	0.057	-0.198	0.565	0.495	0.591
Q62318	Trim28	0.170	0.352	-0.106	0.039	0.000	-0.141	-0.502	-0.101
P84104	Srsf3	0.070	0.193	0.568	-0.014	0.000	-0.104	-0.988	-0.770
P62196	Psmc5	0.040	0.136	0.000	0.308	-0.008	-0.201	-0.818	-0.531
Q9D8E6	Rpl4	0.054	0.163	0.321	-0.021	0.000	0.782	0.316	0.637
Q9CXR1	Dhrs7	0.113	0.266	0.279	0.000	-0.073	0.747	0.268	0.322
Q9CWZ3	Rbm8a	0.096	0.238	0.000	0.642	-0.100	-0.984	-1.000	-0.037
Q07813	Bax	0.323	0.559	-0.110	0.000	0.085	0.671	-0.114	0.210
P68368	Tuba4a	0.020	0.087	0.146	0.000	-0.226	-0.480	-1.030	-1.100
Q99JY0	Hadhb	0.462	0.721	-0.070	0.350	0.000	0.625	-0.024	0.237
Q8BX90	Fndc3a	0.002	0.025	0.055	0.000	-0.428	1.370	1.110	1.570
Q3UPF5	Zc3hav1	0.070	0.193	-0.342	0.000	0.012	-0.376	-0.579	-0.367
Q91X20	Ash2l	0.063	0.180	-0.240	0.077	0.000	-0.381	-0.538	-0.236
Q8BTM8	Flna	0.293	0.520	0.000	0.121	-0.418	0.299	0.000	0.080
P60122	Ruvbl1	0.055	0.165	0.000	-0.042	0.052	-0.122	-0.461	-0.238
Q61823	Pdcd4	0.011	0.065	-0.098	0.000	0.177	-1.090	-2.030	-1.100
P60335	Pcbp1	0.101	0.246	0.188	-0.140	0.000	-0.087	-0.939	-0.627
O88569	Hnrnpa2b1	0.148	0.318	-0.304	0.073	0.000	-0.472	-0.454	-0.145
Q9DBG6	Rpn2	0.000	0.012	0.000	0.145	-0.216	1.580	1.360	1.520
P17426	Ap2a1	0.687	0.963	0.021	0.000	-0.170	0.374	-0.291	0.030
Q9D903	Ebna1bp2	0.166	0.347	-0.081	0.000	0.201	-0.212	-0.623	-0.032
Q9Z110	Aldh18a1	0.109	0.261	0.000	0.052	-0.331	1.350	0.197	0.449
Q8VDK1	Nit1	0.269	0.488	0.029	0.000	-0.100	-0.074	-0.053	-0.391
Q9CPY7	Lap3	0.806	1.000	0.158	0.000	-0.383	0.568	-0.516	0.000
P14211	Calr	0.017	0.080	0.000	0.269	-0.256	0.717	0.570	0.599
Q91VI7	Rnh1	0.157	0.333	0.147	0.000	-0.013	0.060	-0.933	-0.519

Q9JHJ0	Tmod3	0.961	1.000	0.000	0.019	-0.389	0.016	-0.217	-0.146
P10107	Anxa1	0.215	0.412	1.740	0.000	-0.057	2.140	1.090	1.410
O70145	Ncf2	0.290	0.517	1.390	0.000	-0.200	0.382	-0.806	-0.670
P70429	Evl	0.271	0.490	0.032	-0.049	0.000	-0.053	-1.080	-0.145
Q3U9G9	Lbr	0.634	0.910	-0.053	0.000	0.182	0.005	-0.311	0.185
P50516	Atp6v1a	0.725	1.000	0.551	0.000	-0.094	0.623	-0.032	0.181
P50247	Ahcy	0.001	0.020	-0.059	0.000	0.042	-1.130	-1.720	-1.480
P40142	Tkt	0.039	0.132	0.533	-0.106	0.000	-0.340	-1.110	-0.864
Q8BH59	Slc25a12	0.037	0.127	-0.084	0.153	0.000	0.368	0.204	0.480
Q9R0X4	Acot9	0.058	0.171	0.000	0.694	-0.021	1.030	0.770	0.829
O08914	Faah	0.027	0.106	0.000	0.057	-0.048	-0.181	-0.299	-0.124
Q61550	Rad21	0.309	0.542	0.000	-0.027	0.146	-0.235	-0.456	0.157
P52875	Tmem165	0.029	0.110	0.372	0.000	-0.179	1.140	0.576	1.390
Q3TIX9	Usp39	0.018	0.083	0.270	-0.160	0.000	-0.496	-0.734	-0.449
P55302	Lrpap1	0.001	0.015	0.000	0.001	-0.525	1.780	1.710	2.060
Q3U0V2	Tradd	0.076	0.204	0.000	0.234	-0.079	-0.090	-0.626	-0.805
P21958	Tap1	0.423	0.677	-0.148	0.237	0.000	-0.065	-0.190	0.007
Q3TXS7	Psmd1	0.130	0.293	0.328	-0.138	0.000	-0.098	-0.383	-0.247
Q99PV0	Prpf8	0.020	0.087	-0.023	0.225	0.000	-0.196	-0.284	-0.261
Q9Z0P5	Twf2	0.004	0.034	0.062	-0.102	0.000	-1.230	-1.830	-1.120
Q62261	Sptbn1	0.014	0.072	0.000	0.243	-0.084	0.494	0.512	0.429
F6ZDS4	Tpr	0.023	0.096	-0.076	0.000	0.080	-0.279	-0.547	-0.266
P11835	Itgb2	0.068	0.188	0.255	-0.053	0.000	-0.095	-0.485	-0.320
Q9CR51	Atp6v1g1	0.816	1.000	0.620	0.000	-0.365	0.327	-0.391	0.055
P32921	Wars	0.102	0.248	0.000	-0.106	0.007	-0.096	-0.377	-0.196
Q9JIK5	Ddx21	0.023	0.096	0.038	0.000	-0.075	-0.306	-0.743	-0.429
P43404	Zap70	0.038	0.131	-0.465	0.041	0.000	-0.669	-0.946	-0.589
Q5FWK3	Arhgap1	0.296	0.524	0.076	-0.093	0.000	0.053	-0.363	-0.173
Q8BH61	F13a1	0.387	0.635	0.693	0.000	-0.022	0.346	-0.483	-0.175
Q8BWT1	Acaa2	0.089	0.228	0.000	0.088	-0.212	0.592	0.136	0.245
P19973	Lsp1	0.002	0.026	0.000	-0.001	0.158	-1.090	-1.130	-0.706
P48678	Lmna	0.002	0.028	0.000	0.046	-0.348	0.967	0.745	0.972
G5E870	Trip12	0.026	0.105	-0.220	0.190	0.000	-0.368	-0.610	-0.474
Q9JHS9	Cwc15	0.035	0.124	0.000	-0.060	0.174	-0.357	-0.766	-1.240
O54824	Il16	0.400	0.650	-0.327	0.000	0.082	-0.272	-0.328	-0.065
Q91YI4	Arrb2	0.043	0.141	0.421	-0.090	0.000	-0.306	-1.050	-0.648
Q99JY3	Gimap4	0.000	0.014	-0.308	0.000	0.029	-1.380	-1.590	-1.690
P08003	Pdia4	0.111	0.264	0.208	0.000	-0.016	0.512	0.143	0.322
O35295	Purb	0.019	0.085	-0.254	0.142	0.000	-0.477	-0.948	-0.748
P62821	Rab1A	0.807	1.000	0.232	-0.032	0.000	0.737	-0.085	-0.211
Q08943	Ssrp1	0.047	0.149	0.000	0.059	-0.129	-0.264	-0.982	-0.659
Q9CW46	Raver1	0.212	0.409	-0.429	0.000	0.009	-0.636	-0.311	-0.288
Q8BL97	Srsf7	0.044	0.142	0.415	0.000	-0.051	-0.237	-0.986	-0.723
Q8C7E9	Cstf2t	0.231	0.435	0.000	-0.128	0.732	-4.370	-0.082	-0.715
P49722	Psma2	0.000	0.014	-0.245	0.000	0.011	-1.230	-1.480	-1.240
Q9D8W5	Psmd12	0.045	0.146	0.000	0.153	-0.090	-0.282	-1.060	-0.637
Q9WUB3	Pygm	0.033	0.121	-0.282	0.000	0.033	-1.330	-0.756	-0.562
Q99N84	Mrps18b	0.001	0.019	-0.100	0.262	0.000	1.290	1.020	1.220
Q99LX0	Park7	0.001	0.015	0.016	-0.257	0.000	-1.140	-1.450	-1.350
Q3UHJ0	Aak1	0.314	0.547	-0.257	0.000	0.048	-0.279	-0.575	-0.009
Q9DAU1	Cnpy3	0.036	0.125	0.021	0.000	-0.450	0.436	0.377	0.266
Q60710	Samhd1	0.000	0.013	-0.120	0.000	0.000	-0.783	-1.010	-0.907
Q91ZW3	Smarca5	0.092	0.233	0.000	0.038	-0.092	-0.173	-0.514	-0.168
Q9CQF9	Pcyox1	0.003	0.031	0.261	0.000	-0.610	1.640	1.550	1.550
Q3UN02	Lclat1	0.023	0.096	-0.171	0.817	0.000	1.370	1.200	1.660
Q80YR5	Safb2	0.033	0.119	-0.279	0.036	0.000	-0.582	-0.899	-0.420
P26039	Tln1	0.041	0.137	0.059	0.000	-0.282	-0.358	-1.020	-1.000
Q76MZ3	Ppp2r1a	0.013	0.069	0.000	0.029	-0.018	-0.282	-0.657	-0.641
P97371	Psme1	0.001	0.022	-0.041	0.476	0.000	-1.210	-1.170	-1.120

Q9QUJ7	Acsl4	0.326	0.563	0.186	0.000	-0.010	0.509	-0.057	0.321
Q9CQ06	Mrpl24	0.031	0.115	0.158	0.000	-0.175	0.302	0.820	0.865
Q80X90	Flnb	0.001	0.014	0.069	0.000	-0.289	2.350	1.740	2.050
Q9R0E1	Plod3	0.000	0.009	0.000	0.480	-0.111	3.740	3.370	3.760
Q9WU78	Pdcd6ip	0.102	0.247	-0.082	0.153	0.000	-0.146	-0.697	-0.246
Q8BIQ5	Cstf2	0.421	0.675	-0.437	0.000	0.414	-1.200	-0.354	0.232
P67984	Rpl22	0.254	0.467	0.247	0.000	-0.018	0.449	0.053	0.300
P47740	Aldh3a2	0.057	0.169	0.000	0.097	-0.121	0.310	0.102	0.328
Q9CPV4	Glod4	0.023	0.096	0.000	0.150	-0.049	-0.326	-0.824	-0.470
P26040	Ezr	0.241	0.451	0.000	0.009	-0.085	0.234	-0.093	0.286
P42227	Stat3	0.103	0.250	0.000	-0.155	0.131	-0.089	-0.977	-0.688
Q9Z1N5	Ddx39b	0.760	1.000	-0.016	0.782	0.000	0.109	0.138	0.256
P01831	Thy1	0.024	0.098	0.000	-0.028	0.249	0.606	0.378	0.771
Q8K0C4	Cyp51a1	0.000	0.008	0.000	0.282	-0.189	3.810	3.390	3.830
Q9D0L7	Armc10	0.027	0.107	-0.131	0.052	0.000	-0.301	-0.431	-0.208
Q8BMP6	Acbd3	0.380	0.629	0.334	-0.249	0.000	0.447	-1.140	-0.709
O35892	Sp100	0.119	0.276	0.000	-0.023	0.093	-0.048	-0.538	-0.220
P36371	Tap2	0.787	1.000	-0.225	0.273	0.000	-0.153	-0.078	0.133
Q9CZ44	Nsfl1c	0.000	0.011	-0.089	0.000	0.161	-1.340	-1.640	-1.510
Q9CR61	Ndufb7	0.020	0.088	0.000	-0.043	0.113	0.717	0.435	1.080
P23492	Pnp	0.010	0.060	0.405	0.000	-0.271	-0.763	-1.050	-1.040
O88685	Psmc3	0.008	0.054	0.000	0.126	-0.124	-0.422	-0.569	-0.370
P62960	Ybx1	0.498	0.764	0.017	0.000	-0.011	0.279	-0.611	-0.238
Q8C0I1	Agps	0.043	0.142	0.639	0.000	-0.154	1.310	0.764	0.956
Q8R326	Pspc1	0.029	0.110	0.000	0.345	-0.071	-0.607	-0.409	-0.294
Q9DC69	Ndufa9	0.004	0.037	-0.072	0.102	0.000	0.741	0.442	0.635
Q80YV3	Trrap	0.070	0.193	-0.087	0.000	0.024	-0.210	-0.361	-0.101
P47941	Crkl	0.001	0.017	0.039	-0.088	0.000	-1.070	-1.520	-1.270
P11276	Fn1	0.000	0.013	0.759	0.000	-0.029	4.210	3.600	3.790
Q9D1M7	Fkbp11	0.001	0.014	0.000	0.157	-0.617	2.940	2.470	2.970
P56399	Usp5	0.041	0.136	-0.064	0.055	0.000	-0.344	-1.040	-1.390
Q9CQN1	Trap1	0.459	0.719	0.170	0.000	-0.139	0.175	-0.335	-0.254
O89023	Tpp1	0.463	0.722	0.691	0.000	-0.365	0.480	-0.634	-0.724
Q8BHD7	Ptbp3	0.069	0.192	0.000	0.316	-0.167	-0.178	-0.748	-0.545
P11881	Itpr1	0.010	0.059	0.000	0.236	-0.244	0.838	0.632	0.646
Q99KI3	Emc3	0.001	0.015	0.000	0.228	-0.156	1.460	1.280	1.590
Q9WTI7	Myo1c	0.000	0.012	0.000	0.063	-0.194	2.750	2.170	2.720
Q61210	Arhgef1	0.005	0.041	0.000	0.001	-0.005	-0.416	-0.797	-0.644
P48036	Anxa5	0.005	0.042	0.000	0.177	-0.377	0.835	0.814	0.988
B2RY56	Rbm25	0.013	0.071	0.000	-0.016	0.053	-0.439	-1.080	-0.945
P15702	Spn	0.027	0.105	-0.280	0.000	0.175	-0.428	-0.534	-0.621
P12815	Pdcd6	0.518	0.782	-0.178	0.236	0.000	-0.267	0.110	-0.131
Q9D6Z1	Nop56	0.058	0.171	-0.091	0.006	0.000	-0.160	-0.587	-0.343
Q9EPB4	Pycard	0.002	0.023	0.029	0.000	-0.084	-1.520	-1.930	-1.240
D0QMC3	Mndal	0.015	0.076	-0.179	0.041	0.000	-0.432	-0.756	-0.459
P80313	Cct7	0.026	0.104	0.099	0.000	-0.035	-0.245	-0.699	-0.421
Q9Z2Z6	Slc25a20	0.006	0.043	0.161	0.000	-0.081	0.953	0.633	0.619
Q8VHE0	Sec63	0.001	0.014	0.000	0.208	-0.401	2.140	1.810	1.980
Q64674	Srm	0.120	0.277	0.297	0.000	-0.271	-0.147	-0.522	-0.530
P99027	Rplp2	0.515	0.779	0.066	-0.014	0.000	-0.010	-0.366	0.116
Q921H8	Acaa1a	0.168	0.349	0.077	0.000	-0.377	0.732	-0.098	0.503
P21550	Eno3	0.001	0.022	0.000	0.285	-0.011	-0.823	-1.150	-1.090
Q08024	Cbfb	0.018	0.083	-0.165	0.091	0.000	-0.345	-0.749	-0.625
Q6P2L6	Whsc1l1	0.352	0.593	-0.300	0.000	0.252	-0.124	-1.230	-0.019
Q62523	Zyx	0.182	0.369	-0.086	0.110	0.000	0.768	-0.019	0.410
Q9DB05	Napa	0.787	1.000	0.217	0.000	-0.130	0.223	-0.284	-0.005
Q99K48	Nono	0.023	0.096	-0.200	0.000	0.002	-0.434	-0.635	-0.333
O09044	Snap23	0.002	0.023	0.028	0.000	-0.068	0.650	0.420	0.604
Q61233	Lcp1	0.000	0.008	0.000	0.104	-0.046	-1.290	-1.480	-1.380

P24527	Lta4h	0.001	0.019	0.296	0.000	-0.168	-1.520	-2.110	-1.850
P52480	Pkm	0.003	0.031	0.033	0.000	-0.045	-0.658	-1.130	-1.020
Q60520	Sin3a	0.049	0.154	-0.146	0.000	0.086	-0.240	-0.604	-0.309
Q68FL6	Mars	0.598	0.871	0.310	0.000	-0.218	0.343	-0.524	-0.239
Q8BV49	Pyhin1	0.026	0.105	0.000	-0.130	0.140	-0.427	-0.852	-0.407
Q01730	Rsu1	0.373	0.622	0.000	0.204	-0.850	0.353	0.275	-0.182
O08553	Dpysl2	0.008	0.055	-0.381	0.023	0.000	-0.799	-1.040	-0.780
Q3U7R1	Esyt1	0.377	0.625	-0.054	0.266	0.000	0.169	0.087	0.307
Q9JJX6	P2rx4	0.610	0.885	0.645	-0.068	0.000	0.517	-0.345	-0.170
Q91W90	Txndc5	0.003	0.033	0.000	0.085	-0.303	0.869	0.642	0.871
P28740	Kif2a	0.128	0.291	0.126	0.000	-0.368	-0.159	-0.728	-0.764
Q9WVA4	Tagln2	0.004	0.038	-0.036	0.121	0.000	-0.400	-0.593	-0.378
Q99P72	Rtn4	0.000	0.006	0.000	0.066	-0.069	1.910	1.670	1.840
P09405	Ncl	0.005	0.042	0.000	0.013	-0.184	-0.731	-1.300	-1.140
Q9ERS2	Ndufa13	0.056	0.168	0.038	-0.179	0.000	0.652	0.143	0.876
P29391	Ftl1	0.048	0.152	0.334	-0.304	0.000	-0.448	-1.460	-0.987
P70288	Hdac2	0.731	1.000	0.000	0.251	-0.259	-0.156	-0.163	0.118
Q8VE70	Pdcd10	0.005	0.039	0.000	-0.139	0.036	-0.455	-0.496	-0.690
P19783	Cox4i1	0.004	0.038	0.100	-0.024	0.000	0.711	0.422	0.696
Q9CZ42	Naxd	0.001	0.018	0.148	0.000	-0.050	1.030	0.765	0.960
P61967	Ap1s1	0.008	0.054	0.000	-0.021	0.223	-0.424	-0.864	-0.773
P42225	Stat1	0.005	0.039	-0.079	0.271	0.000	-0.603	-0.992	-0.866
Q9CVB6	Arpc2	0.043	0.142	0.217	-0.103	0.000	0.408	0.250	0.503
Q99LC3	Ndufa10	0.006	0.047	0.000	0.010	-0.249	0.718	0.460	0.860
Q6PFD9	Nup98	0.115	0.270	-0.095	0.000	0.190	-0.155	-0.472	-0.120
Q8R2Y8	Ptrh2	0.015	0.077	-0.265	0.190	0.000	1.240	1.150	2.290
P54728	Rad23b	0.023	0.096	-0.121	0.083	0.000	-0.526	-1.300	-0.766
Q9WTM5	Ruvbl2	0.218	0.417	0.000	0.106	-0.153	-0.146	-0.350	-0.056
Q9R1P4	Psma1	0.012	0.067	-0.187	0.086	0.000	-0.997	-2.060	-1.320
Q8R4N0	Clybl	0.061	0.177	0.182	0.000	-0.138	0.704	0.176	0.694
Q91VC3	Eif4a3	0.006	0.046	0.000	-0.016	0.085	-0.409	-0.644	-0.359
O88696	Clpp	0.016	0.078	0.000	0.043	-0.364	0.863	0.401	0.665
P70699	Gaa	0.001	0.022	0.000	-0.360	0.035	1.170	0.910	1.070
Q99NB9	Sf3b1	0.056	0.166	-0.164	0.007	0.000	-0.508	-0.725	-0.208
Q8BHL5	Elmo2	0.073	0.198	-0.116	0.032	0.000	-0.361	-0.697	-1.500
Q9D1Q6	Erp44	0.003	0.033	0.018	0.000	-0.372	0.878	0.686	0.710
D3Z6Q9	Bin2	0.005	0.039	-0.352	0.269	0.000	-1.050	-1.470	-1.300
Q80Y81	Elac2	0.624	0.899	0.000	-0.208	0.155	0.131	-0.831	0.116
P24668	M6pr	0.297	0.524	0.000	-1.460	0.258	0.120	0.295	0.320
Q8BH43	Wasf2	0.373	0.621	-0.073	0.000	0.024	0.173	-0.125	0.272
P61358	Rpl27	0.051	0.157	0.000	0.389	-0.168	0.470	0.530	0.698
Q8BP47	Nars	0.300	0.529	0.063	0.000	-0.627	0.134	-0.049	0.174
P42208	Sept 2	0.001	0.022	0.181	0.000	-0.114	0.895	0.778	1.010
Q9CRD0	Ociad1	0.184	0.372	-0.379	0.247	0.000	-0.636	-0.622	-0.097
Q6PDG5	Smarcc2	0.084	0.220	0.000	-0.027	0.113	-0.028	-0.406	-0.317
Q64105	Spr	0.003	0.033	-0.370	0.096	0.000	-1.650	-1.710	-1.160
P25206	Mcm3	0.066	0.185	0.280	0.000	-0.196	-0.205	-1.110	-0.943
P08752	Gnai2	0.756	1.000	0.074	0.000	-0.107	0.169	-0.194	-0.132
O35382	Exoc4	0.173	0.357	0.171	0.000	-0.131	0.080	-0.688	-0.729
O08788	Dctn1	0.369	0.616	0.018	-0.153	0.000	0.258	-0.787	-0.596
P24161	Cd247	0.811	1.000	-0.617	0.000	0.247	-0.627	0.147	0.420
Q9CW03	Smc3	0.037	0.128	0.000	-0.055	0.001	-0.243	-0.484	-0.187
Q8CIE6	Copa	0.118	0.274	0.168	0.000	-0.012	-0.029	-0.703	-0.326
Q9ESP1	Sdf2l1	0.816	1.000	0.000	0.023	-0.647	-0.293	-0.576	0.035
P09528	Fth1	0.085	0.220	0.442	-0.233	0.000	-0.225	-1.190	-0.715
Q9DB77	Uqcrc2	0.140	0.306	0.000	0.125	-0.097	0.556	0.046	0.312
O08810	Eftud2	0.022	0.095	0.000	0.151	-0.093	-0.237	-0.287	-0.220
Q8C4Q6	Aida	0.026	0.105	-0.212	0.550	0.000	1.430	1.020	0.842
Q05186	Rcn1	0.000	0.003	-0.133	0.156	0.000	3.830	3.540	3.760

Q8K183	Pdxk	0.017	0.081	0.000	-0.383	0.018	-0.842	-1.590	-1.010
P17710	Hk1	0.085	0.221	-0.211	0.114	0.000	-0.278	-0.528	-0.214
Q61425	Hadh	0.375	0.623	-0.733	0.214	0.000	0.578	0.284	-0.255
Q8C570	Rae1	0.961	1.000	-0.124	0.114	0.000	-0.099	0.017	0.085
O54941	Smarce1	0.092	0.232	-0.025	0.000	0.203	-0.414	-0.671	-0.041
Q9Z1Q2	Abhd16a	0.066	0.186	0.213	-0.145	0.000	0.518	0.389	1.190
P10518	Alad	0.007	0.050	0.086	-0.152	0.000	-1.240	-2.500	-1.940
Q9Z1Z2	Strap	0.011	0.064	0.000	-0.050	0.159	-0.265	-0.534	-0.473
Q99MN9	Pccb	0.003	0.032	0.000	0.238	-0.023	0.894	0.712	1.040
Q9DBG3	Ap2b1	0.155	0.329	0.000	0.158	-0.099	0.695	0.126	0.236
Q8VDN2	Atp1a1	0.012	0.069	-0.147	0.017	0.000	0.628	0.290	0.682
P14901	Hmox1	0.394	0.643	0.207	0.000	-0.907	-0.150	-0.921	-0.834
O35083	Agpat1	0.035	0.123	0.411	0.000	-0.054	1.020	0.828	0.500
Q3U1F9	Pag1	0.317	0.550	0.105	-0.106	0.000	-0.473	-0.720	0.213
Q8BZN6	Dock10	0.210	0.406	-0.532	0.000	0.228	-0.481	-1.600	-0.303
P24063	Itgal	0.054	0.163	-0.246	0.010	0.000	-0.641	-0.514	-0.247
Q8BMS1	Hadha	0.932	1.000	0.027	0.000	-0.269	0.492	-0.475	-0.178
Q9R1T4	Sept 6	0.016	0.078	-0.316	0.001	0.000	-0.583	-0.779	-0.525
P38060	Hmgcl	0.061	0.177	0.000	0.148	-0.178	0.592	0.202	0.328
Q61191	Hcfc1	0.920	1.000	-0.021	0.000	0.544	0.085	-0.064	0.590
P06151	Ldha	0.001	0.018	-0.165	0.115	0.000	-0.980	-1.190	-0.934
Q9CZ13	Uqcrc1	0.015	0.076	0.081	0.000	-0.051	0.660	0.292	0.464
Q3TDQ1	Stt3b	0.003	0.031	0.054	0.000	-0.451	1.190	0.890	1.070
Q922W5	Pycr1	0.000	0.014	0.208	0.000	-0.219	2.510	1.920	2.420
Q91W39	Ncoa5	0.060	0.175	0.026	0.000	-0.041	-0.220	-0.959	-0.518
P63168	Dynll1	0.922	1.000	0.502	-0.203	0.000	0.013	-0.054	0.267
Q9EQH3	Vps35	0.223	0.424	0.352	-0.082	0.000	0.112	-0.639	-0.304
P97287	Mcl1	0.225	0.427	0.197	0.000	-0.777	0.487	-0.153	1.300
P50544	Acadvl	0.245	0.456	0.000	0.061	-0.285	0.866	-0.022	0.132
Q9DC51	Gnai3	0.950	1.000	0.028	-0.131	0.000	0.178	-0.388	0.145
Q8BK64	Ahsa1	0.072	0.197	0.000	0.218	-0.021	-0.131	-1.130	-0.729
Q9WTX5	Skp1	0.009	0.057	0.000	0.142	-0.147	-0.508	-0.496	-0.360
P42209	Sept 1	0.040	0.135	-0.383	0.163	0.000	-0.531	-0.641	-0.536
P32233	Drg1	0.735	1.000	0.167	-0.118	0.000	0.094	-0.600	0.256
P26043	Rdx	0.042	0.138	0.000	0.671	-0.017	0.763	1.170	1.520
P63158	Hmgb1	0.474	0.734	0.000	-0.236	0.219	0.196	-0.585	-0.247
Q9R1C7	Prpf40a	0.057	0.169	0.000	0.036	-0.175	-0.196	-0.679	-0.769
P62192	Psmc1	0.016	0.078	0.000	0.007	-0.059	-0.317	-0.671	-0.390
Q61656	Ddx5	0.021	0.090	0.145	0.000	-0.259	-0.449	-0.909	-0.754
Q8CAQ8	Immt	0.003	0.032	0.000	0.109	-0.145	0.915	0.583	0.795
P56480	Atp5b	0.009	0.057	0.000	0.258	-0.162	0.723	0.568	0.808
P60867	Rps20	0.015	0.077	0.165	0.000	-0.084	-0.470	-0.687	-0.324
Q9QXZ0	Macf1	0.523	0.789	0.215	-0.056	0.000	0.505	-0.100	0.160
P97351	Rps3a	0.907	1.000	0.091	-0.290	0.000	0.303	-0.279	-0.309
Q8CEC0	Nup88	0.199	0.394	-0.116	0.129	0.000	-0.168	-0.370	-0.019
Q8R5J9	Arl6ip5	0.031	0.115	-0.357	0.100	0.000	0.322	0.545	0.805
Q64514	Tpp2	0.416	0.669	0.008	0.000	-1.190	-0.674	-0.816	-0.777
Q6GQT9	Nomo1	0.001	0.018	0.066	0.000	-0.516	2.070	1.620	1.860
Q9CYG7	Tomm34	0.013	0.071	-0.013	0.210	0.000	-0.338	-0.800	-0.588
Q9Z329	Itpr2	0.713	0.989	-0.251	0.000	0.041	-0.190	-0.184	0.027
Q8CCJ3	Ufl1	0.399	0.650	0.000	-0.401	0.074	-0.084	-0.609	-0.240
P46460	Nsf	0.134	0.298	0.377	0.000	-0.134	0.037	-0.914	-0.723
P61164	Actr1a	0.105	0.252	0.017	-0.031	0.000	-0.230	-0.245	-0.015
O35857	Timm44	0.016	0.078	0.231	0.000	-0.162	0.827	0.450	0.765
Q3THE2	Myl12b	0.009	0.056	-0.141	0.510	0.000	0.987	1.260	1.210
Q60972	Rbbp4	0.188	0.378	-0.072	0.000	0.231	-0.232	-0.800	-0.009
P09411	Pgk1	0.021	0.091	0.132	-0.121	0.000	-0.542	-1.100	-0.544
Q9WUQ2	Preb	0.007	0.048	-0.081	0.122	0.000	0.725	0.406	0.640
Q8CC88	Vwa8	0.942	1.000	0.000	0.045	-0.313	0.847	-0.752	-0.481

Q9D7N9	Apmap	0.049	0.154	0.018	0.000	-0.061	0.406	0.078	0.386
O54782	Man2b2	0.003	0.033	0.000	0.230	-0.073	-1.030	-1.770	-1.620
P80316	Cct5	0.042	0.140	0.066	-0.104	0.000	-0.220	-0.838	-0.649
Q8BMJ2	Lars	0.881	1.000	0.252	0.000	-0.100	0.278	-0.242	0.028
Q9DBZ1	Ikbip	0.000	0.002	0.000	-0.098	0.101	6.270	5.940	6.260
P43406	Itgav	0.000	0.013	0.172	-0.250	0.000	2.300	1.820	2.020
P10711	Tcea1	0.098	0.242	0.028	-0.179	0.000	-0.348	-0.662	-0.165
Q9D2V8	Mfsd10	0.007	0.051	0.000	0.022	-0.232	0.826	0.424	0.713
Q91VD9	Ndufs1	0.002	0.025	0.000	0.197	-0.065	0.847	0.648	0.786
Q9D2V7	Coro7	0.000	0.008	0.000	-0.014	0.005	-1.400	-1.690	-1.670
Q8BFR5	Tufm	0.440	0.697	0.165	0.000	-0.339	0.256	-0.698	-0.598
P47911	Rpl6	0.036	0.126	0.095	0.000	-0.087	0.610	0.185	0.488
Q14C51	Ptcd3	0.001	0.019	0.000	0.011	-0.187	0.717	0.554	0.741
P62814	Atp6v1b2	0.820	1.000	0.689	0.000	-0.027	0.606	-0.188	-0.001
Q9WV55	Vapa	0.035	0.124	0.000	0.399	-0.125	0.518	0.621	0.719
P49718	Mcm5	0.432	0.688	0.140	0.000	-0.032	0.135	-0.802	-0.001
P97760	Polr2c	0.231	0.436	-0.024	0.099	0.000	-0.330	-1.380	0.008
P10605	Ctsb	0.113	0.266	0.436	0.000	-0.385	-0.190	-1.040	-0.842
Q9CU62	Smc1a	0.086	0.222	0.000	-0.003	0.011	-0.155	-0.506	-0.143
P97855	G3bp1	0.321	0.557	0.068	-0.159	0.000	-0.136	-1.010	-0.025
Q6PDQ2	Chd4	0.367	0.615	0.000	-0.102	0.117	-0.201	-0.551	0.134
Q3TBD2	Hmha1	0.000	0.014	0.000	-0.131	0.051	-1.160	-1.530	-1.440
P26450	Pik3r1	0.468	0.727	0.000	0.958	-0.100	-0.021	0.030	0.038
Q62087	Pon3	0.014	0.073	-0.127	0.000	0.034	0.275	0.248	0.476
Q7TMK9	Syncrip	0.677	0.953	0.000	0.301	-0.343	0.126	-0.176	0.326
Q9CZX9	Emc4	0.015	0.076	-0.448	0.367	0.000	0.940	1.180	0.890
Q91VK1	Bzw2	0.115	0.269	0.350	0.000	-0.019	-0.027	-0.652	-0.298
Q3TKT4	Smarca4	0.343	0.583	0.057	-0.076	0.000	0.065	-0.478	-0.132
Q9DB85	Rrp8	0.469	0.728	-0.371	0.000	0.165	-0.132	-0.454	-0.090
P17427	Ap2a2	0.383	0.632	0.415	0.000	-0.703	0.757	-0.016	0.153
Q99L47	St13	0.014	0.072	-0.081	0.036	0.000	-0.526	-1.130	-0.692
Q99P58	Rab27b	0.322	0.557	0.000	0.955	-1.050	-0.404	-0.436	-1.680
P50580	Pa2g4	0.244	0.454	0.328	-0.040	0.000	0.166	-0.734	-0.310
Q8R0G9	Nup133	0.563	0.832	0.000	-0.023	0.096	0.037	-0.449	0.137
Q9QX60	Dguok	0.708	0.984	-0.077	0.150	0.000	0.045	-0.050	0.196
Q9JJL8	Sars2	0.446	0.703	0.281	0.000	-0.050	0.559	-0.022	0.196
Q8CGC7	Eprs	0.795	1.000	0.120	0.000	-0.150	0.337	-0.462	-0.109
P57780	Actn4	0.021	0.091	-0.172	0.037	0.000	-0.274	-0.547	-0.455
P14152	Mdh1	0.000	0.009	-0.168	0.000	0.080	-1.560	-1.840	-1.800
Q6P4T2	Snrnp200	0.013	0.069	-0.020	0.137	0.000	-0.250	-0.524	-0.332
Q99JR1	Sfxn1	0.018	0.083	0.000	0.180	-0.179	0.403	0.408	0.393
Q9DCD0	Pgd	0.071	0.195	0.917	0.000	-0.095	-0.280	-1.050	-0.718
Q9Z0H3	Smarcb1	0.030	0.113	0.000	-0.066	0.179	-0.294	-0.516	-0.219
O89086	Rbm3	0.009	0.056	-0.209	0.057	0.000	-0.430	-0.622	-0.681
Q922V4	Plrg1	0.064	0.181	0.000	0.843	-0.233	-0.721	-0.595	-0.591
Q6A026	Pds5a	0.449	0.707	-0.685	0.000	0.044	-0.448	-0.612	-0.234
Q80Y14	Glrx5	0.112	0.265	0.079	0.000	-0.218	0.910	0.185	0.266
P50136	Bckdha	0.099	0.243	0.031	0.000	-0.341	0.421	0.014	0.389
Q31125	Slc39a7	0.027	0.105	0.000	-0.045	0.406	0.591	0.957	1.230
P54071	Idh2	0.004	0.036	0.054	0.000	-0.276	1.440	0.889	0.986
F7BJB9	Morc3	0.118	0.274	0.164	-0.166	0.000	-0.243	-0.973	-0.297
Q61584	Fxr1	0.129	0.291	-0.026	0.120	0.000	-0.203	-0.299	0.011
Q921T2	Tor1aip1	0.690	0.965	-0.053	0.000	0.307	0.445	-0.208	0.311
Q7TT37	Ikbkap	0.264	0.481	0.088	0.000	-0.089	0.136	-0.527	-0.447
Q9EQ61	Pes1	0.132	0.295	0.201	0.000	-0.198	-0.092	-0.992	-0.526
Q8C1A5	Thop1	0.006	0.046	0.158	-0.220	0.000	-0.983	-1.770	-1.750
O55222	Ilk	0.020	0.087	0.022	0.000	-0.417	0.976	0.527	0.493
Q91VV4	Dennd2d	0.016	0.079	-0.287	0.000	0.220	-0.634	-0.864	-0.591
Q80XI4	Pip4k2b	0.801	1.000	0.042	-0.073	0.000	0.261	-0.324	-0.109

Q91V41	Rab14	0.967	1.000	0.000	0.178	-0.083	0.345	-0.065	-0.209
P56960	Exosc10	0.282	0.506	0.835	0.000	-0.252	0.633	0.441	0.788
Q8BXA1	Golim4	0.007	0.050	0.892	0.000	-0.477	2.510	2.100	2.200
Q9WTP7	Ak3	0.037	0.129	-0.146	0.101	0.000	0.809	0.233	0.598
Q3U0V1	Khsrp	0.421	0.676	-0.157	0.000	0.015	-0.296	-0.296	0.081
O54692	Zw10	0.471	0.730	0.324	-0.222	0.000	0.295	0.022	0.210
Q9DBR7	Ppp1r12a	0.013	0.071	-0.181	0.000	0.044	-0.554	-1.010	-0.600
Q61881	Mcm7	0.037	0.127	0.253	0.000	-0.194	-0.352	-1.070	-0.804
Q9EP69	Sacm1l	0.008	0.051	-0.137	0.052	0.000	0.613	0.321	0.520
P63330	Ppp2ca	0.032	0.118	0.000	-0.177	0.241	-0.502	-0.750	-0.337
Q9CQE5	Rgs10	0.001	0.018	-0.183	0.000	0.053	-1.900	-1.880	-1.380
Q6NVE9	Pptc7	0.557	0.825	0.000	-0.054	0.016	0.205	-0.490	-0.140
Q7TMF3	Ndufa12	0.011	0.064	-0.161	0.000	0.141	0.792	0.547	1.110
O35874	Slc1a4	0.008	0.051	-2.820	0.000	0.039	3.660	3.800	3.980
P11032	Gzma	0.448	0.706	-1.520	0.000	0.204	-1.490	-1.050	-0.367
Q80VW7	Akna	0.096	0.238	-0.012	0.069	0.000	-0.965	-1.600	-0.137
Q921M7	Fam49b	0.000	0.009	0.127	0.000	-0.104	-1.080	-1.160	-1.120
O35465	Fkbp8	0.521	0.786	0.000	0.167	-0.029	0.317	-0.227	-0.452
P12970	Rpl7a	0.049	0.154	0.141	-0.105	0.000	0.570	0.175	0.428
O35643	Ap1b1	0.185	0.374	0.145	0.000	-0.102	-0.013	-0.683	-0.260
P61982	Ywhag	0.016	0.078	0.076	0.000	-0.065	-0.399	-0.977	-0.678
Q9CXY6	Ilf2	0.540	0.807	0.000	0.631	-0.035	-0.337	0.060	0.300
O70503	Hsd17b12	0.014	0.072	0.001	0.000	-0.328	1.430	0.851	0.653
Q62376	Snrnp70	0.125	0.285	-0.253	0.000	1.590	-0.713	-0.893	-0.482
P62702	Rps4x	0.826	1.000	0.036	0.000	-0.324	0.307	-0.757	-0.072
Q61029	Tmpo	0.756	1.000	-0.325	0.000	0.312	-0.177	-0.353	0.258
Q8K3G5	Vrk3	0.070	0.193	-0.071	0.000	0.328	-0.503	-0.539	-0.092
Q8R016	Blmh	0.021	0.091	-0.097	0.256	0.000	-1.320	-1.100	-0.472
A2AF47	Dock11	0.001	0.018	0.000	0.134	-0.011	-0.552	-0.771	-0.648
Q9EQQ9	Mgea5	0.066	0.186	-0.118	0.185	0.000	-0.295	-0.900	-0.343
Q8VCW4	Unc93b1	0.699	0.975	0.532	-0.017	0.000	0.616	0.022	0.192
Q60932	Vdac1	0.006	0.046	0.000	0.313	-0.052	1.240	1.010	1.700
P68040	Rack1	0.095	0.237	0.132	0.000	-0.220	-0.151	-0.721	-0.490
Q6ZQI3	Mlec	0.323	0.558	0.076	0.000	-0.122	1.290	-0.531	1.210
Q60597	Ogdh	0.007	0.048	0.000	0.045	-0.239	0.747	0.432	0.587
Q61316	Hspa4	0.002	0.027	0.000	-0.007	0.001	-0.700	-1.140	-0.857
P26369	U2af2	0.063	0.181	0.204	0.000	-0.149	-0.162	-0.840	-0.740
Q9ESZ8	Gtf2i	0.049	0.154	-0.230	0.072	0.000	-0.284	-0.490	-0.318
O08900	Ikzf3	0.585	0.857	-0.536	0.000	0.072	-0.517	-0.398	0.008
Q99P88	Nup155	0.894	1.000	-0.030	0.199	0.000	0.139	-0.001	-0.006
Q99K85	Psat1	0.006	0.044	0.000	0.027	-0.015	-0.509	-0.996	-0.778
Q69Z99	Znf512	0.404	0.655	-0.111	0.000	0.395	-0.071	-0.745	0.203
Q78IK4	Apool	0.010	0.060	-0.062	0.205	0.000	0.712	0.459	0.823
P35821	Ptpn1	0.031	0.116	-0.040	0.235	0.000	-0.162	-0.284	-0.302
Q91YT0	Ndufv1	0.001	0.019	0.000	0.034	-0.076	0.817	0.553	0.688
Q91ZA3	Pcca	0.004	0.037	0.000	0.273	-0.086	0.822	0.667	0.847
P46471	Psmc2	0.133	0.297	0.000	0.036	-0.095	-0.069	-0.984	-0.512
Q65Z40	Wapl	0.122	0.280	0.000	0.100	-0.214	-0.278	-0.555	-0.160
Q5SSI6	Utp18	0.118	0.274	0.035	0.000	-0.326	-0.227	-0.724	-0.437
Q61464	Znf638	0.087	0.224	-0.106	0.232	0.000	-0.212	-0.535	-0.164
Q8VDP4	Ccar2	0.001	0.019	-0.213	0.000	0.026	-1.020	-1.310	-1.030
P54923	Adprh	0.020	0.089	-0.015	0.000	0.174	-1.590	-3.200	-1.430
Q9QZQ8	H2afy	0.640	0.916	0.308	-0.121	0.000	0.027	0.161	0.210
Q3UV70	Pdp1	0.009	0.056	-0.168	0.000	0.071	0.763	0.401	0.775
Q922D8	Mthfd1	0.080	0.212	0.193	0.000	-0.406	-0.292	-0.898	-0.884
Q6ZQ38	Cand1	0.009	0.057	-0.021	0.028	0.000	-0.389	-0.813	-0.557
P02468	Lamc1	0.000	0.011	0.000	0.405	-0.653	4.850	4.390	4.910
Q9WUP7	Uchl5	0.001	0.022	0.106	0.000	-0.173	-0.684	-0.856	-0.786
O70133	Dhx9	0.008	0.054	-0.147	0.112	0.000	-0.357	-0.417	-0.383

P57776	Eef1d	0.010	0.058	0.131	-0.085	0.000	-0.355	-0.700	-0.662
Q9D1J1	Necap2	0.072	0.197	0.000	0.059	-0.305	-0.584	-0.378	-1.140
P51174	Acadl	0.039	0.132	0.000	0.343	-0.012	0.526	0.424	0.475
Q8VI75	Ipo4	0.133	0.296	0.315	-0.020	0.000	1.170	0.277	0.490
O55131	Sept 7	0.373	0.621	0.000	0.100	-0.111	0.425	-0.163	0.292
Q9D0F3	Lman1	0.008	0.054	0.000	0.082	-0.462	2.150	1.680	1.050
P06797	Ctsl	0.055	0.166	0.060	-0.053	0.000	-0.223	-0.865	-0.442
Q8K4Z3	Naxe	0.013	0.071	0.012	0.000	-0.125	0.324	0.340	0.156
P26638	Sars	0.134	0.298	0.000	0.015	-0.002	-0.210	-0.327	0.004
Q9ERK4	Cse1l	0.041	0.138	0.073	-0.029	0.000	-0.350	-1.430	-0.960
Q9R233	Tapbp	0.550	0.819	-0.278	0.096	0.000	-0.148	-0.045	-0.232
Q78ZA7	Nap1l4	0.009	0.056	-0.293	0.000	0.003	-0.919	-1.530	-0.967
Q9D7S9	Chmp5	0.646	0.923	0.226	-2.780	0.000	-0.323	-0.670	-0.105
Q8CHY6	Gatad2a	0.072	0.196	-0.214	0.000	0.239	-0.313	-1.050	-0.493
Q5SQX6	Cyfip2	0.133	0.297	0.000	-0.073	0.396	-0.157	-0.338	-0.099
Q61103	Dpf2	0.005	0.039	-0.147	0.000	0.059	-0.516	-0.628	-0.419
Q80TH2	Erbin	0.102	0.248	0.004	0.000	-0.191	0.359	-0.014	0.435
Q9DB29	Iah1	0.002	0.024	-0.084	0.000	0.019	-0.725	-1.100	-0.844
P08775	Polr2a	0.010	0.059	0.000	-0.066	0.050	-0.270	-0.476	-0.290
A2ADY9	Ddi2	0.042	0.140	0.078	-0.450	0.000	-0.584	-1.520	-1.060
Q9Z0M6	Cd97	0.222	0.423	0.067	-0.258	0.000	-0.186	-0.412	-0.150
Q99KI0	Aco2	0.135	0.299	0.000	0.042	-0.166	0.553	0.087	0.138
P26443	Glud1	0.007	0.048	-0.208	0.207	0.000	0.756	0.579	0.813
P01899	H2-D1	0.854	1.000	0.000	0.322	-0.021	0.070	0.088	0.212
Q9QWR8	Naga	0.347	0.588	0.711	-0.217	0.000	0.309	-0.481	-0.619
P56395	Cyb5a	0.102	0.247	0.000	0.774	-0.122	0.724	0.805	0.954
Q9QYC0	Add1	0.099	0.243	0.258	-0.225	0.000	-0.105	-0.792	-0.814
Q9DBS1	Tmem43	0.001	0.019	0.127	-0.398	0.000	1.340	1.310	1.460
Q8CCF0	Prpf31	0.340	0.579	0.000	-0.040	0.902	0.248	-0.333	-0.206
Q8BMF4	Dlat	0.002	0.025	0.000	0.245	-0.071	1.060	0.823	1.080
P70398	Usp9x	0.156	0.330	0.000	0.167	-0.051	0.059	-0.847	-0.528
P09103	P4hb	0.001	0.019	0.181	0.000	-0.258	1.550	1.180	1.470
P46062	Sipa1	0.122	0.280	0.000	-0.687	0.146	-1.190	-0.856	-0.453
Q8BRH0	Tmtc3	0.001	0.019	-0.049	0.564	0.000	2.350	1.990	2.490
Q9Z2I9	Sucla2	0.144	0.311	0.045	0.000	-0.226	0.968	0.063	0.320
Q9DCS9	Ndufb10	0.006	0.043	0.000	0.250	-0.106	0.628	0.918	0.805
P17751	Tpi1	0.001	0.019	-0.153	0.052	0.000	-1.010	-1.410	-1.170
Q99MN1	Kars	0.095	0.237	0.000	0.246	-0.487	-0.474	-0.558	-0.660
Q8C3X8	Lmf2	0.001	0.022	-0.449	0.291	0.000	1.800	2.180	2.370
Q8R123	Flad1	0.186	0.376	0.107	0.000	-0.001	-0.107	-0.914	-0.144
Q8VCT3	Rnpep	0.006	0.047	0.307	0.000	-0.067	-0.773	-1.480	-1.230
Q8BGW0	Themis	0.127	0.288	-1.320	0.000	0.443	-1.890	-1.260	-1.100
Q8CI94	Pygb	0.009	0.058	-0.215	0.120	0.000	-0.661	-1.230	-0.895
P70295	Aup1	0.223	0.424	-0.237	0.153	0.000	0.075	0.109	0.499
Q8BSY0	Asph	0.000	0.014	0.141	0.000	-0.607	3.050	2.490	2.970
Q91WJ8	Fubp1	0.460	0.719	-0.102	0.177	0.000	-0.201	-0.216	0.143
Q8VBT0	Tmx1	0.047	0.149	0.000	0.316	-0.123	0.858	0.556	0.392
P10852	Slc3a2	0.031	0.115	0.358	-0.051	0.000	1.070	0.533	0.706
Q6NV83	U2surp	0.018	0.083	0.000	0.005	-0.147	-0.422	-0.985	-0.714
Q61937	Npm1	0.054	0.164	0.268	-0.137	0.000	-0.286	-1.550	-1.340
Q9JMH6	Txnrd1	0.030	0.114	0.000	-0.115	0.093	-0.408	-1.270	-1.320
Q8VEK3	Hnrnpu	0.023	0.096	0.000	0.135	-0.052	-0.278	-0.465	-0.202
P62843	Rps15	0.638	0.914	0.152	-0.159	0.000	0.269	-0.156	0.113
P08207	S100a10	0.150	0.321	-1.010	0.000	0.570	0.678	0.482	0.974
Q9CSH3	Dis3	0.019	0.087	-0.143	0.044	0.000	-0.478	-0.855	-0.426
Q3UZA1	Rcsd1	0.010	0.060	0.278	-0.314	0.000	-0.995	-1.930	-1.520
Q8BP92	Rcn2	0.000	0.011	-0.104	0.495	0.000	3.300	2.950	3.320
P51150	Rab7a	0.539	0.807	0.095	0.000	-0.023	0.282	-0.085	0.100
O35226	Psmd4	0.124	0.283	0.193	0.000	-0.170	-0.493	-1.620	-0.319

P42669	Pura	0.003	0.033	-0.125	0.129	0.000	-0.642	-0.748	-0.504
Q9D2G2	Dlst	0.003	0.033	0.000	0.150	-0.204	0.862	0.626	0.867
Q9CRB2	Nhp2	0.145	0.314	0.135	-0.347	0.000	-0.114	-0.877	-0.684
Q9WV32	Arpc1b	0.004	0.034	0.000	-0.036	0.060	0.284	0.204	0.307
Q8CDM1	Atad2	0.200	0.394	-0.108	0.046	0.000	-0.369	-0.528	0.035
Q9D154	Serpinb1a	0.050	0.155	1.640	0.000	-0.016	-0.530	-1.950	-1.950
Q8BVE3	Atp6v1h	0.761	1.000	0.447	0.000	-0.035	0.476	-0.415	0.057
O09110	Map2k3	0.051	0.158	0.000	0.028	-0.378	0.174	0.286	0.414
P14206	Rpsa	0.136	0.301	0.104	-0.167	0.000	-0.043	-0.645	-0.462
P62849	Rps24	0.381	0.630	0.000	0.389	-0.243	0.128	0.548	0.148
Q8K224	Nat10	0.136	0.300	0.000	0.122	-0.081	0.017	-0.643	-0.503
P63101	Ywhaz	0.001	0.015	0.000	-0.021	0.113	-1.140	-1.610	-1.370
Q80ZK0	Mrps10	0.027	0.107	0.000	0.562	-0.484	1.350	0.904	1.690
O08547	Sec22b	0.089	0.229	-0.213	0.184	0.000	0.660	0.106	0.678
Q9WTQ5	Akap12	0.001	0.019	0.602	0.000	-1.060	4.110	3.850	4.090
P37040	Por	0.001	0.018	0.000	0.126	-0.066	0.875	0.704	0.940
P05064	Aldoa	0.039	0.132	0.022	-0.139	0.000	-0.265	-0.709	-0.415
Q8CGY8	Ogt	0.046	0.149	0.000	-0.266	0.000	-0.418	-0.946	-0.498
P00920	Ca2	0.051	0.158	0.887	0.000	-0.365	-0.645	-2.020	-2.740
Q9CRB9	Chchd3	0.005	0.039	-0.052	0.233	0.000	0.868	0.646	1.000
P63017	Hspa8	0.008	0.054	0.046	0.000	-0.090	-0.386	-0.759	-0.718
Q9DAM5	Slc25a19	0.512	0.776	0.000	0.235	-0.215	0.146	0.162	-1.530
Q8BG32	Psmd11	0.097	0.240	0.000	0.008	-0.152	-0.205	-0.649	-0.258
Q9Z2U0	Psma7	0.001	0.015	-0.029	0.109	0.000	-1.070	-1.380	-1.020
Q7M6Y3	Picalm	0.391	0.640	-0.430	1.660	0.000	1.050	1.440	0.683
Q8BVK9	Sp110	0.142	0.309	-0.296	0.000	0.048	-0.447	-0.417	-0.158
Q99J39	Mlycd	0.122	0.280	0.000	0.662	-0.127	0.733	0.678	0.583
A2AN08	Ubr4	0.208	0.404	0.203	0.000	-0.345	0.064	-0.983	-0.892
Q9EPK7	Xpo7	0.014	0.072	0.000	-0.014	0.137	-0.561	-1.410	-1.080
Q9DAW9	Cnn3	0.001	0.019	1.350	0.000	-0.597	6.050	5.170	5.740
Q9CQ65	Mtap	0.004	0.036	0.000	-0.105	0.108	-1.190	-1.490	-0.845
Q99LI7	Cstf3	0.246	0.457	-0.352	0.110	0.000	-0.471	-0.629	-0.048
Q9D0I9	Rars	0.267	0.485	0.171	0.000	-0.071	0.100	-0.634	-0.231
P08228	Sod1	0.000	0.013	-0.224	0.010	0.000	-1.080	-1.290	-1.170
Q99LC5	Etfa	0.310	0.542	0.000	0.213	-0.198	0.705	-0.164	0.467
Q6DIC0	Smarca2	0.021	0.090	0.000	0.161	-0.064	-0.474	-0.802	-0.336
Q8CFE3	Rcor1	0.012	0.068	0.000	-0.039	0.014	-0.534	-1.040	-0.568
P61965	Wdr5	0.036	0.125	0.000	-0.113	0.084	-0.312	-0.817	-0.427
Q9CQQ7	Atp5f1	0.000	0.014	0.081	0.000	-0.116	1.040	0.810	0.987
Q8R1Q8	Dync1li1	0.384	0.633	0.000	-0.009	0.173	0.263	-0.011	0.215
Q9CQF3	Nudt21	0.017	0.080	0.226	0.000	-0.079	-0.412	-0.346	-0.277
Q60864	Stip1	0.004	0.035	-0.047	0.027	0.000	-0.677	-1.220	-1.030
Q8CFI7	Polr2b	0.032	0.118	-0.057	0.000	0.025	-0.407	-0.600	-0.191
Q8K124	Plekho2	0.967	1.000	0.080	-0.182	0.000	0.317	-0.430	-0.020
Q8BH04	Pck2	0.009	0.056	0.068	0.000	-0.323	1.320	0.741	0.823
Q99PT1	Arhgdia	0.005	0.039	-0.050	0.000	0.040	-1.120	-1.610	-0.907
Q9JL26	Fmnl1	0.003	0.033	-0.034	0.000	0.049	-0.746	-1.290	-0.945
Q9Z0V7	Timm17b	0.705	0.982	0.000	-0.059	0.096	0.339	-0.298	0.243
Q9WTQ8	Timm23	0.001	0.017	0.000	0.198	-0.057	0.799	0.774	0.865
P28352	Apex1	0.028	0.107	0.000	-0.288	0.047	-0.452	-0.881	-0.582
O88874	Ccnk	0.240	0.449	0.303	-0.212	0.000	-1.210	-0.367	0.032
Q8R2E9	Ero1b	0.121	0.278	0.069	0.000	-0.488	-0.320	-0.915	-0.631
Q80XN0	Bdh1	0.208	0.404	0.000	0.147	-0.057	0.047	-0.615	-0.246
Q9CWJ9	Atic	0.007	0.048	0.044	-0.094	0.000	-0.791	-1.580	-1.370
Q3TEA8	Hp1bp3	0.107	0.256	-0.152	0.000	0.110	-0.294	-0.484	-0.100
Q61081	Cdc37	0.002	0.027	0.019	0.000	-0.009	-0.837	-1.370	-1.050
Q61543	Glg1	0.076	0.203	-0.003	1.010	0.000	1.350	1.020	1.130
P19426	Nelfe	0.017	0.081	-0.074	0.000	0.154	-0.345	-0.656	-0.356
P27612	Plaa	0.023	0.096	-0.191	0.007	0.000	-0.710	-1.900	-1.320

Q9CXI5	Manf	0.067	0.186	0.000	0.366	-0.328	0.585	0.395	0.788
Q3TCN2	Plbd2	0.916	1.000	0.612	-0.005	0.000	0.484	-0.125	0.341
P27546	Map4	0.159	0.336	-0.314	0.000	0.103	0.247	0.014	0.453
A2AR02	Ppig	0.007	0.048	0.137	0.000	-0.311	-0.770	-1.200	-1.030
Q9DCH4	Eif3f	0.200	0.395	0.000	0.017	-0.327	-0.119	-0.590	-0.416
Q61598	Gdi2	0.000	0.013	-0.038	0.000	0.024	-0.676	-0.913	-0.798
Q8BVI4	Qdpr	0.149	0.319	0.000	-0.293	0.016	-0.220	-0.562	-0.276
Q9CQ62	Decr1	0.431	0.687	-0.053	0.110	0.000	0.728	-0.038	0.024
P58681	Tlr7	0.683	0.960	0.829	-0.061	0.000	0.779	0.114	0.332
Q6GV12	Kdsr	0.056	0.168	-1.720	0.162	0.000	0.995	0.983	1.380
Q8VEK0	Tmem30a	0.141	0.308	0.264	0.000	-0.064	0.477	0.148	0.337
Q8CDG3	Vcpip1	0.072	0.197	-0.095	0.062	0.000	-0.188	-1.310	-0.985
P62754	Rps6	0.169	0.350	0.062	-0.038	0.000	0.478	-0.019	0.316
Q8VBZ3	Clptm1	0.007	0.050	0.000	0.091	-0.179	0.984	0.517	0.882
Q8VDM6	Hnrnpul1	0.168	0.350	0.000	0.612	-0.432	-0.761	-0.381	-0.344
Q569Z5	Ddx46	0.021	0.092	0.180	-0.247	0.000	-0.684	-1.790	-1.560
O35286	Dhx15	0.013	0.071	-0.082	0.000	0.065	-0.392	-0.774	-0.454
Q9JM14	Nt5c	0.002	0.023	-0.329	0.393	0.000	-1.550	-1.680	-1.570
O08539	Bin1	0.015	0.076	-0.300	0.232	0.000	-0.726	-1.520	-1.220
Q9WV85	Nme3	0.093	0.234	0.001	-0.129	0.000	-0.343	-0.578	-0.122
Q91YW3	Dnajc3	0.001	0.022	0.000	0.021	-0.207	1.010	0.731	0.763
Q9CT10	Ranbp3	0.004	0.038	-0.211	0.000	0.146	-1.020	-1.690	-1.230
Q8K1B8	Fermt3	0.253	0.466	0.000	0.165	-0.366	-0.172	-0.300	-0.423
P47962	Rpl5	0.228	0.432	0.125	-0.082	0.000	0.097	-0.370	-0.689
Q9Z1G4	Atp6v0a1	0.225	0.427	0.713	-0.329	0.000	2.070	-0.094	1.480
Q9EQK5	Mvp	0.055	0.166	-0.217	0.000	0.037	0.473	0.098	0.340
Q99J99	Mpst	0.334	0.571	0.163	0.000	-0.160	0.378	-0.932	-0.891
Q9D967	Mdp1	0.009	0.057	-0.283	0.000	0.020	-2.080	-3.970	-2.490
O88487	Dync1i2	0.412	0.665	0.000	0.172	-0.041	0.321	-0.021	0.156
Q8C129	Lnpep	0.689	0.965	0.030	0.000	-0.056	0.124	-0.155	0.131
Q3UQ84	Tars2	0.008	0.053	0.000	0.010	-0.095	0.543	0.329	0.293
Q8CD10	Micu2	0.000	0.014	-0.023	0.080	0.000	1.030	0.770	0.952
P61290	Psme3	0.001	0.021	0.000	0.420	-0.049	-1.120	-1.150	-1.340
Q8JZN5	Acad9	0.215	0.412	0.000	0.153	-0.210	0.571	0.123	0.083
Q8CJF7	Ahctf1	0.969	1.000	-0.733	0.000	0.004	-0.494	-0.354	0.158
P35293	Rab18	0.022	0.093	0.000	0.058	-0.235	0.574	0.244	0.439
Q9JLI8	Sart3	0.010	0.058	0.000	0.228	-0.111	-0.934	-1.600	-0.875
Q08288	Lyar	0.211	0.408	0.005	0.000	-0.310	-0.096	-0.931	-0.453
P53994	Rab2a	0.195	0.387	0.000	0.207	-0.108	0.262	0.082	0.656
P62774	Mtpn	0.005	0.041	0.000	-0.056	0.340	-0.915	-1.670	-1.290
Q7TQI3	Otub1	0.007	0.048	0.000	-0.084	0.037	-0.752	-1.500	-1.260
P58252	Eef2	0.192	0.384	0.070	0.000	-0.273	-0.046	-0.716	-0.492
Q91V61	Sfxn3	0.006	0.043	-0.166	0.192	0.000	0.721	0.546	0.762
Q68FD5	Cltc	0.377	0.625	0.000	0.035	-0.230	0.063	-0.437	-0.339
P68510	Ywhah	0.001	0.015	-0.038	0.020	0.000	-0.755	-1.060	-0.878
Q91WD5	Ndufs2	0.006	0.045	0.000	0.406	-0.118	0.946	0.914	1.040
P27773	Pdia3	0.006	0.047	0.000	0.162	-0.091	0.836	0.494	0.787
Q9R0E2	Plod1	0.000	0.010	0.686	-0.258	0.000	4.510	4.250	4.540
Q9JI11	Stk4	0.009	0.057	0.000	-0.122	0.047	-0.744	-1.550	-1.450
A2BH40	Arid1a	0.092	0.233	-0.206	0.044	0.000	-0.488	-0.528	-0.129
Q9ERR7	Sep 15	0.054	0.164	0.000	0.000	0.112	0.582	0.658	0.160
Q8BKZ9	Pdhx	0.019	0.086	0.000	0.072	-0.571	0.880	0.548	0.654
P00405	Mtco2	0.007	0.048	0.000	0.129	-0.089	0.646	0.391	0.643
Q3UVK0	Ermp1	0.187	0.376	0.000	0.130	-0.198	0.330	-0.022	0.382
Q8BL66	Eea1	0.108	0.259	-0.525	0.502	0.000	-0.939	-0.986	-0.339
P40336	Vps26a	0.557	0.825	0.032	0.000	-0.325	0.061	-0.478	-0.248
O08795	Prkcsh	0.006	0.047	0.000	0.339	-0.092	0.821	0.722	0.867
Q9WVK4	Ehd1	0.325	0.561	0.320	0.000	-0.219	0.523	0.037	0.249
Q8VCF0	Mavs	0.900	1.000	-0.276	0.000	0.068	-0.034	-0.283	0.051

P62301	Rps13	0.889	1.000	0.000	0.263	-0.044	-0.105	0.013	0.390
O08917	Flot1	0.054	0.164	0.190	-0.046	0.000	0.567	0.218	0.752
Q9D898	Arpc5l	0.171	0.353	-0.177	0.054	0.000	0.015	0.105	0.281
A2AJI0	Map7d1	0.249	0.462	-0.128	0.647	0.000	1.090	0.041	1.120
Q9JMA1	Usp14	0.074	0.201	0.000	0.381	-0.373	-0.303	-0.956	-1.110
Q60930	Vdac2	0.002	0.023	0.000	0.161	-0.063	1.040	0.946	1.360
P62900	Rpl31	0.502	0.767	0.126	-0.033	0.000	0.385	-0.004	0.012
F8VPU2	Farp1	0.001	0.014	-0.677	0.000	0.355	4.280	3.500	3.700
Q9JMG7	Hdgfrp3	0.136	0.301	-0.347	0.000	0.147	-0.879	-0.986	-0.099
Q922L6	Nelfcd	0.012	0.066	0.000	-0.045	0.091	-0.415	-0.543	-0.247
Q52KI8	Srrm1	0.120	0.276	0.238	-0.450	0.000	-0.234	-2.830	-1.770
Q8BG81	Poldip3	0.098	0.242	-0.525	0.000	0.195	-0.771	-0.787	-0.391
Q921Z5	Tnfaip8	0.082	0.214	0.388	0.000	-0.292	-0.278	-0.792	-0.547
Q3UJU9	Rmdn3	0.332	0.570	0.215	0.000	-0.020	-0.077	-0.868	0.115
Q8CB44	Gramd4	0.430	0.686	0.000	0.225	-0.025	0.064	0.109	0.327
Q922K7	Nop2	0.086	0.223	0.000	0.003	-0.303	-0.235	-0.800	-0.577
P49717	Mcm4	0.023	0.097	0.346	0.000	-0.046	-0.328	-0.924	-0.787
Q3UHX2	Pdap1	0.179	0.365	0.000	0.188	-0.220	-0.300	-0.993	-0.140
Q9R1P1	Psmb3	0.004	0.035	0.033	-0.340	0.000	-1.020	-1.120	-0.826
Q61941	Nnt	0.000	0.013	1.030	-0.638	0.000	5.910	5.440	5.700
P52293	Kpna2	0.813	1.000	0.707	0.000	-0.101	0.887	-0.132	0.151
Q05512	Mark2	0.392	0.641	0.223	-0.177	0.000	0.293	-0.698	-0.474
Q5XJY5	Arcn1	0.085	0.221	0.106	-0.155	0.000	-0.126	-0.943	-0.727
Q99JR8	Smarcd2	0.103	0.250	0.000	-0.058	0.257	-0.113	-0.328	-0.116
Q9D0T1	Snu13	0.001	0.016	0.000	0.041	-0.075	-0.503	-0.523	-0.409
Q8BFZ9	Erlin2	0.006	0.047	0.000	0.127	-0.748	1.380	1.200	1.190
Q9D0S9	Hint2	0.816	1.000	0.000	1.130	-1.100	-0.268	1.220	-0.316
P21107	Tpm3	0.005	0.039	0.000	0.179	-0.001	-0.958	-1.660	-1.080
Q921S7	Mrpl37	0.249	0.462	0.174	0.000	-0.144	0.783	-0.030	0.302
Q3V3R1	Mthfd1l	0.570	0.839	0.201	0.000	-0.215	0.497	-0.751	-0.501
Q91VW3	Sh3bgrl3	0.003	0.033	-0.165	0.000	0.130	-1.470	-2.560	-2.250
E9Q555	Rnf213	0.959	1.000	0.447	0.000	-0.205	0.553	-0.424	0.057
Q8K411	Pitrm1	0.003	0.030	0.219	0.000	-0.224	1.320	0.923	1.190
Q9D1L0	Chchd2	0.596	0.869	-0.345	0.000	0.075	0.112	-1.140	0.029
Q9QYR9	Acot2	0.001	0.018	-0.148	0.041	0.000	0.854	0.676	0.934
P16858	Gapdh	0.043	0.142	-0.051	0.026	0.000	-0.153	-0.557	-0.357
P61222	Abce1	0.300	0.529	0.517	-0.034	0.000	0.313	-0.505	-0.467
P61161	Actr2	0.282	0.505	0.071	-0.134	0.000	0.212	-0.043	0.126
Q8VCH8	Ubxn4	0.013	0.069	0.099	0.000	-0.026	0.633	0.446	0.951
O55023	Impa1	0.000	0.013	-0.248	0.000	0.009	-1.540	-1.950	-1.710
P70248	Myo1f	0.137	0.302	0.249	-0.171	0.000	-0.054	-0.811	-0.449
Q9JHW2	Nit2	0.309	0.542	-0.141	0.431	0.000	0.296	0.245	0.362
Q9JHW4	Eefsec	0.087	0.224	0.249	-0.072	0.000	-0.272	-1.360	-0.515
O88736	Hsd17b7	0.000	0.004	0.000	0.367	-0.023	4.050	3.850	4.130
P57759	Erp29	0.022	0.094	0.000	0.074	-0.443	0.673	0.369	0.667
O35654	Pold2	0.012	0.068	-0.024	0.000	0.141	-0.399	-0.668	-0.328
Q62419	Sh3gl1	0.450	0.708	0.000	-0.110	0.019	0.287	-0.209	0.241
P61082	Ube2m	0.204	0.400	0.058	-0.064	0.000	0.007	-0.882	-0.322
Q8K1E0	Stx5	0.141	0.307	0.053	-0.343	0.000	0.073	0.123	0.426
Q60715	P4ha1	0.000	0.008	0.000	0.805	-0.211	6.380	6.030	6.340
Q9D8Y0	Efhd2	0.003	0.033	0.045	0.000	-0.014	-0.957	-1.570	-1.060
Q9R0P5	Dstn	0.888	1.000	0.713	0.000	-1.130	0.571	-0.381	-0.332
Q9QYC7	Ggcx	0.000	0.003	-0.235	0.000	0.166	5.000	4.810	5.100
Q6ZWX6	Eif2s1	0.438	0.695	0.263	0.000	-0.164	0.248	-0.476	-0.325
Q9DB20	Atp5o	0.001	0.019	0.000	0.129	-0.067	0.904	0.673	0.901
Q8BR65	Suds3	0.139	0.304	0.057	0.000	-0.089	-0.026	-0.482	-0.292
Q9QZ88	Vps29	0.132	0.296	0.190	-0.256	0.000	-0.377	-1.590	-0.445
O08709	Prdx6	0.003	0.031	0.085	0.000	-0.156	-1.090	-1.810	-1.670
Q9CQ22	Lamtor1	0.492	0.756	0.000	-0.355	0.051	0.095	-0.158	0.109

Q8BIJ6	Iars2	0.008	0.053	0.000	0.073	-0.104	0.819	0.408	0.655
Q8CIN4	Pak2	0.001	0.022	-0.049	0.000	0.006	-1.000	-1.520	-1.160
Q9CZW4	Acsl3	0.001	0.022	0.554	-0.639	0.000	3.110	2.640	3.120
Q61739	Itga6	0.274	0.495	0.000	0.607	-0.319	-0.246	-0.143	-0.388
Q8K2Z4	Ncapd2	0.437	0.694	0.059	-0.356	0.000	0.217	-0.607	-0.761
Q91YQ5	Rpn1	0.001	0.018	0.000	0.016	-0.293	1.400	1.040	1.180
Q9DC61	Pmpca	0.012	0.068	0.000	0.351	-0.158	1.180	0.802	0.788
Q3UJB9	Edc4	0.867	1.000	0.128	-0.076	0.000	0.309	-0.341	-0.021
P18242	Ctsd	0.694	0.969	0.717	0.000	-0.835	0.208	-0.516	-0.451
Q99L13	Hibadh	0.000	0.014	0.000	0.215	-0.163	1.320	1.240	1.450
Q03265	Atp5a1	0.002	0.025	0.000	0.050	-0.113	0.804	0.540	0.809
Q9QXX4	Slc25a13	0.004	0.038	-0.014	0.047	0.000	0.518	0.393	0.699
O54879	Hmgb3	0.121	0.278	0.188	-0.126	0.000	-0.298	-0.889	-0.166
Q9QY06	Myo9b	0.117	0.273	0.000	-0.054	0.168	0.018	-0.602	-0.651
Q9Z1F9	Uba2	0.005	0.042	0.000	-0.064	0.102	-1.020	-1.890	-1.320
P62855	Rps26	0.563	0.832	0.024	-0.672	0.000	0.355	-0.968	-0.977
P63005	Pafah1b1	0.015	0.075	0.000	0.142	-0.104	-0.432	-0.897	-0.558
Q6PGG2	Gmip	0.008	0.055	0.062	0.000	-0.212	-1.290	-1.810	-0.933
O35855	Bcat2	0.331	0.569	0.049	0.000	-0.484	0.516	-0.134	0.039
P29351	Ptpn6	0.095	0.238	0.228	-0.143	0.000	-0.147	-0.756	-0.370
Q8BPG6	Sumf2	0.000	0.013	-0.094	0.054	0.000	0.610	0.632	0.744
Q9CQC7	Ndufb4	0.005	0.039	0.000	0.250	-0.034	0.719	0.760	0.569
Q99KH8	Stk24	0.035	0.123	-0.121	0.133	0.000	-0.235	-0.505	-0.703
Q9Z2N8	Actl6a	0.006	0.047	-0.016	0.057	0.000	-0.380	-0.667	-0.402
Q91ZV0	Mia2	0.257	0.472	-0.554	0.166	0.000	0.561	-0.135	0.378
Q8CGK3	Lonp1	0.159	0.335	0.242	0.000	-0.158	1.280	0.204	0.415
Q8C4J7	Tbl3	0.049	0.154	0.189	0.000	-0.100	-0.174	-0.628	-0.759
Q8VEM8	Slc25a3	0.001	0.019	0.000	0.026	-0.130	1.170	0.803	0.950
P60762	Morf4l1	0.009	0.057	-0.318	0.087	0.000	-0.962	-0.761	-0.658
Q9D8U8	Snx5	0.176	0.360	0.255	0.000	-0.005	0.060	-0.518	-0.216
Q9Z2U1	Psma5	0.001	0.018	-0.148	0.000	0.027	-0.997	-1.330	-1.400
P27641	Xrcc5	0.003	0.030	-0.102	0.000	0.052	-1.050	-1.770	-1.530
Q80UU9	Pgrmc2	0.036	0.125	0.101	-1.420	0.000	1.290	0.890	1.240
Q9D051	Pdhb	0.332	0.570	0.000	0.004	-0.255	0.792	-0.134	0.064
P47758	Srprb	0.009	0.057	-0.191	0.223	0.000	0.773	0.554	0.890
Q9CYR0	Ssbp1	0.351	0.592	0.000	0.078	0.000	0.385	-0.069	0.185
Q91ZX7	Lrp1	0.002	0.025	0.234	0.000	-1.060	2.740	2.500	2.710
Q9D1P4	Chordc1	0.031	0.116	0.000	-0.375	0.231	-0.843	-1.750	-0.898
Q8VCB1	Ndc1	0.083	0.216	-0.054	0.125	0.000	0.620	0.106	0.452
P62889	Rpl30	0.194	0.386	0.236	-0.137	0.000	0.653	0.049	0.359
Q64521	Gpd2	0.010	0.061	0.000	0.125	-0.395	0.903	0.600	0.693
O70152	Dpm1	0.009	0.058	-0.055	0.148	0.000	0.621	0.542	0.972
Q64727	Vcl	0.889	1.000	0.423	0.000	-0.698	0.350	-0.352	-0.100
Q61792	Lasp1	0.591	0.864	-0.323	0.046	0.000	0.162	-0.267	0.147
O88532	Zfr	0.756	1.000	-0.374	0.000	0.242	-0.033	-0.343	0.030
Q64012	Raly	0.019	0.086	0.000	0.086	-0.035	-0.193	-0.356	-0.175
Q9EPE9	Atp13a1	0.000	0.013	0.082	0.000	-0.043	0.982	1.020	1.240
Q08093	Cnn2	0.711	0.987	-0.069	0.221	0.000	0.526	-0.210	0.112
Q9Z0X1	Aifm1	0.917	1.000	0.000	0.036	-0.025	0.160	-0.134	0.015
Q8K297	Colgalt1	0.000	0.013	0.020	0.000	-0.798	3.140	2.810	2.900
P27601	Gna13	0.966	1.000	-0.229	0.139	0.000	0.035	-0.168	0.062
P68372	Tubb4b	0.036	0.125	0.000	0.073	-0.162	-0.272	-0.840	-0.694
Q01853	Vcp	0.002	0.025	0.000	0.056	-0.134	-0.465	-0.616	-0.590
Q8K1R3	Pnpt1	0.333	0.571	0.128	0.000	-0.456	1.050	0.609	-0.428
Q6NS46	Pdcd11	0.115	0.270	0.288	0.000	-0.052	0.029	-0.613	-0.698
Q91V92	Acly	0.836	1.000	0.521	0.000	-0.217	0.528	-0.126	0.096
Q61687	Atrx	0.020	0.089	-0.063	0.030	0.000	-0.511	-1.080	-0.541
O35887	Calu	0.000	0.010	0.000	0.381	-0.028	2.430	2.320	2.650
Q8VBV7	Cops8	0.011	0.063	-0.099	0.297	0.000	-0.536	-0.993	-1.100

Q9D892	Itpa	0.015	0.075	0.000	0.571	-0.294	-0.901	-1.130	-1.500
P32067	Ssb	0.024	0.099	0.085	0.000	-0.109	-0.360	-1.020	-0.784
Q8BI72	Cdkn2aip	0.009	0.058	-0.178	0.025	0.000	-0.523	-0.712	-0.421
P97372	Psme2	0.004	0.035	-0.129	0.542	0.000	-1.040	-1.290	-1.300
Q9WUM5	Suclg1	0.579	0.850	0.279	0.000	-0.064	1.160	-0.031	-0.140
P97452	Bop1	0.146	0.315	0.031	0.000	-0.439	-0.274	-0.489	-0.693
Q6WVG3	Kctd12	0.656	0.932	0.315	0.000	-0.034	0.280	-0.225	-0.040
Q9CXW3	Cacybp	0.796	1.000	0.000	0.321	-0.259	0.140	-0.448	0.154
Q8C7V8	Ccdc134	0.086	0.222	0.343	-0.195	0.000	1.030	0.218	1.050
Q9EPU0	Upf1	0.254	0.467	0.070	0.000	-0.442	-0.174	-0.567	-0.419
Q8K2B3	Sdha	0.017	0.081	0.000	0.002	-0.204	0.722	0.297	0.454
P67778	Phb	0.003	0.033	0.000	0.017	-0.309	0.843	0.579	0.735
Q6NZJ6	Eif4g1	0.192	0.384	0.001	-0.411	0.000	0.581	-0.101	0.237
P35235	Ptpn11	0.002	0.029	-0.286	0.010	0.000	-0.906	-1.190	-0.916
Q922Q4	Pycr2	0.016	0.078	0.102	0.000	-0.106	1.190	0.520	0.764
P47753	Capza1	0.230	0.435	-0.227	0.804	0.000	-0.433	-0.018	-0.420
Q61102	Abcb7	0.585	0.857	-0.410	0.169	0.000	0.145	-0.268	0.316
P47802	Mtx1	0.295	0.522	0.000	0.014	-0.282	0.283	-0.076	0.042
P03958	Ada	0.014	0.072	0.754	-0.343	0.000	-1.270	-2.090	-1.410
P63321	Rala	0.001	0.019	0.000	0.267	-0.010	1.050	1.270	1.390
P27808	Mgat1	0.011	0.063	0.341	-0.109	0.000	1.110	0.673	1.070
Q91V08	Clec2d	0.595	0.868	0.000	4.180	-0.694	-1.230	2.370	-0.961
P21279	Gnaq	0.001	0.019	0.000	0.266	-0.175	1.290	1.150	1.370
Q3TWW8	Srsf6	0.078	0.209	0.100	0.000	-0.133	-0.141	-1.130	-1.060
Q80WQ2	Vac14	0.093	0.234	0.126	0.000	-0.005	-0.024	-0.634	-0.440
P19157	Gstp1	0.004	0.036	-0.138	0.000	0.106	-1.050	-1.380	-0.809
Q99JF8	Psip1	0.090	0.230	-0.095	0.000	0.152	-0.474	-0.546	-0.054
Q9EPU4	Cpsf1	0.146	0.315	0.000	0.097	-0.063	-0.601	-0.441	0.028
P61028	Rab8b	0.484	0.747	0.000	0.106	-1.460	-0.015	-0.228	0.075
Q9EQH2	Erap1	0.628	0.904	-0.026	0.272	0.000	0.049	-0.104	0.119
P63276	Rps17	0.251	0.463	0.554	-0.143	0.000	0.393	0.274	1.260
P08249	Mdh2	0.023	0.096	-0.067	0.250	0.000	0.700	0.373	0.737
Q8C0E2	Vps26b	0.098	0.242	0.000	-0.173	0.155	-0.331	-1.450	-0.538
P06537	Nr3c1	0.043	0.141	-0.291	0.000	0.306	-0.491	-0.996	-0.556
P84096	Rhog	0.210	0.406	0.099	-0.086	0.000	-0.027	-0.680	-0.190
Q8K273	Mmgt1	0.040	0.135	0.123	-0.229	0.000	1.120	0.385	1.530
Q6P5D8	Smchd1	0.071	0.194	-0.012	0.080	0.000	-0.246	-0.640	-0.156
Q99KC8	Vwa5a	0.041	0.136	-0.167	0.112	0.000	0.212	0.207	0.290
Q6A0A9	FAM120A	0.722	1.000	-0.005	0.168	0.000	0.747	-0.800	-0.310
P26231	Ctnna1	0.001	0.018	0.052	-0.640	0.000	3.370	2.460	2.860
Q6ZPE2	Sbf1	0.006	0.044	0.052	-0.183	0.000	-0.773	-1.370	-1.020
Q9Z1K5	Arih1	0.021	0.092	0.126	-0.138	0.000	-0.818	-2.130	-1.350
Q3UUQ7	Pgap1	0.013	0.072	0.463	-0.528	0.000	1.170	1.170	1.220
P42125	Eci1	0.192	0.384	0.000	0.429	-0.075	0.361	0.416	0.326
Q8C9B9	Dido1	0.033	0.120	-0.193	0.041	0.000	0.473	0.154	0.515
Q80U72	Scrib	0.649	0.926	-0.386	0.062	0.000	1.500	-0.647	-0.191
O35143	Atpif1	0.013	0.069	0.259	0.000	-0.321	1.590	1.150	0.813
Q8K310	Matr3	0.033	0.119	-0.160	0.000	0.057	-0.247	-0.430	-0.265
Q8BXZ1	Tmx3	0.000	0.008	-0.015	0.000	0.010	0.683	0.570	0.683
Q9JJT0	Rcl1	0.006	0.044	-0.094	0.000	0.033	-0.925	-1.080	-1.640
P35278	Rab5c	0.471	0.730	-0.070	0.126	0.000	0.187	-0.097	0.261
Q91VR5	Ddx1	0.525	0.791	0.054	0.000	-0.008	0.345	-0.164	0.182
P84084	Arf5	0.092	0.233	0.000	0.100	-0.067	-0.116	-1.250	-0.806
P14685	Psmd3	0.048	0.152	0.000	-0.102	0.036	-0.240	-0.752	-0.399
P80315	Cct4	0.030	0.114	-0.089	0.028	0.000	-0.246	-0.738	-0.523
Q7TPD0	Ints3	0.037	0.128	0.000	-0.118	0.448	-0.365	-1.040	-0.669
O88531	Ppt1	0.102	0.248	0.288	0.000	-0.559	-0.350	-1.070	-1.500
Q921M3	Sf3b3	0.003	0.031	-0.017	0.117	0.000	-0.436	-0.637	-0.422
Q8VH51	Rbm39	0.044	0.143	0.079	0.000	-0.126	-0.211	-0.764	-0.586

Q8BHN3	Ganab	0.006	0.047	0.180	0.000	-0.140	0.719	0.487	0.700
Q9CZW5	Tomm70	0.062	0.179	0.000	0.008	-0.151	0.239	0.035	0.226
O35972	Mrpl23	0.003	0.033	0.000	0.107	-0.240	0.886	0.665	0.669
P62315	Snrpd1	0.645	0.922	0.000	-1.410	0.030	-0.557	-0.619	0.778
O35350	Capn1	0.018	0.084	-0.014	0.000	0.021	-0.236	-0.579	-0.351
Q60737	Csnk2a1	0.841	1.000	0.000	0.031	-0.134	-0.077	-0.483	0.308
P97376	Frg1	0.010	0.059	0.020	-0.044	0.000	-0.541	-0.851	-0.422
P68037	Ube2l3	0.133	0.297	0.000	0.339	-0.029	-0.282	-1.350	-0.224
P54103	Dnajc2	0.094	0.236	0.144	0.000	0.000	-0.125	-0.980	-0.423
O08915	Aip	0.025	0.102	0.016	-0.067	0.000	-0.672	-1.950	-1.290
O35841	Api5	0.032	0.116	-0.044	0.000	0.040	-0.768	-0.726	-0.225
Q8CHP6	Phc3	0.947	1.000	-0.400	0.000	0.610	0.040	0.081	0.027
Q8K284	Gtf3c1	0.105	0.252	0.000	0.163	-0.024	-0.119	-1.060	-0.453
Q09014	Ncf1	0.407	0.658	1.740	-0.022	0.000	1.230	-1.690	-0.706
P81269	Atf1	0.311	0.544	0.000	-2.120	0.502	0.421	-0.113	1.130
O35343	Kpna4	0.217	0.415	0.000	0.365	-0.431	-1.080	-0.695	-0.005
Q9CSU0	Rprd1b	0.000	0.009	-0.009	0.058	0.000	-0.946	-1.160	-1.000
Q3UQ44	Iqgap2	0.285	0.509	0.213	0.000	-0.073	0.235	-0.465	-0.837
Q6P5F9	Xpo1	0.204	0.400	0.004	0.000	-0.804	-0.562	-0.972	-0.620
Q9D0D3	Mtpap	0.039	0.134	-0.042	0.000	0.411	0.553	0.544	0.831
P51859	Hdgf	0.004	0.034	-0.010	0.000	0.062	-0.903	-1.530	-1.040
P11942	Cd3g	0.167	0.349	-0.367	0.000	0.062	-0.670	-0.495	-0.158
Q91VE6	Nifk	0.244	0.454	0.009	0.000	-0.055	0.059	-0.834	-0.332
O88543	Cops3	0.037	0.128	-0.261	0.000	0.061	-0.532	-1.550	-0.983
Q9D6S7	Mrrf	0.086	0.223	0.000	0.433	-0.651	0.785	0.917	0.434
Q9CXT8	Pmpcb	0.017	0.082	0.000	0.156	-0.441	1.050	0.643	0.643
O09172	Gclm	0.514	0.778	0.497	0.000	-0.914	0.147	-0.892	-0.812
O70370	Ctss	0.016	0.078	0.174	0.000	-0.123	-0.289	-0.491	-0.485
Q9ES52	Inpp5d	0.069	0.191	-0.054	0.183	0.000	-0.248	-0.967	-0.380
Q8CHT0	Aldh4a1	0.875	1.000	0.335	-0.067	0.000	1.090	-0.715	-0.397
O35326	Srsf5	0.051	0.157	0.057	0.000	-0.537	-0.598	-1.720	-1.290
P61255	Rpl26	0.969	1.000	0.146	0.000	-0.044	0.302	-0.198	-0.021
Q9WV54	Asah1	0.436	0.693	0.241	0.000	-0.799	-0.033	-0.720	-0.851
Q5SWD9	Tsr1	0.007	0.048	0.044	-0.144	0.000	-0.590	-0.766	-0.441
Q9Z0N1	Eif2s3x	0.715	0.992	0.118	0.000	-0.435	0.405	-0.210	-0.202
Q9JIG8	Praf2	0.001	0.022	-0.152	0.388	0.000	1.550	1.310	1.510
P17742	Ppia	0.001	0.020	-0.069	0.072	0.000	-0.744	-1.100	-0.888
Q9D1N9	Mrpl21	0.001	0.019	0.000	0.089	-0.218	0.892	0.811	1.010
P21460	Cst3	0.344	0.583	0.555	0.000	-0.002	0.303	-0.365	-0.291
Q8QZT1	Acat1	0.050	0.156	0.000	0.181	-0.006	0.765	0.228	0.592
P60843	Eif4a1	0.768	1.000	0.365	0.000	-0.115	0.496	-0.360	-0.167
Q9DCS3	Mecr	0.121	0.279	0.000	0.207	-0.206	1.210	0.269	0.392
Q9QUI0	Rhoa	0.092	0.233	0.000	-0.019	0.008	-0.050	-0.555	-0.397
Q7TPH6	Mycbp2	0.256	0.470	0.044	-0.016	0.000	0.048	-0.906	-0.239
Q8C0C0	Zhx2	0.138	0.303	-0.142	0.000	0.004	-0.455	-0.390	-0.046
B2RXR6	Ankrd44	0.004	0.036	0.067	-0.178	0.000	-1.230	-2.070	-1.460
O89053	Coro1a	0.001	0.021	-0.088	0.000	0.009	-0.623	-0.917	-0.740
Q9Z0E6	Gbp2	0.096	0.238	-0.595	0.652	0.000	-0.971	-1.400	-0.481
Q8VCM8	Ncln	0.000	0.013	0.163	0.000	-0.176	2.230	1.750	2.080
P39054	Dnm2	0.326	0.562	0.075	-0.001	0.000	0.448	-0.098	0.271
O55106	Strn	0.053	0.162	-0.090	0.271	0.000	-0.484	-0.533	-0.141
Q9QX47	Son	0.342	0.582	-0.273	0.117	0.000	-0.128	-0.274	-0.155
Q9D883	U2af1	0.235	0.442	0.393	-0.112	0.000	0.274	-0.934	-0.764
Q7TMR0	Prcp	0.537	0.805	0.534	0.000	-0.186	0.295	-0.429	-0.127
Q8JZQ9	Eif3b	0.260	0.476	0.275	-0.073	0.000	0.153	-0.697	-0.305
Q3V0C5	Usp48	0.002	0.023	-0.188	0.240	0.000	-1.040	-1.390	-1.230
Q922B2	Dars	0.310	0.543	0.005	0.000	-0.176	-0.007	-0.316	-0.229
Q3THK3	Gtf2f1	0.002	0.024	0.000	0.200	-0.068	-1.120	-1.740	-1.450
Q8CFX1	H6pd	0.003	0.031	0.000	0.021	-0.537	1.370	1.020	1.180

Q8K1I7	Wipf1	0.146	0.315	-0.001	0.000	0.402	-0.311	-0.419	0.062
Q4VA53	Pds5b	0.031	0.116	-0.082	0.000	0.035	-0.347	-0.548	-0.198
Q7TPV4	Mybbp1a	0.078	0.208	0.135	0.000	-0.117	-0.129	-0.809	-0.532
Q3ULD5	Mccc2	0.004	0.036	0.000	0.315	-0.197	1.080	0.893	0.981
Q8BJS4	Sun2	0.725	1.000	0.000	0.079	-0.089	0.028	-0.229	0.073
Q64324	Stxbp2	0.030	0.112	0.000	0.170	-0.102	-0.258	-0.789	-0.715
Q9CWK8	Snx2	0.007	0.050	0.074	0.000	-0.016	-0.329	-0.673	-0.635
Q9DCC8	Tomm20	0.641	0.917	-0.091	0.350	0.000	0.068	0.058	0.398
O88342	Wdr1	0.001	0.019	-0.112	0.000	0.084	-0.978	-1.390	-1.190
P55096	Abcd3	0.092	0.232	0.042	0.000	-0.929	1.360	1.060	0.053
Q80W54	Zmpste24	0.080	0.211	0.027	0.000	-0.220	0.715	0.060	0.482
P32020	Scp2	0.103	0.248	0.172	0.000	-0.126	0.611	0.091	0.465
Q8BUH8	Senp7	0.036	0.125	-0.092	0.000	0.105	-0.578	-0.970	-0.310
Q07797	Lgals3bp	0.056	0.168	0.660	0.000	-0.240	1.040	0.809	0.805
Q9QYB1	Clic4	0.007	0.051	0.074	0.000	-0.173	1.240	0.654	0.833
Q9CR16	Ppid	0.050	0.155	0.063	0.000	-0.400	-0.464	-0.946	-0.621
P19096	Fasn	0.558	0.826	0.157	0.000	-0.066	0.282	-0.464	-0.161
Q8BTS4	Nup54	0.174	0.357	-0.078	0.000	0.036	-0.224	-0.263	0.002
P61750	Arf4	0.057	0.168	0.156	-0.200	0.000	1.220	0.315	0.670
P43247	Msh2	0.040	0.135	0.112	0.000	-0.005	-0.303	-0.900	-0.393
Q9CXF4	Tbc1d15	0.442	0.698	0.404	0.000	-0.414	0.186	-0.566	-0.489
P47856	Gfpt1	0.260	0.476	0.041	-0.007	0.000	0.080	-0.490	-0.204
Q640M1	Utp14a	0.054	0.163	0.000	-0.037	0.384	-0.252	-0.903	-0.422
Q9QZM0	Ubqln2	0.002	0.025	0.000	-0.073	0.286	-0.713	-0.834	-0.761
Q60739	Bag1	0.001	0.015	-0.089	0.000	0.076	-1.060	-1.260	-0.917
Q8BH95	Echs1	0.035	0.124	0.000	0.240	-0.097	0.915	0.414	0.524
Q9JLV6	Pnkp	0.033	0.121	0.186	0.000	-0.012	-0.137	-0.517	-0.435
P56391	Cox6b1	0.015	0.076	0.030	0.000	-0.083	0.406	0.174	0.409
P48758	Cbr1	0.045	0.146	0.000	-0.362	0.229	-0.750	-2.180	-1.160
Q60668	Hnrnpd	0.029	0.111	-0.167	0.000	0.014	-0.668	-0.810	-0.301
Q99LF4	Rtcb	0.551	0.820	0.000	0.036	-0.061	0.119	-0.108	0.122
Q91V64	Isoc1	0.027	0.106	0.263	0.000	-0.176	-0.967	-1.210	-2.380
Q9D3E6	Stag1	0.097	0.241	-0.110	0.000	0.040	-0.192	-0.391	-0.105
Q9DBE9	Ftsj3	0.149	0.319	0.213	0.000	-0.414	-0.104	-0.908	-1.010
Q9JLV5	Cul3	0.171	0.353	0.033	-0.575	0.000	-0.300	-1.190	-0.676
Q99N96	Mrpl1	0.091	0.231	0.000	-1.980	0.201	0.820	0.852	1.270
Q8K2T8	Paf1	0.000	0.013	0.000	-0.060	0.046	-0.832	-0.873	-0.672
O09159	Man2b1	0.658	0.934	0.557	-0.062	0.000	0.520	0.127	0.181
Q9Z1X4	Ilf3	0.531	0.798	-0.320	0.120	0.000	-0.211	-0.422	0.045
P42567	Eps15	0.012	0.067	0.268	-0.005	0.000	-0.508	-1.210	-0.973
Q9CPR7	Sike1	0.285	0.509	-0.334	0.039	0.000	-0.127	-0.901	-0.255
Q9D0R2	Tars	0.045	0.146	-0.112	0.000	0.099	-0.225	-0.415	-0.184
Q07076	Anxa7	0.833	1.000	0.000	0.052	-0.034	0.275	-0.212	0.053
Q9QYE6	Golga5	0.161	0.339	0.042	-0.319	0.000	0.172	0.003	0.235
Q61768	Kif5b	0.532	0.799	0.107	0.000	-0.003	0.348	-0.450	-0.300
Q99KE1	Me2	0.952	1.000	0.000	0.140	-0.205	0.190	-0.212	-0.072
Q60931	Vdac3	0.007	0.050	0.000	0.252	-0.058	1.080	0.902	1.570
Q63850	Nup62	0.333	0.571	0.264	-3.320	0.000	0.165	-0.153	0.862
O55098	Stk10	0.435	0.692	-0.223	0.161	0.000	-0.488	-0.706	0.292
Q8BZH4	Pogz	0.153	0.326	-0.120	0.000	0.033	-0.410	-0.449	-0.010
Q9QYJ0	Dnaja2	0.688	0.965	0.097	0.000	-0.099	0.154	-0.236	-0.084
Q8CDN6	Txnl1	0.008	0.051	0.000	0.040	-0.283	-0.969	-1.380	-0.821
Q8R5C5	Actr1b	0.301	0.529	-0.159	0.417	0.000	0.472	0.374	0.126
P09602	Hmgn2	0.380	0.629	-1.030	0.000	0.113	-0.438	-0.284	-3.170
P09055	Itgb1	0.001	0.019	0.000	0.434	0.000	1.880	1.520	1.680
Q924W5	Smc6	0.171	0.353	0.165	0.000	-0.119	-0.122	-0.954	-0.241
O08663	Metap2	0.390	0.640	0.466	-3.020	0.000	-1.320	-1.100	-4.830
P09925	Surf1	0.000	0.013	0.069	0.000	-0.073	0.682	0.590	0.722
O89100	Grap2	0.049	0.154	-0.257	0.110	0.000	-0.559	-2.360	-1.760

P06745	Gpi	0.005	0.039	0.533	-0.070	0.000	-1.010	-1.620	-1.360
Q9EQU5	Set	0.009	0.055	0.000	0.489	-0.112	-0.929	-0.885	-0.687
P58742	Aaas	0.006	0.044	0.000	0.068	-0.024	0.523	0.402	0.731
Q8BMD8	Slc25a24	0.023	0.096	-0.447	0.176	0.000	0.526	0.574	0.757
O70172	Pip4k2a	0.136	0.301	0.051	0.000	-0.088	-0.007	-0.946	-0.646
P62880	Gnb2	0.106	0.254	0.181	-0.405	0.000	0.353	0.177	0.720
P11103	Parp1	0.506	0.770	-0.125	0.040	0.000	-0.190	-0.321	0.120
Q99LC2	Cstf1	0.099	0.242	-0.452	0.024	0.000	-0.702	-0.620	-0.334
P11352	Gpx1	0.131	0.294	0.457	0.000	-0.235	-0.028	-0.804	-0.877
Q04750	Top1	0.149	0.319	0.118	-0.057	0.000	-0.028	-0.407	-0.160
Q8VE47	Uba5	0.001	0.020	0.048	-0.035	0.000	-0.621	-0.940	-0.867
Q8K4G5	Ablim1	0.436	0.693	-0.562	0.000	0.335	-0.503	-0.450	-0.049
P06332	Cd4	0.187	0.377	-0.346	0.000	0.028	-0.404	-0.507	-0.161
Q8BRF7	Scfd1	0.745	1.000	0.000	-0.031	0.146	0.060	-0.193	0.131
P04627	Araf	0.149	0.319	0.195	-0.430	0.000	-2.140	-1.090	-7.290
Q61545	Ewsr1	0.344	0.584	-0.147	0.000	0.141	-0.110	-0.516	0.030
Q9CY64	Blvra	0.001	0.017	0.070	0.000	-0.027	-1.010	-1.480	-1.270
P54116	Stom	0.556	0.825	0.677	0.000	-0.243	0.446	-0.539	-0.245
Q0P678	Zc3h18	0.031	0.114	0.000	-0.085	0.167	-0.421	-1.190	-0.663
P59235	Nup43	0.560	0.829	-0.120	0.383	0.000	-0.494	0.070	0.192
Q3UW53	Fam129a	0.316	0.550	-0.006	0.203	0.000	0.205	-0.653	-0.238
Q02819	Nucb1	0.009	0.056	0.039	0.000	-0.189	0.852	0.418	0.648
P62334	Psmc6	0.204	0.400	-0.038	0.043	0.000	0.062	-0.326	-0.538
P99029	Prdx5	0.996	1.000	0.234	0.000	-0.266	0.051	-0.201	0.121
P56183	Rrp1	0.829	1.000	1.090	0.000	-2.470	0.744	-3.520	0.226
Q9DCR2	Ap3s1	0.247	0.458	0.237	-0.394	0.000	0.805	0.129	0.105
Q9JLZ8	Sigirr	0.827	1.000	-1.170	0.459	0.000	-0.526	-0.206	-0.321
Q9QUR6	Prep	0.027	0.106	0.102	-0.262	0.000	-0.737	-1.200	-0.530
Q9DBP5	Cmpk1	0.018	0.084	0.000	0.122	-0.056	-0.724	-1.950	-1.410
Q5SFM8	Rbm27	0.299	0.528	-0.487	0.001	0.000	0.226	-0.114	0.081
Q7JJ13	Brd2	0.032	0.117	-0.064	0.006	0.000	-0.586	-1.290	-0.544
Q6PHZ2	Camk2d	0.078	0.209	-0.861	0.391	0.000	-1.070	-2.170	-0.983
P29416	Hexa	0.886	1.000	0.576	-0.195	0.000	0.462	-0.293	0.066
O70252	Hmox2	0.188	0.378	0.000	0.189	-0.038	0.252	0.067	0.428
Q9DB73	Cyb5r1	0.000	0.008	0.000	0.094	-0.277	2.440	2.150	2.440
Q9DBH5	Lman2	0.002	0.025	0.000	0.055	-0.225	0.807	0.612	0.822
P62242	Rps8	0.850	1.000	0.000	0.181	-0.355	0.070	-0.089	-0.056
Q6P069	Sri	0.434	0.691	-0.202	0.027	0.000	-0.084	-0.301	-0.063
Q9CZX8	Rps19	0.508	0.772	0.243	-0.007	0.000	0.331	-0.433	-0.184
Q07417	Acads	0.730	1.000	0.089	0.000	-0.125	0.435	-0.537	-0.263
Q8R1B4	Eif3c	0.348	0.589	0.218	0.000	-0.044	0.214	-0.467	-0.267
O88379	Baz1a	0.138	0.303	0.035	0.000	-0.139	-0.281	-0.452	-0.062
P14429	H2-Q7	0.206	0.403	-0.383	0.114	0.000	-0.472	-0.369	-0.198
P70452	Stx4	0.379	0.628	0.000	0.361	-1.230	0.582	0.088	-0.010
P12787	Cox5a	0.002	0.028	0.000	0.139	-0.028	0.629	0.454	0.629
Q8BKE6	Cyp20a1	0.000	0.014	0.000	0.088	-0.320	1.730	1.440	1.780
P04104	Krt1	0.277	0.498	-0.073	0.000	0.585	-0.156	1.390	1.350
P17439	Gba	0.007	0.049	0.708	0.000	-0.006	1.550	1.370	1.490
Q9D0M3	Cyc1	0.192	0.384	0.243	0.000	-0.038	0.016	0.587	0.597
Q7TSI3	Ppp6r1	0.005	0.040	0.000	-0.040	0.250	-0.738	-1.240	-0.832
Q80X85	Mrps7	0.051	0.158	0.000	0.066	-0.627	0.765	0.358	0.418
Q99ME9	Gtpbp4	0.155	0.329	0.013	-0.133	0.000	-0.088	-0.821	-0.361
Q00417	Tcf7	0.842	1.000	-1.160	0.000	0.065	-0.570	-0.372	0.138
Q922Q8	Lrrc59	0.002	0.028	0.000	0.070	-0.353	0.875	0.825	0.768
P24547	Impdh2	0.232	0.437	0.041	0.000	-0.289	-0.073	-0.748	-0.361
P70670	Naca	0.788	1.000	0.000	0.099	-0.468	-0.113	-0.429	-0.013
Q61152	Ptpn18	0.001	0.020	-0.173	0.000	0.344	-1.430	-1.960	-1.780
Q8BGQ7	Aars	0.540	0.807	0.245	0.000	-0.060	0.323	-0.318	-0.276
P35486	Pdha1	0.264	0.482	0.044	0.000	-0.235	0.792	0.073	-0.010

Q9CYZ2	Tpd52l2	0.037	0.129	0.129	-0.440	0.000	-0.566	-0.943	-1.290
P48024	Eif1	0.101	0.247	0.057	0.000	-0.056	-0.341	-0.676	-0.087
Q9CXU9	Eif1b	0.101	0.247	0.057	0.000	-0.056	-0.341	-0.676	-0.087
P70168	Kpnb1	0.204	0.400	-0.040	0.088	0.000	-0.081	-0.545	-0.064
P80318	Cct3	0.041	0.138	0.007	0.000	-0.029	-0.227	-0.751	-0.412
O88665	Brd7	0.381	0.630	0.000	-0.139	0.144	-0.197	-0.413	0.108
Q1HFZ0	Nsun2	0.102	0.247	0.289	0.000	-0.086	-0.029	-1.110	-0.849
P70333	Hnrnph2	0.656	0.932	-0.681	0.027	0.000	-0.074	-0.027	-0.211
Q80ZW2	Them6	0.880	1.000	0.000	2.290	-4.050	-2.070	0.845	0.459
Q9D1D4	Tmed10	0.017	0.081	0.000	0.326	-0.089	1.230	0.979	2.020
Q61166	Mapre1	0.016	0.079	0.211	-0.152	0.000	-0.500	-0.907	-1.160
Q6P542	Abcf1	0.342	0.582	0.361	-0.057	0.000	0.327	-0.666	-0.406
Q9DCE5	Pak1ip1	0.033	0.120	0.064	-0.123	0.000	-0.401	-0.996	-0.500
Q9JMH9	Myo18a	0.465	0.723	-0.001	0.000	0.173	0.109	-0.451	0.058
O88456	Capns1	0.707	0.983	-0.351	0.263	0.000	-0.211	-0.070	-0.033
Q8BJ71	Nup93	0.605	0.878	0.000	0.099	-0.117	0.056	-0.115	0.244
O88520	Shoc2	0.850	1.000	0.000	-0.539	0.000	-0.051	-0.911	0.193
P53810	Pitpna	0.002	0.023	0.000	0.117	-0.135	-1.010	-1.530	-1.350
P61804	Dad1	0.002	0.025	0.054	0.000	-0.232	1.770	1.190	1.270
P39749	Fen1	0.169	0.351	0.002	-0.215	0.000	-0.243	-0.778	-0.197
Q9WU81	Slc37a2	0.276	0.497	0.431	0.000	-0.628	0.442	0.392	0.173
P14148	Rpl7	0.006	0.044	0.207	-0.004	0.000	0.767	0.494	0.739
P61620	Sec61a1	0.001	0.019	0.000	0.072	-0.131	1.560	1.060	1.370
Q61171	Prdx2	0.040	0.134	0.089	0.000	-0.208	-0.353	-0.755	-0.402
Q9R1P3	Psmb2	0.006	0.044	0.222	0.000	-0.028	-0.810	-1.210	-1.570
Q8C7X2	Emc1	0.001	0.015	0.000	0.211	-0.285	1.450	1.340	1.440
Q9CQB5	Cisd2	0.179	0.365	0.000	0.161	-0.086	0.224	0.101	0.145
Q8CHP5	Pym1	0.018	0.083	-0.344	0.000	0.040	-0.801	-1.330	-0.753
Q8K3A0	Hscb	0.378	0.627	0.020	0.000	-7.410	-0.071	0.082	-0.054
Q3V1T4	P3h1	0.001	0.022	0.735	-0.994	0.000	4.580	3.830	4.310
Q8R3N6	Thoc1	0.334	0.571	0.080	0.000	-0.098	-0.259	-0.213	0.073
Q64737	Gart	0.258	0.473	0.691	0.000	-0.051	0.448	-0.834	-1.050
Q920Q6	Msi2	0.276	0.497	0.000	0.706	-0.324	1.510	1.050	-0.068
Q9Z1Q5	Clic1	0.005	0.039	-0.013	0.084	0.000	-0.653	-1.240	-1.010
Q9D6K5	Synj2bp	0.056	0.167	0.000	0.262	-0.061	0.573	0.279	0.805
O54786	Dffa	0.079	0.210	0.000	-0.630	0.300	-0.522	-1.400	-1.040
Q9EPL9	Acox3	0.674	0.951	0.021	0.000	-0.636	0.263	-0.494	-0.944
P97384	Anxa11	0.008	0.053	0.041	0.000	-0.141	-0.485	-0.611	-0.358
O35381	Anp32a	0.000	0.012	-0.159	0.084	0.000	-1.290	-1.590	-1.380
P07901	Hsp90aa1	0.021	0.090	0.000	0.010	-0.335	-0.557	-1.180	-1.010
Q64518	Atp2a3	0.176	0.360	0.078	0.000	-0.382	-0.145	-0.536	-0.666
Q9QZD8	Slc25a10	0.003	0.030	0.000	0.214	-0.415	1.190	1.260	1.510
P52633	Stat6	0.079	0.210	0.000	-0.088	0.044	-0.100	-0.740	-0.588
Q02111	Prkcq	0.722	1.000	0.000	1.430	-0.213	-0.229	0.752	0.016
P62305	Snrpe	0.123	0.283	0.000	0.203	-0.302	-0.339	-0.394	-1.200
Q80YV2	Zc3hc1	0.030	0.113	-0.583	0.055	0.000	-0.937	-0.866	-1.450
Q5SUR0	Pfas	0.006	0.045	-0.054	0.678	0.000	-1.340	-1.880	-1.200
Q80X50	Ubap2l	0.003	0.032	0.000	-0.103	0.353	1.140	0.971	1.270
Q9D8C4	Ifi35	0.019	0.085	-0.500	0.000	0.050	-1.010	-1.030	-1.700
Q7TMY8	Huwe1	0.222	0.423	0.263	-0.109	0.000	0.159	-0.989	-0.543
Q9JII6	Akr1a1	0.500	0.765	0.262	-0.011	0.000	0.150	-0.272	0.031
P25425	Pou2f1	0.006	0.047	-0.147	0.120	0.000	-0.422	-0.622	-0.496
O89017	Lgmn	0.545	0.813	1.600	-0.051	0.000	0.284	-0.932	0.738
Q9CWW6	Pin4	0.376	0.624	0.000	0.180	-0.252	-0.110	-0.344	-0.071
Q61733	Mrps31	0.132	0.296	0.000	0.114	-0.108	0.841	-0.005	0.687
Q8BHZ0	Fam49a	0.018	0.083	0.100	0.000	-0.048	-0.759	-2.060	-1.560
Q61074	Ppm1g	0.015	0.076	0.076	0.000	0.000	-0.823	-1.880	-1.110
Q8R2U0	Seh1l	0.165	0.347	-0.177	0.102	0.000	-0.189	-0.511	-0.117
Q64378	Fkbp5	0.009	0.056	-0.004	0.047	0.000	-0.637	-1.400	-1.220

Q91YE7	Rbm5	0.008	0.052	0.000	-0.307	0.016	-0.952	-1.520	-0.983
Q8BQ30	Ppp1r18	0.409	0.660	-0.391	0.000	0.142	-0.536	-0.383	0.028
Q8CBW3	Abi1	0.213	0.409	-0.378	0.171	0.000	0.201	0.069	0.305
P25976	Ubtf	0.014	0.072	-0.184	0.000	0.021	-0.544	-0.926	-0.527
Q9CQH3	Ndufb5	0.003	0.032	0.000	0.202	-0.151	0.682	0.684	0.812
Q8BH74	Nup107	0.749	1.000	-0.109	0.284	0.000	-0.001	-0.230	0.225
P97429	Anxa4	0.589	0.861	0.000	0.197	-0.094	0.014	-0.212	0.083
P03921	Mtnd5	0.051	0.156	0.000	0.416	-0.059	0.695	0.639	1.400
Q9CY50	Ssr1	0.002	0.025	0.000	0.307	-0.320	1.390	1.330	1.580
P20060	Hexb	0.512	0.776	0.530	-0.073	0.000	0.432	-0.428	-0.245
Q922Y1	Ubxn1	0.019	0.087	0.000	-0.150	0.077	-0.496	-0.940	-0.493
P26350	Ptma	0.061	0.176	0.136	0.000	-0.615	-0.753	-2.920	-2.010
Q8C3J5	Dock2	0.006	0.047	0.009	0.000	-0.011	-0.628	-1.100	-0.665
O35593	Psmd14	0.036	0.125	0.000	-0.104	0.054	-0.306	-0.715	-0.334
Q64213	Sf1	0.008	0.054	0.000	0.089	-0.065	-0.205	-0.298	-0.238
Q8C9V1	Tbc1d10c	0.042	0.139	0.000	-0.523	0.236	-0.798	-1.680	-0.940
Q8K2I4	Manba	0.513	0.777	0.000	0.090	-0.405	0.151	-0.227	0.198
Q7TNV0	Dek	0.006	0.047	0.137	-0.007	0.000	-0.513	-1.050	-0.888
P62748	Hpcal1	0.005	0.041	0.000	0.247	-0.109	-0.560	-0.589	-0.515
P62918	Rpl8	0.638	0.914	0.175	-0.165	0.000	0.451	-0.129	-0.005
Q9Z1J3	Nfs1	0.866	1.000	0.239	0.000	-0.042	0.878	-0.349	-0.124
P17095	Hmga1	0.277	0.498	0.000	-0.453	0.203	-0.194	-1.160	-0.274
Q8VHR5	Gatad2b	0.198	0.392	-0.373	0.000	0.093	-0.487	-0.543	-0.135
Q8BHG1	Nrdc	0.000	0.008	-0.090	0.116	0.000	-1.730	-1.490	-1.520
P83741	Wnk1	0.046	0.149	-0.144	0.000	0.437	-0.714	-1.090	-0.318
O08582	Gtpbp1	0.039	0.133	0.342	-0.062	0.000	-0.272	-1.110	-0.866
Q9D0E1	Hnrnpm	0.116	0.271	-0.089	0.068	0.000	-0.065	-0.477	-0.243
Q60631	Grb2	0.005	0.041	-0.091	0.403	0.000	-1.080	-1.510	-0.944
Q3UDW8	Hgsnat	0.535	0.803	0.177	0.000	-0.116	0.499	-0.142	0.120
P58059	Mrps21	0.054	0.163	0.000	0.089	-0.066	0.647	0.140	0.589
P14094	Atp1b1	0.004	0.034	-0.400	0.000	0.313	2.120	1.460	2.270
P53395	Dbt	0.046	0.149	0.000	0.286	-0.049	0.968	0.326	0.859
Q9ESW4	Agk	0.183	0.372	0.000	-0.572	0.077	0.527	-0.141	0.703
Q8BKS9	Pum3	0.196	0.389	0.136	0.000	-0.234	-0.103	-1.360	-0.447
O89110	Casp8	0.012	0.067	0.166	-0.032	0.000	-1.050	-1.120	-2.060
P42230	Stat5a	0.015	0.076	-0.331	0.072	0.000	-0.731	-1.550	-1.370
Q9Z2L7	Crlf3	0.015	0.076	0.078	-0.288	0.000	-0.879	-2.030	-1.700
Q9CZ83	Mrpl55	0.003	0.031	0.173	0.000	-0.267	0.903	0.758	0.928
O70492	Snx3	0.413	0.665	0.054	0.000	-0.003	0.688	-0.177	0.226
Q8BXA5	Clptm1l	0.009	0.056	0.000	0.069	-0.288	0.729	0.507	0.942
Q8BG48	Stk17b	0.005	0.039	0.000	-0.182	0.023	-1.390	-1.950	-1.130
P46664	Adss	0.051	0.157	0.264	-0.674	0.000	-0.768	-1.940	-1.330
Q9DCT2	Ndufs3	0.021	0.090	-0.343	0.080	0.000	0.597	0.506	1.070
P62082	Rps7	0.231	0.435	0.000	0.021	-0.201	0.193	-0.095	0.370
Q3UM45	Ppp1r7	0.011	0.061	-0.459	0.000	0.147	-1.720	-1.920	-1.040
Q62465	Vat1	0.029	0.110	0.420	0.000	-0.104	1.170	0.561	1.040
Q61107	Gbp4	0.002	0.026	0.000	-0.009	0.293	-1.020	-1.590	-1.530
P43275	Hist1h1a	0.473	0.733	0.000	-0.398	0.015	-0.138	-0.623	-0.127
Q91VH2	Snx9	0.007	0.051	0.000	0.205	-0.383	1.650	1.140	0.994
Q9CRD2	Emc2	0.000	0.012	0.000	0.161	-0.136	1.450	1.330	1.590
Q99JX7	Nxf1	0.309	0.542	0.393	-0.510	0.000	-0.281	-0.727	-0.190
P63094	Gnas	0.019	0.087	-0.209	0.111	0.000	0.694	0.307	0.605
Q11011	Npepps	0.059	0.174	0.306	0.000	-0.087	-0.142	-0.600	-0.447
P02535	Krt10	0.910	1.000	-0.437	0.000	0.988	-0.544	0.928	0.386
P97314	Csrp2	0.001	0.019	-1.840	0.000	0.161	5.250	4.780	5.010
P18760	Cfl1	0.003	0.033	-0.271	0.034	0.000	-0.898	-1.220	-0.868
P62962	Pfn1	0.000	0.012	0.000	0.175	-0.052	-1.240	-1.510	-1.260
P49312	Hnrnpa1	0.225	0.427	-0.046	0.209	0.000	-0.160	-0.235	0.055
P97333	Nrp1	0.223	0.425	-0.500	0.000	0.295	-0.584	-0.652	-0.167

Q8VDP6	Cdipt	0.044	0.142	-0.068	0.082	0.000	0.492	0.133	0.466
P51660	Hsd17b4	0.034	0.122	0.027	0.000	-0.287	1.110	0.293	0.795
Q9D8N0	Eef1g	0.431	0.687	0.665	-0.080	0.000	0.863	-1.110	-0.938
Q05920	Pc	0.015	0.076	0.211	0.000	-0.913	2.850	1.420	1.760
P10649	Gstm1	0.260	0.476	1.250	0.000	-0.306	0.019	-0.763	-0.382
P61226	Rap2b	0.010	0.059	0.419	0.000	-0.170	1.050	0.866	0.897
Q3TDN2	Faf2	0.182	0.369	-0.615	0.705	0.000	0.072	1.380	2.500
Q8K358	Pigu	0.174	0.357	0.343	0.000	-1.340	0.317	1.360	5.340
P49442	Inpp1	0.013	0.070	-0.686	0.009	0.000	-2.310	-3.380	-1.760
P61957	Sumo2	0.255	0.469	0.000	-0.147	0.071	-0.215	-3.690	-0.576
Q8VCW8	Acsf2	0.374	0.623	0.347	-0.054	0.000	0.597	-1.130	-0.804
Q8K1J6	Trnt1	0.089	0.227	-0.056	0.000	0.076	-0.250	-0.666	-0.153
Q02248	Ctnnb1	0.001	0.018	0.000	1.470	-0.278	6.150	5.320	5.830
Q80VP0	Tecpr1	0.211	0.407	-0.458	0.000	0.797	-0.779	-0.878	-0.031
Q9WUN2	Tbk1	0.159	0.336	0.000	-0.120	0.216	-0.002	-1.180	-0.558
Q9CPR5	Mrpl15	0.032	0.119	0.144	-1.190	0.000	1.030	0.900	1.190
Q8CH25	Sltm	0.673	0.950	-0.415	0.000	0.073	-0.058	-0.117	0.051
Q8CBA2	Slfn5	0.102	0.247	0.000	-0.213	0.161	-0.133	-0.594	-0.422
Q9DCW4	Etfb	0.322	0.558	0.252	0.000	-0.128	0.867	-0.052	0.291
Q9DBR0	Akap8	0.021	0.092	0.000	0.298	-0.040	-0.520	-0.856	-0.359
Q3UQU0	Brd9	0.145	0.314	-0.026	0.000	0.187	-0.021	-1.600	-0.720
Q8VD26	Tmem143	0.001	0.022	-0.252	0.062	0.000	0.775	0.891	1.040
O35638	Stag2	0.144	0.311	0.000	0.186	-0.178	-0.177	-0.493	-1.410
Q8C1D8	Iws1	0.115	0.270	0.000	-0.155	0.078	-0.329	-0.906	-0.206
Q9Z315	Sart1	0.004	0.038	-0.166	0.000	0.034	-0.707	-1.120	-0.776
Q9CQ54	Ndufc2	0.001	0.022	0.044	0.000	-0.180	0.889	0.645	0.923
P17918	Pcna	0.142	0.309	0.516	0.000	-0.203	-0.004	-0.794	-0.676
Q9CX86	Hnrnpa0	0.237	0.445	-0.227	0.000	0.302	0.083	-0.505	-0.595
Q8VCY6	Utp6	0.039	0.133	0.158	-0.187	0.000	-0.292	-0.675	-0.422
P99024	Tubb5	0.970	1.000	0.000	-0.069	0.025	0.542	-0.296	-0.257
P62317	Snrpd2	0.037	0.129	-0.005	0.066	0.000	-0.224	-0.407	-0.116
Q9CQ75	Ndufa2	0.016	0.078	-0.063	0.189	0.000	0.742	0.367	0.640
P45376	Akr1b1	0.002	0.023	-0.133	0.414	0.000	-1.310	-1.620	-1.300
Q00899	Yy1	0.078	0.208	0.000	-0.096	0.192	-0.240	-1.020	-0.413
P23780	Glb1	0.709	0.985	0.541	0.000	-0.380	0.396	0.028	0.086
Q8BKJ9	Sirt7	0.048	0.152	0.000	-0.263	0.089	-0.314	-0.839	-0.582
Q3UUV5	Skap1	0.002	0.023	-0.314	0.000	0.236	-1.930	-2.300	-1.630
Q91VM5	Rbmxl1	0.172	0.354	-0.370	0.000	0.091	-0.513	-0.512	-0.165
P09671	Sod2	0.023	0.096	-0.061	0.348	0.000	0.715	0.476	0.682
Q9D958	Spcs1	0.952	1.000	0.410	-1.850	0.000	0.580	-2.270	0.466
O88844	Idh1	0.617	0.891	0.610	0.000	-0.573	0.268	0.140	0.187
Q9DB42	Znf593	0.300	0.529	-0.360	0.000	0.352	-0.645	-0.477	0.060
P04441	Cd74	0.860	1.000	0.000	0.083	-0.265	-0.059	-0.135	-0.049
O55029	Copb2	0.051	0.158	0.023	0.000	-0.067	-0.281	-1.100	-0.638
P52431	Pold1	0.005	0.043	-0.206	0.235	0.000	-0.797	-1.220	-0.903
Q9Z1D1	Eif3g	0.484	0.747	0.391	0.000	-0.762	0.185	-1.290	-0.524
P27048	Snrpb	0.016	0.079	0.000	0.086	-0.061	-0.366	-0.857	-0.543
Q8K4L3	Svil	0.677	0.953	0.060	-0.124	0.000	0.329	-0.177	-0.004
Q99LP6	Grpel1	0.003	0.030	0.000	0.185	-0.177	1.010	0.736	0.977
Q9CR62	Slc25a11	0.026	0.105	0.013	0.000	-0.081	0.658	0.233	0.369
O35309	Nmi	0.000	0.008	0.000	-0.038	0.026	-0.966	-1.090	-0.910
Q8BKC5	Ipo5	0.805	1.000	0.128	0.000	-0.716	0.189	-0.657	-0.408
Q8BH97	Rcn3	0.000	0.010	0.000	0.300	-1.020	5.700	5.470	5.630
P42932	Cct8	0.038	0.129	0.019	0.000	-0.027	-0.248	-0.835	-0.494
Q8BHJ5	Tbl1xr1	0.162	0.341	-0.117	0.000	0.120	-0.368	-2.330	-0.539
Q9WUK4	Rfc2	0.057	0.169	0.113	0.000	-0.228	-0.245	-0.935	-0.770
Q8BH24	Tm9sf4	0.003	0.032	0.024	0.000	-0.422	0.875	0.752	0.962
Q921G8	Tubgcp2	0.065	0.184	0.000	-0.034	0.085	-0.377	-0.541	-0.083
Q3UPL0	Sec31a	0.955	1.000	0.030	-0.312	0.000	0.370	-0.502	-0.200

P45952	Acadm	0.441	0.697	0.020	0.000	-0.423	0.852	-0.186	-0.129
Q9Z2E1	Mbd2	0.061	0.176	-0.257	0.000	0.089	-0.475	-0.467	-0.235
Q6ZQF7	Jade2	0.007	0.048	-0.162	0.087	0.000	-0.756	-0.903	-0.512
Q9ERN0	Scamp2	0.659	0.935	0.258	0.000	-0.346	0.217	-0.347	-0.321
O35900	Lsm2	0.000	0.011	-0.100	0.000	0.134	-1.180	-1.020	-1.150
Q9CX30	Yif1b	0.001	0.018	-0.142	0.000	0.081	1.740	1.320	1.910
P51912	Slc1a5	0.002	0.025	0.161	0.000	-0.159	0.905	0.762	1.060
Q80ZS3	Mrps26	0.050	0.155	-0.354	0.562	0.000	0.950	0.666	0.952
Q99LH2	Ptdss1	0.004	0.038	0.073	0.000	-0.255	0.781	1.180	0.770
Q01965	Ly9	0.539	0.807	0.248	-0.106	0.000	-0.213	-0.306	0.254
P23198	Cbx3	0.053	0.162	-0.100	0.000	0.464	-0.392	-0.638	-0.271
O09061	Psmb1	0.002	0.022	0.000	0.060	-0.048	-0.871	-1.280	-0.911
P63028	Tpt1	0.111	0.264	-0.589	1.150	0.000	-2.170	-1.060	-0.523
Q9Z2Q5	Mrpl40	0.003	0.031	0.154	-0.056	0.000	0.713	0.525	0.546
Q9ES46	Parvb	0.600	0.873	0.000	0.158	-1.990	-0.638	-0.108	-3.010
E9PVA8	Gcn1	0.557	0.825	0.132	0.000	-0.329	0.500	-0.383	0.255
O88291	Znf326	0.531	0.798	-0.617	0.000	0.031	-0.141	-0.078	0.089
E9Q394	Akap13	0.012	0.068	-0.244	0.000	0.099	-0.813	-1.320	-0.739
Q8C2K5	Rasal3	0.018	0.083	-0.082	0.141	0.000	-0.512	-1.320	-0.924
P05555	Itgam	0.903	1.000	1.640	-0.097	0.000	1.180	0.078	0.022
P23475	Xrcc6	0.009	0.056	0.040	0.000	-0.446	-0.978	-1.630	-1.300
Q04447	Ckb	0.070	0.192	0.000	0.157	-0.135	-0.164	-0.636	-0.365
Q924T2	Mrps2	0.014	0.073	0.000	0.348	-0.330	0.736	1.060	0.937
Q9Z1M8	Ik	0.021	0.092	0.000	0.281	-0.201	-0.702	-0.501	-0.461
D3YXK2	Safb	0.010	0.060	-0.321	0.077	0.000	-0.723	-0.601	-0.827
Q9CQA3	Sdhb	0.005	0.042	0.000	0.126	-0.143	0.648	0.433	0.552
O70251	Eef1b	0.042	0.140	0.083	-0.155	0.000	-0.239	-0.809	-0.633
Q6Q899	Ddx58	0.144	0.311	0.000	0.060	-0.766	-0.397	-1.090	-1.250
Q9CQ71	Rpa3	0.181	0.368	-0.082	0.000	0.202	-0.347	-0.889	-3.700
P21447	Abcb1a	0.208	0.404	-0.389	0.059	0.000	-0.616	-0.615	-0.099
Q99JI4	Psmd6	0.028	0.107	0.000	0.155	-0.067	-0.324	-0.253	-0.600
Q9DAS9	Gng12	0.017	0.080	1.250	-0.499	0.000	2.910	2.090	2.560
Q8CH02	Sugp1	0.095	0.237	-0.273	0.000	0.142	-0.752	-0.679	-0.159
O55128	Sap18	0.043	0.142	-0.163	0.000	0.168	-0.507	-2.010	-1.380
Q8CG76	Akr7a2	0.383	0.632	-0.121	0.000	0.029	0.397	-0.088	0.045
Q9QY24	Zbp1	0.179	0.364	-0.179	0.000	0.456	-0.213	-0.556	-0.097
Q8BJW6	Eif2a	0.743	1.000	0.280	-0.084	0.000	0.391	-0.452	-0.025
Q8C2Q3	Rbm14	0.597	0.870	-0.370	0.000	0.306	-0.338	-0.331	0.164
Q9D3P8	Plgrkt	0.012	0.069	-0.004	0.232	0.000	0.479	0.379	0.528
Q3UJD6	Usp19	0.246	0.457	0.704	-0.028	0.000	0.999	0.247	0.759
Q9ER69	Wtap	0.018	0.084	0.000	-0.142	0.111	-0.903	-1.710	-0.814
Q9D8B3	Chmp4b	0.033	0.119	0.000	-0.068	0.070	-0.181	-0.595	-0.445
Q9WVJ2	Psmd13	0.040	0.134	0.000	-0.037	0.010	-0.179	-0.699	-0.638
Q9D358	Acp1	0.010	0.060	0.000	0.034	-0.377	-0.763	-1.300	-1.250
P11031	Sub1	0.128	0.291	0.485	0.000	-0.229	-0.017	-1.030	-0.866
Q6ZWU9	Rps27	0.484	0.747	0.000	0.208	-0.051	0.074	-0.521	0.101
Q64282	Ifit1	0.387	0.636	0.484	-0.260	0.000	0.741	-0.075	0.507
Q9JK48	Sh3glb1	0.086	0.222	0.000	0.081	-0.382	-0.354	-0.952	-0.542
Q8BY89	Slc44a2	0.203	0.400	0.143	-0.178	0.000	0.730	-0.130	0.759
P98083	Shc1	0.721	1.000	0.042	-0.011	0.000	1.180	-0.727	0.209
Q9EQ28	Pold3	0.000	0.013	0.036	0.000	-0.085	-0.691	-0.831	-0.668
Q8BTI8	Srrm2	0.272	0.492	-0.683	0.000	0.080	-0.469	-0.866	-0.362
Q9CPW4	Arpc5	0.450	0.708	0.000	-0.098	0.034	0.163	-0.086	0.066
Q9WUU7	Ctsz	0.118	0.274	0.656	-0.102	0.000	0.028	-1.080	-0.862
Q922Q1	Mosc 2	0.217	0.416	0.000	0.380	-0.531	0.398	0.188	0.486
Q3THS6	Mat2a	0.000	0.013	0.099	-0.109	0.000	-0.881	-1.060	-0.919
Q6NVF9	Cpsf6	0.003	0.032	0.000	-0.136	0.019	-0.650	-0.911	-0.604
P05202	Got2	0.002	0.028	0.000	0.188	-0.073	0.799	0.617	0.665
P62874	Gnb1	0.000	0.013	0.000	0.012	-0.274	1.780	1.420	1.780

O08756	Hsd17b10	0.087	0.224	-0.012	0.447	0.000	0.862	0.393	0.563
Q9JL35	Hmgn5	0.058	0.171	-0.153	0.000	0.090	-0.490	-0.558	-0.153
Q05816	Fabp5	0.681	0.957	0.000	0.842	-2.280	0.637	-5.690	0.568
Q9QYB5	Add3	0.122	0.281	0.000	-0.422	0.087	-0.199	-1.070	-0.858
Q64133	Maoa	0.000	0.011	0.151	0.000	-0.761	3.640	3.580	3.770
P22315	Fech	0.179	0.364	0.378	-1.300	0.000	1.010	0.491	0.277
Q80V26	Impad1	0.000	0.014	0.210	-0.225	0.000	1.400	1.310	1.370
Q9DAA6	Exosc1	0.980	1.000	0.000	1.410	-0.356	-0.065	0.682	0.391
Q921K9	Bcl7b	0.026	0.105	-0.081	0.372	0.000	-0.672	-1.020	-0.387
Q99PV8	Bcl11b	0.314	0.547	-0.287	0.000	0.223	-0.356	-0.661	0.065
Q9CPQ8	Atp5l	0.001	0.022	-0.234	0.085	0.000	0.683	0.763	0.798
P24369	Ppib	0.003	0.033	0.000	0.096	-0.188	0.909	0.584	0.780
Q99LE6	Abcf2	0.419	0.673	0.556	-0.007	0.000	0.704	0.144	0.369
Q8BHB4	Wdr3	0.325	0.561	-0.953	0.000	0.292	-0.509	-0.492	-1.080
P21956	Mfge8	0.010	0.061	0.000	-0.252	0.038	1.480	0.660	1.380
O55126	Gbas	0.027	0.106	1.180	-0.355	0.000	1.730	1.880	2.050
O35368	Ifi203	0.019	0.087	-0.210	0.033	0.000	-0.572	-1.050	-1.390
Q8CH18	Ccar1	0.158	0.335	-0.956	0.094	0.000	-0.824	-1.620	-0.710
Q3UMR5	Mcu	0.004	0.038	0.000	0.207	-0.231	0.923	0.701	0.918
P84099	Rpl19	0.926	1.000	0.090	0.000	-0.109	0.249	-0.392	0.186
Q9R0H0	Acox1	0.000	0.012	0.521	0.000	-0.037	2.920	2.570	2.770
Q9D024	Ccdc47	0.003	0.031	0.000	0.288	-0.108	1.170	0.992	1.430
Q9WUR2	Eci2	0.392	0.641	-0.002	0.400	0.000	0.637	0.088	0.274
Q60766	Irgm1	0.018	0.083	0.051	0.000	-0.187	0.495	0.228	0.373
P62983	Rps27a	0.185	0.374	0.000	-0.150	0.017	-0.164	-0.623	-0.142
Q6ZWV3	Rpl10	0.061	0.176	0.051	-0.075	0.000	0.587	0.110	0.392
B9EJ86	Osbpl8	0.137	0.302	0.203	0.000	-0.375	0.463	0.106	0.376
Q9Z2X1	Hnrnpf	0.015	0.076	-0.128	0.274	0.000	-0.417	-0.823	-0.664
P35700	Prdx1	0.130	0.293	0.028	0.000	-0.030	0.421	-0.006	0.325
P16330	Cnp	0.135	0.298	0.000	0.133	-0.102	-0.008	-0.671	-0.448
Q61735	Cd47	0.827	1.000	0.608	0.000	-0.357	-0.209	-0.255	1.080
Q05CL8	Larp7	0.002	0.029	0.038	-0.083	0.000	-0.704	-1.150	-0.895
Q61578	Fdxr	0.099	0.243	0.000	0.172	-0.061	-0.043	-0.801	-0.540
Q61136	Prpf4b	0.129	0.291	0.000	0.121	-0.928	-0.820	-0.932	-0.971
O88559	Men1	0.002	0.025	-0.161	0.000	0.096	-0.644	-0.763	-0.597
Q8K363	Ddx18	0.197	0.391	0.093	-0.221	0.000	0.055	-0.960	-0.698
Q8JZQ2	Afg3l2	0.015	0.076	0.000	0.090	-0.109	0.828	0.358	0.590
P62313	Lsm6	0.017	0.081	0.000	-0.578	0.234	-1.060	-1.350	-1.030
P62259	Ywhae	0.027	0.105	0.000	0.068	-0.183	-0.420	-1.160	-0.884
Q61753	Phgdh	0.305	0.536	0.093	0.000	-0.202	0.150	-0.694	-0.523
Q505F5	Lrrc47	0.193	0.385	0.492	0.000	-0.011	0.172	-0.814	-0.432
P07742	Rrm1	0.156	0.330	1.310	-0.668	0.000	-0.320	-1.880	-1.010
Q9CSN1	Snw1	0.101	0.247	0.000	-0.182	0.300	-0.364	-0.567	-0.141
Q9D880	Timm50	0.034	0.123	0.000	0.165	-0.056	0.558	0.221	0.486
Q6ZQ88	Kdm1a	0.170	0.353	0.000	0.467	-0.476	-0.467	-0.683	-0.320
Q08642	Padi2	0.002	0.027	0.000	-0.067	0.169	-1.410	-1.390	-0.906
Q7TSG2	Ctdp1	0.053	0.162	-0.333	0.000	0.669	-0.845	-1.500	-0.585
P97496	Smarcc1	0.887	1.000	-0.284	1.510	0.000	0.430	1.030	-0.553
Q9CPR4	Rpl17	0.058	0.171	0.038	0.000	-0.028	0.436	0.083	0.437
Q6A068	Cdc5l	0.072	0.196	-0.551	0.000	0.012	-0.825	-0.927	-0.473
P53986	Slc16a1	0.170	0.352	0.555	0.000	-0.036	1.090	0.354	0.539
Q60902	Eps15l1	0.605	0.878	0.310	-0.100	0.000	-0.085	-0.310	0.258
Q9QXT0	Cnpy2	0.001	0.019	0.000	0.051	-0.133	1.060	0.742	0.879
Q6A4J8	Usp7	0.012	0.067	0.000	-0.009	0.033	-0.659	-1.400	-0.850
Q9CYL5	Glipr2	0.076	0.205	0.000	-0.110	0.013	0.537	0.043	0.419
Q9CQW1	Ykt6	0.330	0.568	-0.082	0.999	0.000	0.328	0.902	1.060
Q61595	Ktn1	0.022	0.095	0.000	-0.106	0.163	0.570	0.650	1.220
Q6PF93	Pik3c3	0.004	0.034	0.000	0.082	-0.066	-0.656	-0.946	-1.160
P70296	Pebp1	0.000	0.012	-0.033	0.000	0.005	-1.230	-1.610	-1.490

P28271	Aco1	0.002	0.024	0.000	-0.026	0.042	-0.794	-1.280	-1.150
Q9QXB9	Drg2	0.746	1.000	0.545	-0.088	0.000	0.489	-0.337	-0.021
P29341	Pabpc1	0.216	0.413	0.000	0.523	-0.083	-0.141	-0.164	-0.096
Q64735	Cr1l	0.871	1.000	0.107	0.000	-0.145	0.063	-0.267	0.095
Q8BGU5	Ccny	0.233	0.438	-0.007	0.027	0.000	0.181	-0.036	0.360
Q8K2C9	Hacd3	0.081	0.213	-0.211	0.067	0.000	0.124	0.145	0.335
O70439	Stx7	0.141	0.308	0.241	0.000	-0.122	-0.013	-0.452	-0.348
Q8K2C7	Os9	0.015	0.075	-0.244	0.661	0.000	1.410	1.240	1.200
P62482	Kcnab2	0.556	0.825	0.000	0.764	-1.840	-0.436	-2.640	-0.126
Q501J6	Ddx17	0.007	0.048	-0.046	0.116	0.000	-0.532	-0.750	-0.397
P20065	Tmsb4x	0.010	0.058	0.187	0.000	-0.161	-1.300	-2.470	-1.480
P23708	Nfya	0.195	0.387	0.098	0.000	-0.157	-0.432	-0.614	-3.200
Q922Q9	Chid1	0.852	1.000	0.030	0.000	-0.341	-0.419	0.214	-0.239
P70399	Tp53bp1	0.062	0.178	0.267	0.000	-0.013	-0.418	-1.440	-0.510
Q3TIR3	Ric8a	0.860	1.000	0.000	-0.572	0.168	0.033	-1.140	0.413
Q5SSL4	Abr	0.067	0.188	0.000	-0.140	0.026	-0.313	-1.340	-0.724
Q9CZM2	Rpl15	0.017	0.080	0.110	0.000	-0.154	0.555	0.302	0.373
Q80UW8	Polr2e	0.036	0.126	0.000	-0.030	0.211	-0.203	-0.628	-0.347
Q9Z2A7	Dgat1	0.041	0.136	0.000	0.214	-0.404	0.617	0.361	0.650
Q99LJ6	Gpx7	0.000	0.012	0.150	-1.130	0.000	5.360	4.910	5.340
Q9WTX6	Cul1	0.660	0.936	0.000	-2.550	0.489	0.572	-1.490	-3.190
Q924Z4	Cers2	0.033	0.119	0.000	0.616	-0.229	0.912	0.981	0.929
Q9Z103	Adnp	0.796	1.000	-0.620	0.000	0.145	-0.140	-0.320	0.219
Q07113	Igf2r	0.000	0.008	0.000	-0.092	0.121	1.850	1.700	1.910
Q5XKN4	Jagn1	0.013	0.069	-0.346	0.043	0.000	0.703	0.682	1.270
Q9D706	Rpap3	0.076	0.205	0.123	-0.216	0.000	-0.201	-0.665	-0.416
Q9JHS3	Lamtor2	0.211	0.407	0.426	-0.037	0.000	0.206	-0.733	-0.512
A2AGT5	Ckap5	0.082	0.216	0.000	-0.236	0.124	-0.162	-0.706	-0.630
Q60865	Caprin1	0.278	0.499	0.134	0.000	-0.385	-0.068	-0.729	-0.380
Q8K019	Bclaf1	0.022	0.095	-0.039	0.072	0.000	-0.301	-0.823	-0.523
Q8BWF2	Gimap5	0.015	0.075	-0.145	0.000	0.167	-0.476	-0.802	-0.463
Q99MI6	Gimap3	0.015	0.075	-0.145	0.000	0.167	-0.476	-0.802	-0.463
Q8R323	Rfc3	0.159	0.336	0.374	0.000	-0.078	0.115	-1.040	-0.693
P97315	Csrp1	0.051	0.158	-2.340	0.000	1.880	2.360	4.050	4.750
Q8C522	Endod1	0.757	1.000	0.000	0.145	-0.042	0.232	-0.229	-0.045
Q9CQV8	Ywhab	0.002	0.028	-0.049	0.104	0.000	-0.851	-1.410	-1.150
Q921F2	Tardbp	0.080	0.211	0.004	0.000	-0.021	-0.142	-0.728	-0.349
P63280	Ube2i	0.764	1.000	0.000	0.245	-0.255	-0.134	-0.038	0.016
P68254	Ywhaq	0.006	0.046	-0.139	0.000	0.050	-0.858	-1.630	-1.230
Q8VDL4	Adpgk	0.170	0.352	0.785	0.000	-0.022	1.230	0.505	0.737
Q5XJE5	Leo1	0.033	0.119	-0.326	0.153	0.000	-0.839	-0.635	-1.470
Q4FK66	Prpf38a	0.020	0.088	0.219	-0.245	0.000	-0.513	-0.993	-0.699
Q9D855	Uqcrb	0.013	0.069	0.000	0.173	-0.233	0.669	0.448	0.554
Q5M8N4	Sdr39u1	0.975	1.000	-0.090	1.850	0.000	-0.753	1.040	1.390
Q8BRN9	Cc2d1b	0.026	0.105	0.000	0.668	-0.145	-1.050	-1.940	-0.829
Q8BH69	Sephs1	0.009	0.056	-0.042	0.000	0.303	-1.280	-1.270	-0.629
P70302	Stim1	0.048	0.152	0.074	0.000	-0.139	0.247	0.124	0.379
P19536	Cox5b	0.287	0.512	-0.201	0.104	0.000	0.204	-0.054	0.216
Q62093	Srsf2	0.063	0.181	0.470	-0.085	0.000	-0.167	-1.240	-1.120
Q8R050	Gspt1	0.272	0.492	0.345	-0.104	0.000	0.231	-0.805	-0.456
Q791V5	Mtch2	0.007	0.048	0.000	0.565	-0.017	1.160	1.140	1.190
P35279	Rab6a	0.697	0.973	0.155	0.000	-0.059	0.176	-0.128	0.205
P57784	Snrpa1	0.013	0.070	-0.141	0.155	0.000	-0.593	-0.649	-0.350
P14869	Rplp0	0.093	0.235	0.077	0.000	-0.078	0.564	0.057	0.406
P61022	Chp1	0.001	0.019	0.138	-0.075	0.000	1.490	1.050	1.450
Q8BLF1	Nceh1	0.001	0.019	0.000	0.056	-0.064	0.628	0.446	0.552
O54890	Itgb3	0.385	0.633	0.000	0.662	-0.700	-0.246	-0.257	-0.808
Q9JJ28	Flii	0.231	0.435	0.000	0.161	-0.371	-0.054	-0.816	-0.487
O70310	Nmt1	0.544	0.811	0.098	-0.428	0.000	0.144	-1.000	-0.217

Q3U1J4	Ddb1	0.021	0.091	-0.034	0.216	0.000	-0.354	-1.040	-0.931
Q9Z130	Hnrnpdl	0.499	0.764	0.000	0.040	-2.550	-0.201	-0.411	0.032
P62257	Ube2h	0.503	0.767	0.000	-0.443	0.167	-0.211	-0.606	-0.014
P62915	Gtf2b	0.008	0.054	0.170	0.000	-0.046	-0.454	-0.911	-0.672
O35250	Exoc7	0.017	0.080	-0.249	0.000	0.056	-0.422	-0.781	-0.742
P50172	Hsd11b1	0.261	0.477	0.205	0.000	-0.348	-0.044	-0.433	-0.522
Q6NZB0	Dnajc8	0.099	0.243	-0.028	0.000	0.279	-0.162	-0.578	-0.129
Q9CZY3	Ube2v1	0.009	0.057	0.000	-0.037	0.271	-0.626	-1.190	-0.739
Q9CY57	Chtop	0.143	0.309	-0.418	0.117	0.000	-0.544	-0.832	-0.228
Q9ET26	Rnf114	0.004	0.033	-0.147	0.271	0.000	-1.180	-1.710	-2.010
P35276	Rab3d	0.330	0.567	0.916	-0.442	0.000	0.434	-2.060	-0.646
Q91WN1	Dnajc9	0.034	0.122	-0.034	0.000	0.155	-0.381	-0.634	-0.195
Q7TNG5	Eml2	0.001	0.016	0.205	-0.013	0.000	-1.330	-1.840	-1.470
Q924K8	Mta3	0.030	0.114	-0.251	0.006	0.000	-0.565	-0.629	-0.314
Q9QXY6	Ehd3	0.016	0.079	-0.307	0.000	0.187	-0.555	-0.968	-0.899
Q62167	Ddx3x	0.437	0.694	0.572	-0.079	0.000	0.360	-0.179	-0.557
Q60996	Ppp2r5c	0.122	0.280	0.000	-0.686	0.064	-0.519	-1.030	-0.730
Q8BJY1	Psmd5	0.005	0.039	0.000	-0.013	0.093	-0.877	-1.520	-0.988
Q61699	Hsph1	0.043	0.141	0.078	-0.155	0.000	-0.310	-1.080	-0.742
Q99K41	Emilin1	0.416	0.670	0.000	0.316	-1.210	0.517	0.065	-0.112
P62911	Rpl32	0.562	0.831	0.000	0.444	-0.453	0.516	-0.354	0.583
Q9CWX9	Ddx47	0.062	0.178	0.000	-0.105	0.012	-0.508	-1.220	-0.386
Q04207	Rela	0.003	0.030	0.000	-0.006	0.032	-0.840	-1.440	-1.170
B2RUP2	Unc13d	0.001	0.019	-0.112	0.000	0.027	-0.618	-0.760	-0.569
Q7TQK1	Ints7	0.079	0.211	-0.115	0.068	0.000	-0.097	-0.432	-0.299
P21278	Gna11	0.000	0.008	-0.109	0.262	0.000	3.110	2.800	3.100
Q60605	Myl6	0.043	0.141	-0.374	0.009	0.000	0.591	0.152	0.570
Q80T69	Rsbn1	0.064	0.183	0.000	-0.349	0.070	-0.907	-0.869	-0.298
P09450	Junb	0.437	0.694	-0.450	0.000	0.615	-0.225	0.461	1.360
Q8VDD9	Phip	0.465	0.724	-0.464	0.000	0.167	-0.384	-0.642	0.057
Q811D0	Dlg1	0.287	0.512	0.849	-0.648	0.000	0.548	-2.010	-1.710
Q8K4B0	Mta1	0.791	1.000	0.000	-0.152	0.426	0.202	-0.311	0.179
P70280	Vamp7	0.346	0.587	-0.078	0.033	0.000	0.104	-0.400	-0.234
Q9QZE7	Tsnax	0.001	0.022	0.000	0.385	-0.165	-1.410	-1.900	-1.600
Q8R3N1	Nop14	0.332	0.570	-0.160	0.000	0.304	0.053	-0.677	-0.095
Q8BFP9	Pdk1	0.386	0.634	0.043	0.000	-0.101	0.101	-0.625	-0.165
Q99P81	Abcg3	0.243	0.453	0.251	0.000	-0.027	0.093	-0.358	-0.158
Q9CZ30	Ola1	0.090	0.230	0.164	0.000	-0.069	-0.178	-0.868	-0.330
Q6A0D4	Rftn1	0.286	0.511	0.030	0.000	-0.148	-0.005	-0.496	-0.184
Q99PP7	Trim33	0.047	0.150	-0.262	0.000	0.007	-0.335	-0.889	-0.588
Q99LM2	Cdk5rap3	0.442	0.699	0.000	-0.004	0.018	0.014	-0.017	0.147
Q6P1Y8	Inpp4b	0.035	0.125	-0.467	0.000	0.045	-1.560	-3.990	-2.020
O35604	Npc1	0.052	0.160	0.406	-0.105	0.000	0.918	0.427	0.686
P25444	Rps2	0.856	1.000	0.223	-0.069	0.000	0.402	-0.165	0.026
Q8R2M2	Dnttip2	0.473	0.733	-0.541	0.000	0.436	-0.515	-0.348	-0.001
Q3UMU9	Hdgfrp2	0.090	0.230	0.082	-0.189	0.000	-0.315	-2.380	-1.430
Q9JM76	Arpc3	0.056	0.166	0.000	-0.035	0.058	0.405	0.091	0.299
Q9EQ20	Aldh6a1	0.368	0.615	0.300	0.000	-0.150	1.460	-0.061	0.226
O55135	Eif6	0.023	0.096	0.057	-0.012	0.000	-0.371	-0.879	-1.130
Q9Z0S1	Bpnt1	0.290	0.515	-1.300	0.000	0.199	-3.920	-2.080	0.204
Q3UYV9	Ncbp1	0.003	0.030	-0.103	0.026	0.000	-0.664	-0.913	-0.589
Q8R0X7	Sgpl1	0.003	0.033	0.000	0.081	-0.094	1.270	0.727	1.060
Q8K2M0	Mrpl38	0.002	0.024	0.114	0.000	-0.089	0.995	0.671	0.855
P16125	Ldhb	0.000	0.013	-0.212	0.132	0.000	-1.330	-1.570	-1.550
Q9DC50	Crot	0.119	0.276	0.000	-0.124	0.034	-0.050	-0.367	-0.340
P62071	Rras2	0.017	0.081	0.120	0.000	-0.083	2.130	0.816	1.860
Q80XU3	Nucks1	0.047	0.149	0.000	-0.362	0.201	-0.682	-2.200	-1.300
P23591	Tsta3	0.050	0.155	0.000	-0.683	0.362	-0.877	-1.760	-2.630
P97300	Nptn	0.044	0.143	-0.405	0.611	0.000	0.707	1.630	1.810

Q9Z127	Slc7a5	0.005	0.043	0.399	0.000	-0.183	1.980	1.200	1.790
G5E829	Atp2b1	0.001	0.020	-0.137	0.083	0.000	1.270	0.902	1.290
O35435	Dhodh	0.072	0.196	0.000	0.359	-1.330	0.638	1.240	1.160
Q9D7X8	Ggct	0.001	0.019	-0.269	0.000	0.357	-2.090	-1.980	-2.600
O35691	Pnn	0.019	0.085	-0.156	0.000	0.067	-0.334	-0.570	-0.348
Q91YU8	Ppan	0.916	1.000	-0.220	0.149	0.000	0.107	-0.314	0.077
Q9QXG4	Acss2	0.016	0.078	-0.138	0.694	0.000	-1.240	-2.430	-1.330
P40237	Cd82	0.269	0.488	-0.180	0.212	0.000	-0.365	-0.262	0.030
Q922J3	Clip1	0.034	0.122	0.415	-0.534	0.000	-1.450	-1.440	-0.712
Q6P5B0	Rrp12	0.301	0.529	0.369	-0.321	0.000	0.174	-0.805	-0.596
P21995	Emb	0.102	0.247	-0.018	0.022	0.000	0.401	0.018	0.398
Q6P9J9	Ano6	0.002	0.023	0.138	0.000	-0.273	1.220	0.975	1.320
Q8BUM3	Ptpn7	0.004	0.038	0.125	-0.871	0.000	-2.120	-2.240	-1.970
P50518	Atp6v1e1	0.837	1.000	0.431	0.000	-0.221	0.368	-0.169	0.175
Q8BYW1	Arhgap25	0.003	0.033	-0.351	0.267	0.000	-1.140	-1.230	-1.400
O88441	Mtx2	0.276	0.496	0.000	0.113	-0.108	0.703	-0.135	0.393
Q6P9Q4	Fhod1	0.095	0.238	0.000	0.192	-0.114	-0.066	-0.781	-0.583
Q9R0I7	Ylpm1	0.058	0.171	0.000	0.437	-0.223	-0.318	-0.631	-0.534
Q921N6	Ddx27	0.244	0.454	0.486	0.000	-0.037	0.216	-0.895	-0.357
Q9Z1Z0	Uso1	0.335	0.573	0.000	0.009	-0.654	0.355	-2.660	-1.290
Q8R127	Sccpdh	0.064	0.183	-0.667	0.216	0.000	0.428	0.527	0.710
B1AZI6	Thoc2	0.998	1.000	0.453	-0.343	0.000	-0.050	-0.330	0.488
Q18PI6	Cd84	0.101	0.247	-0.282	0.224	0.000	-0.350	-1.080	-0.397
Q8BY87	Usp47	0.774	1.000	-1.190	0.000	0.428	-0.271	-1.430	0.297
Q8BJ03	Cox15	0.015	0.075	0.000	-0.638	0.346	1.240	1.100	1.810
Q8R164	Bphl	0.031	0.114	0.197	0.000	-0.133	0.714	0.305	0.778
Q3THG9	Aarsd1	0.001	0.019	-0.043	0.000	0.016	-0.899	-1.240	-1.330
Q920E5	Fdps	0.104	0.252	1.020	0.000	-0.336	1.880	0.878	1.120
Q9JII5	Dazap1	0.028	0.107	0.000	0.377	-0.062	-0.451	-0.655	-0.308
Q8K003	Tma7	0.203	0.399	0.000	-0.024	0.550	0.396	0.384	0.845
P28656	Nap1l1	0.113	0.267	0.177	-0.254	0.000	-0.193	-0.671	-0.353
Q9DAW6	Prpf4	0.005	0.039	0.000	0.312	-0.251	-0.873	-0.940	-1.050
Q3TW96	Uap1l1	0.784	1.000	-0.947	0.000	0.254	0.223	-0.282	-0.282
Q62383	Supt6h	0.838	1.000	0.314	-0.950	0.000	0.804	0.583	-1.490
O09130	Nfatc2ip	0.031	0.115	0.000	-0.134	0.049	-0.815	-2.260	-1.240
Q8VDM4	Psmd2	0.239	0.447	0.042	-0.316	0.000	-0.103	-0.642	-0.329
Q9DBF1	Aldh7a1	0.000	0.009	0.000	0.318	-0.191	2.700	2.460	2.520
Q99MR8	Mccc1	0.382	0.631	0.014	0.000	-0.317	0.811	-0.251	0.108
Q6ZQB6	Ppip5k2	0.003	0.033	0.000	0.042	-0.056	-0.461	-0.578	-0.345
P28063	Psmb8	0.001	0.014	-0.160	0.000	0.024	-1.350	-1.790	-1.440
P11438	Lamp1	0.050	0.156	0.166	-0.304	0.000	0.879	0.236	0.715
Q60790	Rasa3	0.846	1.000	0.000	0.108	-0.282	0.257	-0.193	-0.123
Q9CY97	Ssu72	0.032	0.118	-0.131	0.000	0.023	-0.703	-1.630	-0.740
Q925I1	Atad3	0.245	0.456	0.000	0.064	-0.526	0.689	-0.048	0.094
Q8R149	Bud13	0.404	0.655	0.211	-0.128	0.000	0.438	-0.129	-5.790
Q8BJ05	Zc3h14	0.250	0.462	0.000	-0.321	0.346	-1.400	-0.546	0.077
Q8BG05	Hnrnpa3	0.572	0.842	0.000	0.531	-0.203	-0.478	-0.064	0.294
Q9WVL0	Gstz1	0.112	0.265	0.000	0.163	-0.024	1.190	0.487	0.239
Q9DB25	Alg5	0.009	0.058	0.000	-0.448	0.027	0.870	0.529	0.843
O55143	Atp2a2	0.001	0.018	0.000	0.045	-0.311	1.460	1.100	1.270
Q6QD59	Bnip1	0.831	1.000	0.000	-0.712	0.039	-0.271	-0.640	0.026
P56376	Acyp1	0.087	0.224	0.000	-0.217	0.506	-0.816	-1.530	-0.252
P58771	Tpm1	0.375	0.623	0.000	0.208	-0.022	0.058	-0.868	0.051
Q7TNC4	Luc7l2	0.063	0.180	0.454	-0.011	0.000	-0.152	-1.330	-1.050
Q922H2	Pdk3	0.193	0.385	0.065	0.000	-0.219	0.781	-0.001	0.229
Q9CQJ6	Denr	0.330	0.568	0.249	-0.263	0.000	0.475	-0.043	0.254
Q00612	G6pdx	0.184	0.373	1.060	-0.237	0.000	0.116	-0.895	-0.924
P29452	Casp1	0.002	0.027	-0.321	0.027	0.000	-1.120	-1.610	-1.380
O54957	Lat	0.507	0.771	-0.255	0.567	0.000	0.081	0.134	0.862

Q05117	Acp5	0.229	0.433	0.477	0.000	-0.821	-0.165	-0.959	-1.720
Q3TZM9	Alg11	0.001	0.021	0.265	0.000	-0.147	1.390	1.080	1.260
Q6ZQ03	Fnbp4	0.001	0.017	0.000	-0.040	0.153	-0.833	-1.160	-1.090
Q99J62	Rfc4	0.246	0.457	0.532	-0.054	0.000	0.414	-0.865	-1.300
Q9CQU3	Rer1	0.001	0.015	0.049	0.000	-0.033	0.920	0.802	1.120
P56812	Pdcd5	0.007	0.049	-0.173	0.137	0.000	-1.160	-1.590	-0.851
Q5SVQ0	Kat7	0.002	0.025	-0.004	0.000	0.180	-0.725	-1.080	-0.767
Q8BGT7	Smndc1	0.168	0.349	-0.203	0.000	0.691	-1.140	-1.620	0.190
Q08481	Pecam1	0.631	0.907	0.000	1.060	-0.637	-0.841	0.355	-0.038
Q4VAA2	Cdv3	0.723	1.000	0.000	-0.140	0.131	0.438	-0.344	0.172
P61087	Ube2k	0.014	0.072	0.193	0.000	-0.032	-0.552	-1.380	-1.130
P59114	Pcif1	0.284	0.508	0.000	-0.966	0.255	-0.704	-2.550	-0.344
Q6PAM1	Txlna	0.425	0.679	0.512	-0.234	0.000	1.380	0.111	0.072
Q9EPL8	Ipo7	0.513	0.776	0.430	-0.700	0.000	0.125	-1.260	-0.270
Q9JMA2	Qtrt1	0.001	0.019	0.000	0.059	-0.158	-0.700	-0.758	-0.891
Q640N3	Arhgap30	0.048	0.151	-0.074	0.000	0.135	-0.461	-1.900	-1.130
P41241	Csk	0.102	0.248	0.002	0.000	-0.016	-0.072	-0.837	-0.511
Q3UBX0	Tmem109	0.186	0.376	0.000	-4.730	0.023	0.839	0.983	1.030
Q8R146	Apeh	0.003	0.031	-0.048	0.000	0.071	-1.000	-1.700	-1.640
Q8R0L9	Tada3	0.199	0.394	-0.089	0.639	0.000	-0.084	-0.212	-0.232
Q6P9N1	Fam126a	0.432	0.687	-0.621	2.050	0.000	0.332	1.680	1.890
Q8VCX5	Micu1	0.567	0.836	-0.165	0.177	0.000	1.300	-0.698	0.513
Q99JW4	Lims1	0.478	0.740	0.000	0.120	-0.598	0.349	-0.126	-0.073
Q6ZWR6	Syne1	0.727	1.000	0.859	-1.990	0.000	0.386	0.188	-0.688
Q99M87	Dnaja3	0.029	0.112	0.003	0.000	-0.329	0.713	0.216	0.625
Q80SU7	Gvin1	0.117	0.273	0.231	0.000	-0.019	0.045	-0.881	-0.733
B2RY04	Dock5	0.009	0.057	-0.206	0.037	0.000	-0.766	-1.150	-0.647
Q5SUF2	Luc7l3	0.021	0.091	0.264	0.000	-0.087	-0.609	-1.770	-1.470
Q09200	B4galnt1	0.004	0.036	0.052	-0.019	0.000	-0.568	-0.695	-0.380
Q06138	Cab39	0.004	0.036	0.105	-0.146	0.000	-0.761	-1.280	-1.010
Q9D379	Ephx1	0.000	0.012	0.114	-0.040	0.000	1.700	1.320	1.580
Q9JIF7	Copb1	0.018	0.084	0.000	0.412	-0.073	-0.488	-1.040	-1.230
Q9ESE1	Lrba	0.000	0.013	-0.292	0.080	0.000	-1.440	-1.500	-1.340
Q9CQ40	Mrpl49	0.092	0.233	0.339	0.000	-0.837	0.637	0.883	0.445
O08573	Lgals9	0.391	0.640	0.475	0.000	-0.125	0.251	-0.413	-0.271
Q6PR54	Rif1	0.046	0.148	0.000	-0.091	0.018	-0.254	-0.829	-0.460
Q9ESV0	Ddx24	0.175	0.360	0.000	0.189	-0.128	0.133	-1.170	-0.925
Q8CIM8	Ints4	0.007	0.048	-0.070	0.095	0.000	-0.300	-0.542	-0.502
Q6P6J9	Txndc15	0.896	1.000	0.417	0.000	-1.790	-0.136	-1.240	0.342
Q8VBW6	Nae1	0.028	0.108	-0.019	0.000	0.206	-0.776	-2.140	-1.120
Q3TGF2	Fam107b	0.166	0.347	0.000	-0.310	0.205	-0.458	-0.714	-0.106
P50171	Hsd17b8	0.804	1.000	-0.055	0.000	0.133	0.345	-0.437	-0.015
Q68FL4	Ahcyl2	0.099	0.244	0.091	-0.030	0.000	-0.016	-0.637	-0.577
Q60960	Kpna1	0.132	0.296	0.000	0.990	-0.334	-1.180	-0.338	-0.508
Q6PD26	Pigs	0.001	0.020	0.000	0.161	-0.055	0.792	0.665	0.633
P62267	Rps23	0.689	0.965	0.013	-0.028	0.000	0.271	-0.356	0.363
P16675	Ctsa	0.839	1.000	0.308	0.000	-0.004	1.220	-0.818	-0.515
Q8R3F5	Mcat	0.181	0.368	0.020	0.000	-0.178	0.816	-0.052	0.333
Q9DCN2	Cyb5r3	0.000	0.005	0.000	0.161	-0.051	2.080	1.950	2.120
O08550	Kmt2b	0.024	0.100	0.000	-0.032	0.324	-0.352	-0.570	-0.287
Q9DB15	Mrpl12	0.006	0.044	0.000	0.110	-0.240	0.803	0.551	0.609
Q921L3	Tmco1	0.013	0.070	0.210	0.000	-0.647	1.070	1.350	0.878
Q99NB1	Acss1	0.101	0.247	0.000	0.064	-0.261	-0.149	-0.573	-0.593
Q9EQI8	Mrpl46	0.017	0.081	0.194	0.000	-0.083	1.000	0.471	0.691
Q8R574	Prpsap2	0.015	0.076	0.000	-0.221	0.127	-0.692	-1.420	-0.898
O35639	Anxa3	0.011	0.063	0.503	0.000	-0.324	1.340	1.040	1.270
Q9WVJ3	Cpq	0.146	0.315	0.375	0.000	-0.250	-0.094	-0.745	-0.448
Q68FH4	Galk2	0.009	0.056	0.000	0.100	-0.032	-0.657	-1.280	-0.781
Q8C163	Exog	0.071	0.194	-0.225	0.654	0.000	0.703	0.765	1.070

O35609	Scamp3	0.623	0.898	-0.255	0.000	0.047	-0.117	-0.199	-0.056
P03930	Mtatp8	0.027	0.105	-0.070	0.024	0.000	0.738	0.252	0.830
P62270	Rps18	0.436	0.693	0.154	-0.073	0.000	0.130	-0.385	-0.087
Q80UM7	Mogs	0.021	0.090	0.000	0.122	-0.001	0.478	0.248	0.578
Q8C1B7	Sept 11	0.137	0.302	-0.140	0.191	0.000	0.444	0.081	0.326
Q80X82	Sympk	0.294	0.522	0.000	1.070	-0.036	-0.491	-0.519	0.363
Q5U458	Dnajc11	0.066	0.185	0.771	-0.042	0.000	1.780	1.580	0.657
P97311	Mcm6	0.032	0.116	0.272	-0.017	0.000	-0.280	-1.070	-0.927
Q8VDJ3	Hdlbp	0.079	0.209	0.211	0.000	-0.388	1.260	0.312	0.588
Q61292	Lamb2	0.013	0.070	0.471	-3.200	0.000	4.150	3.830	4.190
O08600	Endog	0.058	0.171	-0.172	0.406	0.000	-0.439	-0.313	-0.402
Q9ER00	Stx12	0.134	0.298	0.205	0.000	-1.040	1.720	-0.121	1.470
Q61263	Soat1	0.202	0.397	-1.560	0.617	0.000	0.381	0.756	1.000
P08030	Aprt	0.013	0.071	0.398	-0.078	0.000	-0.482	-0.881	-0.702
O35459	Ech1	0.045	0.146	0.000	0.120	-0.262	0.291	0.279	0.264
P47968	Rpia	0.160	0.337	0.718	-0.577	0.000	-0.741	-2.220	-0.389
Q91XD7	Creld1	0.012	0.067	0.000	0.730	-0.301	1.590	1.500	2.140
Q6ZPQ6	Pitpnm2	0.007	0.048	-0.296	0.000	0.192	-0.918	-1.170	-0.791
Q924C1	Xpo5	0.198	0.392	0.232	0.000	-0.052	0.150	-1.420	-0.684
Q8VDC0	Lars2	0.390	0.640	0.288	0.000	-4.090	0.945	-0.292	-0.213
Q8CEC6	Ppwd1	0.002	0.025	0.000	0.104	-0.053	-0.865	-0.835	-0.565
Q8K021	Scamp1	0.737	1.000	0.000	0.045	-0.281	-0.544	-0.201	1.040
P62908	Rps3	0.186	0.376	0.099	0.000	-0.178	-0.074	-0.632	-0.252
Q497V5	Srbd1	0.629	0.904	0.000	-0.755	0.097	-0.082	-0.855	-0.278
P34022	Ranbp1	0.071	0.195	0.406	-0.097	0.000	-0.243	-1.450	-0.795
Q9CRA8	Exosc5	0.006	0.046	-0.019	0.000	0.245	-0.631	-0.827	-0.461
Q8K2X3	Obfc1	0.025	0.101	-0.192	0.301	0.000	-0.567	-1.010	-0.532
Q3UHB1	Nt5dc3	0.001	0.014	0.000	0.448	-0.077	2.160	2.160	2.560
Q9ESU6	Brd4	0.610	0.884	0.000	0.102	-0.420	-0.365	-0.366	0.059
Q60775	Elf1	0.189	0.380	-0.298	0.000	0.179	-0.917	-2.490	-0.101
Q9JJK2	Lancl2	0.097	0.240	0.477	-0.052	0.000	-0.074	-0.884	-0.483
Q80YW0	Cyth4	0.013	0.070	0.000	-0.147	0.123	-0.448	-0.948	-0.724
Q9CWW7	Cxxc1	0.381	0.630	-0.019	0.834	0.000	-0.213	0.075	0.075
Q9DCJ5	Ndufa8	0.075	0.202	0.000	1.300	-0.142	1.220	1.620	2.010
O08529	Capn2	0.746	1.000	-0.207	0.000	0.072	0.305	-0.569	-0.147
Q9D020	Nt5c3a	0.008	0.053	0.000	0.443	-0.151	-0.917	-1.350	-0.880
Q62393	Tpd52	0.002	0.027	0.298	-0.017	0.000	-0.642	-0.624	-0.600
Q9Z0H4	Celf2	0.075	0.202	-0.319	0.000	0.094	-0.466	-0.487	-0.283
Q8VDP3	Mical1	0.105	0.252	-0.304	0.256	0.000	-0.186	-1.030	-1.600
Q921G7	Etfdh	0.009	0.055	0.011	0.000	-0.217	0.785	0.394	0.573
Q9JHS4	Clpx	0.557	0.825	0.335	0.000	-0.328	1.540	-0.635	0.363
Q8BK72	Mrps27	0.012	0.068	0.000	0.528	-0.256	1.200	1.050	1.510
O70475	Ugdh	0.023	0.096	0.529	0.000	-0.191	2.030	0.941	1.470
Q93092	Taldo1	0.006	0.044	0.013	0.000	-0.200	-1.010	-1.870	-1.490
O70293	Grk6	0.047	0.150	0.170	-0.136	0.000	-0.401	-1.250	-0.613
Q99LH1	Gnl2	0.400	0.651	-0.012	0.347	0.000	0.181	0.019	-0.682
Q03347	Runx1	0.085	0.221	-0.073	0.000	0.462	-0.343	-0.360	-0.146
Q9DBR1	Xrn2	0.619	0.893	0.182	-3.030	0.000	-0.079	-0.512	-0.558
Q8BVQ5	Ppme1	0.046	0.149	0.000	-0.304	0.094	-0.600	-2.320	-1.730
P58389	Ppp2r4	0.004	0.035	0.000	0.159	-0.258	-0.987	-1.580	-1.380
Q9D7S7	Rpl22l1	0.056	0.168	0.639	-0.126	0.000	1.190	0.683	0.870
Q9CQI3	Gmfb	0.027	0.105	-0.614	0.344	0.000	-1.170	-1.820	-1.060
Q6P8X1	Snx6	0.080	0.211	0.179	-0.169	0.000	-0.355	-1.340	-0.535
Q922S4	Pde2a	0.073	0.199	0.280	-0.058	0.000	-0.909	-2.760	-0.790
Q9Z2W0	Dnpep	0.888	1.000	-0.137	0.062	0.000	0.083	-0.199	0.092
Q6ZPR5	Smpd4	0.671	0.948	-0.120	0.000	0.029	0.054	-0.374	0.028
Q8R0F5	Rbmx2	0.033	0.119	0.125	0.000	-0.033	-0.510	-1.190	-0.479
Q62186	Ssr4	0.004	0.036	0.000	0.264	-0.140	1.460	1.390	2.160
Q8BFZ3	Actbl2	0.527	0.794	0.000	-1.890	1.230	-0.488	0.989	0.984

P48962	Slc25a4	0.000	0.013	0.000	0.359	-0.136	2.120	1.860	2.070
P14234	Fgr	0.214	0.412	0.781	0.000	-0.781	-0.032	-1.290	-1.570
Q61805	Lbp	0.929	1.000	1.500	0.000	-0.715	0.961	-0.240	-0.151
Q8K3H0	Appl1	0.122	0.280	-0.019	0.000	0.087	0.005	-0.899	-0.625
Q9CQU0	Txndc12	0.033	0.119	0.153	-0.268	0.000	0.484	0.520	1.080
Q9D2H6	Sp2	0.814	1.000	-0.748	0.000	0.314	-0.304	-0.704	0.256
Q9Z183	Padi4	0.348	0.589	0.465	-0.149	0.000	0.230	-0.725	-0.248
Q6ZPZ3	Zc3h4	0.051	0.157	0.058	-0.071	0.000	-0.559	-1.210	-0.369
Q9CQX2	Cyb5b	0.013	0.071	-0.164	0.064	0.000	0.975	0.423	0.947
P30412	Ppic	0.000	0.004	0.211	0.000	-0.045	4.510	4.650	5.030
Q8R1V4	Tmed4	0.003	0.033	0.009	0.000	-0.106	0.740	0.516	0.912
Q02257	Jup	0.002	0.027	-0.066	0.000	0.472	1.290	1.510	1.590
Q8CG46	Smc5	0.067	0.188	0.000	-0.111	0.174	-0.168	-0.552	-0.273
P42232	Stat5b	0.007	0.048	-0.226	0.000	0.150	-0.984	-1.490	-0.914
P55200	Kmt2a	0.043	0.142	-0.334	0.008	0.000	-0.582	-1.070	-0.506
Q91W98	Slc15a4	0.614	0.889	0.885	-1.730	0.000	0.497	-0.217	0.175
Q9CPQ3	Tomm22	0.010	0.061	-0.029	0.000	0.186	1.040	0.523	0.946
Q8C147	Dock8	0.007	0.050	-0.108	0.071	0.000	-0.609	-1.200	-0.943
Q9R1T2	Sae1	0.001	0.022	-0.100	0.000	0.086	-1.310	-1.900	-1.410
Q8BFQ4	Wdr82	0.490	0.754	-0.084	1.340	0.000	-0.615	-2.410	1.500
P70349	Hint1	0.003	0.030	-0.064	0.000	0.148	-1.010	-1.670	-1.260
Q9D2M8	Ube2v2	0.013	0.069	-0.093	0.000	0.331	-0.566	-1.180	-0.850
O70318	Epb41l2	0.014	0.073	0.087	-0.042	0.000	1.630	0.720	1.050
P70335	Rock1	0.917	1.000	-0.464	0.059	0.000	0.136	-0.564	0.117
Q8BWY3	Etf1	0.036	0.125	0.100	-0.068	0.000	-0.716	-1.340	-0.441
Q01405	Sec23a	0.212	0.409	0.000	-0.038	0.008	0.653	-0.087	0.364
Q9D287	Bcas2	0.044	0.145	-0.673	0.000	0.145	-1.230	-1.000	-0.769
P70224	Gimap1	0.037	0.127	-0.253	0.025	0.000	-0.508	-0.642	-0.305
Q6ZQ08	Cnot1	0.189	0.380	0.131	0.000	-0.006	0.096	-0.868	-0.442
Q9D1M0	Sec13	0.774	1.000	0.000	0.076	-0.324	-0.229	0.354	-0.169
P18155	Mthfd2	0.034	0.122	0.000	0.357	-0.941	0.795	1.290	1.380
O70546	Kdm6a	0.111	0.263	0.014	-0.113	0.000	-0.286	-0.636	-0.129
P70697	Urod	0.023	0.096	0.306	0.000	-0.652	-1.230	-2.010	-1.240
Q9QYJ3	Dnajb1	0.023	0.096	0.060	0.000	-0.117	-0.733	-1.600	-0.788
P26323	Fli1	0.274	0.494	0.000	-0.113	0.253	0.070	-0.670	-0.183
Q9DCZ4	Apoo	0.004	0.037	0.000	0.124	-0.197	0.733	0.566	0.860
P15920	Atp6v0a2	0.003	0.033	0.000	0.059	-0.066	0.643	0.434	0.744
Q61466	Smarcd1	0.126	0.287	-0.202	0.000	0.061	-0.260	-0.833	-0.252
Q8BSQ9	Pbrm1	0.031	0.114	0.000	0.231	-0.058	-0.850	-1.020	-0.291
Q9QVP9	Ptk2b	0.038	0.131	-1.180	0.133	0.000	-1.480	-1.840	-1.650
P62996	Tra2b	0.066	0.186	0.283	-0.028	0.000	-0.167	-0.937	-1.350
O88890	Sh2d1a	0.185	0.374	-0.831	0.461	0.000	-2.030	-0.784	-0.473
Q8CHI8	Ep400	0.615	0.889	-2.080	0.000	0.300	-0.174	-0.489	0.144
Q8K2H4	Acap1	0.037	0.129	-0.174	0.000	0.030	-0.286	-0.504	-0.266
Q8BWU5	Osgep	0.031	0.114	0.000	0.021	-0.337	-1.120	-2.230	-0.990
Q9DCM0	Ethe1	0.636	0.912	0.000	0.137	-0.820	0.135	-0.445	0.180
Q99N93	Mrpl16	0.172	0.355	0.000	0.020	-0.274	0.737	-0.068	0.326
Q91WQ5	Taf5l	0.070	0.193	-0.572	0.072	0.000	-0.475	-1.060	-0.998
Q78XF5	Ostc	0.000	0.012	0.000	0.306	-0.226	2.330	2.190	2.510
P62897	Cycs	0.205	0.401	0.000	0.081	-0.533	-0.300	-0.469	-0.708
Q9CXE7	Tmed5	0.012	0.068	0.000	0.150	-0.065	0.610	0.334	0.624
Q8BWG8	Arrb1	0.003	0.032	0.094	0.000	-0.186	-0.771	-1.060	-0.747
Q6NVG1	Lpcat4	0.557	0.825	1.640	-0.603	0.000	0.733	-2.380	0.392
P97352	S100a13	0.076	0.205	0.121	-0.269	0.000	-0.471	-1.150	-0.410
Q61990	Pcbp2	0.167	0.348	0.037	0.000	-0.006	-0.016	-0.517	-0.184
O35598	Adam10	0.952	1.000	0.337	0.000	-0.128	0.350	-0.150	0.048
P54729	Nub1	0.979	1.000	0.840	0.000	-0.638	1.160	0.303	-1.330
P84089	Erh	0.027	0.107	-0.352	0.000	0.217	-1.060	-2.580	-1.450
Q8BQ47	Cnpy4	0.431	0.687	-0.487	0.000	0.258	-1.090	-0.890	0.397

P13379	Cd5	0.724	1.000	0.000	0.432	-2.100	-0.529	-0.610	0.443
P31786	Dbi	0.001	0.019	0.000	0.100	-0.167	-1.290	-1.720	-1.290
P16045	Lgals1	0.944	1.000	0.000	1.160	-0.293	0.381	0.217	0.367
Q9WUK2	Eif4h	0.613	0.888	0.000	-0.268	0.057	-0.012	-0.659	0.052
P97821	Ctsc	0.036	0.126	0.633	-0.023	0.000	-0.313	-1.010	-0.904
P63325	Rps10	0.296	0.524	0.877	0.000	-0.932	1.130	0.005	1.120
Q9R045	Angptl2	0.066	0.186	0.000	1.160	-3.870	3.180	2.590	3.020
Q91WM3	Rrp9	0.048	0.152	0.000	-0.070	0.006	-0.208	-0.689	-0.376
P97370	Atp1b3	0.009	0.056	0.000	0.096	-0.048	0.414	0.419	0.703
P62751	Rpl23a	0.082	0.216	0.090	-0.198	0.000	0.425	0.101	0.242
Q9D0B0	Srsf9	0.011	0.066	0.020	0.000	-0.159	-0.652	-1.270	-0.798
Q9R062	Gyg1	0.243	0.454	0.654	0.000	-0.020	0.224	-0.695	-0.316
Q921I1	Tf	0.819	1.000	0.000	-0.479	0.122	0.389	-0.261	-0.276
Q9DAT2	Mrgbp	0.207	0.404	-0.081	0.000	0.111	-0.097	-1.350	-0.296
Q9R1E0	Foxo1	0.005	0.039	-0.131	0.075	0.000	-0.360	-0.460	-0.388
Q9WTR1	Trpv2	0.227	0.430	0.000	0.025	-0.046	0.378	-0.095	0.370
Q80TG1	Kansl1	0.986	1.000	-1.300	0.000	0.119	-0.573	-0.707	0.071
O70194	Eif3d	0.591	0.864	0.071	0.000	-0.109	0.166	-0.463	-0.074
Q99LR1	Abhd12	0.002	0.026	0.137	0.000	-0.385	1.300	1.020	1.320
P97310	Mcm2	0.021	0.090	0.214	-0.065	0.000	-0.388	-1.080	-0.810
Q62095	Ddx3y	0.466	0.724	0.597	-0.003	0.000	0.486	-0.267	-0.483
Q9JLI6	Scly	0.000	0.013	-0.263	0.000	0.067	-1.830	-1.890	-1.550
P26645	Marcks	0.049	0.154	0.063	0.000	-0.390	2.040	0.474	1.140
Q3UKJ7	Smu1	0.022	0.095	-0.083	0.468	0.000	-0.675	-0.709	-0.392
Q9DCU6	Mrpl4	0.041	0.138	0.000	0.039	-0.885	0.969	0.968	0.394
Q9ERL7	Gmfg	0.000	0.011	0.000	0.112	-0.049	-1.510	-1.900	-1.610
Q8R180	Ero1a	0.132	0.296	0.317	0.000	-0.244	0.636	0.184	0.436
Q8K190	Saysd1	0.726	1.000	-0.379	0.000	0.155	-0.127	-0.154	0.302
E9Q1P8	Irf2bp2	0.832	1.000	0.177	0.000	-0.061	-0.107	-0.185	0.296
Q99KU0	Vmp1	0.001	0.018	0.000	0.098	-0.354	1.510	1.260	1.280
Q8R5K4	Nol6	0.229	0.434	-0.275	0.000	0.070	-0.056	-0.418	-0.407
P51863	Atp6v0d1	0.117	0.273	0.335	0.000	-0.216	0.754	0.302	0.348
Q9CQW9	Ifitm3	0.039	0.133	1.730	0.000	-0.642	2.840	2.290	2.540
P62281	Rps11	0.867	1.000	0.187	-0.095	0.000	0.460	-0.436	-0.080
A2A432	Cul4b	0.030	0.112	-0.179	0.020	0.000	-0.399	-1.010	-0.636
O35984	Pbx2	0.209	0.405	-0.193	0.047	0.000	-0.257	-0.992	-0.140
P16460	Ass1	0.143	0.310	-0.587	0.000	0.018	0.512	0.153	0.075
Q9DCG9	Trmt112	0.673	0.950	0.000	-4.950	0.195	-0.811	-3.810	-2.730
P27870	Vav1	0.049	0.154	0.325	0.000	-0.114	-0.272	-1.240	-1.350
P01901	H2-K1	0.918	1.000	0.000	1.100	-0.068	-0.245	0.777	0.664
Q8VDG3	Parn	0.009	0.055	0.000	0.031	-0.430	-0.991	-1.720	-1.620
Q6P8I4	Pcnp	0.120	0.277	0.000	1.530	-0.170	-0.543	-0.732	-0.579
P63154	Crnkl1	0.013	0.069	-0.110	0.068	0.000	-0.499	-0.964	-0.564
Q9CR58	Slc25a30	0.011	0.062	0.000	0.243	-0.162	0.866	0.561	0.664
P62717	Rpl18a	0.672	0.949	0.000	-0.370	0.023	0.764	-0.398	-0.192
Q791T5	Mtch1	0.001	0.019	0.000	0.409	-0.005	1.910	1.480	1.660
P10630	Eif4a2	0.000	0.013	-0.146	0.000	0.209	-1.250	-1.450	-1.260
Q9D7G0	Prps1	0.019	0.087	0.000	-0.080	0.012	-0.415	-1.060	-1.060
Q9DB70	Fundc1	0.256	0.470	-0.517	1.650	0.000	2.470	0.712	1.270
Q9QWT9	Kifc1	0.255	0.469	0.245	0.000	-0.236	0.102	-1.330	-0.505
P18654	Rps6ka3	0.028	0.108	0.000	-0.035	0.109	-0.413	-1.370	-1.310
Q9JJI8	Rpl38	0.723	1.000	0.346	-0.062	0.000	0.418	0.040	0.031
Q99KF1	Tmed9	0.007	0.047	0.000	0.304	-0.089	0.916	0.738	1.130
P00375	Dhfr	0.067	0.187	0.141	-0.471	0.000	-0.723	-1.620	-0.675
P37913	Lig1	0.180	0.367	0.049	0.000	-0.161	0.072	-0.870	-1.240
P29533	Vcam1	0.146	0.315	0.746	0.000	-0.120	0.114	-1.040	-0.893
P59325	Eif5	0.094	0.236	0.365	0.000	-0.125	-0.059	-1.180	-0.979
Q9DC16	Ergic1	0.000	0.008	0.000	0.202	0.000	1.680	1.520	1.720
P43276	Hist1h1b	0.047	0.151	-0.012	0.000	0.126	-0.123	-0.379	-0.159

O88673	Dgka	0.006	0.046	0.000	0.135	-0.029	-0.968	-1.940	-1.570
Q61712	Dnajc1	0.294	0.522	0.000	-0.008	0.013	0.295	-0.127	0.666
Q9CXZ1	Ndufs4	0.012	0.067	-0.105	0.323	0.000	0.881	0.660	1.120
O70400	Pdlim1	0.000	0.014	-0.049	0.191	0.000	-0.883	-0.928	-0.774
P27046	Man2a1	0.004	0.038	0.100	0.000	-0.187	0.809	0.590	0.982
Q3TVI8	Pbxip1	0.001	0.017	-0.130	0.192	0.000	2.250	1.830	1.640
Q8BY71	Hat1	0.097	0.239	0.491	0.000	-0.180	-0.101	-1.180	-0.855
Q9CQD1	Rab5a	0.358	0.603	-0.260	0.000	0.058	0.299	-0.184	0.248
Q06185	Atp5i	0.001	0.020	0.000	-0.021	0.001	0.709	0.524	0.804
Q80X71	Tmem106b	0.015	0.076	-0.073	0.000	0.115	1.150	0.542	0.683
Q6Q477	Atp2b4	0.616	0.890	-0.793	0.069	0.000	-0.045	-0.917	-0.375
Q9JK81	Myg1	0.012	0.068	-0.941	0.000	0.003	-1.670	-1.890	-1.610
Q62388	Atm	0.107	0.257	-0.265	0.116	0.000	-0.451	-1.290	-0.368
Q6DVA0	Lemd2	0.002	0.024	-0.038	0.000	0.064	0.752	0.567	0.883
Q8BMS9	Rassf2	0.010	0.059	0.048	-0.167	0.000	-0.794	-1.680	-1.350
P18653	Rps6ka1	0.130	0.293	0.310	-0.305	0.000	-0.035	-2.120	-1.510
Q9JMD0	Znf207	0.085	0.221	-0.017	0.438	0.000	-0.369	-0.827	-0.122
P62137	Ppp1ca	0.822	1.000	0.237	-0.916	0.000	-0.030	-0.612	-0.318
Q8BQZ5	Cpsf4	0.017	0.080	0.000	0.329	-0.003	-0.392	-0.615	-0.331
A2BE28	Las1l	0.144	0.311	0.000	0.020	-0.132	-0.086	-0.921	-0.445
Q8BFV2	Pcid2	0.056	0.167	0.000	-0.229	0.168	-0.404	-1.510	-0.871
Q9CQN7	Mrpl41	0.001	0.019	-0.057	0.242	0.000	1.080	0.872	1.000
Q8BHS3	Rbm22	0.033	0.120	-0.099	0.000	0.141	-0.234	-0.540	-0.298
P08920	Cd2	0.013	0.069	-0.128	0.000	0.180	-0.418	-0.328	-0.464
O35114	Scarb2	0.362	0.607	0.450	-0.680	0.000	0.717	0.016	0.235
O35129	Phb2	0.003	0.030	0.000	0.062	-0.164	0.922	0.591	0.718
P61027	Rab10	0.511	0.776	0.322	0.000	-0.168	-0.098	0.011	-0.079
Q9D8T2	Gsdmdc1	0.014	0.073	0.032	0.000	-0.234	-0.817	-1.860	-1.440
Q9JI44	Dmap1	0.183	0.371	0.000	-0.120	0.075	-0.268	-0.446	-0.006
Q80TY0	Fnbp1	0.000	0.014	-0.105	0.201	0.000	-1.180	-1.500	-1.310
O70404	Vamp8	0.765	1.000	0.004	-0.083	0.000	0.196	-0.274	-0.138
Q8BYK4	Rdh12	0.365	0.612	0.037	0.000	-0.061	0.037	-0.072	-0.880
Q3UMY5	Eml4	0.063	0.180	0.000	-0.076	0.798	-1.170	-0.342	-0.609
Q62426	Cstb	0.075	0.202	0.000	0.417	-0.437	-0.446	-0.934	-0.684
P17047	Lamp2	0.008	0.053	0.337	0.000	-0.296	1.220	0.851	1.180
P97823	Lypla1	0.168	0.350	0.000	-0.250	0.196	0.058	-1.140	-0.961
P35585	Ap1m1	0.027	0.107	0.008	-0.381	0.000	-0.626	-1.250	-0.796
P54726	Rad23a	0.000	0.014	-0.136	0.081	0.000	-1.310	-1.450	-1.100
P62858	Rps28	0.085	0.221	0.213	-0.107	0.000	-0.184	-0.653	-0.247
Q61503	Nt5e	0.368	0.615	-0.517	0.000	0.009	-0.654	-0.461	-0.109
Q8BVF7	Aph1a	0.804	1.000	0.000	-0.832	2.030	2.600	-2.380	2.440
Q9CXW2	Mrps22	0.536	0.803	0.837	-1.070	0.000	1.960	1.660	-1.400
Q91VT4	Cbr4	0.890	1.000	0.000	0.188	-0.136	0.418	-0.152	-0.122
Q9JJZ4	Ube2j1	0.339	0.578	-0.229	0.072	0.000	0.103	-0.107	0.459
P61089	Ube2n	0.014	0.073	-0.057	0.000	0.306	-0.454	-0.788	-0.468
Q9JKF7	Mrpl39	0.494	0.759	0.000	-3.790	0.010	1.100	-0.144	-1.440
P20444	Prkca	0.030	0.113	-0.268	0.109	0.000	-0.390	-0.460	-0.434
P32037	Slc2a3	0.192	0.384	0.000	0.198	-1.110	-0.827	-0.767	-1.920
P50543	S100a11	0.359	0.603	0.112	-0.422	0.000	0.231	-1.160	-0.762
Q9JJZ6	Klf13	0.026	0.103	-0.094	0.000	0.094	-0.359	-0.751	-1.050
Q8BNW9	Kbtbd11	0.007	0.048	-0.061	0.000	0.260	-1.280	-2.450	-1.660
Q60787	Lcp2	0.009	0.057	-0.096	0.845	0.000	-1.390	-1.550	-1.040
Q02242	Pdcd1	0.396	0.646	-0.498	0.000	0.112	-0.263	0.315	0.333
Q9Z0L8	Ggh	0.394	0.643	-0.367	0.486	0.000	-0.505	-0.272	0.051
Q8VC03	Eml3	0.027	0.105	0.000	-0.105	0.575	-0.465	-0.766	-0.650
A2AQ19	Rtf1	0.008	0.052	0.023	-0.348	0.000	-0.937	-1.470	-1.690
Q8BG30	Nelfa	0.687	0.963	-0.364	0.000	0.565	-0.184	0.126	-0.112
Q91VR2	Atp5c1	0.035	0.125	0.127	0.000	-0.028	1.150	0.535	0.475
Q62425	Ndufa4	0.219	0.419	-0.214	0.210	0.000	0.195	0.073	0.541

P63242	Eif5a	0.208	0.404	0.466	-0.135	0.000	0.222	-1.650	-0.813
Q6ZQ58	Larp1	0.589	0.861	0.238	-0.291	0.000	0.329	-0.257	0.307
P46978	Stt3a	0.000	0.013	0.000	0.119	-0.213	1.570	1.280	1.410
Q9D5T0	Atad1	0.387	0.636	0.005	-0.010	0.000	0.504	-0.072	0.068
Q9JKX6	Nudt5	0.000	0.014	0.033	-0.029	0.000	-1.110	-1.520	-1.260
Q91YR7	Prpf6	0.053	0.162	0.000	0.162	-0.181	-0.213	-0.817	-0.784
Q6P1F6	Ppp2r2a	0.006	0.044	-0.191	0.010	0.000	-0.731	-1.310	-1.050
Q8K212	Pacs1	0.237	0.444	-0.105	0.819	0.000	0.622	0.460	1.960
P35980	Rpl18	0.134	0.298	0.241	-0.111	0.000	0.694	0.106	0.452
Q9R099	Tbl2	0.033	0.120	0.000	0.449	-0.050	0.634	0.647	0.976
Q64261	Cdk6	0.020	0.089	0.186	-0.124	0.000	-0.539	-1.450	-1.390
P63037	Dnaja1	0.340	0.580	0.072	0.000	-0.107	0.131	-0.492	-0.294
Q3V009	Tmed1	0.002	0.022	0.000	0.224	-0.659	3.120	2.230	2.850
Q8BGD9	Eif4b	0.065	0.184	0.178	-0.283	0.000	-0.447	-1.800	-1.000
Q8QZS1	Hibch	0.404	0.655	0.000	0.318	-0.092	0.156	0.135	0.318
Q8BYB9	Poglut1	0.033	0.121	0.293	-0.547	0.000	1.410	0.557	1.140
Q9EPJ9	Arfgap1	0.189	0.379	0.000	0.443	-0.044	-0.772	0.231	-1.060
Q99J56	Derl1	0.057	0.168	0.000	0.353	-0.292	0.483	0.610	0.489
Q8CBG9	Rnf170	0.048	0.152	-0.653	0.156	0.000	0.587	0.421	0.902
P15379	Cd44	0.076	0.205	0.008	-0.076	0.000	0.622	0.100	0.309
Q9CXT7	Tmem192	0.444	0.701	0.035	0.000	-0.127	-0.080	-0.516	0.051
Q99L04	Dhrs1	0.470	0.729	0.040	0.000	-0.227	0.315	-0.898	-0.478
Q4VBD2	Tapt1	0.005	0.039	0.000	0.047	-0.033	0.806	0.438	0.702
P70158	Smpdl3a	0.093	0.234	0.547	-0.004	0.000	-0.204	-1.990	-0.899
P05201	Got1	0.000	0.013	-0.145	0.019	0.000	-1.300	-1.680	-1.440
P83917	Cbx1	0.031	0.115	-0.155	0.623	0.000	-1.040	-0.915	-0.466
Q99KB8	Hagh	0.754	1.000	0.142	0.000	-0.442	-0.018	-0.512	-0.014
Q8VD65	Pik3r4	0.075	0.203	0.065	0.000	-0.273	-0.475	-2.190	-1.180
P70388	Rad50	0.176	0.360	0.000	-0.036	0.010	0.053	-0.597	-0.457
Q8BHA0	Ino80c	0.191	0.383	-0.478	0.295	0.000	-0.586	-0.487	-0.263
Q9D1C9	Rrp7a	0.568	0.837	0.000	1.660	-0.041	-1.260	0.528	0.770
Q05D44	Eif5b	0.751	1.000	0.544	0.000	-0.060	1.210	-0.708	0.591
Q91ZR1	Rab4b	0.047	0.151	0.000	-0.097	0.211	-0.147	-0.349	-0.366
Q91VX9	Tmem168	0.078	0.208	0.000	0.153	-0.166	0.492	0.337	0.135
Q9CWS4	Cpsf3l	0.800	1.000	-1.180	0.444	0.000	-0.383	-0.246	0.328
Q0VGB7	Ppp4r2	0.007	0.048	0.097	-0.490	0.000	-1.190	-1.610	-1.190
Q8VCG3	Wdr74	0.068	0.188	0.053	0.000	-0.255	-0.230	-0.618	-0.810
P48377	Rfx1	0.168	0.350	0.000	1.250	-1.230	-2.680	-3.290	-0.112
P03888	Mtnd1	0.570	0.839	-0.830	0.541	0.000	-0.768	0.658	1.100
Q91XA2	Golm1	0.144	0.311	0.162	-0.030	0.000	-0.479	-0.405	0.053
Q9JLJ2	Aldh9a1	0.081	0.213	0.030	-0.088	0.000	-0.239	-1.410	-0.779
Q9CR57	Rpl14	0.016	0.078	0.142	0.000	-0.271	0.753	0.423	0.557
Q689Z5	Sbno1	0.023	0.096	0.000	0.229	-0.069	-0.550	-1.670	-1.430
P31266	Rbpj	0.029	0.110	0.068	-0.003	0.000	-0.262	-0.886	-0.617
P62342	Selt	0.115	0.270	0.000	0.100	-0.390	0.284	0.120	0.255
P41245	Mmp9	0.231	0.435	2.280	0.000	-0.046	0.785	-1.710	-1.570
Q03147	Cdk7	0.029	0.110	0.000	-0.008	0.017	-0.257	-0.564	-0.237
Q8K2Y9	Ccm2	0.105	0.252	0.000	-0.079	0.056	-0.182	-0.525	-0.131
P61205	Arf3	0.041	0.138	0.110	0.000	-0.115	-0.215	-0.803	-0.684
P84078	Arf1	0.041	0.138	0.110	0.000	-0.115	-0.215	-0.803	-0.684
Q8CG48	Smc2	0.516	0.779	0.399	-0.110	0.000	0.404	-0.520	-0.271
Q6PFR5	Tra2a	0.863	1.000	-0.783	1.460	0.000	0.388	0.513	0.140
Q8C0M9	Asrgl1	0.000	0.011	0.000	-0.030	0.298	-1.320	-1.440	-1.400
Q8CI11	Gnl3	0.503	0.767	-0.054	0.404	0.000	0.261	-0.582	0.031
P59708	Sf3b6	0.380	0.630	0.000	0.753	-0.018	-1.090	0.063	0.292
Q9ERU3	Znf22	0.139	0.304	0.026	-0.097	0.000	-0.267	-2.210	-0.809
Q91VM9	Ppa2	0.070	0.193	-0.028	0.474	0.000	0.613	0.457	0.874
P10126	Eef1a1	0.463	0.721	0.222	-0.135	0.000	0.343	-0.593	-0.404
P13597	Icam1	0.007	0.050	0.000	0.319	-0.108	0.945	0.690	0.810

Q8C0C7	Farsa	0.044	0.144	0.000	0.024	-0.200	-0.294	-0.996	-0.832
P28658	Atxn10	0.868	1.000	0.308	0.000	-0.205	0.902	-0.791	-0.288
P28474	Adh5	0.003	0.032	-0.238	0.142	0.000	-1.150	-1.810	-1.400
O70551	Srpk1	0.012	0.067	0.029	0.000	-0.348	-0.612	-0.911	-0.979
Q9D6T0	Nosip	0.272	0.491	0.000	-0.462	0.101	-0.324	-0.810	-0.199
Q9ES97	Rtn3	0.173	0.357	0.075	0.000	-0.021	0.550	0.036	0.226
P10639	Txn	0.016	0.079	0.196	-0.365	0.000	-0.914	-2.060	-1.810
Q9D8P4	Mrpl17	0.062	0.179	-0.135	0.609	0.000	0.806	0.896	0.632
Q922D4	Ppp6r3	0.039	0.133	0.000	0.104	-0.045	-0.337	-1.250	-0.779
Q61144	Psen2	0.255	0.468	-0.215	0.716	0.000	0.567	0.830	0.354
P57716	Ncstn	0.012	0.067	0.058	0.000	-0.094	1.230	0.526	1.070
Q61187	Tsg101	0.722	1.000	0.000	0.043	-0.115	0.395	-0.280	0.042
Q9Z204	Hnrnpc	0.274	0.494	-0.174	0.244	0.000	-0.399	-0.244	0.040
Q923D2	Blvrb	0.072	0.196	0.052	0.000	-0.959	-0.794	-2.040	-1.690
Q91WG4	Elp2	0.084	0.220	0.228	-0.048	0.000	-0.173	-1.420	-0.763
P47791	Gsr	0.018	0.084	0.341	0.000	-0.118	-0.466	-1.080	-1.080
Q75N62	Gimap8	0.012	0.069	-0.070	0.000	0.009	-0.447	-0.926	-0.575
Q9JLT4	Txnrd2	0.839	1.000	0.000	0.351	-0.041	0.125	0.030	0.247
Q9DCD2	Xab2	0.010	0.058	-0.080	0.000	0.077	-0.302	-0.602	-0.563
Q9CQ69	Uqcrq	0.004	0.038	0.000	0.165	-0.022	0.638	0.470	0.464
Q9CZU6	Cs	0.005	0.041	0.000	0.137	-0.034	0.868	0.509	0.669
Q9D662	Sec23b	0.820	1.000	0.129	-2.060	0.000	-0.568	-1.170	-0.727
O88967	Yme1l1	0.017	0.081	0.000	-0.237	0.021	0.822	0.328	0.838
Q8K1R7	Nek9	0.096	0.238	-0.055	0.220	0.000	-0.017	-0.634	-0.594
P68181	Prkacb	0.979	1.000	-1.250	0.245	0.000	-0.353	-0.234	-0.378
Q9DBG5	Plin3	0.127	0.288	-0.512	0.000	0.399	-0.702	-1.290	-0.333
P48193	Epb41	0.179	0.364	0.171	0.000	-0.123	0.082	-0.784	-0.608
Q5DU31	Ipcef1	0.108	0.259	-0.777	0.000	0.265	-1.270	-2.170	-0.550
Q60648	Gm2a	0.038	0.129	0.000	-0.182	0.048	-0.348	-1.050	-0.713
P83940	Tceb1	0.011	0.064	-0.336	0.000	0.298	-1.000	-1.880	-1.370
Q9JHK5	Plek	0.404	0.655	0.273	0.000	-0.425	0.193	-0.584	-0.757
P35282	Rab21	1.000	1.000	-0.077	0.256	0.000	0.281	-0.147	0.045
Q6ZPY7	Kdm3b	0.027	0.107	0.000	0.495	-0.124	-0.530	-1.600	-1.240
Q8BU14	Sec62	0.007	0.049	0.047	-0.056	0.000	0.844	0.419	0.756
P46414	Cdkn1b	0.005	0.039	-0.490	0.000	0.483	-2.560	-3.040	-1.880
Q99J72	Apobec3	0.052	0.160	0.065	-0.374	0.000	-0.391	-1.200	-1.040
Q811U4	Mfn1	0.420	0.674	0.000	1.260	-0.739	2.090	1.140	-0.275
P35329	Cd22	0.083	0.218	-0.729	0.548	0.000	-3.200	-2.990	-0.495
Q99J47	Dhrs7b	0.010	0.059	0.000	0.380	-0.485	1.100	1.150	1.120
Q91YI0	Asl	0.025	0.102	-0.108	0.298	0.000	-1.820	-0.654	-2.060
P68433	Hist1h3a	0.422	0.676	0.323	-0.184	0.000	0.348	-0.110	0.569
P84228	Hist1h3b	0.422	0.676	0.323	-0.184	0.000	0.348	-0.110	0.569
P84244	H3f3a	0.422	0.676	0.323	-0.184	0.000	0.348	-0.110	0.569
Q62433	Ndrg1	0.116	0.271	0.000	0.021	-0.415	0.448	0.000	0.329
Q9D1R9	Rpl34	0.210	0.407	0.167	-0.106	0.000	0.638	0.073	0.196
Q9JHI7	Exosc9	0.019	0.087	-0.189	0.046	0.000	-0.782	-0.947	-0.406
Q9CQM5	Txndc17	0.008	0.055	-0.143	0.362	0.000	-1.500	-1.760	-2.830
Q9D6J5	Ndufb8	0.039	0.132	-0.884	0.149	0.000	0.958	0.797	0.597
Q9QZ06	Tollip	0.998	1.000	0.443	0.000	-0.223	0.233	-0.413	0.398
Q8BJ64	Chdh	0.375	0.623	0.970	-0.135	0.000	0.659	-1.190	-0.567
Q9Z1R2	Bag6	0.633	0.909	0.089	0.000	-0.303	0.178	-0.646	-0.160
Q6NZF1	Zc3h11a	0.003	0.032	-0.026	0.242	0.000	-0.859	-0.855	-0.564
Q8R0F3	Sumf1	0.002	0.024	0.000	0.232	-0.568	1.860	1.650	1.660
P48722	Hspa4l	0.006	0.047	-0.299	0.139	0.000	-1.170	-0.804	-1.320
Q810A7	Ddx42	0.810	1.000	0.110	-5.730	0.000	-0.901	-2.390	-0.792
Q9CQN6	Tmem14c	0.066	0.186	0.146	0.000	-0.210	0.760	0.282	0.302
Q9CQL5	Mrpl18	0.012	0.067	-0.027	0.185	0.000	0.925	0.512	1.060
Q8VEE4	Rpa1	0.072	0.197	0.054	-0.271	0.000	-0.678	-1.690	-0.560
Q03267	Ikzf1	0.113	0.266	-0.067	0.000	0.167	-0.242	-1.470	-0.496

Q8CI08	Slain2	0.911	1.000	-1.240	0.000	1.400	-0.021	-0.848	1.380
Q9CQR2	Rps21	0.095	0.237	0.311	0.000	-0.025	-0.024	-0.691	-0.461
Q8R2Q4	Gfm2	0.142	0.309	0.000	0.144	-0.128	0.042	-1.100	-0.903
P43274	Hist1h1e	0.210	0.406	0.191	0.000	-0.177	-0.111	-0.417	-0.115
Q76KJ5	Cd3eap	0.295	0.522	-0.247	0.366	0.000	-0.128	-0.249	-0.160
Q9D710	Tmx2	0.003	0.030	0.122	0.000	-0.249	0.828	0.670	0.867
O35316	Slc6a6	0.021	0.092	1.240	0.000	-0.326	3.830	2.060	2.770
Q9QYI3	Dnajc7	0.064	0.183	0.049	0.000	-0.092	-0.189	-0.999	-0.668
Q9JKB3	Ybx3	0.504	0.768	0.000	0.000	-0.501	0.146	-0.242	0.042
Q9CPT5	Nop16	0.751	1.000	0.000	0.393	-0.018	0.600	-0.657	0.037
P70403	Cux1	0.336	0.573	-2.080	0.000	1.250	0.628	0.354	1.600
Q9D8V0	Hm13	0.029	0.110	-0.063	0.378	0.000	0.955	0.512	0.731
Q9CPX6	Atg3	0.178	0.364	0.037	0.000	-0.063	-0.014	-0.809	-0.341
P97313	Prkdc	0.549	0.818	0.000	-0.329	0.216	-0.441	-0.410	0.217
Q9DBG7	Srpra	0.996	1.000	0.111	0.000	-0.032	0.308	-0.444	0.219
P31230	Aimp1	0.280	0.502	0.052	0.000	-0.151	0.072	-0.920	-0.350
Q99J95	Cdk9	0.029	0.110	0.000	-0.077	0.030	-0.617	-1.760	-1.040
Q8JZU2	Slc25a1	0.000	0.012	0.000	0.212	-0.083	1.600	1.430	1.730
Q3TJD7	Pdlim7	0.000	0.014	-0.291	0.000	0.069	2.390	2.110	2.820
Q6PD03	Ppp2r5a	0.406	0.658	0.305	-0.812	0.000	-0.080	-1.290	-0.489
Q8CG72	Adprhl2	0.003	0.033	-0.365	0.161	0.000	-1.260	-1.820	-1.390
Q8BXV2	Bri3bp	0.054	0.163	0.300	-0.665	0.000	1.270	0.551	0.721
Q62311	Taf6	0.460	0.719	-0.606	0.000	0.112	-0.200	-0.935	-0.177
Q99K23	Ufsp2	0.019	0.087	0.081	0.000	-0.422	0.696	0.388	0.640
P61924	Copz1	0.013	0.070	0.000	0.191	-0.211	-0.551	-0.896	-0.603
P31041	Cd28	0.187	0.376	-0.410	0.000	0.214	-0.507	-0.630	-0.161
Q60973	Rbbp7	0.041	0.138	-0.182	0.071	0.000	-0.476	-0.460	-0.205
Q9EP97	Senp3	0.650	0.926	-0.050	0.562	0.000	0.402	-0.247	-0.046
Q8BU88	Mrpl22	0.017	0.080	0.003	-0.068	0.000	0.895	0.346	0.595
P53702	Hccs	0.001	0.022	-0.130	0.313	0.000	1.420	1.460	1.860
Q6PGC1	Dhx29	0.175	0.359	-0.668	0.188	0.000	-0.453	-1.060	-0.550
Q9D787	Ppil2	0.371	0.618	-0.918	0.000	0.190	-0.048	-2.360	-0.653
Q80TJ7	Phf8	0.268	0.487	-0.121	1.600	0.000	0.400	-1.350	-0.462
P04202	Tgfb1	0.054	0.163	0.000	0.044	-0.363	-0.731	-0.344	-0.794
Q9WUM4	Coro1c	0.649	0.925	0.000	0.820	-0.303	0.531	0.356	0.152
Q8R502	Lrrc8c	0.354	0.597	-0.207	0.177	0.000	-0.160	-0.207	-0.043
O70572	Smpd2	0.081	0.213	0.525	-4.690	0.000	2.230	2.590	2.610
Q61036	Pak3	0.803	1.000	-1.200	0.000	0.506	-0.050	-0.933	-0.175
P36552	Cpox	0.116	0.271	0.330	0.000	-0.246	-0.140	-0.571	-0.479
Q9QUM9	Psma6	0.010	0.060	0.000	0.763	-0.042	-1.220	-1.070	-0.858
Q8R322	Gle1	0.568	0.837	0.082	0.000	-0.787	-0.422	-0.427	-0.374
P61021	Rab5b	0.370	0.617	0.000	-1.020	0.280	0.548	-2.430	-1.880
P99026	Psmb4	0.001	0.021	0.000	0.003	-0.115	-1.070	-1.620	-1.350
Q91V04	Tram1	0.196	0.389	0.000	2.330	-0.017	1.120	2.790	2.950
Q9D0G0	Mrps30	0.012	0.068	0.000	0.044	-0.091	1.010	0.479	0.609
Q9QYA2	Tomm40	0.010	0.059	0.000	0.202	-0.235	1.300	0.723	0.891
P12023	App	0.032	0.117	4.640	-1.820	0.000	7.180	7.130	7.230
Q9Z0M5	Lipa	0.093	0.234	0.471	0.000	-0.153	-0.105	-0.870	-0.654
Q62448	Eif4g2	0.342	0.582	0.405	-0.041	0.000	0.127	-0.804	-0.006
Q9Z0W3	Nup160	0.694	0.970	0.000	0.186	-0.070	-0.051	-0.145	0.162
P10922	H1f0	0.130	0.293	0.000	-0.145	0.190	-0.040	-1.060	-0.629
Q8CJG0	Ago2	0.043	0.141	-0.346	0.000	0.075	-0.501	-0.885	-0.493
O35144	Terf2	0.061	0.176	-0.147	0.408	0.000	-0.504	-0.507	-0.217
Q62446	Fkbp3	0.063	0.181	-0.025	0.000	0.005	-0.303	-0.475	-0.092
Q9JLQ0	Cd2ap	0.993	1.000	0.063	-1.320	0.000	-0.269	-1.480	0.514
Q3TLH4	Prrc2c	0.072	0.197	-0.333	0.208	0.000	-0.639	-1.180	-0.375
Q8BYH7	Tbc1d17	0.194	0.387	0.112	-0.197	0.000	0.008	-0.707	-0.450
P70290	Mpp1	0.605	0.878	0.118	0.000	-0.851	-0.046	-0.940	-0.420
P06342	H2-Ab1	0.941	1.000	0.000	1.270	-0.824	-0.149	0.502	0.243

Q8C208	Ikzf4	0.461	0.719	-0.864	0.212	0.000	-0.745	-0.405	-0.361
Q8VC28	Akr1c13	0.001	0.019	-0.340	0.000	0.003	-1.700	-2.330	-1.900
Q8BYM8	Cars2	0.412	0.665	0.076	0.000	-0.350	0.743	-0.258	0.119
Q64310	Surf4	0.002	0.029	0.000	0.103	-0.025	1.070	0.679	0.805
Q91YK2	Rrp1b	0.028	0.107	-0.039	0.046	0.000	-0.265	-0.819	-0.549
Q9CRA9	Fgfr1op2	0.213	0.409	-0.430	0.257	0.000	-0.553	-0.291	-0.299
Q6PGH1	Bud31	0.005	0.039	0.183	-0.025	0.000	-0.445	-0.774	-0.762
Q8BTX9	Hsdl1	0.769	1.000	0.000	0.163	-0.252	0.135	-0.129	0.040
Q61334	Bcap29	0.670	0.948	0.026	-0.020	0.000	0.419	-0.310	0.194
Q80US4	Actr5	0.919	1.000	-1.160	0.377	0.000	1.380	-1.400	-1.080
P60766	Cdc42	0.652	0.928	0.377	-0.109	0.000	0.126	-0.409	0.199
Q61072	Adam9	0.035	0.124	0.660	-3.660	0.000	3.520	2.950	3.250
P62331	Arf6	0.347	0.587	-0.116	0.120	0.000	0.221	-0.074	0.255
Q9CXW4	Rpl11	0.252	0.466	0.375	-0.080	0.000	0.127	-0.615	-0.243
Q8CBE3	Wdr37	0.204	0.400	0.000	0.548	-0.350	-0.276	-0.350	-0.371
O88455	Dhcr7	0.003	0.029	-0.220	0.000	0.700	2.230	1.960	2.310
Q63932	Map2k2	0.693	0.969	0.000	-0.792	0.019	0.453	-1.150	-0.779
P14131	Rps16	0.613	0.887	0.169	0.000	-0.079	0.180	-0.279	-0.059
P97434	Mprip	0.001	0.019	0.000	-0.488	0.742	3.430	3.150	3.770
Q8VE99	Ccdc115	0.807	1.000	-0.113	0.000	0.110	-0.028	-0.201	0.135
Q60770	Stxbp3	0.565	0.834	0.090	0.000	-0.054	0.526	-0.195	0.106
Q80UM3	Naa15	0.844	1.000	0.164	-1.690	0.000	0.202	-1.150	-1.040
Q9DBY8	Nvl	0.158	0.334	0.081	-0.257	0.000	-0.001	-0.936	-1.120
Q920Q4	Vps16	0.992	1.000	0.246	0.000	-0.008	0.326	-0.203	0.110
Q9CZ28	Snf8	0.469	0.728	0.006	-0.006	0.000	-0.028	-0.403	0.079
Q7TMM9	Tubb2a	0.111	0.264	0.000	0.407	-0.148	1.530	0.414	0.633
P04235	Cd3d	0.281	0.504	-0.353	0.097	0.000	-0.435	-0.393	-0.084
Q9CX99	Grap	0.077	0.207	-0.922	0.152	0.000	-1.990	-2.090	-0.687
Q99K70	Rragc	0.587	0.858	0.115	0.000	-0.184	0.184	-0.455	-0.160
Q8JZN7	Rhot2	0.010	0.061	0.084	0.000	-0.204	0.554	0.327	0.480
P47963	Rpl13	0.796	1.000	-0.012	0.034	0.000	0.497	-0.522	0.307
Q571I9	Aldh16a1	0.015	0.076	0.000	0.059	-0.391	-0.726	-1.380	-1.130
B1AZA5	Tmem245	0.094	0.236	0.000	-4.310	0.381	1.750	1.800	2.530
Q8R3C6	Rbm19	0.085	0.220	0.110	0.000	-0.230	-0.172	-1.180	-0.999
Q9Z0J0	Npc2	0.678	0.954	0.038	-0.325	0.000	0.294	-0.329	0.035
Q3UGR5	Hdhd2	0.093	0.234	0.000	0.123	-0.395	-0.377	-0.501	-0.971
P14115	Rpl27a	0.809	1.000	0.307	0.000	-0.285	0.495	-0.240	-0.018
Q9WUU9	Mcm3ap	0.519	0.784	0.121	0.000	-0.223	-0.095	-0.723	0.133
Q99KY4	Gak	0.193	0.385	0.471	-0.202	0.000	-0.838	-3.880	-0.314
A2AL36	Cntrl	0.237	0.444	0.000	-0.173	0.276	-0.143	-0.798	-0.066
Q80X41	Vrk1	0.007	0.048	0.000	-0.019	0.080	-0.523	-0.890	-0.509
O54734	Ddost	0.002	0.025	0.000	0.042	-0.469	1.490	1.120	1.220
Q9JIF0	Prmt1	0.208	0.404	-2.050	1.030	0.000	-1.680	-2.110	-1.400
Q149F5	Tmem71	0.621	0.895	-0.561	0.000	0.498	-0.795	-0.363	0.363
Q9CXJ4	Abcb8	0.014	0.074	0.000	0.642	-0.065	1.350	1.020	1.280
Q61142	Spin1	0.161	0.339	-0.044	0.000	0.623	-0.259	-0.903	-0.017
Q91WS0	Cisd1	0.396	0.646	-0.215	1.180	0.000	0.469	0.808	0.987
O09174	Amacr	0.030	0.113	0.000	0.612	-0.235	1.240	0.949	0.900
P62852	Rps25	0.696	0.972	0.125	-0.126	0.000	0.233	-0.473	-0.035
P60670	Nploc4	0.740	1.000	1.190	0.000	-0.041	0.721	-0.556	0.388
Q9JLN9	Mtor	0.116	0.271	0.095	0.000	-0.092	-0.022	-0.391	-0.444
Q9D7N3	Mrps9	0.122	0.280	0.267	-0.306	0.000	1.060	0.047	1.140
P22682	Cbl	0.000	0.013	-0.024	0.000	0.154	-1.190	-1.550	-1.380
P35922	Fmr1	0.469	0.729	0.174	-0.169	0.000	0.691	-1.830	-0.615
Q5SYD0	Myo1d	0.002	0.025	0.242	0.000	-0.716	3.190	2.310	3.330
Q6P9R1	Ddx51	0.054	0.164	0.000	0.058	-0.347	-0.583	-1.340	-0.615
Q8BWM0	Ptges2	0.946	1.000	-1.850	2.870	0.000	1.240	0.282	-0.189
Q8R5F7	Ifih1	0.396	0.645	0.526	0.000	-0.048	0.713	-1.360	-0.697
P24788	Cdk11b	0.579	0.850	0.534	-6.110	0.000	0.018	-1.130	-0.567

Q64522	Hist2h2ab	0.685	0.962	0.000	0.243	-0.083	-0.414	-0.437	0.562
Q99PU8	Dhx30	0.416	0.670	0.000	0.380	-0.875	1.330	-0.133	-0.053
Q9JL16	Isg20	0.132	0.296	0.573	0.000	-0.300	-0.033	-1.030	-1.080
Q8R035	Ict1	0.016	0.077	0.201	-0.053	0.000	1.010	0.503	0.676
Q9CPT4	Mydgf	0.595	0.868	-0.032	0.503	0.000	0.538	0.152	-1.160
Q8BK63	Csnk1a1	0.143	0.310	0.250	0.000	-0.233	-0.046	-0.568	-0.775
Q99020	Hnrnpab	0.020	0.088	-0.016	0.000	0.002	-0.493	-0.788	-0.309
Q05144	Rac2	0.029	0.112	0.254	-0.036	0.000	-0.369	-1.240	-0.844
P97872	Fmo5	0.485	0.748	0.755	-0.101	0.000	0.639	-0.667	-0.436
Q99J36	Thumpd1	0.295	0.522	-0.210	0.125	0.000	-5.210	-0.726	-0.026
Q7TMB8	Cyfip1	0.108	0.259	0.000	0.124	-1.260	0.703	0.315	0.700
Q3URS9	Ccdc51	0.007	0.048	0.194	0.000	-0.221	0.635	0.568	0.670
Q3KNM2	Marchf5	0.008	0.055	0.127	0.000	-0.352	1.370	0.744	1.400
P62737	Acta2	0.095	0.238	0.856	0.000	-0.683	1.790	0.877	3.750
P68033	Actc1	0.095	0.238	0.856	0.000	-0.683	1.790	0.877	3.750
Q8C2K1	Def6	0.035	0.125	0.000	-0.029	0.144	-0.322	-0.929	-0.442
Q9CQW2	Arl8b	0.497	0.763	0.125	0.000	-0.062	0.265	-1.000	-0.059
P28798	Grn	0.144	0.311	0.111	0.000	-1.300	-0.647	-1.980	-2.100
Q5DW34	Ehmt1	0.432	0.687	0.000	-0.729	0.184	-0.148	-1.100	-0.351
Q8BHZ4	Znf592	0.469	0.728	-0.184	0.000	0.011	0.150	-1.000	-0.161
Q9ET30	Tm9sf3	0.062	0.178	0.000	0.413	-0.902	0.864	0.711	0.992
Q8QZY9	Sf3b4	0.531	0.798	-2.010	0.478	0.000	-3.370	-0.510	-0.237
Q7TQH0	Atxn2l	0.095	0.238	-0.117	0.000	0.068	-0.604	-0.867	-0.096
Q91W59	Rbms1	0.033	0.121	0.806	-0.066	0.000	2.090	0.979	1.920
Q8VBV3	Exosc2	0.039	0.133	-0.349	0.471	0.000	-0.627	-0.957	-0.651
Q99LI2	Clcc1	0.023	0.096	-0.100	0.000	0.227	0.468	0.534	0.896
Q9WUA3	Pfkp	0.033	0.119	-0.074	0.000	0.023	-0.325	-1.040	-0.682
Q9DC70	Ndufs7	0.005	0.040	0.000	0.110	-0.184	0.684	0.466	0.575
Q3UND0	Skap2	0.206	0.403	0.518	0.000	-0.624	-0.258	-0.599	-1.130
A6PWY4	Wdr76	0.081	0.213	0.000	-0.414	0.003	-0.428	-1.580	-0.920
O70494	Sp3	0.078	0.208	-0.010	0.000	0.263	-0.254	-1.040	-0.332
Q91X84	Crtc3	0.778	1.000	-1.050	0.000	0.635	-0.708	-0.435	0.214
P84091	Ap2m1	0.321	0.556	0.149	0.000	-0.439	0.525	0.040	-0.018
P67871	Csnk2b	0.499	0.765	0.026	-0.042	0.000	0.326	-0.131	0.085
P55258	Rab8a	0.478	0.740	0.000	0.081	-1.520	0.007	-0.248	0.041
P48428	Tbca	0.000	0.003	0.052	0.000	-0.010	-1.980	-2.160	-2.200
Q9CY27	Tecr	0.001	0.019	0.000	0.089	-0.113	1.390	0.978	1.330
Q9QZD9	Eif3i	0.115	0.269	0.000	0.168	-0.055	-0.088	-1.120	-0.527
Q62158	Trim27	0.431	0.687	0.111	-0.657	0.000	0.009	-1.310	-0.441
P26516	Psmd7	0.010	0.059	0.000	0.058	-0.009	-0.723	-0.864	-0.382
Q60634	Flot2	0.111	0.264	0.405	0.000	-0.394	1.280	0.318	0.643
Q8CAS9	Parp9	0.397	0.647	0.000	-0.344	0.030	0.545	-1.360	-1.310
Q91WQ3	Yars	0.766	1.000	0.202	-0.103	0.000	0.408	-0.347	-0.198
Q8CI32	Bag5	0.037	0.129	0.971	0.000	-0.099	-0.561	-1.150	-0.939
Q60749	Khdrbs1	0.078	0.208	-0.028	0.000	0.031	-0.155	-0.632	-0.249
Q99KG3	Rbm10	0.006	0.046	-0.268	0.000	0.234	-0.923	-1.030	-0.744
Q9EP89	Lactb	0.009	0.055	0.000	0.227	-0.004	0.611	0.438	0.673
Q9D3D9	Atp5d	0.126	0.287	-0.224	0.095	0.000	0.573	0.056	0.267
Q80W00	Ppp1r10	0.269	0.487	0.000	-0.002	0.060	-0.042	-1.260	-0.145
Q9DC48	Cdc40	0.029	0.110	0.078	0.000	-0.189	-0.334	-0.713	-0.444
Q9D0F6	Rfc5	0.453	0.711	0.422	0.000	-0.107	0.315	-0.450	-0.249
E9Q6J5	Bod1l	0.006	0.047	-0.187	0.000	0.048	-0.937	-1.340	-0.761
Q8BT60	Cpne3	0.133	0.297	1.050	-0.584	0.000	-0.468	-1.280	-0.789
Q9JL61	Rfx5	0.105	0.252	-0.505	0.086	0.000	-1.050	-0.889	-0.307
Q8C0L0	Tmx4	0.533	0.800	0.070	-0.116	0.000	0.113	-0.118	0.169
Q9CR67	Tmem33	0.011	0.064	0.000	0.299	-0.015	1.150	0.633	1.090
O08912	Galnt1	0.941	1.000	0.124	-0.001	0.000	0.241	-0.251	0.096
O88384	Vti1b	0.769	1.000	0.080	0.000	-0.234	0.387	-0.713	-0.140
O88986	Gcat	0.640	0.917	-0.398	0.000	0.308	0.250	-0.442	-0.349

Q9WV80	Snx1	0.053	0.162	0.124	0.000	-0.011	-0.162	-0.776	-0.438
P70318	Tial1	0.250	0.463	-0.420	0.005	0.000	-0.234	-0.572	-0.309
Q9WU00	Nrf1	0.250	0.462	0.000	0.342	-0.076	-0.339	-0.325	0.125
Q9JKX4	Aatf	0.350	0.592	0.000	0.709	-1.030	-0.247	-0.828	-1.010
O54946	Dnajb6	0.385	0.634	-0.028	0.357	0.000	-0.199	-0.392	0.254
Q6KCD5	Nipbl	0.198	0.392	-0.044	0.000	0.205	0.043	-0.828	-0.282
Q9EST5	Anp32b	0.003	0.033	-0.081	0.000	0.074	-0.928	-1.530	-1.580
O35685	Nudc	0.046	0.149	0.279	-0.199	0.000	-0.351	-1.340	-0.968
Q7TSC1	Prrc2a	0.109	0.260	-0.311	0.000	0.082	-0.383	-0.282	-0.324
P59729	Rin3	0.908	1.000	0.881	-4.610	0.000	-0.058	-4.180	-0.287
O55234	Psmb5	0.002	0.022	0.000	-0.040	0.005	-0.534	-0.717	-0.480
Q9Z0P4	Palm	0.078	0.209	4.740	0.000	-1.440	5.760	4.970	5.890
Q9JHI5	Ivd	0.333	0.571	0.000	0.127	-0.011	0.544	-0.052	0.212
Q922J9	Far1	0.001	0.020	0.251	0.000	-0.223	1.600	1.300	1.720
Q8BLN5	Lss	0.000	0.011	0.000	-0.076	0.465	3.020	2.690	3.120
Q64685	St6gal1	0.007	0.047	-0.227	0.000	0.149	-0.709	-0.705	-0.551
O35864	Cops5	0.075	0.202	-0.163	0.000	0.098	-1.120	-1.010	-3.290
Q9CPP6	Ndufa5	0.245	0.456	-0.536	0.555	0.000	0.694	0.147	0.649
P15532	Nme1	0.004	0.038	0.000	0.373	-0.032	-0.827	-1.420	-1.230
P70271	Pdlim4	0.116	0.271	0.000	0.283	-1.460	0.861	0.415	0.941
Q9D0M1	Prpsap1	0.155	0.330	-0.116	0.000	0.125	-0.059	-2.380	-1.090
Q8CB77	Tceb3	0.095	0.237	-0.143	0.026	0.000	-0.321	-0.886	-0.258
Q99LD9	Eif2b2	0.175	0.359	0.033	-0.145	0.000	-0.005	-0.644	-0.430
P17879	Hspa1b	0.401	0.652	0.000	-1.290	0.818	0.709	0.226	0.366
Q61696	Hspa1a	0.401	0.652	0.000	-1.290	0.818	0.709	0.226	0.366
O88544	Cops4	0.001	0.019	-0.084	0.083	0.000	-0.508	-0.674	-0.571
O55125	Nipsnap1	0.039	0.134	-0.109	0.000	0.160	0.335	0.199	0.384
Q8BZR9	Ncbp3	0.042	0.139	0.000	-0.357	0.139	-0.557	-1.920	-1.740
Q8BX10	Pgam5	0.212	0.409	-0.303	0.066	0.000	0.400	-0.081	0.256
Q8CG47	Smc4	0.107	0.256	0.051	0.000	-0.195	-0.164	-1.130	-0.645
Q9Z1T1	Ap3b1	0.903	1.000	0.355	0.000	-0.119	0.549	-0.293	0.090
P70245	Ebp	0.136	0.301	-0.271	0.136	0.000	0.523	0.032	0.355
P41105	Rpl28	0.677	0.953	0.058	-0.160	0.000	0.474	-0.870	-0.236
Q8C5P7	Tdrp	0.289	0.514	0.000	0.540	-3.900	-0.646	0.680	3.180
Q921G6	Lrch4	0.128	0.290	0.000	0.144	-0.354	-0.491	-0.524	-1.780
P48410	Abcd1	0.804	1.000	0.401	-0.082	0.000	0.518	-0.244	0.258
P07091	S100a4	0.014	0.073	-0.132	0.000	0.151	-0.450	-0.771	-0.446
Q9D1I2	Card19	0.027	0.106	-0.929	0.000	0.091	1.010	0.663	1.220
Q99LI5	Znf281	0.332	0.569	-0.459	0.034	0.000	-0.422	-0.184	-0.403
Q99M28	Rnps1	0.025	0.101	0.023	0.000	-0.010	-0.173	-0.526	-0.366
P19253	Rpl13a	0.055	0.166	0.072	-0.126	0.000	0.649	0.148	0.402
Q9D0R4	Ddx56	0.301	0.530	-0.058	0.000	0.322	0.224	-0.377	-0.453
Q9JJU8	Sh3bgrl	0.001	0.016	-0.279	0.031	0.000	-1.640	-2.170	-2.160
Q9D753	Exosc8	0.007	0.050	-0.219	0.000	0.109	-0.795	-0.804	-0.526
Q8C8U0	Ppfibp1	0.004	0.036	-1.930	0.000	0.118	4.310	3.430	3.420
P20491	Fcer1g	0.868	1.000	0.025	0.000	-0.757	0.174	-0.883	-0.237
P08226	Apoe	0.508	0.772	0.861	-0.299	0.000	0.960	0.161	0.360
Q62384	Zpr1	0.001	0.019	0.000	-0.018	0.224	-1.120	-1.420	-1.640
Q9DAV9	Tmem38b	0.014	0.073	0.128	0.000	-0.142	0.489	0.347	0.671
Q3UYC0	Ppm1h	0.126	0.287	-0.049	0.465	0.000	-0.111	-0.156	-0.485
O35405	Pld3	0.088	0.227	0.695	-0.043	0.000	-0.040	-1.100	-0.976
P46061	Rangap1	0.066	0.186	-0.024	0.000	0.159	-0.246	-0.623	-0.156
Q6ZQH8	Nup188	0.536	0.803	0.000	0.306	-0.291	-0.168	-0.324	0.082
Q9CQS8	Sec61b	0.002	0.025	0.034	0.000	-0.122	1.150	0.720	0.971
Q61686	Cbx5	0.059	0.173	0.129	-0.280	0.000	-0.287	-1.000	-0.770
P97480	Eya3	0.133	0.296	0.000	-0.166	0.589	-0.090	-2.020	-0.889
Q6A009	Ltn1	0.985	1.000	0.052	0.000	-0.178	0.434	-0.683	0.102
P12382	Pfkl	0.013	0.070	-0.225	0.544	0.000	-0.976	-1.550	-0.976
Q8BGC0	Htatsf1	0.081	0.213	0.000	-0.444	0.487	-1.040	-4.100	-1.620

O35648	Cetn3	0.061	0.176	0.000	-0.110	0.216	-0.144	-0.637	-0.458
Q9R1K9	Cetn2	0.007	0.048	0.000	-0.087	0.218	-0.638	-1.140	-0.782
Q63844	Mapk3	0.097	0.239	0.625	-0.043	0.000	-0.104	-0.713	-0.406
Q8JZM7	Cdc73	0.003	0.033	0.000	0.219	-0.127	-0.762	-1.210	-1.040
P50429	Arsb	0.524	0.790	0.190	-1.080	0.000	-0.004	-1.780	-0.486
Q3B7Z2	Osbp	0.301	0.529	0.000	-0.645	0.139	-0.287	-1.600	-0.361
Q8BMC4	Nop9	0.058	0.171	-0.300	0.866	0.000	-0.880	-0.720	-1.950
P42228	Stat4	0.004	0.034	-0.425	0.000	0.128	-1.440	-1.590	-1.150
Q8VEH3	Arl8a	0.427	0.683	0.000	0.064	-0.007	0.164	-2.160	0.049
Q8C7R4	Uba6	0.049	0.154	-0.131	0.000	0.079	-0.551	-1.370	-0.519
Q61216	Mre11a	0.103	0.249	0.000	0.005	-0.020	-0.225	-0.399	-0.043
Q9CQT1	Mri1	0.010	0.059	-0.208	0.000	0.005	-1.030	-2.150	-1.650
Q99KK2	Cmas	0.118	0.274	0.000	0.039	-0.028	-0.189	-0.483	-0.065
P58854	Tubgcp3	0.950	1.000	0.322	0.000	-0.040	0.316	-0.178	0.107
Q60854	Serpinb6	0.023	0.096	0.000	0.176	-0.003	-0.265	-0.736	-0.439
Q8K3K7	Agpat2	0.136	0.300	0.673	-0.697	0.000	0.782	0.680	0.732
Q9D8S3	Arfgap3	0.403	0.655	0.116	-0.337	0.000	0.416	-0.238	0.275
O88942	Nfatc1	0.007	0.049	0.000	0.025	-0.018	-0.426	-0.651	-0.342
P30416	Fkbp4	0.034	0.122	0.210	-0.131	0.000	-0.417	-1.450	-1.070
Q9WTP6	Ak2	0.009	0.057	0.187	0.000	-0.011	-0.394	-0.833	-0.616
Q5I012	Slc38a10	0.015	0.076	0.000	0.102	-0.889	1.920	0.930	1.770
Q8CIV2	Tmem259	0.933	1.000	0.000	-0.305	3.950	1.260	4.740	-1.730
Q8BG51	Rhot1	0.015	0.076	0.000	0.146	-0.220	0.857	0.556	0.462
Q9ERI2	Rab27a	0.016	0.078	-0.088	0.258	0.000	-0.439	-0.539	-0.320
Q8K327	Champ1	0.072	0.197	0.000	0.682	-0.760	-1.110	-0.962	-1.050
Q8K3Z9	Pom121	0.636	0.912	-0.467	0.020	0.000	-0.303	-0.455	1.110
Q60759	Gcdh	0.450	0.708	0.000	0.355	-0.371	0.773	-0.053	0.092
Q91X78	Erlin1	0.006	0.047	0.506	0.000	-0.214	1.620	1.150	1.490
O08808	Diaph1	0.008	0.053	0.000	0.331	-0.056	-0.629	-1.230	-1.130
E9Q3L2	Pi4ka	0.023	0.096	0.000	-0.187	0.098	-0.443	-1.100	-0.794
P47226	Tes	0.054	0.164	-0.065	0.000	0.382	-0.256	-1.230	-0.735
P20108	Prdx3	0.006	0.046	0.214	-0.058	0.000	0.778	0.509	0.675
P28076	Psmb9	0.000	0.008	0.000	0.158	-0.071	-1.320	-1.440	-1.440
Q922Q2	Riok1	0.209	0.406	0.319	-0.036	0.000	0.212	-1.780	-0.775
Q3U2A8	Vars2	0.089	0.227	0.000	0.110	-0.376	0.144	0.368	0.307
Q99NB8	Ubqln4	0.918	1.000	-0.973	0.205	0.000	-0.248	-0.431	-0.210
P61961	Ufm1	0.109	0.260	0.161	-0.247	0.000	-0.168	-0.732	-0.429
Q80YD1	Supv3l1	0.046	0.149	0.000	0.195	-0.039	0.533	0.318	0.254
Q9WUM3	Coro1b	0.004	0.036	0.000	0.494	-0.016	-0.912	-0.833	-0.805
P62245	Rps15a	0.129	0.291	0.000	0.085	-0.316	-0.152	-0.630	-0.945
Q6ZWQ0	Syne2	0.001	0.014	-0.133	0.058	0.000	-0.906	-1.160	-0.960
Q9D162	Ccdc167	0.095	0.237	0.528	-0.276	0.000	1.370	0.371	2.040
Q6NSR8	Npepl1	0.008	0.053	-0.164	0.000	0.177	-0.998	-1.940	-1.350
B1AY13	Usp24	0.332	0.570	0.320	-0.715	0.000	0.167	-1.930	-0.876
Q6NZN0	Rbm26	0.151	0.323	0.000	-0.189	0.425	-0.493	-1.240	-0.077
O35737	Hnrnph1	0.264	0.482	-0.105	0.106	0.000	0.047	-0.222	-0.287
Q01768	Nme2	0.028	0.109	0.000	0.131	-0.266	-0.484	-1.090	-0.705
Q9D8Y8	Ing5	0.137	0.302	-0.463	0.040	0.000	-0.637	-0.577	-0.289
Q921F4	Hnrnpll	0.081	0.213	-0.224	0.000	0.360	-0.213	-0.501	-0.736
Q8K337	Inpp5b	0.001	0.019	-0.129	0.000	0.150	-1.020	-1.310	-1.430
Q62077	Plcg1	0.003	0.033	-0.125	0.000	0.471	-1.190	-1.370	-1.750
Q9DBC0	Selo	0.169	0.350	0.118	-0.027	0.000	0.290	-2.830	-2.140
Q99KK9	Hars2	0.327	0.564	0.000	0.815	-0.658	0.593	0.065	1.500
Q8BMQ2	Gtf3c4	0.311	0.544	-0.322	0.051	0.000	-0.220	-0.870	-0.103
Q99KP3	Cryl1	0.002	0.027	-0.510	0.000	0.271	-1.670	-2.060	-2.120
Q8BHG9	Cggbp1	0.310	0.542	-0.314	0.000	0.385	-0.552	-0.518	0.112
Q3UDE2	Ttll12	0.009	0.058	-0.158	0.219	0.000	-0.662	-1.330	-1.070
P54822	Adsl	0.060	0.175	-0.145	0.046	0.000	-0.266	-0.814	-0.390
P41242	Matk	0.064	0.183	-0.128	0.000	0.032	-0.458	-1.140	-0.374

O54825	Bysl	0.040	0.134	0.000	0.199	-0.082	-0.345	-0.495	-0.172
Q60591	Nfatc2	0.930	1.000	0.183	-1.590	0.000	-0.316	-0.458	-0.478
Q91V12	Acot7	0.020	0.089	-0.038	0.193	0.000	0.736	0.449	0.396
Q8BWD8	Cdk19	0.857	1.000	-1.270	0.000	0.173	-0.518	-0.267	-0.035
Q8C5L3	Cnot2	0.073	0.198	-0.390	0.000	0.063	-0.329	-1.130	-0.861
Q6PEB6	Mob4	0.779	1.000	-0.331	0.004	0.000	0.295	0.251	-0.595
Q8BHF7	Pgs1	0.017	0.080	0.000	1.120	-0.175	2.030	1.790	2.010
Q8K3J1	Ndufs8	0.002	0.025	0.000	0.081	-0.026	0.747	0.504	0.553
Q64433	Hspe1	0.001	0.018	0.000	0.100	-0.080	1.090	0.779	0.991
Q8C5N3	Cwc22	0.023	0.096	0.000	0.191	-0.464	-0.905	-2.000	-1.370
O70161	Pip5k1c	0.114	0.268	-0.827	0.696	0.000	0.554	0.961	1.390
Q80UJ7	Rab3gap1	0.391	0.640	-0.189	0.000	0.108	0.130	-0.432	-0.341
Q80XP8	Fam76b	0.240	0.449	0.252	-1.180	0.000	0.005	-3.390	-2.020
Q3V1L4	Nt5c2	0.047	0.149	0.000	0.109	-0.392	-0.430	-1.090	-0.869
Q9CXG3	Ppil4	0.025	0.103	0.000	-0.294	0.128	-0.723	-1.650	-0.961
Q6ZWN5	Rps9	0.784	1.000	0.205	0.000	-0.149	0.324	-0.286	-0.170
Q3ULJ0	Gpd1l	0.045	0.146	0.027	0.000	-0.287	-0.381	-1.230	-1.180
O35215	Ddt	0.032	0.118	-0.099	1.310	0.000	-3.690	-1.070	-2.560
Q9DB27	Mcts1	0.959	1.000	0.152	-0.168	0.000	0.247	-0.337	0.042
Q9WUP4	Srd5a3	0.212	0.409	0.098	-1.150	0.000	0.613	-0.036	0.341
Q9JKC8	Ap3m1	0.127	0.288	0.404	0.000	-0.143	0.882	0.358	0.385
Q99KQ4	Nampt	0.129	0.291	0.000	0.160	-0.599	-1.710	-1.790	-0.193
Q9WUA2	Farsb	0.086	0.223	0.000	0.165	-0.086	-0.105	-0.380	-0.183
Q8K0D5	Gfm1	0.253	0.467	0.000	0.253	-0.248	1.020	0.078	0.217
P62320	Snrpd3	0.040	0.135	0.000	0.221	-0.144	-0.306	-0.280	-0.293
P53026	Rpl10a	0.307	0.538	0.158	-0.322	0.000	0.118	-0.555	-0.840
P36993	Ppm1b	0.001	0.021	0.086	-0.228	0.000	-1.300	-1.800	-1.840
Q61035	Hars	0.035	0.123	-0.560	0.683	0.000	-1.710	-0.913	-1.280
Q8BP67	Rpl24	0.126	0.288	0.000	0.147	-0.328	0.301	0.143	0.228
Q8BQZ4	Ralgapb	0.057	0.170	-0.188	0.101	0.000	-0.179	-0.555	-0.491
Q99KN9	Clint1	0.085	0.221	0.000	-0.072	0.053	-0.466	-1.160	-0.263
Q6ZWV7	Rpl35	0.412	0.665	0.100	-0.032	0.000	0.532	-0.166	0.271
Q9QZL0	Ripk3	0.408	0.660	0.000	0.733	-0.238	0.654	-1.470	-0.577
Q62219	Tgfb1i1	0.001	0.022	-0.852	0.592	0.000	5.540	3.950	5.150
Q99M51	Nck1	0.034	0.122	0.044	0.000	-0.172	-1.730	-2.880	-0.932
Q8K157	Galm	0.001	0.018	-0.472	0.286	0.000	-2.590	-2.270	-2.830
Q6R891	Ppp1r9b	0.007	0.048	-0.300	0.179	0.000	-1.040	-1.590	-1.050
Q8VE97	Srsf4	0.080	0.211	0.065	-0.149	0.000	-0.350	-2.830	-2.070
P97868	Rbbp6	0.093	0.234	0.000	-0.157	0.004	0.164	0.016	0.278
Q9R1J0	Nsdhl	0.000	0.010	0.212	0.000	-0.154	1.860	1.730	1.980
Q9JLQ2	Git2	0.003	0.033	-0.185	0.261	0.000	-0.937	-1.420	-1.150
Q99K51	Pls3	0.002	0.027	0.000	0.943	-0.084	3.190	2.810	3.720
P54818	Galc	0.358	0.603	0.640	-0.012	0.000	0.282	-0.448	-0.145
P33609	Pola1	0.060	0.176	0.058	0.000	-0.834	-1.570	-1.450	-0.752
O70591	Pfdn2	0.026	0.103	0.000	0.302	-0.526	-0.767	-1.420	-1.340
Q60875	Arhgef2	0.124	0.284	0.011	-0.367	0.000	-0.122	-2.670	-3.400
Q66JX5	Fgfr1op	0.672	0.949	0.516	0.000	-0.404	-0.396	-2.760	1.530
P49117	Nr2c2	0.046	0.147	-0.415	0.000	0.288	-1.000	-1.560	-0.578
Q5EBH1	Rassf5	0.008	0.052	-0.317	0.000	0.413	-1.520	-2.820	-2.000
Q99N94	Mrpl9	0.093	0.234	0.142	0.000	-0.854	1.030	0.602	0.221
Q8C3X2	Ccdc90b	0.005	0.040	-0.005	0.303	0.000	1.660	1.020	1.250
P81117	Nucb2	0.002	0.027	0.376	0.000	-0.305	1.870	1.440	1.640
O54788	Dffb	0.056	0.166	0.000	1.160	-0.052	-1.010	-0.828	-0.474
Q9QY76	Vapb	0.449	0.707	0.020	0.000	-0.313	0.435	0.068	-0.237
P58404	Strn4	0.482	0.745	0.000	-0.039	0.280	-0.045	-0.554	0.248
Q9CQI7	Snrpb2	0.009	0.058	0.000	-0.133	0.059	-0.549	-0.991	-0.617
Q9CQN4	Sostdc1	0.262	0.478	-0.797	0.000	0.018	-1.030	-0.651	-0.382
Q9JKV1	Adrm1	0.232	0.436	0.000	-0.776	0.108	0.423	-4.760	-2.820
Q9JHC9	Elf2	0.083	0.216	-0.198	0.039	0.000	-0.443	-1.150	-0.367

P63087	Ppp1cc	0.072	0.197	-0.142	0.000	0.013	-0.169	-0.780	-0.518
P70371	Terf1	0.025	0.102	0.000	0.395	-0.619	-1.120	-1.050	-1.160
P46467	Vps4b	0.061	0.177	0.037	-0.210	0.000	-0.384	-1.500	-0.859
Q924A2	Cic	0.368	0.615	0.000	-0.386	0.805	-0.032	-0.150	-0.582
P55012	Slc12a2	0.024	0.099	0.000	-2.210	1.390	3.800	3.040	3.760
Q8R5A3	Apbb1ip	0.010	0.059	0.149	0.000	-0.079	-0.505	-1.010	-1.070
Q9JM90	Stap1	0.167	0.348	0.120	-0.071	0.000	-0.139	-0.977	-0.217
Q99N87	Mrps5	0.000	0.012	0.039	0.000	-0.040	1.140	0.934	0.888
Q8VDF2	Uhrf1	0.486	0.749	0.987	0.000	-0.817	0.598	-0.873	-1.370
Q91W50	Csde1	0.837	1.000	0.000	1.520	-0.584	0.018	-0.602	1.010
Q6IRU5	Cltb	0.010	0.059	0.000	-0.318	0.052	-0.644	-0.946	-0.715
Q8R3B7	Brd8	0.126	0.286	-0.614	0.116	0.000	-0.398	-2.410	-1.310
P25799	Nfkb1	0.017	0.080	0.000	-0.065	0.139	-0.430	-1.120	-0.909
Q9WTX8	Mad1l1	0.041	0.136	0.000	-0.010	0.350	-0.176	-0.603	-0.413
Q09XV5	Chd8	0.586	0.857	0.000	-1.090	0.094	-0.128	-0.357	0.228
Q923G2	Polr2h	0.104	0.252	-0.057	0.000	0.218	-0.239	-2.030	-0.901
Q61555	Fbn2	0.000	0.013	-0.574	0.551	0.000	3.680	3.550	3.750
Q921Y2	Imp3	0.195	0.387	0.000	0.390	-0.130	-0.035	-0.345	-0.201
Q8C5W3	Tbcel	0.029	0.112	0.000	0.915	-0.754	-2.390	-1.250	-2.060
Q8JZX4	Rbm17	0.033	0.121	0.000	0.276	-0.213	-0.865	-0.990	-0.361
Q9ER38	Tor3a	0.003	0.032	0.000	0.258	-0.172	0.998	0.891	1.220
P51881	Slc25a5	0.203	0.400	0.000	1.090	-0.121	0.477	1.260	1.440
O88627	Slc28a2	0.316	0.549	0.079	-0.550	0.000	-0.170	-1.540	-0.377
Q8K4L0	Ddx54	0.274	0.495	0.000	-0.315	0.208	0.291	-1.620	-0.988
Q8R1S0	Coq6	0.178	0.364	0.000	0.621	-0.148	0.936	0.515	0.414
E2JF22	Piezo1	0.024	0.098	0.000	-2.270	2.080	4.710	3.970	4.720
Q5SV85	Synrg	0.000	0.008	0.080	-0.246	0.000	5.810	5.470	6.420
Q9DBL7	Coasy	0.134	0.298	7.440	-0.978	0.000	7.090	7.330	7.010
P83870	Phf5a	0.029	0.110	-0.244	0.000	0.161	-0.640	-1.280	-0.627
Q5HZI9	Slc25a51	0.018	0.083	-0.031	0.632	0.000	1.200	0.956	1.100
Q91VC9	Ghitm	0.003	0.030	0.000	0.088	-0.147	1.130	0.700	0.953
Q8BGB5	Limd2	0.130	0.293	0.000	-0.039	0.115	-0.066	-0.503	-0.158
Q8CAK1	Iba57	0.322	0.558	0.036	0.000	-0.449	0.439	-0.205	0.176
Q4QQM4	Trp53i11	0.420	0.674	0.000	0.090	-0.068	-0.104	-0.396	0.108
Q3UFY0	Rrp36	0.228	0.431	-0.058	0.250	0.000	-0.563	-0.442	0.160
P59999	Arpc4	0.531	0.798	0.000	0.139	-0.184	0.125	0.054	-0.015
Q9D6K8	Fundc2	0.013	0.071	0.000	0.474	-0.255	1.690	1.410	0.958
Q8BRT1	Clasp2	0.206	0.402	-0.569	0.000	0.003	-0.061	-1.620	-1.150
P62827	Ran	0.003	0.031	-0.041	0.044	0.000	-0.674	-1.100	-0.771
Q9Z108	Stau1	0.006	0.046	0.086	0.000	-0.256	1.060	0.598	1.010
P28867	Prkcd	0.874	1.000	0.163	0.000	-1.170	-0.357	0.299	-1.260
Q9ET54	Palld	0.003	0.031	0.370	-0.667	0.000	2.810	2.010	2.300
O08807	Prdx4	0.256	0.470	0.038	0.000	-0.002	0.439	-0.070	0.262
Q9QZ23	Nfu1	0.022	0.094	0.000	0.235	-0.727	1.080	0.755	1.350
Q3UEB3	Puf60	0.005	0.039	0.000	-0.093	0.127	-0.739	-1.290	-0.937
Q8C4B4	Unc119b	0.448	0.706	-0.956	0.211	0.000	0.115	-0.456	-2.910
Q9JKB1	Uchl3	0.006	0.045	0.000	0.255	-0.128	-1.230	-2.330	-1.720
Q9D906	Atg7	0.013	0.070	0.028	-0.171	0.000	-0.567	-1.170	-0.790
P51175	Ppox	0.909	1.000	0.000	0.082	-0.711	0.651	-0.730	-0.373
P54775	Psmc4	0.018	0.083	-0.123	0.000	0.106	-0.391	-0.884	-0.580
Q9ES56	Trappc4	0.865	1.000	0.000	0.560	-0.421	-0.618	0.358	0.174
P51163	Uros	0.072	0.196	0.559	-0.181	0.000	-0.372	-1.170	-2.130
P11440	Cdk1	0.243	0.453	0.534	0.000	-1.540	1.200	-0.031	0.779
Q810B6	Ankfy1	0.392	0.641	-0.006	0.356	0.000	0.165	0.066	-1.070
P52019	Sqle	0.002	0.027	0.000	1.740	-0.441	5.210	5.320	6.150
Q9JHR7	Ide	0.004	0.036	0.000	0.341	-0.020	-0.764	-0.819	-0.576
Q91W53	Golga7	0.087	0.224	0.117	0.000	-0.062	0.932	0.100	0.924
Q8BUK6	Hook3	0.569	0.837	0.000	-0.177	0.034	0.322	-0.911	-0.229
O88447	Klc1	0.910	1.000	0.000	-0.262	0.051	0.542	-0.641	0.015

Q9D1P0	Mrpl13	0.522	0.787	0.000	0.348	-0.702	0.959	-0.679	0.596
Q8BU31	Rap2c	0.351	0.592	0.427	-4.150	0.000	0.508	-0.089	0.526
Q99104	Myo5a	0.027	0.107	-0.006	0.000	0.152	0.704	1.170	1.920
Q9CQ80	Vps25	0.220	0.420	0.000	0.351	-0.161	0.672	0.002	1.430
Q8K1M6	Dnm1l	0.892	1.000	0.042	0.000	-0.313	0.147	-0.185	-0.310
Q9D2R0	Aacs	0.237	0.444	1.080	0.000	-0.323	1.580	0.944	0.464
O88271	Cfdp1	0.005	0.043	0.000	-0.146	0.287	-1.330	-2.440	-2.380
P83882	Rpl36a	0.645	0.922	0.260	0.000	-0.007	0.602	-0.206	0.227
P46638	Rab11b	0.020	0.089	0.000	0.117	-0.097	-0.270	-0.495	-0.285
Q9ERG2	Strn3	0.489	0.754	1.350	-1.770	0.000	1.130	-0.551	1.530
Q6ZQL4	Wdr43	0.934	1.000	0.170	0.000	-0.109	0.209	-0.182	-0.003
P23249	Mov10	0.371	0.618	0.000	0.062	-0.014	0.456	-0.062	0.118
P00493	Hprt1	0.004	0.038	0.000	0.060	-0.208	-1.260	-1.950	-1.240
O88351	Ikbkb	0.730	1.000	0.314	-0.360	0.000	0.876	-0.396	-0.052
Q64191	Aga	0.034	0.122	0.000	0.123	-1.280	0.991	1.240	0.958
Q9D517	Agpat3	0.027	0.107	0.000	0.348	-0.252	0.753	0.547	0.673
Q8R5H1	Usp15	0.003	0.031	0.364	0.000	-0.025	-1.680	-2.580	-1.690
Q62193	Rpa2	0.053	0.161	-4.040	0.454	0.000	-5.270	-6.020	-4.510
Q8K4X7	Agpat4	0.005	0.042	0.000	0.300	-0.338	1.030	0.953	1.130
P31996	Cd68	0.988	1.000	0.659	-0.005	0.000	0.757	-0.122	0.002
Q6PIP5	Nudcd1	0.042	0.139	0.000	-0.718	0.138	-0.872	-2.330	-1.830
Q9QYS9	Qki	0.249	0.462	-0.069	0.000	0.024	-0.109	-0.787	-0.085
Q9Z2M7	Pmm2	0.001	0.021	-0.080	0.000	0.173	-1.300	-1.910	-1.880
Q9CZL5	Pcbd2	0.058	0.171	0.028	0.000	-0.711	0.373	0.407	0.453
Q9QXA5	Lsm4	0.002	0.028	0.000	-0.110	0.040	-0.957	-1.370	-0.916
Q9Z266	Snapin	0.175	0.359	0.011	0.000	-0.270	-0.152	-0.396	-0.918
P63166	Sumo1	0.032	0.119	-0.103	0.000	0.363	-0.366	-0.411	-0.342
P62806	Hist1h4a	0.776	1.000	0.000	0.764	-0.074	-0.043	-0.259	0.643
Q99KD5	Unc45a	0.428	0.684	-0.049	0.000	0.165	0.289	-0.589	-0.285
Q9CQR6	Ppp6c	0.166	0.347	0.000	0.129	-1.020	-1.250	-0.873	-0.759
Q8R4K2	Irak4	0.016	0.078	0.152	0.000	-0.178	-0.685	-1.670	-1.410
P48771	Cox7a2	0.957	1.000	-0.456	0.000	0.309	0.089	-0.409	0.125
Q8R0J7	Vps37b	0.293	0.520	0.183	-0.042	0.000	0.116	-0.393	-0.175
P47964	Rpl36	0.019	0.085	0.146	-0.148	0.000	0.783	0.357	0.578
P23298	Prkch	0.210	0.407	-0.013	0.000	0.048	-0.075	-0.813	-0.138
Q8C4Y3	Nelfb	0.269	0.488	-0.182	0.000	0.311	0.143	-1.250	-0.417
Q9CQC9	Sar1b	0.173	0.356	0.127	-0.333	0.000	-0.158	-1.010	-0.454
Q80U78	Pum1	0.834	1.000	0.000	-0.142	0.437	0.833	-0.404	-0.438
Q91ZJ5	Ugp2	0.039	0.133	0.000	0.158	-0.579	-0.976	-1.240	-0.692
Q9ER64	Osbpl5	0.463	0.721	-0.879	0.118	0.000	-0.307	-0.060	0.631
P03911	Mtnd4	0.026	0.105	-1.040	0.000	0.181	0.916	1.730	1.150
Q9EP72	Emc7	0.060	0.175	0.022	0.000	-0.211	1.430	0.205	1.070
Q9CY66	Gar1	0.549	0.818	0.000	0.455	-0.523	-0.108	-0.504	-0.073
Q7TMI3	Uhrf2	0.019	0.087	-0.049	0.200	0.000	-0.215	-0.398	-0.482
Q8R332	Nup58	0.087	0.224	0.000	0.032	-0.337	-0.313	-1.300	-0.783
Q9JL56	Gde1	0.140	0.306	-1.780	0.755	0.000	0.971	0.895	1.310
Q60767	Ly75	0.069	0.192	-0.111	0.422	0.000	-0.712	-1.810	-0.459
Q9D6L8	Ppil3	0.024	0.098	0.014	-0.545	0.000	-0.749	-0.893	-0.939
E9PZM4	Chd2	0.002	0.022	0.000	0.221	-0.104	-0.784	-0.978	-1.070
Q925J9	Med1	0.684	0.960	0.315	-0.113	0.000	0.119	-0.251	0.102
Q7TQ95	Lnp	0.459	0.719	0.255	-0.105	0.000	0.500	-0.269	0.679
O70435	Psma3	0.004	0.035	0.000	0.181	-0.215	-0.858	-1.230	-1.360
Q9QYF9	Ndrg3	0.462	0.721	0.109	-0.092	0.000	0.101	-2.550	0.247
P97427	Crmp1	0.006	0.046	-0.518	0.000	0.065	-2.230	-2.140	-1.360
P27782	Lef1	0.652	0.928	-1.410	0.000	1.930	0.644	-0.499	-1.250
Q6P9R4	Arhgef18	0.035	0.124	-0.095	0.000	0.552	-0.603	-1.540	-0.742
Q8BL74	Gtf3c2	0.500	0.765	-0.311	0.113	0.000	-0.136	-0.581	0.027
Q61207	Psap	0.175	0.359	0.324	-0.624	0.000	-0.131	-1.780	-1.130
P70404	Idh3g	0.775	1.000	0.000	0.070	-0.248	0.756	-0.504	-0.078

Q8VE92	Rbm4b	0.900	1.000	0.000	0.638	-0.243	0.413	-0.485	0.642
Q9CPU0	Glo1	0.000	0.014	-0.265	0.169	0.000	-1.470	-1.690	-1.770
Q9CYI4	Luc7l	0.092	0.233	0.092	0.000	-0.204	-0.138	-1.260	-1.150
Q9R1Q9	Atp6ap1	0.509	0.773	0.000	-3.800	1.510	2.510	-2.850	3.560
Q9D3G5	Stx11	0.059	0.174	-0.116	0.240	0.000	-0.192	-0.385	-0.238
Q6P4S8	Ints1	0.654	0.930	-0.251	0.000	0.398	-0.260	0.115	0.830
Q9WTJ4	Fiz1	0.019	0.085	-0.768	0.551	0.000	-2.790	-4.340	-2.030
Q8BYU6	Tor1aip2	0.011	0.064	-0.119	0.338	0.000	2.410	1.340	2.900
Q8VDI9	Alg9	0.565	0.834	0.000	0.345	-1.230	0.923	-0.370	-0.251
Q8R4X3	Rbm12	0.025	0.101	0.114	-0.295	0.000	-0.754	-1.930	-1.300
P97822	Anp32e	0.017	0.080	-0.110	0.199	0.000	-1.090	-1.940	-0.873
Q9D6Y7	Msra	0.068	0.189	0.549	-0.217	0.000	-0.252	-0.768	-0.814
O55013	Trappc3	0.197	0.390	-0.068	0.000	0.058	0.042	-0.786	-0.387
Q5DTM8	Rnf20	0.061	0.177	-0.043	0.000	0.212	-0.235	-0.631	-0.198
P40630	Tfam	0.889	1.000	0.483	0.000	-0.272	0.049	0.003	0.264
O35166	Gosr2	0.289	0.514	0.000	-0.790	0.687	0.295	0.517	0.710
Q8BVL3	Snx17	0.151	0.322	0.000	1.180	-1.990	1.400	1.220	1.500
Q9WVE8	Pacsin2	0.460	0.719	0.260	0.000	-0.091	0.564	-0.113	0.262
Q9CR00	Psmd9	0.000	0.011	-0.220	0.000	0.049	-1.150	-1.140	-1.140
O88508	Dnmt3a	0.201	0.397	-0.367	0.018	0.000	-0.065	-1.310	-0.720
P97386	Lig3	0.578	0.849	0.068	0.000	-0.173	0.026	-0.596	0.058
Q571H0	Urb1	0.375	0.623	0.847	0.000	-0.023	0.911	-0.805	-1.810
P57080	Usp25	0.109	0.260	0.084	-0.165	0.000	-0.069	-1.320	-1.140
P62141	Ppp1cb	0.218	0.417	0.078	-0.044	0.000	0.122	-0.514	-0.526
Q9R0P6	Sec11a	0.066	0.186	0.000	0.620	-0.132	0.704	0.707	0.867
Q8R422	Cd109	0.002	0.025	0.000	-1.010	1.720	6.180	6.070	6.910
Q9DBA9	Gtf2h1	0.488	0.752	-0.068	1.270	0.000	0.286	0.054	-0.181
P58058	Nadk	0.361	0.606	1.210	0.000	-0.691	-0.082	-0.474	-0.743
Q4VAE3	Tmem65	0.014	0.072	0.000	0.487	-0.336	1.190	1.030	1.570
Q91Z38	Ttc1	0.021	0.091	-0.290	1.550	0.000	-1.560	-1.660	-1.960
P56135	Atp5j2	0.005	0.040	0.000	0.393	-0.087	0.924	1.130	0.968
Q6P9Q6	Fkbp15	0.205	0.402	0.000	0.346	-0.021	0.306	-1.450	-0.962
Q8C0D5	Efl1	0.065	0.184	0.000	0.237	-0.014	-0.080	-0.766	-0.729
Q8R1N0	Znf830	0.022	0.093	-0.006	0.000	0.395	-0.705	-1.570	-0.748
Q62348	Tsn	0.000	0.003	-0.097	0.000	0.053	-1.470	-1.540	-1.490
Q6PE01	Snrnp40	0.016	0.078	0.000	0.172	-0.032	-0.475	-0.884	-0.420
Q9DCM2	Gstk1	0.701	0.977	0.010	0.000	-0.212	0.377	-0.571	-0.378
Q9BCZ4	Vimp	0.732	1.000	-0.386	0.179	0.000	0.206	-0.445	-0.249
P02469	Lamb1	0.000	0.010	0.000	-0.970	0.049	4.590	4.580	4.770
Q9QUM4	Slamf1	0.023	0.096	0.042	-0.099	0.000	-0.350	-0.725	-0.965
Q9D0C4	Trmt5	0.109	0.260	-0.090	0.320	0.000	-0.850	-4.670	-1.540
Q8K4D3	Slc36a1	0.353	0.594	0.603	0.000	-0.040	0.838	0.183	0.432
Q8BGZ4	Cdc23	0.969	1.000	-0.048	0.000	0.477	0.290	-0.073	0.187
Q8BYA0	Tbcd	0.073	0.198	0.208	-0.046	0.000	-0.191	-1.560	-1.040
Q9CU65	Zmym2	0.023	0.097	0.139	0.000	-0.153	-0.563	-1.080	-0.512
Q9D6N5	Drap1	0.026	0.104	0.000	-0.142	0.076	-0.451	-1.270	-0.887
P70268	Pkn1	0.139	0.305	0.296	-0.083	0.000	0.064	-1.320	-0.873
Q8K268	Abcf3	0.662	0.938	0.000	0.314	-0.079	0.491	-0.526	-0.185
P18181	Cd48	0.140	0.306	-0.550	0.111	0.000	-1.040	-0.896	-0.252
O35704	Sptlc1	0.322	0.557	0.000	0.610	-0.610	1.960	-0.450	1.180
Q60848	Hells	0.176	0.360	0.617	-0.364	0.000	0.051	-1.150	-0.971
Q91VU0	Fam3c	0.497	0.763	-0.324	0.000	0.112	0.324	-0.226	0.155
Q80UP3	Dgkz	0.013	0.070	0.041	0.000	-0.034	-0.490	-1.080	-0.681
Q9CWK3	Cd2bp2	0.003	0.030	0.000	-0.074	0.360	-0.959	-1.200	-0.874
O35954	Pitpnm1	0.016	0.079	-0.371	0.600	0.000	-0.920	-1.220	-1.360
P52432	Polr1c	0.009	0.058	0.073	-0.049	0.000	-0.297	-0.468	-0.254
E9QAM5	Helz2	0.272	0.492	0.165	0.000	-0.002	0.296	-0.785	-0.678
Q9R117	Tyk2	0.498	0.763	-0.303	1.680	0.000	1.680	-1.290	5.770
O09126	Sema4d	0.389	0.638	0.000	-0.504	0.016	-0.443	-0.502	-0.137

Q8C5L6	Inpp5k	0.406	0.658	0.323	-0.057	0.000	0.022	-0.757	0.149
Q9D1M4	Eef1e1	0.658	0.934	0.000	0.081	-0.123	0.368	-0.741	-0.135
P08556	Nras	0.188	0.378	-0.332	0.239	0.000	0.065	0.343	0.475
Q8R3C0	Mcmbp	0.017	0.081	0.000	0.437	-0.374	-0.773	-1.280	-1.210
P16332	Mut	0.015	0.076	0.000	0.403	-0.314	1.300	0.791	1.140
P98195	Atp9b	0.441	0.697	0.290	0.000	-1.440	0.627	-0.514	0.370
Q9WU84	Ccs	0.004	0.036	-0.087	0.000	0.263	-0.735	-1.230	-1.050
Q8BG15	Ctdspl2	0.161	0.339	0.158	0.000	0.000	-0.044	-1.070	-0.326
Q7TNP2	Ppp2r1b	0.648	0.925	0.000	-1.640	0.062	-0.091	-1.450	-1.050
Q9QZH3	Ppie	0.006	0.045	-0.384	0.000	0.099	-1.000	-1.100	-0.840
Q9D0B6	Pbdc1	0.040	0.134	-0.101	0.317	0.000	-0.847	-1.480	-0.430
Q8VDD8	Wash1	0.001	0.014	0.000	-0.181	0.148	-2.180	-2.970	-2.460
Q9CWF2	Tubb2b	0.013	0.070	-1.480	0.205	0.000	2.340	1.630	2.050
Q924L1	Letmd1	0.672	0.950	-1.210	0.847	0.000	-1.630	0.966	-1.040
P58871	Tnks1bp1	0.036	0.125	0.000	0.067	-0.956	0.608	0.849	0.825
Q9JLR1	Sec61a2	0.001	0.015	0.743	-0.345	0.000	3.980	3.380	3.910
Q8JZR0	Acsl5	0.110	0.261	-0.081	0.000	0.085	0.658	0.086	0.323
Q80UU2	Rpp38	0.005	0.039	0.284	0.000	-0.181	-0.693	-0.891	-0.837
Q9D8S4	Rexo2	0.107	0.257	-0.191	0.000	0.052	0.356	0.007	0.431
Q7TSQ8	Pdpr	0.166	0.347	0.000	0.234	-0.035	1.070	0.066	0.599
Q99LD4	Gps1	0.116	0.271	0.000	-0.608	0.075	-0.392	-2.690	-1.650
Q9D7B6	Acad8	0.170	0.353	0.000	-1.970	0.001	1.380	0.114	0.357
Q9D2E2	Toe1	0.038	0.129	-0.230	0.099	0.000	-0.498	-1.300	-0.711
Q8BGK6	Slc7a6	0.020	0.088	0.473	0.000	-0.733	1.870	1.030	1.780
Q3U319	Rnf40	0.939	1.000	-0.282	0.018	0.000	-0.232	-0.673	0.745
Q8BWW9	Pkn2	0.014	0.073	0.067	-0.265	0.000	1.650	0.718	1.050
Q69ZX6	Morc2a	0.031	0.116	0.000	0.070	-0.068	-0.314	-1.090	-0.935
Q8BMG7	Rab3gap2	0.815	1.000	0.974	0.000	-2.280	-0.344	-1.730	-0.051
P52332	Jak1	0.177	0.363	0.099	-0.263	0.000	0.484	0.026	0.174
Q8K370	Acad10	0.053	0.162	0.324	0.000	-0.339	0.796	0.651	0.382
Q9ERG0	Lima1	0.016	0.078	-1.030	0.000	1.290	3.320	2.760	2.660
P70677	Casp3	0.003	0.030	-0.016	0.138	0.000	-1.190	-2.040	-1.570
P70303	Ctps2	0.915	1.000	-1.140	0.507	0.000	-0.358	-0.806	0.335
Q8C079	Strip1	0.020	0.089	0.000	0.023	-0.001	-0.227	-0.556	-0.309
O35963	Rab33b	0.738	1.000	0.000	0.257	-0.073	0.208	-0.033	0.142
Q91V01	Lpcat3	0.000	0.014	0.081	0.000	-0.276	1.120	1.080	1.090
Q3U308	Ctu2	0.630	0.906	-0.090	0.000	0.201	0.290	-0.324	-0.173
Q9R0N0	Galk1	0.017	0.082	0.220	0.000	-0.038	-0.664	-1.340	-0.646
Q6TEK5	Vkorc1l1	0.114	0.268	0.438	0.000	-0.745	0.971	0.277	1.730
P51612	Xpc	0.055	0.166	-0.178	0.000	0.301	-0.793	-1.460	-0.385
Q8VI93	Oas3	0.907	1.000	-1.180	1.910	0.000	-0.757	1.090	0.796
Q9R0L7	Akap8l	0.946	1.000	-0.713	0.000	5.100	1.220	-0.563	3.270
A2ALW5	Dnajc25	0.089	0.227	0.000	0.099	-0.136	0.114	1.400	1.090
Q8BX80	Engase	0.040	0.135	0.000	0.000	-0.421	-0.479	-1.130	-0.991
O35345	Kpna6	0.521	0.787	0.000	0.117	-0.022	0.282	-0.333	-0.277
Q6P9R2	Oxsr1	0.403	0.653	0.000	-0.051	0.401	0.108	-0.789	0.103
Q8BX70	Vps13c	0.314	0.547	-0.587	4.430	0.000	-1.540	-0.507	0.164
Q9D2X5	Mau2	0.343	0.582	1.700	-0.299	0.000	0.721	-2.370	-0.480
Q9CQZ6	Ndufb3	0.002	0.025	-0.037	0.054	0.000	0.800	0.535	0.580
Q8VEH8	Erlec1	0.003	0.030	-0.264	0.815	0.000	2.390	2.290	2.460
Q9DBS9	Osbpl3	0.028	0.108	-0.747	0.622	0.000	-1.900	-2.520	-1.220
Q9ET22	Dpp7	0.022	0.093	0.280	-0.651	0.000	1.120	0.745	1.040
Q7TT00	Supt20h	0.529	0.796	-0.259	0.000	0.989	-0.214	-1.420	0.814
P55264	Adk	0.048	0.152	0.000	-0.071	0.107	-0.916	-2.270	-0.795
Q99J45	Nrbp1	0.236	0.444	0.000	0.419	-0.402	0.398	-1.600	-2.240
Q99MD9	Nasp	0.010	0.058	0.000	0.025	-0.302	-0.880	-1.720	-1.430
Q61183	Papola	0.003	0.031	-0.236	0.000	0.015	-2.470	-1.820	-3.070
Q9JK38	Gnpnat1	0.020	0.087	-0.428	0.206	0.000	-1.870	-4.330	-2.580
Q9EQN3	Tsc22d4	0.102	0.247	-0.175	0.000	0.444	-0.414	-1.580	-0.445

Q64523	Hist2h2ac	0.516	0.779	0.000	-4.270	2.780	1.090	-7.740	-1.910
Q6GSS7	Hist2h2aa1	0.516	0.779	0.000	-4.270	2.780	1.090	-7.740	-1.910
Q8C5L7	Rbm34	0.393	0.641	0.000	-0.513	0.047	-0.005	-1.680	-0.339
Q61164	Ctcf	0.396	0.646	0.000	-0.032	0.363	0.254	-1.530	-0.016
Q9EQW7	Kif13a	0.113	0.266	0.000	-0.297	0.102	-0.141	-3.210	-2.580
P45878	Fkbp2	0.153	0.325	0.467	-0.204	0.000	0.711	0.217	0.684
P05063	Aldoc	0.181	0.368	0.123	-0.392	0.000	-0.459	-0.168	-0.728
Q08122	Tle3	0.005	0.040	-0.384	0.000	0.107	-1.320	-2.140	-1.610
Q8VEJ4	Nle1	0.656	0.932	0.581	0.000	-0.057	0.782	-1.120	0.012
Q9D6M3	Slc25a22	0.001	0.016	0.000	0.088	-0.280	1.510	1.170	1.410
Q6PGB6	Naa50	0.083	0.216	0.000	1.470	-0.508	-1.190	-2.400	-0.760
Q9ERE7	Mesdc2	0.075	0.203	0.218	-0.024	0.000	0.875	0.330	0.366
P97808	Fxyd5	0.556	0.825	0.040	-0.208	0.000	0.153	-0.912	-0.054
Q9D2R6	Coa3	0.017	0.080	0.000	0.304	-0.248	1.120	0.611	0.904
Q6PDK2	Kmt2d	0.835	1.000	-0.075	0.000	0.729	0.995	-3.110	1.750
O35955	Psmb10	0.003	0.033	-0.486	0.139	0.000	-1.520	-1.850	-1.380
Q9D666	Sun1	0.011	0.064	-0.159	0.054	0.000	0.644	0.375	0.368
Q69ZQ2	Isy1	0.308	0.540	0.000	0.024	-0.086	-0.353	-4.570	-0.172
Q9CQM9	Glrx3	0.670	0.948	0.000	0.737	-0.831	0.182	-0.361	-0.620
Q3UHD9	Agap2	0.512	0.776	0.000	-1.590	2.090	1.140	0.229	1.570
P62869	Tceb2	0.005	0.040	-0.160	0.000	0.361	-0.924	-1.230	-0.867
Q8VE18	Smg8	0.067	0.186	0.000	-0.252	0.155	-0.327	-0.869	-0.432
P59997	Kdm2a	0.046	0.148	-0.142	0.000	0.321	-0.327	-0.528	-0.298
Q5U4D9	Thoc6	0.253	0.467	-0.007	0.107	0.000	0.133	-0.430	-0.299
Q61790	Lag3	0.676	0.952	-1.220	1.200	0.000	-1.810	-0.508	0.885
Q8BMA6	Srp68	0.366	0.613	0.286	-0.009	0.000	0.374	-0.726	-0.412
P47934	Crat	0.395	0.644	0.000	-3.600	0.145	2.180	-1.640	0.743
Q9R0M6	Rab9a	0.599	0.872	0.489	-0.023	0.000	1.030	-0.266	0.401
Q91WK1	Spryd4	0.045	0.146	0.000	0.231	-0.354	0.951	0.398	0.566
Q9CQE1	Nipsnap3b	0.179	0.365	0.000	0.874	-0.677	0.769	0.963	0.692
Q9WVD5	Slc25a15	0.005	0.042	-0.508	0.364	0.000	1.270	1.750	1.660
Q8BFU2	Hist3h2a	0.777	1.000	0.000	-4.410	1.560	0.303	-3.670	-1.420
Q8CGP5	Hist1h2af	0.777	1.000	0.000	-4.410	1.560	0.303	-3.670	-1.420
Q8CGP7	Hist1h2ak	0.777	1.000	0.000	-4.410	1.560	0.303	-3.670	-1.420
Q6DID3	Scaf8	0.074	0.200	-0.313	0.005	0.000	-0.483	-1.190	-0.490
Q4FZC9	Syne3	0.324	0.559	0.000	-0.003	0.647	0.218	-0.099	-0.456
Q7TQK4	Exosc3	0.740	1.000	0.915	0.000	-1.500	0.287	-2.560	0.408
Q3U3R4	Lmf1	0.048	0.151	0.000	-1.190	0.317	1.070	0.891	1.090
Q8VCS3	Fam20b	0.046	0.148	-0.069	0.014	0.000	0.362	0.123	0.555
P63328	Ppp3ca	0.969	1.000	0.252	-1.670	0.000	-0.681	-0.504	-0.307
Q99LG2	Tnpo2	0.144	0.311	-0.517	0.405	0.000	-0.599	-0.596	-0.407
P34902	Il2rg	0.090	0.230	-0.469	0.000	0.113	-0.901	-0.440	-0.531
O70422	Gtf2h4	0.073	0.198	0.000	-0.232	0.120	-0.256	-0.742	-0.400
Q9R059	Fhl3	0.007	0.049	0.173	-0.055	0.000	1.130	0.644	0.797
Q99N69	Lpxn	0.148	0.318	-1.040	0.000	0.363	-0.818	-1.570	-0.895
Q921E6	Eed	0.268	0.487	0.000	-0.754	0.004	-0.297	-1.060	-0.684
Q8BVW3	Trim14	0.019	0.086	-0.022	0.118	0.000	-0.230	-0.342	-0.147
Q5SU73	Coil	0.142	0.309	-0.437	0.214	0.000	-0.490	-1.940	-0.572
P97360	Etv6	0.033	0.119	0.065	-0.481	0.000	-0.573	-0.940	-1.020
Q8BFR4	Gns	0.030	0.113	0.302	-0.538	0.000	1.030	0.604	0.845
Q91Z53	Grhpr	0.060	0.176	0.000	-0.087	0.077	-0.125	-0.652	-0.656
P11688	Itga5	0.002	0.028	1.160	-0.159	0.000	3.840	3.150	3.570
P97494	Gclc	0.814	1.000	0.000	-3.610	0.176	-1.950	-2.290	-0.243
Q9DCC4	Pycrl	0.014	0.073	-0.043	0.149	0.000	-0.894	-0.823	-0.369
Q9WTK3	Gpaa1	0.096	0.239	0.000	0.450	-0.747	0.795	0.416	1.080
Q61165	Slc9a1	0.476	0.736	0.000	3.350	-0.438	1.690	2.590	1.560
P35285	Rab22a	0.166	0.347	0.077	-0.430	0.000	-0.291	-0.396	-0.563
O08759	Ube3a	0.729	1.000	-1.650	0.000	0.109	-0.266	-1.150	-0.824
P55937	Golga3	0.555	0.824	0.743	-0.155	0.000	0.761	-0.700	-0.507

Q8CGF7	Tcerg1	0.003	0.030	0.000	-0.052	0.036	-0.456	-0.710	-0.493
Q8R0A0	Gtf2f2	0.054	0.164	0.000	0.383	-0.040	-0.234	-1.020	-0.550
Q69ZR2	Hectd1	0.209	0.406	0.000	-0.236	0.376	0.342	-1.490	-2.030
P01887	B2m	0.002	0.024	-0.085	0.041	0.000	-0.362	-0.494	-0.399
Q3TZX8	Nol9	0.039	0.134	0.000	0.630	-0.031	-0.350	-0.702	-0.502
P15864	Hist1h1c	0.098	0.241	0.000	-0.140	0.151	-0.276	-0.471	-0.109
P70218	Map4k1	0.001	0.015	-0.001	0.000	0.020	-0.901	-1.290	-1.110
O55142	Rpl35a	0.064	0.183	0.103	0.000	-0.035	0.678	0.149	0.445
Q9D7H3	RtcA	0.331	0.569	0.000	0.509	-0.242	-0.137	-0.464	0.003
O08585	Clta	0.023	0.095	0.196	0.000	-0.151	-0.400	-0.895	-0.573
Q00651	Itga4	0.060	0.176	-0.013	0.000	0.220	-0.247	-0.705	-0.212
Q9JHQ5	Lztfl1	0.249	0.461	0.000	1.550	-1.860	-0.883	-2.190	-1.500
Q8CI33	Cwf19l1	0.057	0.168	0.000	-0.338	0.204	-0.426	-1.560	-1.040
Q9DBD5	Pelp1	0.585	0.857	0.000	0.295	-0.055	0.379	-0.345	-0.239
Q8BPB0	Mob1b	0.065	0.184	0.002	-0.022	0.000	-1.070	-0.971	-0.160
Q921Y0	Mob1a	0.065	0.184	0.002	-0.022	0.000	-1.070	-0.971	-0.160
Q91VJ4	Stk38	0.126	0.288	0.000	0.140	-0.080	-0.206	-1.220	-0.366
Q91VA6	Poldip2	0.121	0.278	0.000	0.211	-1.280	1.200	0.544	0.335
Q8R307	Vps18	0.451	0.708	0.576	0.000	-2.330	-5.690	0.344	-1.420
Q9Z1P6	Ndufa7	0.004	0.037	-0.044	0.000	0.010	1.000	0.554	0.732
O35492	Clk3	0.094	0.236	0.396	0.000	-0.283	-0.169	-1.650	-1.240
Q9CQY5	Magt1	0.003	0.030	0.204	0.000	-0.242	1.620	1.090	1.260
Q80UK8	Ints2	0.770	1.000	0.000	0.667	-0.462	0.070	-0.378	0.167
Q9JJA7	Ccnl2	0.068	0.188	-0.146	0.098	0.000	-1.860	-1.480	-4.980
Q91VT1	Nsmce2	0.108	0.258	0.022	0.000	-0.060	-0.181	-1.100	-0.457
Q9QZA0	Ca5b	0.641	0.917	-0.511	1.310	0.000	0.880	0.859	-3.100
P53798	Fdft1	0.001	0.020	0.237	-0.533	0.000	3.080	2.240	2.750
Q8CGC6	Rbm28	0.512	0.776	0.000	0.234	-0.130	0.198	-0.353	-0.157
Q91XC9	Pex16	0.174	0.359	0.000	0.199	-0.204	-0.082	-0.321	-0.286
Q8BJU0	Sgta	0.034	0.122	0.181	-0.136	0.000	-0.501	-1.020	-0.422
Q80YQ2	Med23	0.492	0.756	-0.693	0.000	0.423	-1.010	-4.040	1.240
P58281	Opa1	0.111	0.263	-0.010	0.052	0.000	-0.089	-0.563	-0.194
Q9D1K2	Atp6v1f	0.511	0.776	0.318	0.000	-0.373	1.120	-0.116	-0.082
Q9QY36	Naa10	0.267	0.486	-0.260	0.090	0.000	-0.029	-1.070	-0.333
Q03526	Itk	0.005	0.041	0.620	-0.355	0.000	-2.150	-1.480	-1.840
Q9D0L8	Rnmt	0.050	0.155	-0.425	0.000	0.004	-0.704	-1.880	-1.010
Q8VDT9	Mrpl50	0.179	0.365	0.195	-2.670	0.000	0.890	0.464	0.716
Q6ZWY3	Rps27l	0.220	0.419	0.023	-0.195	0.000	0.934	-0.035	0.227
P70700	Polr1b	0.494	0.759	-2.100	0.525	0.000	-0.634	-1.670	-1.200
A2RSY6	Trmt1l	0.025	0.102	0.000	0.306	-0.067	-0.295	-0.736	-0.522
Q8K215	Lyrm4	0.019	0.086	0.000	0.224	-0.231	0.718	0.439	0.602
Q8VE11	Mtmr6	0.246	0.457	0.271	-0.215	0.000	0.096	-0.464	-0.672
P35601	Rfc1	0.381	0.631	0.361	-0.017	0.000	0.169	-0.405	-0.034
P68134	Acta1	0.003	0.033	0.940	-1.600	0.000	5.180	4.230	4.750
Q8BTV2	Cpsf7	0.081	0.213	0.276	-0.116	0.000	-0.122	-0.689	-0.432
Q9R1P0	Psma4	0.005	0.039	0.000	-0.149	0.029	-0.825	-1.490	-1.300
Q6NXI6	Rprd2	0.317	0.551	-1.120	0.219	0.000	-2.430	-0.277	-0.826
O88958	Gnpda1	0.039	0.133	-0.415	0.245	0.000	-1.350	-1.430	-0.531
P97814	Pstpip1	0.021	0.091	0.000	-0.470	0.035	-0.725	-1.330	-1.030
Q9CR20	Ier3ip1	0.008	0.054	-0.005	0.000	0.262	1.340	0.984	0.751
P61963	Dcaf7	0.926	1.000	-0.608	0.000	0.679	-0.850	-0.695	1.910
Q9JLZ3	Auh	0.243	0.453	0.028	0.000	-0.092	0.540	-0.170	0.570
Q9DBC3	Cmtr1	0.022	0.093	-0.160	0.062	0.000	-0.408	-0.824	-0.464
Q8BGS7	Cept1	0.152	0.323	-0.085	0.000	0.517	0.219	1.040	0.812
Q9JLV1	Bag3	0.004	0.038	-0.462	0.056	0.000	1.980	1.160	1.520
Q61462	Cyba	0.727	1.000	0.833	-0.144	0.000	0.806	-0.516	-0.163
Q8BGA9	Oxa1l	0.039	0.133	0.000	-1.550	0.191	1.400	1.020	1.330
O88286	Wiz	0.633	0.909	0.000	-0.611	0.289	-0.058	-1.210	0.168
Q91WK2	Eif3h	0.199	0.394	0.008	0.000	-0.211	-0.065	-0.983	-0.428

Q9CZE3	Rab32	0.582	0.853	0.695	0.000	-0.235	0.973	0.064	0.146
Q3UMW8	Cln5	0.192	0.384	0.765	-0.088	0.000	0.123	-0.679	-0.468
Q8BUY5	Timmdc1	0.012	0.067	0.000	0.616	-0.099	1.080	1.290	1.470
B2RRE7	Otud4	0.049	0.154	0.191	-0.090	0.000	-0.294	-1.270	-0.795
Q62018	Ctr9	0.075	0.202	0.000	0.236	-0.610	0.292	0.707	0.680
Q9CZV5	Supt7l	0.575	0.845	-0.216	0.417	0.000	-1.000	0.236	0.159
Q9EQ06	Hsd17b11	0.029	0.111	0.144	0.000	-0.254	0.493	0.301	0.386
Q9ERB0	Snap29	0.440	0.697	0.870	-3.570	0.000	0.006	0.284	0.520
Q9WV70	Noc2l	0.688	0.964	1.540	-3.470	0.000	0.186	-0.279	-4.600
P18572	Bsg	0.144	0.311	0.286	0.000	-0.498	1.030	0.217	0.378
Q8R5L3	Vps39	0.681	0.957	0.177	0.000	-0.224	0.208	-0.321	-0.195
Q99JX3	Gorasp2	0.318	0.551	0.543	-0.132	0.000	-0.070	-1.110	0.110
Q7TN29	Smap2	0.490	0.754	0.000	0.100	-0.152	-0.023	-1.170	0.172
P33215	Nedd1	0.164	0.344	-0.152	0.000	0.093	0.011	-0.730	-0.524
O08528	Hk2	0.670	0.948	0.751	0.000	-0.383	0.849	-0.130	0.255
Q8BZ21	Kat6a	0.030	0.114	-0.637	0.000	0.235	-0.967	-1.210	-1.830
O35218	Cpsf2	0.010	0.060	-0.237	0.000	0.049	-0.570	-0.956	-1.070
O09000	Ncoa3	0.012	0.067	-0.143	0.000	0.327	-0.566	-1.070	-0.840
Q8K394	Plcl2	0.265	0.483	-1.200	0.866	0.000	-2.160	0.368	-3.650
Q9QYF1	Rdh11	0.005	0.043	-0.299	0.000	0.105	1.200	1.590	0.889
Q68FG3	Spty2d1	0.592	0.864	0.222	0.000	-0.215	0.424	0.089	-1.650
O54918	Bcl2l11	0.580	0.851	0.000	0.199	-0.024	0.457	-0.129	0.176
P70279	Surf6	0.040	0.135	-0.194	0.000	0.050	-0.442	-1.100	-0.554
P10853	Hist1h2bf	0.891	1.000	0.000	1.230	-0.393	-0.370	-0.110	1.030
P10854	Hist1h2bm	0.891	1.000	0.000	1.230	-0.393	-0.370	-0.110	1.030
Q64475	Hist1h2bb	0.891	1.000	0.000	1.230	-0.393	-0.370	-0.110	1.030
Q64478	Hist1h2bh	0.891	1.000	0.000	1.230	-0.393	-0.370	-0.110	1.030
Q64525	Hist2h2bb	0.891	1.000	0.000	1.230	-0.393	-0.370	-0.110	1.030
Q6ZWY9	Hist1h2bc	0.891	1.000	0.000	1.230	-0.393	-0.370	-0.110	1.030
Q8CGP2	Hist1h2bp	0.891	1.000	0.000	1.230	-0.393	-0.370	-0.110	1.030
Q3U4G3	Xxylt1	0.129	0.292	-3.840	0.000	0.665	2.220	0.974	1.950
Q9D8Y1	Tmem126a	0.000	0.011	0.000	0.009	-0.058	1.380	1.100	1.350
Q8VE10	Naa40	0.971	1.000	0.406	-0.674	0.000	0.640	-0.660	-0.192
P29352	Ptpn22	0.929	1.000	-0.876	0.000	0.428	0.287	-0.538	-0.326
Q9D067	Mdm1	0.213	0.409	-0.765	0.076	0.000	-1.780	-1.030	-0.213
Q3TL44	Nlrx1	0.072	0.196	0.000	0.222	-0.158	0.479	0.242	0.303
P61202	Cops2	0.045	0.146	0.034	-0.233	0.000	-0.510	-1.110	-0.490
Q9CZN8	Qrsl1	0.705	0.982	0.000	0.121	-0.527	0.510	-0.341	-0.174
Q9WUD1	Stub1	0.453	0.711	0.263	0.000	-0.427	-2.900	0.114	0.084
Q9CQC6	Bzw1	0.586	0.858	0.316	0.000	-0.007	0.514	-0.502	-0.281
P27577	Ets1	0.124	0.284	0.000	-0.224	0.369	-0.182	-1.230	-0.507
O35239	Ptpn9	0.099	0.243	1.850	0.000	-0.292	1.650	2.830	1.930
Q8BTY2	Slc4a7	0.880	1.000	-1.360	0.085	0.000	1.850	-2.210	-0.310
P62311	Lsm3	0.001	0.015	-0.195	0.357	0.000	-1.830	-1.570	-1.770
Q8CI95	Osbpl11	0.208	0.404	0.301	-0.146	0.000	0.187	-3.070	-1.250
Q9JMG1	Edf1	0.286	0.511	-0.171	0.000	0.306	-0.570	-0.968	0.252
Q91VN4	Chchd6	0.012	0.068	0.000	0.253	-1.110	1.520	1.500	1.590
Q9ESJ0	Xpo4	0.053	0.162	-0.029	0.000	0.040	-0.345	-1.510	-0.877
Q8BSU7	Mob3a	0.407	0.658	0.000	0.097	-2.220	-0.040	-0.204	0.256
Q6S5J6	Krit1	0.383	0.632	-0.145	0.136	0.000	0.151	-5.620	-0.086
Q61211	Eif2d	0.125	0.285	0.228	-0.376	0.000	-0.314	-1.080	-0.460
Q9WTS2	Fut8	0.130	0.293	0.405	0.000	-0.479	0.706	0.666	0.242
Q78IS1	Tmed3	0.014	0.074	0.000	0.223	-1.510	1.870	1.690	2.000
Q8R079	Bfar	0.008	0.054	0.000	0.725	-0.217	1.700	1.460	1.620
Q8VEB4	Pla2g15	0.315	0.549	0.547	0.000	-0.266	0.162	-0.645	-0.399
Q9WU56	Pus1	0.903	1.000	2.900	-0.702	0.000	2.620	0.251	-1.290
Q6PHN9	Rab35	0.343	0.583	0.000	0.085	-0.300	0.045	-0.496	-0.430
Q8K4I3	Arhgef6	0.010	0.058	-0.259	0.000	0.183	-0.812	-1.510	-1.100
Q91ZN5	Slc35b2	0.050	0.156	0.290	-0.058	0.000	0.603	0.284	0.611

Q9CQT2	Rbm7	0.006	0.045	0.000	0.373	-0.119	-1.020	-0.864	-0.687
B2RWS6	Ep300	0.670	0.948	0.000	1.170	-0.194	0.000	0.248	0.134
O35904	Pik3cd	0.505	0.769	0.000	-0.553	0.026	-0.185	-0.997	-0.099
P53569	Cebpz	0.047	0.150	-0.954	0.335	0.000	-1.100	-1.420	-1.770
Q71RI9	Kyat3	0.016	0.079	0.000	0.041	-0.711	0.821	0.680	0.805
O88587	Comt	0.014	0.072	-0.457	0.392	0.000	1.030	0.947	1.110
Q3U3I9	Znf865	0.618	0.892	-0.629	0.000	0.065	-0.096	-1.410	0.093
O55187	Cbx4	0.330	0.567	-0.691	0.501	0.000	-0.719	-0.528	-0.198
Q03958	Pfdn6	0.085	0.220	0.000	1.140	-1.340	-2.000	-2.560	-1.220
P50295	Nat2	0.967	1.000	-0.476	1.580	0.000	-0.090	1.210	0.073
Q91YJ2	Snx4	0.196	0.389	-2.760	1.530	0.000	2.230	0.355	3.230
P10820	Prf1	0.674	0.951	-2.310	0.297	0.000	-1.810	-1.130	-0.338
Q8CGZ0	Cherp	0.085	0.220	-0.172	0.165	0.000	-0.178	-0.463	-0.254
Q3UFM5	Nom1	0.004	0.036	0.326	0.000	-0.274	-1.150	-1.300	-0.992
Q9D338	Mrpl19	0.721	1.000	0.000	-0.168	0.031	1.100	-0.582	-0.080
Q8R3Q0	Saraf	0.011	0.063	0.000	-0.070	0.063	0.953	0.424	0.822
Q8BIP0	Dars2	0.009	0.056	0.571	-0.292	0.000	1.550	1.440	2.180
Q9Z1G3	Atp6v1c1	0.811	1.000	0.655	-0.150	0.000	0.660	-0.397	-0.062
P63085	Mapk1	0.087	0.224	0.263	0.000	-0.001	-0.072	-0.496	-0.204
Q6DID7	Wls	0.003	0.031	0.000	0.433	-0.720	2.130	2.080	2.410
A2A4P0	Dhx8	0.062	0.178	0.141	-0.124	0.000	-0.336	-1.520	-0.833
Q3TB48	Tmem104	0.184	0.373	0.520	-0.340	0.000	0.970	0.323	0.427
A3KMP2	Ttc38	0.005	0.039	-0.216	0.000	0.035	-1.560	-2.530	-1.630
Q60974	Ncor1	0.231	0.436	-0.323	0.025	0.000	-0.567	-1.790	-0.112
Q60855	Ripk1	0.652	0.929	0.790	0.000	-0.944	0.818	-1.660	-0.584
Q810V0	Mphosph10	0.560	0.829	0.000	-0.034	0.392	0.108	-0.121	0.077
P42128	Foxk1	0.148	0.318	-0.996	0.000	0.660	-1.930	-3.160	-0.337
P39688	Fyn	0.582	0.853	-0.633	0.103	0.000	-0.287	-0.529	-0.169
O88842	Fgd3	0.002	0.024	0.129	0.000	-0.012	-1.240	-1.930	-1.440
Q99LB2	Dhrs4	0.046	0.147	0.000	0.173	-0.424	1.020	0.369	0.601
Q8C5H8	Nadk2	0.914	1.000	0.228	0.000	-0.004	0.494	-0.258	-0.095
Q99JP7	Ggt7	0.000	0.008	-0.382	0.079	0.000	3.770	3.880	4.330
O54901	Cd200	0.061	0.176	0.000	-0.070	0.296	-0.290	-0.577	-0.177
Q8R0H9	Gga1	0.483	0.746	0.000	-0.245	1.100	0.656	-5.080	0.722
Q9CYD3	Crtap	0.001	0.019	0.000	1.040	-0.922	5.360	4.890	5.340
Q9DBU0	Tm9sf1	0.008	0.051	0.000	0.173	-0.179	0.623	0.620	0.936
A2A5R2	Arfgef2	0.338	0.577	0.091	0.000	-0.236	0.211	-0.703	-0.690
Q9DBB4	Naa16	0.660	0.936	0.000	1.260	-0.821	0.296	1.320	-0.120
Q8CJG1	Ago1	0.840	1.000	-0.612	1.730	0.000	0.802	1.310	-0.427
Q3TAA7	Stk11ip	0.010	0.061	0.014	-0.019	0.000	-0.701	-1.570	-1.160
Q61749	Eif2b4	0.245	0.456	0.000	0.607	-0.066	-0.100	-0.030	-1.620
Q9JJ80	Rpf2	0.464	0.722	0.000	2.000	-0.260	0.242	-1.260	0.554
Q6VN19	Ranbp10	0.266	0.485	0.000	-1.080	0.050	0.796	0.177	-0.191
Q64700	Rbl2	0.364	0.611	-0.325	0.595	0.000	-0.538	-0.970	0.337
Q64697	Ptprcap	0.230	0.434	-0.165	0.189	0.000	0.135	0.105	0.266
O88738	Birc6	0.391	0.640	0.401	0.000	-0.399	0.604	-1.420	-1.120
Q9QXK7	Cpsf3	0.058	0.171	-0.218	0.098	0.000	-0.222	-0.525	-0.385
O35975	Art2b	0.057	0.170	0.000	-0.256	0.901	-1.100	-1.580	-0.463
Q9R0Q7	Ptges3	0.026	0.105	-0.221	0.085	0.000	-2.030	-0.845	-1.090
O70496	Clcn7	0.194	0.386	0.493	-0.414	0.000	0.741	0.292	0.423
Q9CQE3	Mrps17	0.067	0.187	0.000	1.020	-0.199	0.968	1.420	1.540
Q8BPS4	Gpr180	0.028	0.107	0.000	-3.310	0.570	3.540	2.880	3.280
Q80TP3	Ubr5	0.617	0.891	0.000	-1.080	0.287	0.572	-1.580	2.050
Q9JKL4	Ndufaf3	0.124	0.284	0.000	-1.040	0.623	0.737	0.694	1.040
Q8BTY1	Kyat1	0.005	0.039	-0.231	0.533	0.000	-1.400	-2.010	-2.310
Q8VD04	Gripap1	0.146	0.315	0.000	0.152	-0.449	-0.246	-1.500	-0.746
Q8BHC4	Dcakd	0.017	0.081	0.466	0.000	-1.560	2.210	1.810	2.370
Q61553	Fscn1	0.003	0.030	0.000	-0.057	0.058	2.340	1.390	1.810
Q8VDI1	Epsti1	0.617	0.891	0.000	-3.110	0.484	-0.654	-2.470	-1.520

Race	Sex	Age
Black	Male	60
Caucasian	Male	61
Black	Male	66
Black	Female	59
Caucasian	Male	61
Black	Male	64
Black	Male	63
Black	Male	51
Black	Female	54
Black	Female	66

Supplementary Table 12. Race, Sex and Age of Donors

Q99MK8	Adrbk1	0.014	0.073	0.104	0.000	-0.048	-0.465	-1.150	-0.916
Q9D1I6	Mrpl14	0.431	0.687	0.279	0.000	-0.206	0.925	-0.016	0.044
Q8BM72	Hspa13	0.001	0.015	0.000	0.147	-0.238	1.210	1.070	1.230
Q8VE19	Mios	0.833	1.000	0.167	-1.300	0.000	-1.170	-0.653	0.273
Q8R4Z4	Etv3	0.143	0.309	-1.260	0.000	2.930	2.380	3.170	3.030
P97820	Map4k4	0.129	0.291	0.077	-0.561	0.000	1.420	0.039	0.619
P41216	Acsl1	0.926	1.000	0.000	0.371	-0.869	0.044	0.090	-0.768
Q5SF07	Igf2bp2	0.047	0.150	-0.112	3.740	0.000	5.410	4.380	4.900
Q8BUV3	Gphn	0.039	0.133	-0.540	0.113	0.000	-0.819	-1.730	-1.000
Q9DBS5	Klc4	0.089	0.227	0.147	-0.698	0.000	-0.502	-1.210	-1.180
Q9R020	Zranb2	0.054	0.164	0.490	-0.013	0.000	-0.266	-1.240	-0.669
P24638	Acp2	0.034	0.121	-0.034	0.054	0.000	0.460	0.246	0.763
Q9D0D5	Gtf2e1	0.030	0.112	0.202	-0.161	0.000	-0.852	-2.490	-1.470
P52503	Ndufs6	0.095	0.237	-0.325	0.000	0.739	0.967	0.626	1.040
Q99LS3	Psph	0.014	0.072	-0.560	0.141	0.000	-1.410	-2.990	-2.800
Q6A028	Swap70	0.926	1.000	0.163	-0.424	0.000	0.391	-0.367	-0.378
Q3UMF0	Cobll1	0.420	0.674	-1.450	0.298	0.000	-1.040	-1.100	-0.545
Q8R5F3	Oard1	0.041	0.138	0.000	-0.160	0.309	-0.415	-1.080	-1.560
Q6NTA4	Rragb	0.839	1.000	0.315	0.000	-0.024	0.559	-0.365	-0.095
Q80X95	Rraga	0.839	1.000	0.315	0.000	-0.024	0.559	-0.365	-0.095
Q8CFT2	Setd1b	0.197	0.391	0.000	0.729	-0.041	-0.218	-0.221	-2.330
Q9CQ56	Use1	0.012	0.069	-0.371	0.000	0.230	1.080	0.697	0.796
Q3U186	Rars2	0.906	1.000	0.398	-0.283	0.000	1.060	-0.833	-0.334
P62488	Polr2g	0.001	0.022	-0.119	0.000	0.092	-0.560	-0.762	-0.686
Q9WVM1	Racgap1	0.320	0.555	1.770	-0.913	0.000	2.990	0.689	0.859
Q61112	Sdf4	0.541	0.808	-0.105	0.540	0.000	0.492	-0.149	0.767
Q924M7	Mpi	0.117	0.273	-0.284	0.000	0.250	-0.620	-2.930	-0.916
Q8CAY6	Acat2	0.170	0.352	0.652	-0.172	0.000	-0.123	-0.377	-0.341
Q8R2T8	Gtf3c5	0.023	0.096	-0.196	0.161	0.000	-0.379	-0.708	-0.482
Q8K4P0	Wdr33	0.948	1.000	-1.110	0.000	0.822	-1.840	1.850	-0.538
Q9Z0U1	Tjp2	0.008	0.051	-0.391	0.328	0.000	-1.280	-2.290	-2.170
Q64516	Gk	0.095	0.238	0.000	-0.012	0.006	0.349	0.017	0.348
Q8K2F0	Brd3	0.367	0.614	0.328	-1.040	0.000	-0.693	-0.824	-0.484
Q9JHU9	Isyna1	0.002	0.029	0.000	0.098	-0.167	-1.140	-1.850	-1.610
Q9D1H7	Get4	0.857	1.000	0.078	-0.294	0.000	0.092	-0.419	0.000
Q9CYN9	Atp6ap2	0.094	0.236	0.000	0.055	-0.400	1.080	0.129	0.492
Q9WUV0	Orc5	0.343	0.582	0.199	0.000	-0.561	-0.287	-0.714	-0.241
O88712	Ctbp1	0.008	0.052	0.000	-0.306	0.027	-1.060	-1.940	-1.410
Q8VEK6	Ing3	0.841	1.000	-2.000	0.000	0.232	-0.688	-1.700	0.065
Q6Q783	Kmt5c	0.331	0.568	0.000	-0.746	0.263	-0.306	-0.727	-0.531
Q91W96	Anapc4	0.893	1.000	0.000	-0.318	0.245	0.275	-0.305	0.058
P56671	Maz	0.246	0.457	-1.300	0.426	0.000	0.187	0.273	1.070
Q6P1H6	Ankle2	0.334	0.571	0.000	-0.919	0.452	0.030	0.201	0.880
Q6A065	Cep170	0.998	1.000	0.069	-0.070	0.000	0.013	-0.627	0.616
Q3U5Q7	Cmpk2	0.171	0.354	0.545	0.000	-0.063	0.143	-0.649	-0.640
Q9R0A0	Pex14	0.500	0.765	-0.427	0.003	0.000	0.334	-1.500	-0.479
A6X919	Dpy19l1	0.008	0.055	-0.026	0.177	0.000	1.360	0.686	1.070
Q9R0Q9	Mpdu1	0.207	0.404	0.000	-0.009	0.020	0.421	-0.073	0.373
Q8BK08	Tmem11	0.001	0.019	-0.043	0.285	0.000	1.080	0.954	1.080
Q9WUH1	Tmem115	0.086	0.223	-0.017	0.000	0.140	0.458	0.247	1.020
Q8K400	Stxbp5	0.503	0.767	-2.540	0.000	1.020	-0.396	-2.860	-1.110
Q61122	Nab1	0.355	0.598	-0.983	0.000	0.250	-0.976	-1.240	-0.108
Q99JT2	Stk26	0.019	0.087	-0.010	0.035	0.000	-0.926	-2.380	-1.490
Q8R409	Hexim1	0.026	0.103	-0.301	0.000	0.002	-0.507	-0.763	-1.100
Q80ZV0	Rnaseh2b	0.816	1.000	2.290	0.000	-0.370	2.040	-0.713	-0.300
P55821	Stmn2	0.078	0.208	0.511	-0.166	0.000	-0.361	-2.580	-1.480
P54227	Stmn1	0.078	0.208	0.511	-0.166	0.000	-0.361	-2.580	-1.480
Q8BU30	Iars	0.239	0.448	0.356	0.000	-0.013	0.658	0.088	0.450
Q61510	Trim25	0.944	1.000	-0.760	2.540	0.000	0.403	-0.707	1.800

Q60649	Clpb	0.990	1.000	0.421	0.000	-0.108	0.340	-0.304	0.289
P23506	Pcmt1	0.001	0.022	-0.179	0.000	0.198	-0.923	-0.926	-0.835
Q3TC33	Ccdc127	0.031	0.114	-0.180	0.197	0.000	0.651	0.308	0.506
Q9CQI6	Cotl1	0.000	0.011	0.009	-0.028	0.000	-1.450	-1.890	-1.690
Q8BFQ3	Gpr68	0.340	0.579	-0.551	0.216	0.000	-1.070	-1.480	0.309
P61514	Rpl37a	0.417	0.671	0.000	-0.469	0.078	0.438	-1.460	-0.984
P70362	Ufd1l	0.819	1.000	0.000	0.014	-1.410	-0.501	-0.022	-1.310
Q3UVL4	Vps51	0.208	0.404	0.208	-1.640	0.000	-0.988	-2.320	-1.320
Q6PAQ4	Rexo4	0.491	0.755	0.000	1.990	-4.510	0.958	0.732	0.192
Q8K3X4	Irf2bpl	0.277	0.498	0.000	-0.251	0.040	0.100	-0.645	-0.692
P81183	Ikzf2	0.043	0.141	-0.323	0.000	0.107	-0.403	-0.664	-0.474
Q921E2	Rab31	0.164	0.343	0.261	0.000	-0.128	1.650	0.002	0.999
P70122	Sbds	0.148	0.318	0.000	-0.375	0.036	-0.198	-0.845	-0.521
Q8BYK6	Ythdf3	0.400	0.650	-0.967	1.010	0.000	-0.910	-0.617	-0.155
Q8CFE4	Scyl2	0.940	1.000	0.208	0.000	-0.054	0.460	-0.112	-0.250
Q8BTT6	Diexf	0.150	0.320	0.268	-0.494	0.000	-0.240	-1.120	-0.670
A2AM29	Mllt3	0.347	0.587	-3.040	0.000	0.145	1.570	-0.014	-0.553
P97461	Rps5	0.392	0.641	0.431	0.000	-0.092	0.476	-0.557	-0.791
P59016	Vps33b	0.021	0.090	0.000	0.285	-0.108	-0.524	-1.370	-0.946
Q9CR26	Vta1	0.047	0.150	0.000	0.330	-0.281	-0.431	-0.914	-0.549
O54984	Asna1	0.442	0.698	0.075	0.000	-0.446	0.147	-0.143	0.099
Q8C827	Zfp62	0.293	0.520	-0.534	0.344	0.000	0.573	0.335	0.005
O88746	Tom1	0.080	0.212	0.000	0.397	-0.025	-0.094	-1.590	-1.260
Q78PG9	Ccdc25	0.908	1.000	-3.370	0.752	0.000	-0.726	-1.210	-1.150
P48453	Ppp3cb	0.034	0.122	-0.837	0.064	0.000	-2.010	-3.260	-1.390
Q9CY73	Mrpl44	0.009	0.057	0.000	0.358	-0.175	0.827	0.770	0.815
Q3UIA2	Arhgap17	0.173	0.356	0.521	-0.634	0.000	0.359	0.548	1.870
Q80U63	Mfn2	1.000	1.000	0.000	0.057	-0.064	-0.674	0.298	0.369
Q9CX00	Ist1	0.541	0.807	0.000	-0.229	0.048	0.269	-0.707	-0.339
Q6PDL0	Dync1li2	0.091	0.231	-1.050	0.729	0.000	1.510	0.945	0.902
Q6PCM2	Ints6	0.035	0.124	-0.670	0.000	0.128	-1.040	-1.070	-0.853
Q8VE96	Slc35f6	0.285	0.509	0.589	-0.450	0.000	0.607	-1.970	-1.740
Q8VDQ8	Sirt2	0.284	0.508	-0.577	0.764	0.000	-0.309	-2.220	-0.144
Q8VDM1	Zgpat	0.055	0.166	0.000	-0.755	0.028	-0.768	-1.080	-1.150
Q8BX09	Rbbp5	0.060	0.175	-0.133	0.000	0.042	-0.266	-0.743	-0.324
Q9D2C7	Tmbim6	0.209	0.406	-0.632	0.134	0.000	-0.118	0.420	0.780
Q8K2C8	Gpat4	0.002	0.023	0.456	0.000	-0.135	2.420	1.830	1.920
Q9DC71	Mrps15	0.711	0.987	-0.194	0.000	0.044	0.841	-0.657	0.193
Q3TCH7	Cul4a	0.019	0.085	0.019	-0.448	0.000	1.410	0.531	1.040
Q8R0K4	Ccdc137	0.576	0.846	-3.380	0.000	0.605	-0.132	-0.475	0.118
E9Q784	Zc3h13	0.413	0.665	0.000	-0.003	1.120	0.228	1.240	0.968
Q920A5	Scpep1	0.166	0.347	0.029	-0.044	0.000	0.724	-0.059	0.508
Q9CR59	Gadd45gip1	0.130	0.293	0.000	0.151	-0.194	0.895	0.031	0.578
Q64131	Runx3	0.617	0.891	0.000	2.480	-1.840	0.041	1.500	1.260
P70195	Psmb7	0.075	0.202	0.000	0.109	-0.848	-0.791	-1.820	-1.190
Q9WTL7	Lypla2	0.069	0.191	-0.604	1.470	0.000	-1.450	-2.490	-0.908
Q8BVU5	Nudt9	0.155	0.330	0.000	-0.351	0.224	0.896	2.140	0.089
O70378	Emc8	0.013	0.070	-0.015	0.398	0.000	1.360	0.752	1.150
Q8BVA5	Ldah	0.407	0.658	0.000	0.014	-0.054	0.285	-1.310	-0.310
P39428	Traf1	0.026	0.103	0.000	0.232	-0.135	-0.817	-1.800	-0.841
Q9ES74	Nek7	0.578	0.849	0.000	-0.083	0.235	0.231	-0.367	-0.069
P70428	Ext2	0.130	0.293	0.004	-4.570	0.000	1.530	1.060	1.580
Q8BFQ6	Dirc2	0.209	0.405	0.419	0.000	-0.130	0.891	0.085	0.610
Q2TPA8	Hsdl2	0.861	1.000	0.190	-0.464	0.000	0.705	-0.463	-0.284
P48725	Pcnt	0.954	1.000	0.634	-3.730	0.000	0.667	-2.740	-1.330
Q8R344	Ccdc12	0.022	0.094	0.045	-0.401	0.000	-0.707	-1.280	-1.590
Q9Z172	Sumo3	0.504	0.768	0.000	0.965	-0.168	-0.170	-3.010	1.160
Q80ZD3	Slc26a11	0.066	0.185	-0.440	0.256	0.000	0.382	0.561	1.090
Q9WU28	Pfdn5	0.026	0.104	0.337	0.000	-0.011	-0.630	-0.847	-0.279

Q9CQE7	Ergic3	0.027	0.105	0.103	-0.420	0.000	0.997	0.398	0.714
P70269	Ctse	0.222	0.423	0.292	0.000	-0.683	-0.227	-1.010	-0.757
Q4QRL3	Ccdc88b	0.044	0.142	0.000	0.221	-0.969	-2.650	-4.350	-1.550
P18052	Ptpra	0.075	0.202	-0.163	0.203	0.000	0.660	0.156	0.735
Q8BIH0	Sap130	0.032	0.117	0.000	-0.146	1.120	1.420	1.820	1.790
Q99MU3	Adar	0.004	0.038	-0.077	0.000	0.110	-1.330	-1.330	-0.753
Q811M1	Arhgap15	0.024	0.097	0.063	-0.056	0.000	-0.320	-0.940	-0.689
Q14AX6	Cdk12	0.354	0.596	0.044	-0.085	0.000	0.080	-0.202	-0.290
Q3TC72	Fahd2	0.460	0.719	0.000	0.309	-0.221	0.143	0.026	0.366
Q921I2	Klhdc4	0.441	0.697	0.550	-0.537	0.000	1.260	-0.103	0.190
Q9D0I8	Mrto4	0.098	0.242	0.240	0.000	-0.243	-0.157	-0.805	-0.552
Q8CIC2	Nupl2	0.671	0.949	-1.300	0.382	0.000	-4.180	2.690	4.170
Q8C092	Taf5	0.864	1.000	-0.202	2.780	0.000	1.600	0.516	-0.136
P97492	Rgs14	0.001	0.019	-0.396	0.195	0.000	-2.050	-1.910	-1.620
Q9R1C6	Dgke	0.653	0.929	0.000	2.230	-1.630	0.861	0.474	0.905
Q61205	Pafah1b3	0.001	0.019	-0.071	0.230	0.000	-0.968	-1.320	-1.130
Q9D8S9	Bola1	0.460	0.719	0.000	-2.370	0.187	1.050	-0.214	-0.646
Q9R1S3	Pign	0.869	1.000	-2.550	0.000	5.880	-3.330	7.380	-3.010
Q9CR68	Uqcrfs1	0.007	0.048	0.000	0.074	-0.106	0.656	0.355	0.524
Q8C7Q4	Rbm4	0.306	0.538	-0.094	0.211	0.000	-0.147	-0.129	0.023
Q80TN4	Dnajc16	0.527	0.793	0.000	3.370	-3.120	0.741	1.600	1.870
Q8CJ40	Crocc	0.148	0.318	0.071	0.000	-0.008	0.321	0.014	0.498
Q9JJY4	Ddx20	0.299	0.528	-0.178	0.000	0.117	0.198	-0.676	-0.663
Q9CQZ5	Ndufa6	0.233	0.438	0.000	0.678	-0.132	0.410	0.479	0.879
Q6PAR5	Gapvd1	0.538	0.806	0.400	0.000	-0.331	0.219	-0.246	-0.508
Q8CE46	Pus7l	0.019	0.087	0.615	-0.188	0.000	-0.920	-2.350	-2.310
Q922H4	Gmppa	0.029	0.112	0.060	-0.305	0.000	-0.535	-1.460	-1.280
Q64455	Ptprj	0.728	1.000	0.025	0.000	-1.110	-0.320	-0.571	-0.618
P63323	Rps12	0.343	0.582	-1.410	0.000	0.032	-1.460	-0.606	-1.040
Q9Z148	Ehmt2	0.654	0.930	-0.141	0.000	0.802	-0.151	-0.571	0.696
Q9JI78	Ngly1	0.004	0.037	-0.505	0.000	0.109	-1.300	-1.710	-1.850
Q99N89	Mrpl43	0.003	0.030	0.000	0.185	-0.319	1.180	0.974	1.330
O88879	Apaf1	0.004	0.033	0.100	-0.012	0.000	-0.683	-1.230	-1.050
P56382	Atp5e	0.000	0.013	0.000	0.115	-0.081	0.914	0.754	0.858
Q8BK58	Hspbap1	0.857	1.000	-0.469	0.577	0.000	1.280	-0.737	-0.052
P30355	Alox5ap	0.424	0.678	0.786	0.000	-0.301	1.160	0.291	0.218
P51829	Adcy7	0.555	0.824	-0.535	0.000	0.058	-0.200	-0.585	-0.144
P61294	Rab6b	0.031	0.116	-0.459	0.121	0.000	-1.130	-0.840	-0.616
Q9D4H8	Cul2	0.495	0.760	-1.430	1.200	0.000	0.568	-1.510	-1.640
Q8CES0	Naa30	0.115	0.270	-0.122	0.000	0.086	-0.325	-1.500	-0.472
Q9D659	Vsir	0.049	0.154	-0.084	0.110	0.000	-0.152	-0.651	-0.493
Q9Z129	Recql	0.224	0.426	0.000	-0.274	0.511	-0.249	-1.960	-0.223
P62274	Rps29	0.691	0.967	0.000	0.231	-0.784	-0.246	-0.324	-0.381
Q91YJ3	Thyn1	0.253	0.466	0.000	-0.137	0.320	-0.121	-0.330	-0.020
Q61508	Ecm1	0.050	0.155	0.000	0.399	-0.581	0.993	0.600	0.775
Q3UHD6	Snx27	0.771	1.000	-0.204	0.000	0.423	-1.050	-0.625	1.220
Q9JKN1	Slc30a7	0.061	0.176	0.000	0.548	-0.468	1.350	0.531	1.200
P47930	Fosl2	0.051	0.158	-0.388	0.000	0.238	0.409	0.428	0.671
Q9WV84	Nme4	0.027	0.105	1.300	-1.640	0.000	3.340	2.630	2.720
B1AVZ0	Uprt	0.350	0.591	0.000	-0.111	0.166	0.131	-0.627	-0.192
Q00422	Gabpa	0.266	0.485	-0.698	0.165	0.000	-0.673	-0.617	-0.342
Q9CWN7	Cnot11	0.150	0.320	0.000	-0.054	0.281	0.098	-2.610	-1.490
Q9D1P2	Kat8	0.635	0.912	0.000	1.430	-0.154	0.253	-0.149	0.359
Q9DAK9	Phpt1	0.001	0.014	-0.073	0.450	0.000	-2.300	-2.910	-3.060
Q8BYC6	Taok3	0.583	0.855	0.000	0.347	-0.177	0.237	-0.245	-0.214
Q9CTH6	Fcf1	0.015	0.076	0.162	-0.174	0.000	-0.675	-0.546	-0.377
Q9CXK9	Rbm33	0.775	1.000	0.000	-0.130	1.580	0.673	0.060	1.320
Q9D1B9	Mrpl28	0.304	0.534	0.000	0.671	-1.410	0.836	0.095	0.635
P57787	Slc16a3	0.600	0.873	0.578	0.000	-3.850	-0.543	0.643	-0.876

Q91W86	Vps11	0.178	0.364	0.000	0.651	-0.315	-0.712	-0.050	-0.625
O70469	Dok2	0.058	0.172	-0.026	0.000	0.092	-0.602	-1.430	-2.690
Q61179	Irf9	0.154	0.327	0.021	-0.204	0.000	-0.140	-1.020	-0.437
Q80U87	Usp8	0.729	1.000	0.000	-0.253	0.161	0.513	-0.883	-0.191
Q9CWP6	Mospd2	0.067	0.187	-1.080	0.277	0.000	1.090	0.599	2.170
Q6ZWM4	Lsm8	0.003	0.032	-0.155	0.000	0.168	-0.915	-1.470	-1.420
Q9CQZ0	Ormdl2	0.062	0.179	0.000	0.413	-0.174	1.130	0.665	0.468
O54988	Slk	0.686	0.963	-0.072	0.488	0.000	-0.441	0.770	0.635
Q8BGQ1	Vipas39	0.027	0.107	0.014	-0.411	0.000	-1.310	-1.870	-0.768
Q60692	Psmb6	0.528	0.795	0.114	0.000	-0.437	-0.196	-0.763	0.035
Q3UXZ9	Kdm5a	0.036	0.126	-1.040	0.237	0.000	-1.650	-3.230	-1.830
Q922R5	Ppp4r3b	0.120	0.276	-0.578	0.000	0.820	-0.544	-3.400	-1.380
Q8R4E9	Cdt1	0.525	0.791	-0.052	0.000	1.510	0.633	0.311	-0.962
Q8BIG7	Comtd1	0.937	1.000	0.000	0.166	-0.276	0.673	-0.609	-0.276
P28843	Dpp4	0.067	0.186	-0.165	0.000	0.192	-0.310	-0.587	-0.216
Q8K2D3	Edc3	0.186	0.376	0.000	0.370	-0.732	-0.284	-2.170	-0.966
Q5IRJ6	Slc30a9	0.003	0.030	0.000	0.577	-0.728	2.360	2.540	2.810
Q9CXY9	Pigk	0.948	1.000	0.000	0.302	-1.030	1.300	-1.050	-0.800
Q8QZY6	Tspan14	0.105	0.252	0.000	0.194	-0.577	-0.743	-0.701	-0.480
Q9D2D7	Znf687	0.027	0.107	-0.869	0.000	0.043	-1.140	-1.420	-1.520
Q32NY4	Cnnm3	0.062	0.179	0.143	0.000	-0.041	0.185	0.199	0.396
Q63810	Ppp3r1	0.027	0.106	0.029	0.000	-0.303	-0.823	-0.787	-1.610
Q9DB40	Med27	0.112	0.265	-0.150	0.455	0.000	0.392	0.886	0.476
Q8R480	Nup85	0.020	0.089	0.000	-0.337	0.052	-0.995	-2.360	-1.570
Q8K339	Kin	0.040	0.134	0.000	0.000	0.258	-0.190	-0.860	-0.618
Q8BJW5	Nol11	0.203	0.399	0.000	0.243	-0.456	-0.152	-1.030	-0.521
Q9CVD2	Atxn3	0.128	0.290	0.000	2.050	-1.220	-2.050	-2.400	-0.837
P12399	Ctla2a	0.017	0.081	0.000	0.559	-1.130	-2.010	-3.210	-2.490
Q924D0	Rtn4ip1	0.540	0.807	0.000	0.155	-0.927	0.162	-2.980	-0.090
P15535	B4galt1	0.213	0.410	-0.367	0.178	0.000	0.336	0.153	0.102
Q8CBC4	Cnst	0.009	0.057	-0.306	0.000	0.270	-0.789	-1.300	-1.290
Q8VDY9	Caap1	0.372	0.621	-0.225	0.000	0.447	-0.044	-0.844	0.064
Q8R2Q8	Bst2	0.003	0.033	0.189	-0.754	0.000	2.100	1.580	1.960
Q5U3K5	Rabl6	0.406	0.658	1.260	-2.210	0.000	1.200	-3.130	-4.630
Q8BZ98	Dnm3	0.116	0.271	0.100	0.000	-0.235	0.384	0.055	0.252
Q9D023	Mpc2	0.034	0.121	0.000	0.091	-0.908	0.954	0.558	0.976
O70493	Snx12	0.150	0.321	-0.260	0.065	0.000	-0.143	-0.807	-0.403
Q9DBY1	Syvn1	0.020	0.087	0.000	0.682	-0.142	1.310	1.110	1.100
Q5H8C4	Vps13a	0.282	0.505	-0.287	3.230	0.000	-3.080	-0.289	0.484
Q99KX1	Mlf2	0.315	0.549	0.000	-0.307	0.193	-0.085	-0.336	-0.256
Q60596	Xrcc1	0.290	0.517	0.023	-0.284	0.000	0.104	-0.792	-0.651
P59481	Lman2l	0.552	0.821	0.000	0.129	-0.521	0.946	-8.880	1.110
P97789	Xrn1	0.074	0.201	0.000	-0.143	0.334	-0.210	-0.476	-0.909
Q99K01	Pdxdc1	0.918	1.000	0.000	-0.172	0.089	1.040	-0.335	-0.985
Q8CIG3	Kdm1b	0.705	0.982	0.120	-1.320	0.000	-1.960	-1.000	0.665
Q9CZT6	Cmss1	0.064	0.183	0.000	-1.570	0.227	-1.610	-2.350	-1.960
Q99KK1	Reep3	0.256	0.470	0.000	-2.660	0.078	0.631	0.147	0.263
Q6P5G6	Ubxn7	0.109	0.260	0.000	1.210	-0.071	-1.190	-0.345	-0.395
Q8K2Y7	Mrpl47	0.048	0.152	0.000	0.273	-0.157	1.070	0.895	0.325
Q9CPQ1	Cox6c	0.008	0.052	0.000	0.148	-0.102	0.358	0.542	0.504
O54833	Csnk2a2	0.725	1.000	0.164	-0.263	0.000	0.721	-0.602	-0.770
Q9CRG1	Tm7sf3	0.667	0.945	0.418	-0.002	0.000	0.609	-0.354	-0.319
Q9CQA5	Med4	0.999	1.000	0.000	-2.040	1.460	0.168	-1.730	0.990
Q60676	Ppp5c	0.129	0.291	0.000	-1.090	0.502	-0.225	-4.320	-4.210
P62743	Ap2s1	0.350	0.592	0.421	-1.680	0.000	0.710	0.072	0.099
Q99N85	Mrps18a	0.010	0.060	0.176	-0.091	0.000	0.951	0.506	0.708
Q9CR11	Yeats4	0.614	0.888	0.000	2.770	-1.020	-0.268	1.670	2.690
Q9D8X2	Ccdc124	0.201	0.397	0.000	-0.531	0.050	-0.129	-1.780	-0.916
Q9D832	Dnajb4	0.901	1.000	0.150	0.000	-0.565	0.480	-0.352	-0.401

A2A8Z1	Osbpl9	0.215	0.412	0.344	0.000	-0.424	0.033	-0.872	-0.935
P14069	S100a6	0.187	0.376	-0.046	0.238	0.000	-0.128	-0.390	0.011
O55100	Syngr1	0.000	0.013	0.248	-0.546	0.000	4.430	3.640	4.470
Q6PB44	Ptpn23	0.645	0.922	0.000	0.015	-0.813	0.790	-0.854	0.086
P63030	Mpc1	0.002	0.028	0.158	-0.024	0.000	1.540	0.979	1.210
Q921N7	Tmem70	0.029	0.111	-0.295	3.890	0.000	5.300	5.780	6.340
Q9D920	Borcs5	0.162	0.340	0.264	-0.348	0.000	-0.244	-0.500	-0.331
Q9CRA4	Msmo1	0.001	0.019	0.000	-1.340	0.329	4.080	4.320	4.870
P97346	Nxn	0.333	0.570	0.333	0.000	-1.090	-2.170	0.304	-1.790
Q91VX2	Ubap2	0.246	0.457	0.280	-0.451	0.000	0.798	-0.235	1.300
Q9R0Q6	Arpc1a	0.701	0.977	0.000	0.404	-0.663	-0.086	-0.302	-0.265
Q9CYK1	Wars2	0.461	0.720	-0.361	0.518	0.000	0.617	-0.291	0.913
Q7TMQ7	Wdr91	0.770	1.000	0.000	0.261	-0.766	-0.437	-1.500	0.756
Q6DFX2	Antxr2	0.938	1.000	0.081	-4.330	0.000	0.051	-0.084	-3.740
Q6PCN7	Hltf	0.161	0.339	-0.167	0.000	0.167	-0.166	-2.240	-0.792
Q99LI8	Hgs	0.616	0.891	0.000	-3.760	1.220	0.693	-0.749	0.054
O70456	Sfn	0.035	0.123	0.000	-0.144	0.061	-0.606	-2.040	-1.400
Q6NVF4	Helb	0.030	0.113	0.000	0.432	-0.446	-3.290	-2.260	-1.130
Q61586	Gpam	0.000	0.012	0.000	-0.259	0.263	2.000	1.940	2.100
Q8VC70	Rbms2	0.050	0.155	0.875	-3.560	0.000	4.120	2.420	2.850
Q8VDV8	Mitd1	0.674	0.950	0.863	0.000	-0.130	-0.222	0.264	0.219
A2BDX3	Mocs3	0.016	0.079	-0.131	0.015	0.000	-0.574	-0.554	-0.274
P63073	Eif4e	0.110	0.261	0.056	0.000	-0.079	-0.067	-0.746	-0.443
Q8BYL4	Yars2	0.539	0.807	0.319	-1.240	0.000	0.125	-1.890	-0.671
Q9JHI9	Slc40a1	0.774	1.000	1.360	-0.984	0.000	1.010	-0.349	0.438
P41233	Abca1	0.053	0.162	2.580	-1.450	0.000	3.720	3.300	3.800
Q8C052	Map1s	0.324	0.559	1.440	0.000	-0.235	-2.860	0.557	-0.363
Q80TA6	Mtmr12	0.017	0.081	-0.216	0.000	0.039	-0.710	-1.700	-1.270
Q920L1	Fads1	0.002	0.022	0.753	0.000	-1.200	4.390	4.140	4.320
Q6NZQ4	Paxip1	0.002	0.024	-0.702	0.000	0.105	-2.740	-2.460	-2.170
Q9D786	Haus5	0.097	0.240	-0.007	0.000	0.031	-0.178	-1.330	-0.640
A2AGH6	Med12	0.669	0.947	-0.883	0.364	0.000	-0.436	-0.859	0.129
Q8VDG5	Ppcs	0.001	0.015	0.000	-0.088	0.459	-1.780	-2.250	-2.160
Q8BX17	Gemin5	0.136	0.300	0.000	-0.197	0.340	-0.034	-0.557	-0.653
Q60949	Tbc1d1	0.929	1.000	-2.370	0.000	0.522	-0.861	0.527	-1.210
P34152	Ptk2	0.297	0.524	0.207	0.000	-0.153	0.088	-0.575	-0.244
Q8VCH6	Dhcr24	0.003	0.030	-1.970	0.475	0.000	4.270	4.540	4.840
Q3UFY7	Nt5c3b	0.132	0.296	1.580	0.000	-1.680	2.400	1.420	1.670
P36536	Sar1a	0.972	1.000	0.038	-1.740	0.000	-0.594	-0.758	-0.420
O70433	Fhl2	0.300	0.529	0.301	-2.800	0.000	1.230	-0.207	0.303
P47811	Mapk14	0.232	0.436	0.000	0.568	-0.624	-3.770	-0.803	-0.291
Q9ERI6	Rdh14	0.189	0.379	0.000	0.132	-0.126	-0.072	-0.191	-0.680
O88561	Slc27a3	0.002	0.025	0.000	1.250	-0.059	3.450	3.940	4.190
Q8C1E7	Tmem120a	0.021	0.090	0.000	1.190	-0.322	3.090	2.270	1.920
P97765	Wbp2	0.002	0.024	-0.004	0.131	0.000	-1.010	-1.590	-1.550
Q3U0M1	Trappc9	0.817	1.000	-0.454	0.109	0.000	-0.300	-0.560	0.289
Q8R313	Exoc6	0.164	0.343	0.374	0.000	-0.064	-5.790	-1.000	-1.050
A6X8Z5	Arhgap31	0.879	1.000	1.430	-1.790	0.000	0.384	-2.660	2.810
Q9D6U8	Fam162a	0.000	0.013	-0.044	0.195	0.000	1.030	0.901	0.963
Q8K3C3	Lzic	0.143	0.310	0.371	-1.190	0.000	-0.852	-1.480	-1.240
Q9D125	Mrps25	0.125	0.285	5.570	-3.410	0.000	6.160	5.650	5.610
Q9R049	Amfr	0.388	0.636	-0.100	0.870	0.000	0.513	0.788	0.419
Q9DB34	Chmp2a	0.798	1.000	0.119	-0.014	0.000	0.221	-0.174	0.165
Q5U4C3	Scaf1	0.618	0.892	0.000	-0.994	0.360	0.667	-1.780	-0.847
Q8BKX6	Smg1	0.474	0.733	0.353	-1.190	0.000	-0.243	-1.940	-0.363
P49586	Pcyt1a	0.554	0.822	0.423	0.000	-0.798	0.117	-0.722	-0.634
Q9DC29	Abcb6	0.117	0.272	0.022	-0.201	0.000	-0.174	-0.821	-0.399
O54998	Fkbp7	0.022	0.095	-0.509	2.610	0.000	4.180	3.980	4.750
Q8VDZ4	Zdhhc5	0.251	0.463	0.000	-0.624	1.340	0.439	1.390	1.660

Q61037	Tsc2	0.494	0.759	-0.314	0.000	0.272	0.238	-0.069	0.232
Q9CZ57	Nsun4	0.430	0.686	0.129	-1.190	0.000	0.559	-0.895	1.310
P62309	Snrpg	0.091	0.232	0.000	0.621	-0.692	-0.743	-1.190	-0.818
Q80U70	Suz12	0.113	0.266	0.000	0.065	-0.310	-0.990	-2.960	-0.684
O35344	Kpna3	0.032	0.118	0.000	0.111	-0.203	-0.415	-1.000	-0.587
Q9DAT5	Trmu	0.965	1.000	0.330	-0.121	0.000	0.746	-0.391	-0.095
O88811	Stam2	0.844	1.000	0.793	-0.066	0.000	-2.650	4.530	0.183
P52623	Uck1	0.106	0.254	0.000	-0.181	0.131	-0.078	-0.618	-0.720
Q60575	Kif1b	0.549	0.817	0.000	0.476	-0.013	1.190	-2.150	-0.500
Q8BZQ7	Anapc2	0.858	1.000	0.000	0.388	-1.980	1.800	-1.690	-2.560
P26011	Itgb7	0.206	0.403	-0.370	1.190	0.000	-0.580	-0.272	-0.497
B2RX14	Zcchc11	0.288	0.513	0.813	-0.294	0.000	0.265	-2.670	-0.521
O35130	Emg1	0.066	0.186	0.220	-0.200	0.000	-0.494	-2.150	-1.090
Q8CD26	Slc35e1	0.070	0.193	1.180	-0.035	0.000	2.010	1.630	1.070
Q69ZC8	Gpalpp1	0.047	0.150	-0.158	1.170	0.000	3.410	1.640	1.870
Q9JM13	Rabgef1	0.270	0.489	0.000	0.936	-0.963	0.109	-2.890	-1.190
Q5SS80	Dhrs13	0.001	0.019	-0.233	0.000	0.067	1.520	1.110	1.520
Q3UE37	Ube2z	0.134	0.298	0.000	-0.126	0.232	0.110	-2.060	-2.380
Q7TN58	Tmc8	0.075	0.202	-0.585	0.000	0.174	-1.240	-1.290	-0.445
Q8BLR5	Psd4	0.060	0.175	0.000	-0.228	0.469	-1.850	-2.990	-0.583
Q3UL36	Arglu1	0.210	0.407	0.098	-0.107	0.000	0.196	-0.943	-1.200
P34884	Mif	0.193	0.385	-0.437	0.000	0.174	-0.562	-4.920	-1.210
Q8VC65	Nrm	0.086	0.222	0.000	0.145	-0.254	0.254	0.325	0.165
Q03141	Mark3	0.872	1.000	0.347	0.000	-0.830	0.273	-0.380	-0.172
Q3TWF6	Wdr70	0.840	1.000	0.000	2.870	-1.380	0.191	1.450	-1.090
Q99NH0	Ankrd17	0.978	1.000	0.000	-0.078	0.040	0.641	-0.560	-0.088
Q9D1R1	Tmem126b	0.441	0.698	0.146	-0.371	0.000	-0.573	0.779	0.798
Q99LI9	Clp1	0.049	0.154	0.000	-0.466	0.106	-0.628	-0.539	-0.741
Q8BKI2	Tnrc6b	0.181	0.368	0.385	-0.531	0.000	-0.390	-0.386	-0.939
O35216	Cenpa	0.114	0.268	0.001	-0.082	0.000	-0.253	-0.531	-0.092
Q6PIJ4	Nfrkb	0.972	1.000	-1.190	0.727	0.000	0.003	-0.075	-0.451
Q3U487	Hectd3	0.509	0.773	-0.887	0.876	0.000	0.088	-1.320	-0.223
P59178	L3mbtl2	0.800	1.000	0.000	0.358	-1.860	0.594	-0.726	-2.230
A6H630	Armt1	0.012	0.067	0.101	-0.391	0.000	-1.820	-4.060	-3.390
P83887	Tubg1	0.204	0.400	-0.306	0.000	0.031	-0.359	-0.367	-0.142
Q7TPN9	Prr14	0.091	0.231	-0.071	0.000	0.554	-0.152	-0.582	-0.336
Q6ZQE4	Nemp1	0.975	1.000	0.000	-0.209	0.239	0.125	-0.173	0.093
Q9QYR6	Map1a	0.277	0.498	-1.020	0.047	0.000	-0.594	-0.990	-0.769
Q8K296	Mtmr3	0.128	0.290	0.000	0.462	-0.686	-0.643	-0.845	-0.685
Q68FE8	Znf280d	0.869	1.000	0.000	-3.330	0.418	-1.590	-1.570	-0.412
Q3TQB2	Foxred1	0.796	1.000	-0.086	0.930	0.000	0.212	0.095	0.873
Q9WU62	Incenp	0.140	0.306	0.524	0.000	-0.282	-0.029	-0.934	-0.856
Q9CQ91	Ndufa3	0.595	0.868	-0.286	0.062	0.000	0.320	-0.326	0.166
Q8CIG8	Prmt5	0.035	0.124	0.000	0.259	-0.546	-0.723	-1.530	-1.800
P30677	Gna14	0.352	0.593	0.000	2.050	-0.325	3.440	0.286	1.720
Q8C5P5	Nt5dc1	0.002	0.025	-0.291	0.000	0.247	-1.180	-1.430	-1.520
P30285	Cdk4	0.927	1.000	-1.000	0.000	0.348	0.457	-0.400	-0.875
Q99JW2	Acy1	0.219	0.418	-1.060	3.310	0.000	-0.115	-1.590	-2.610
Q9CS42	Prps2	0.013	0.070	0.016	-0.356	0.000	-0.738	-1.420	-1.340
P23772	Gata3	0.502	0.767	-6.200	0.000	0.195	-0.520	-0.462	-0.382
Q8BGR9	Ublcp1	0.647	0.923	0.155	-1.020	0.000	-0.287	-0.813	-0.360
Q9EQG9	Col4a3bp	0.061	0.177	0.743	0.000	-0.010	-0.270	-1.100	-0.586
Q99KW3	Triobp	0.447	0.704	1.350	0.000	-0.569	1.560	0.174	0.802
P49446	Ptpre	0.361	0.607	0.248	0.000	-0.421	0.026	-0.348	-0.818
P81069	Gabpb2	0.101	0.247	-0.582	0.000	0.001	-0.633	-0.904	-0.497
Q9CPR8	Nsmce3	0.214	0.411	0.219	-0.997	0.000	-0.521	-1.170	-0.944
Q99N95	Mrpl3	0.067	0.187	0.000	0.558	-0.395	1.000	0.576	0.857
Q9R1C0	Taf7	0.309	0.542	0.000	0.218	-0.124	-0.012	-0.048	-0.424
Q9QZS3	Numb	0.846	1.000	0.000	-2.960	1.490	-1.000	-0.066	0.457

Q9JIB4	Gtf2h2	0.243	0.453	0.000	-0.382	0.134	-0.180	-1.860	-0.442
Q9DB10	Smdt1	0.087	0.224	-0.402	0.296	0.000	0.541	0.458	0.326
A2A6Q5	Cdc27	0.004	0.038	0.037	0.000	-0.024	1.120	0.633	0.785
Q8CG50	Rab43	0.799	1.000	-3.230	0.000	0.170	0.428	-0.190	-4.960
O88796	Rpp30	0.185	0.374	0.000	0.657	-0.507	1.050	0.269	0.787

Supplementary Table 2. T cell exhaustion associated proteins detected in CD4+ T cells from old mice with or without mito-transfer. CD4+ T cells from young and old mice, and from old mice after mito-transfer were cultured for 4 h before processing for mass spectrometry analysis. Data expressed as median protein Log2 fold change of CD4+ T cells from old mice, from 3 individual old mice (paired experiment).

GO	Category	Description	Count	%	Log10(P)	Log10(q)
GO:0045333	GO BP	cellular respiration	37	12.71	-31.24	-26.97
mmu04141	KEGG	Protein processing in endoplasmic reticulum - Mus musculus (house mouse)	29	9.97	-22.64	-19.77
mmu00100	KEGG	Steroid biosynthesis - Mus musculus (house mouse)	9	3.09	-11.64	-9.19
R-MMU-1592230	Reactome	Mitochondrial biogenesis	9	3.09	-10.23	-7.82
GO:0006637	GO BP	acyl-CoA metabolic process	13	4.47	-9.97	-7.57
GO:0061024	GO BP	membrane organization	34	11.68	-9.92	-7.53
GO:0006457	GO BP	protein folding	17	5.84	-9.74	-7.36
GO:0043603	GO BP	amide metabolic process	35	12.03	-8.81	-6.49
GO:0090150	GO BP	establishment of protein localization to membrane	18	6.19	-8.81	-6.49
mmu01200	KEGG	Carbon metabolism - Mus musculus (house mouse)	13	4.47	-7.9	-5.62
GO:0018126	GO BP	protein hydroxylation	7	2.41	-7.1	-4.84
WP662	WikiPathwa	Amino acid metabolism	11	3.78	-7.06	-4.81
CORUM:413	CORUM	(ER)-localized multiprotein complex, Ig heavy chains associated	5	1.72	-6.95	-4.7
GO:0007007	GO BP	inner mitochondrial membrane organization	7	2.41	-6.66	-4.44
GO:0098660	GO BP	inorganic ion transmembrane transport	26	8.93	-6.59	-4.37
GO:0034976	GO BP	response to endoplasmic reticulum stress	15	5.15	-6.12	-3.96
R-MMU-8949664	Reactome	Processing of SMDT1	5	1.72	-5.74	-3.6
GO:0031647	GO BP	regulation of protein stability	17	5.84	-5.7	-3.57
GO:0006851	GO BP	mitochondrial calcium ion transmembrane transport	5	1.72	-5.59	-3.48
GO:1902930	GO BP	regulation of alcohol biosynthetic process	7	2.41	-5.11	-3.03

Supplementary Table 3. The Top 20 processes of significantly upregulated DEPs in CD4+ T cells from old mice, with or without mito-transfer in relation to T Cell exhaustion. Data generated through Pathway & Process Enrichment (PPE) analysis using the Metascape analysis platform.

GO	Description	Log10(P)
GO:0045333	cellular respiration	-33.8
R-MMU-1428517	The citric acid (TCA) cycle and respiratory electron transport	-33.3
GO:0009060	aerobic respiration	-32.8

Supplementary Table 4. The Top 3 Protein-Protein Interaction (PPI) networks in CD4+ T cells from old mice, with or without mito-transfer, in relation to T Cell exhaustion. Data generated using the Metascape analysis platform.

MCODE	GO	Description	Log10(P)
MCODE_1	R-MMU-163200	Respiratory electron transport, ATP synthesis by chemiosmotic coupling, and heat production by uncoupling proteins.	-48.1
MCODE_1	mmu00190	Oxidative phosphorylation - Mus musculus (house mouse)	-46.8
MCODE_1	WP295	Electron transport chain	-46.8
MCODE_2	GO:0032543	mitochondrial translation	-22.7
MCODE_2	R-MMU-5389840	Mitochondrial translation elongation	-21.6
MCODE_2	R-MMU-5419276	Mitochondrial translation termination	-21.4
MCODE_3	mmu04141	Protein processing in endoplasmic reticulum - Mus musculus (house mouse)	-21
MCODE_3	mmu00513	Various types of N-glycan biosynthesis - Mus musculus (house mouse)	-17
MCODE_3	GO:0018279	protein N-linked glycosylation via asparagine	-16.5
MCODE_4	GO:0071681	cellular response to indole-3-methanol	-9.1
MCODE_4	GO:0071680	response to indole-3-methanol	-9.1
MCODE_4	mmu05200	Pathways in cancer - Mus musculus (house mouse)	-9.1
MCODE_5	GO:0030036	actin cytoskeleton organization	-4.6
MCODE_5	GO:0030029	actin filament-based process	-4.4
MCODE_5	mmu04510	Focal adhesion - Mus musculus (house mouse)	-4.3
MCODE_6	mmu00280	Valine, leucine and isoleucine degradation - Mus musculus (house mouse)	-8.8
MCODE_7	mmu04141	Protein processing in endoplasmic reticulum - Mus musculus (house mouse)	-12.6
MCODE_7	GO:0006457	protein folding	-7.2
MCODE_7	GO:0034976	response to endoplasmic reticulum stress	-6.7
MCODE_8	mmu00100	Steroid biosynthesis - Mus musculus (house mouse)	-18.5
MCODE_8	R-MMU-191273	Cholesterol biosynthesis	-17.5
MCODE_8	GO:1902653	secondary alcohol biosynthetic process	-16.4
MCODE_9	GO:0009060	aerobic respiration	-10.7
MCODE_9	R-MMU-1428517	The citric acid (TCA) cycle and respiratory electron transport	-10.6
MCODE_9	GO:0009152	purine ribonucleotide biosynthetic process	-10.5
MCODE_10	mmu04141	Protein processing in endoplasmic reticulum - Mus musculus (house mouse)	-5.7
MCODE_11	WP103	Cholesterol biosynthesis	-8.9
MCODE_11	mmu00100	Steroid biosynthesis - Mus musculus (house mouse)	-8.5
MCODE_11	R-MMU-191273	Cholesterol biosynthesis	-8.1
MCODE_12	R-MMU-1650814	Collagen biosynthesis and modifying enzymes	-7.7
MCODE_12	mmu00310	Lysine degradation - Mus musculus (house mouse)	-7.6
MCODE_12	R-MMU-1474290	Collagen formation	-7.4
MCODE_13	GO:0010817	regulation of hormone levels	-4.6
MCODE_13	GO:0014070	response to organic cyclic compound	-4.3
MCODE_14	GO:0006457	protein folding	-6.3
MCODE_14	GO:0051604	protein maturation	-5

Supplementary Table 5. The MCODE networks identified for significantly upregulated DEPs in CD4+ T cells from old mice, with or without mito-transfer, in relation to T Cell exhaustion. Data generated using the Metascape analysis platform.

GO	Category	Description	Count	%	Log10(P)	Log10(q)
R-MMU-8953854	Reactome	Metabolism of RNA	55	19.57	-29.98	-25.71
R-MMU-450531	Reactome	Regulation of mRNA stability by proteins that bind AU-rich elements	23	8.19	-23.34	-19.38
mmu00010	KEGG	Glycolysis / Gluconeogenesis - Mus musculus (house mouse)	11	3.91	-8.91	-6.72
R-MMU-6798695	Reactome	Neutrophil degranulation	27	9.61	-8.71	-6.53
GO:0030029	GO BP	actin filament-based process	28	9.96	-8.37	-6.2
GO:0034655	GO BP	nucleobase-containing compound catabolic process	18	6.41	-7.59	-5.44
mmu00270	KEGG	Cysteine and methionine metabolism - Mus musculus (house mouse)	9	3.2	-7.38	-5.24
GO:0050684	GO BP	regulation of mRNA processing	13	4.63	-7.35	-5.22
GO:0098761	GO BP	cellular response to interleukin-7	6	2.14	-7.25	-5.13
WP572	WikiPathwa	EGFR1 signaling pathway	14	4.98	-7.01	-4.92
R-MMU-194315	Reactome	Signaling by Rho GTPases	25	8.9	-6.68	-4.6
GO:0010563	GO BP	negative regulation of phosphorus metabolic process	21	7.47	-6.47	-4.4
GO:0052548	GO BP	regulation of endopeptidase activity	19	6.76	-6.41	-4.34
GO:0044283	GO BP	small molecule biosynthetic process	20	7.12	-5.57	-3.56
GO:0031123	GO BP	RNA 3'-end processing	9	3.2	-5.51	-3.51
WP2185	WikiPathwa	Purine metabolism	12	4.27	-5.47	-3.47
WP387	WikiPathwa	IL-6 signaling pathway	9	3.2	-5.16	-3.19
R-MMU-109606	Reactome	Intrinsic Pathway for Apoptosis	6	2.14	-5	-3.04
GO:0071364	GO BP	cellular response to epidermal growth factor stimulus	6	2.14	-5	-3.04
GO:0022613	GO BP	ribonucleoprotein complex biogenesis	18	6.41	-4.75	-2.82

Supplementary Table 6. The Top 20 processes of significantly downregulated DEPs in CD4+ T cells from old mice, with or without mito-transfer, in relation to T Cell exhaustion. Data generated through Pathway & Process Enrichment (PPE) analysis using the Metascape analysis platform.

GO	Description	Log10(P)
R-MMU-8953854	Metabolism of RNA	-30.6
R-MMU-450531	Regulation of mRNA stability by proteins that bind AU-rich elements	-24.5
R-MMU-1236978	Cross-presentation of soluble exogenous antigens (endosomes)	-22

Supplementary Table 7. The Top 3 Protein-Protein Interaction (PPI) networks of significantly downregulated DEPs in CD4+ T cells from old mice, with or without mito-transfer, in relation to T Cell exhaustion. Data generated using the Metascape analysis platform.

MCODE	GO	Description	Log10(P)
MCODE_1	R-MMU-1169091	Activation of NF-kappaB in B cells	-47
MCODE_1	R-MMU-1234176	hydroxylation of Hypoxia-inducible Factor Alpha	-46.5
MCODE_1	R-MMU-1234174	Cellular response to hypoxia	-46
MCODE_2	mmu00010	Glycolysis / Gluconeogenesis - Mus musculus (house mouse)	-13.4
MCODE_2	WP157	Glycolysis and gluconeogenesis	-9.5
MCODE_2	GO:0006007	glucose catabolic process	-8.5
MCODE_3	R-MMU-75035	Chk1/Chk2(Cds1) mediated inactivation of Cyclin B:Cdk1 complex	-10
MCODE_3	R-MMU-111447	mitochondria	-10
MCODE_3	R-MMU-114452	Activation of BH3-only proteins	-9.2
MCODE_4	mmu03040	mouse)	-17
MCODE_4	R-MMU-72163	mRNA Splicing - Major Pathway	-16.7
MCODE_4	R-MMU-72172	mRNA Splicing	-16.6
MCODE_5	GO:1902115	regulation of organelle assembly	-3.7
MCODE_5	GO:0032535	regulation of cellular component size	-3
MCODE_5	GO:0043254	complex assembly	-2.9
MCODE_6	GO:0016051	carbohydrate biosynthetic process	-4.6
MCODE_6	GO:0006006	glucose metabolic process	-4.5
MCODE_6	GO:0019318	hexose metabolic process	-4.2
MCODE_7	WP407	Kit receptor signaling pathway	-8.9
MCODE_7	mmu04658	Th1 and Th2 cell differentiation - Mus musculus (house mouse)	-8.4
MCODE_7	R-MMU-8854691	Interleukin-20 family signaling	-8.3
MCODE_8	R-MMU-6807505	genes	-9.2
MCODE_8	GO:0006366	transcription by RNA polymerase II	-6.5
MCODE_8	GO:0006351	DNA-templated transcription	-5.9
MCODE_9	GO:0097435	supramolecular fiber organization	-4.1
MCODE_10	R-MMU-6791226	Major pathway of rRNA processing in the nucleolus and cytosol	-6.2
MCODE_10	R-MMU-72312	rRNA processing	-6.2
MCODE_10	R-MMU-8868773	cytosol	-6.2

Supplementary Table 8. The MCODE networks identified significantly downregulated DEPs in CD4+ T cells from old mice, with or without mito-transfer, in relation to T Cell exhaustion. Data generated using the Metascape analysis platform.

Uniprot ID	Gene Symbol	t-test	FDR (adj. P-val.)	O1	O2	O3	OM1	OM2	OM3
P16125	Ldhb	0.000	0.013	-0.212	0.132	0.000	-1.330	-1.570	-1.550
P08228	Sod1	0.000	0.013	-0.224	0.010	0.000	-1.080	-1.290	-1.170
Q922W5	Pycr1	0.000	0.014	0.208	0.000	-0.219	2.510	1.920	2.420
P63101	Ywhaz	0.001	0.015	0.000	-0.021	0.113	-1.140	-1.610	-1.370
P06151	Ldha	0.001	0.018	-0.165	0.115	0.000	-0.980	-1.190	-0.934
Q9DB20	Atp5o	0.001	0.019	0.000	0.129	-0.067	0.904	0.673	0.901
P23506	Pcmt1	0.001	0.022	-0.179	0.000	0.198	-0.923	-0.926	-0.835
Q8R0F3	Sumf1	0.002	0.024	0.000	0.232	-0.568	1.860	1.650	1.660
Q01853	Vcp	0.002	0.025	0.000	0.056	-0.134	-0.465	-0.616	-0.590
P17182	Eno1	0.002	0.028	0.055	-0.141	0.000	-0.948	-1.530	-1.280
P48678	Lmna	0.002	0.028	0.000	0.046	-0.348	0.967	0.745	0.972
P35235	Ptpn11	0.002	0.029	-0.286	0.010	0.000	-0.906	-1.190	-0.916
Q04207	Rela	0.003	0.030	0.000	-0.006	0.032	-0.840	-1.440	-1.170
P27641	Xrcc5	0.003	0.030	-0.102	0.000	0.052	-1.050	-1.770	-1.530
P28474	Adh5	0.003	0.032	-0.238	0.142	0.000	-1.150	-1.810	-1.400
Q62077	Plcg1	0.003	0.033	-0.125	0.000	0.471	-1.190	-1.370	-1.750
P19157	Gstp1	0.004	0.036	-0.138	0.000	0.106	-1.050	-1.380	-0.809
P00493	Hprt1	0.004	0.038	0.000	0.060	-0.208	-1.260	-1.950	-1.240
Q9QXS6	Dbn1	0.005	0.039	-1.190	2.450	0.000	7.040	6.310	6.710
Q9R1E0	Foxo1	0.005	0.039	-0.131	0.075	0.000	-0.360	-0.460	-0.388
Q60631	Grb2	0.005	0.041	-0.091	0.403	0.000	-1.080	-1.510	-0.944
P52431	Pold1	0.005	0.043	-0.206	0.235	0.000	-0.797	-1.220	-0.903
P63038	Hspd1	0.006	0.044	0.000	0.119	-0.240	1.090	0.650	0.822
P42232	Stat5b	0.007	0.048	-0.226	0.000	0.150	-0.984	-1.490	-0.914
P63017	Hspa8	0.008	0.054	0.046	0.000	-0.090	-0.386	-0.759	-0.718
P23475	Xrcc6	0.009	0.056	0.040	0.000	-0.446	-0.978	-1.630	-1.300
O08734	Bak1	0.010	0.060	0.000	0.028	-0.547	0.687	0.764	0.655
Q9D666	Sun1	0.011	0.064	-0.159	0.054	0.000	0.644	0.375	0.368
Q06890	Clu	0.014	0.073	0.000	0.199	-0.686	-1.550	-2.880	-1.990
P42230	Stat5a	0.015	0.076	-0.331	0.072	0.000	-0.731	-1.550	-1.370
Q8R4K2	Irak4	0.016	0.078	0.152	0.000	-0.178	-0.685	-1.670	-1.410
P10639	Txn	0.016	0.079	0.196	-0.365	0.000	-0.914	-2.060	-1.810
P25799	Nfkb1	0.017	0.080	0.000	-0.065	0.139	-0.430	-1.120	-0.909
P47791	Gsr	0.018	0.084	0.341	0.000	-0.118	-0.466	-1.080	-1.080
P07901	Hsp90aa1	0.021	0.090	0.000	0.010	-0.335	-0.557	-1.180	-1.010
P09671	Sod2	0.023	0.096	-0.061	0.348	0.000	0.715	0.476	0.682
P70371	Terf1	0.025	0.102	0.000	0.395	-0.619	-1.120	-1.050	-1.160
P39428	Traf1	0.026	0.103	0.000	0.232	-0.135	-0.817	-1.800	-0.841
P28352	Apex1	0.028	0.107	0.000	-0.288	0.047	-0.452	-0.881	-0.582
Q03147	Cdk7	0.029	0.110	0.000	-0.008	0.017	-0.257	-0.564	-0.237
P20444	Prkca	0.030	0.113	-0.268	0.109	0.000	-0.390	-0.460	-0.434
P35821	Ptpn1	0.031	0.116	-0.040	0.235	0.000	-0.162	-0.284	-0.302
P12023	App	0.032	0.117	4.640	-1.820	0.000	7.180	7.130	7.230
P63166	Sumo1	0.032	0.119	-0.103	0.000	0.363	-0.366	-0.411	-0.342
Q9QVP9	Ptk2b	0.038	0.131	-1.180	0.133	0.000	-1.480	-1.840	-1.650
P43404	Zap70	0.038	0.131	-0.465	0.041	0.000	-0.669	-0.946	-0.589
Q9Z2A7	Dgat1	0.041	0.136	0.000	0.214	-0.404	0.617	0.361	0.650
P06537	Nr3c1	0.043	0.141	-0.291	0.000	0.306	-0.491	-0.996	-0.556
P16858	Gapdh	0.043	0.142	-0.051	0.026	0.000	-0.153	-0.557	-0.357
P24270	Cat	0.046	0.148	0.177	0.000	-0.253	1.040	0.403	0.494
Q8BKJ9	Sirt7	0.048	0.152	0.000	-0.263	0.089	-0.314	-0.839	-0.582
Q60520	Sin3a	0.049	0.154	-0.146	0.000	0.086	-0.240	-0.604	-0.309
P04202	Tgfb1	0.054	0.163	0.000	0.044	-0.363	-0.731	-0.344	-0.794
P14733	Lmnb1	0.060	0.175	-0.138	0.000	0.107	-0.348	-0.608	-0.184
P33609	Pola1	0.060	0.176	0.058	0.000	-0.834	-1.570	-1.450	-0.752

O09172	Gclm	0.514	0.778	0.497	0.000	-0.914	0.147	-0.892	-0.812
Q9QXS6	Dbn1	0.005	0.039	-1.190	2.450	0.000	7.040	6.310	6.710
Q80W54	Zmpste24	0.080	0.211	0.027	0.000	-0.220	0.715	0.060	0.482
P11103	Parp1	0.506	0.770	-0.125	0.040	0.000	-0.190	-0.321	0.120
P11352	Gpx1	0.131	0.294	0.457	0.000	-0.235	-0.028	-0.804	-0.877
Q04750	Top1	0.149	0.319	0.118	-0.057	0.000	-0.028	-0.407	-0.160
P39749	Fen1	0.169	0.351	0.002	-0.215	0.000	-0.243	-0.778	-0.197
Q9CQB5	Cisd2	0.179	0.365	0.000	0.161	-0.086	0.224	0.101	0.145
P07901	Hsp90aa1	0.021	0.090	0.000	0.010	-0.335	-0.557	-1.180	-1.010
Q02111	Prkcq	0.722	1.000	0.000	1.430	-0.213	-0.229	0.752	0.016
Q60631	Grb2	0.005	0.041	-0.091	0.403	0.000	-1.080	-1.510	-0.944
P42230	Stat5a	0.015	0.076	-0.331	0.072	0.000	-0.731	-1.550	-1.370
P17918	Pcna	0.142	0.309	0.516	0.000	-0.203	-0.004	-0.794	-0.676
Q8BKJ9	Sirt7	0.048	0.152	0.000	-0.263	0.089	-0.314	-0.839	-0.582
P09671	Sod2	0.023	0.096	-0.061	0.348	0.000	0.715	0.476	0.682
P52431	Pold1	0.005	0.043	-0.206	0.235	0.000	-0.797	-1.220	-0.903
P23475	Xrcc6	0.009	0.056	0.040	0.000	-0.446	-0.978	-1.630	-1.300
P98083	Shc1	0.721	1.000	0.042	-0.011	0.000	1.180	-0.727	0.209
P35700	Prdx1	0.130	0.293	0.028	0.000	-0.030	0.421	-0.006	0.325
P70399	Tp53bp1	0.062	0.178	0.267	0.000	-0.013	-0.418	-1.440	-0.510
Q9Z2A7	Dgat1	0.041	0.136	0.000	0.214	-0.404	0.617	0.361	0.650
P63280	Ube2i	0.764	1.000	0.000	0.245	-0.255	-0.134	-0.038	0.016
Q04207	Rela	0.003	0.030	0.000	-0.006	0.032	-0.840	-1.440	-1.170
P16125	Ldhb	0.000	0.013	-0.212	0.132	0.000	-1.330	-1.570	-1.550
Q99J62	Rfc4	0.246	0.457	0.532	-0.054	0.000	0.414	-0.865	-1.300
Q08481	Pecam1	0.631	0.907	0.000	1.060	-0.637	-0.841	0.355	-0.038
Q6GQT1	A2m	0.349	0.590	0.000	-0.058	0.442	0.387	0.048	0.814
Q8K3H0	Appl1	0.122	0.280	-0.019	0.000	0.087	0.005	-0.899	-0.625
P42232	Stat5b	0.007	0.048	-0.226	0.000	0.150	-0.984	-1.490	-0.914
Q9QVP9	Ptk2b	0.038	0.131	-1.180	0.133	0.000	-1.480	-1.840	-1.650
Q9R1E0	Foxo1	0.005	0.039	-0.131	0.075	0.000	-0.360	-0.460	-0.388
P01901	H2-K1	0.918	1.000	0.000	1.100	-0.068	-0.245	0.777	0.664
P29533	Vcam1	0.146	0.315	0.746	0.000	-0.120	0.114	-1.040	-0.893
Q62388	Atm	0.107	0.257	-0.265	0.116	0.000	-0.451	-1.290	-0.368
P62137	Ppp1ca	0.822	1.000	0.237	-0.916	0.000	-0.030	-0.612	-0.318
P20444	Prkca	0.030	0.113	-0.268	0.109	0.000	-0.390	-0.460	-0.434
P41245	Mmp9	0.231	0.435	2.280	0.000	-0.046	0.785	-1.710	-1.570
Q03147	Cdk7	0.029	0.110	0.000	-0.008	0.017	-0.257	-0.564	-0.237
P10126	Eef1a1	0.463	0.721	0.222	-0.135	0.000	0.343	-0.593	-0.404
P28474	Adh5	0.003	0.032	-0.238	0.142	0.000	-1.150	-1.810	-1.400
P10639	Txn	0.016	0.079	0.196	-0.365	0.000	-0.914	-2.060	-1.810
P47791	Gsr	0.018	0.084	0.341	0.000	-0.118	-0.466	-1.080	-1.080
P68181	Prkacb	0.979	1.000	-1.250	0.245	0.000	-0.353	-0.234	-0.378
Q8R0F3	Sumf1	0.002	0.024	0.000	0.232	-0.568	1.860	1.650	1.660
Q8VEE4	Rpa1	0.072	0.197	0.054	-0.271	0.000	-0.678	-1.690	-0.560
P97313	Prkdc	0.549	0.818	0.000	-0.329	0.216	-0.441	-0.410	0.217
P04202	Tgfb1	0.054	0.163	0.000	0.044	-0.363	-0.731	-0.344	-0.794
P12023	App	0.032	0.117	4.640	-1.820	0.000	7.180	7.130	7.230
O35144	Terf2	0.061	0.176	-0.147	0.408	0.000	-0.504	-0.507	-0.217
P60766	Cdc42	0.652	0.928	0.377	-0.109	0.000	0.126	-0.409	0.199
Q9JLN9	Mtor	0.116	0.271	0.095	0.000	-0.092	-0.022	-0.391	-0.444
P28798	Grn	0.144	0.311	0.111	0.000	-1.300	-0.647	-1.980	-2.100
Q5DW34	Ehmt1	0.432	0.687	0.000	-0.729	0.184	-0.148	-1.100	-0.351
P17879	Hspa1b	0.401	0.652	0.000	-1.290	0.818	0.709	0.226	0.366
P08226	Apoe	0.508	0.772	0.861	-0.299	0.000	0.960	0.161	0.360
Q63844	Mapk3	0.097	0.239	0.625	-0.043	0.000	-0.104	-0.713	-0.406
Q8K3K7	Agpat2	0.136	0.300	0.673	-0.697	0.000	0.782	0.680	0.732
Q62077	Plcg1	0.003	0.033	-0.125	0.000	0.471	-1.190	-1.370	-1.750
P33609	Pola1	0.060	0.176	0.058	0.000	-0.834	-1.570	-1.450	-0.752

P70371	Terf1	0.025	0.102	0.000	0.395	-0.619	-1.120	-1.050	-1.160
P25799	Nfkb1	0.017	0.080	0.000	-0.065	0.139	-0.430	-1.120	-0.909
P28867	Prkcd	0.874	1.000	0.163	0.000	-1.170	-0.357	0.299	-1.260
P11440	Cdk1	0.243	0.453	0.534	0.000	-1.540	1.200	-0.031	0.779
P00493	Hprt1	0.004	0.038	0.000	0.060	-0.208	-1.260	-1.950	-1.240
O88351	Ikbkb	0.730	1.000	0.314	-0.360	0.000	0.876	-0.396	-0.052
P63166	Sumo1	0.032	0.119	-0.103	0.000	0.363	-0.366	-0.411	-0.342
Q8R4K2	Irak4	0.016	0.078	0.152	0.000	-0.178	-0.685	-1.670	-1.410
P35991	Btk	0.317	0.551	0.632	0.000	-0.712	-0.088	-0.620	-0.963
Q925J9	Med1	0.684	0.960	0.315	-0.113	0.000	0.119	-0.251	0.102
Q9D6Y7	Msra	0.068	0.189	0.549	-0.217	0.000	-0.252	-0.768	-0.814
Q60848	Hells	0.176	0.360	0.617	-0.364	0.000	0.051	-1.150	-0.971
Q9D1M4	Eef1e1	0.658	0.934	0.000	0.081	-0.123	0.368	-0.741	-0.135
P08556	Nras	0.188	0.378	-0.332	0.239	0.000	0.065	0.343	0.475
Q9D666	Sun1	0.011	0.064	-0.159	0.054	0.000	0.644	0.375	0.368
P34902	Il2rg	0.090	0.230	-0.469	0.000	0.113	-0.901	-0.440	-0.531
P97494	Gclc	0.814	1.000	0.000	-3.610	0.176	-1.950	-2.290	-0.243
Q9WUD1	Stub1	0.453	0.711	0.263	0.000	-0.427	-2.900	0.114	0.084
B2RWS6	Ep300	0.670	0.948	0.000	1.170	-0.194	0.000	0.248	0.134
O35904	Pik3cd	0.505	0.769	0.000	-0.553	0.026	-0.185	-0.997	-0.099
Q60974	Ncor1	0.231	0.436	-0.323	0.025	0.000	-0.567	-1.790	-0.112
Q60855	Ripk1	0.652	0.929	0.790	0.000	-0.944	0.818	-1.660	-0.584
Q61510	Trim25	0.944	1.000	-0.760	2.540	0.000	0.403	-0.707	1.800
P23506	Pcmt1	0.001	0.022	-0.179	0.000	0.198	-0.923	-0.926	-0.835
P39428	Traf1	0.026	0.103	0.000	0.232	-0.135	-0.817	-1.800	-0.841
Q9Z148	Ehmt2	0.654	0.930	-0.141	0.000	0.802	-0.151	-0.571	0.696
P51829	Adcy7	0.555	0.824	-0.535	0.000	0.058	-0.200	-0.585	-0.144
P63073	Eif4e	0.110	0.261	0.056	0.000	-0.079	-0.067	-0.746	-0.443
P34152	Ptk2	0.297	0.524	0.207	0.000	-0.153	0.088	-0.575	-0.244
O70433	Fhl2	0.300	0.529	0.301	-2.800	0.000	1.230	-0.207	0.303
P47811	Mapk14	0.232	0.436	0.000	0.568	-0.624	-3.770	-0.803	-0.291
Q61037	Tsc2	0.494	0.759	-0.314	0.000	0.272	0.238	-0.069	0.232
P34884	Mif	0.193	0.385	-0.437	0.000	0.174	-0.562	-4.920	-1.210
Q06890	Clu	0.014	0.073	0.000	0.199	-0.686	-1.550	-2.880	-1.990
Q9JIB4	Gtf2h2	0.243	0.453	0.000	-0.382	0.134	-0.180	-1.860	-0.442

Supplementary Table 9. Senescence associated proteins detected in CD4+ T cells from old mice with or without mito-transfer. CD4+ T cells from young and old mice, and from old mice after mito-transfer were cultured for 4 h before processing for mass spectrometry analysis. Data expressed as median protein Log2 fold change of CD4+ T cells from old mice, from 3 individual old mice (paired experiment). Differentially expressed proteins (DEPs) with adj. p value > 0.5 are highlighted in orange

GO	Category	Description	Count	%	Log10(P)	Log10(q)
R-MMU-186763	Reactome	Downstream signal transduction	4	22.22	-8.2	-3.99
mmu04066	KEGG	HIF-1 signaling pathway - Mus musculus (house mouse)	5	27.78	-7.47	-3.91
GO:2000377	GO BP	regulation of reactive oxygen species metabolic process	4	22.22	-4.97	-2.31
GO:0006974	GO BP	DNA damage response	6	33.33	-4.62	-2.02
GO:1903828	GO BP	negative regulation of protein localization	4	22.22	-4.33	-1.88
GO:0062197	GO BP	cellular response to chemical stress	4	22.22	-4.11	-1.72
GO:0046434	GO BP	organophosphate catabolic process	3	16.67	-3.7	-1.39
GO:0032496	GO BP	response to lipopolysaccharide	4	22.22	-3.58	-1.3
GO:0062012	GO BP	regulation of small molecule metabolic process	4	22.22	-3.55	-1.27
GO:0001819	GO BP	positive regulation of cytokine production	4	22.22	-3	-0.85
GO:0002443	GO BP	leukocyte mediated immunity	3	16.67	-2.41	-0.37

Supplementary Table 10. The Top 20 processes of significantly downregulated DEPs in CD4+ T cells with or without mitochondrial transfer, in relation to T Cell senescence. Data generated using the Metascape analysis platform.

GO	Description	Log10(P)
R-MMU-186763	Downstream signal transduction	-8.7
WP373	IL-3 signaling pathway	-8.4
WP387	IL-6 signaling pathway	-8.4

Supplementary Table 11. The Top 3 Protein-Protein Interaction (PPI) networks of significantly downregulated DEPs in CD4+ T cells, with or without mito-transfer, in relation to T Cell senescence. Data generated using the Metascape analysis platform.

MCODE	GO	Description	Log10(P)
MCODE_1	mmu00010	Glycolysis / Gluconeogenesis - Mus musculus (house mouse)	-9.3
MCODE_1	GO:0032787	monocarboxylic acid metabolic process	-7.8
MCODE_1	mmu00620	Pyruvate metabolism - Mus musculus (house mouse)	-7.1
MCODE_2	R-MMU-186763	Downstream signal transduction	-11.7
MCODE_2	R-MMU-186797	Signaling by PDGF	-10.6
MCODE_2	WP407	Kit receptor signaling pathway	-10

Supplementary Table 12. The MCODE networks identified significantly downregulated DEPs in CD4+ T cells, with or without mito-transfer, in relation to T Cell senescence. Data generated using the Metascape analysis platform.
